# Cooking with liquefied petroleum gas or biomass and fetal growth outcomes: a multi-country randomised controlled trial

**DOI:** 10.1016/S2214-109X(24)00033-0

**Published:** 2024-04-11

**Authors:** William Checkley, Lisa M Thompson, Shakir Hossen, Laura Nicolaou, Kendra N Williams, Stella M Hartinger, Marilu Chiang, Kalpana Balakrishnan, Sarada S Garg, Gurusamy Thangavel, Vigneswari Aravindalochanan, Ghislaine Rosa, Alexie Mukeshimana, Florien Ndagijimana, John P McCracken, Anaité Diaz-Artiga, Sheela S Sinharoy, Lance Waller, Jiantong Wang, Shirin Jabbarzadeh, Yunyun Chen, Kyle Steenland, Miles A Kirby, Usha Ramakrishnan, Michael Johnson, Ajay Pillarisetti, Eric D McCollum, Rachel Craik, Eric O Ohuma, Victor G Dávila-Román, Lisa de las Fuentes, Suzanne M Simkovich, Jennifer L Peel, Thomas F Clasen, Aris T Papageorghiou, Household Air Pollution Intervention Network (HAPIN) Investigators, Gloriose Bankundiye, Dana Boyd Barr, Vanessa Burrowes, Alejandra Bussalleu, Devan Campbell, Eduardo Canuz, Adly Castañaza, Maggie Clark, Mary Crocker, Oscar De León, Ephrem Dusabimana, Lisa Elon, Juan G Espinoza, Irma Pineda Fuentes, Ahana Ghosh, Dina Goodman, Savannah Gupton, Sarah Hamid, Steven Harvey, Mayari Hengstermann, Ian Hennessee, Phabiola Herrera, Marjorie Howard, Penelope P. Howards, Lindsay Jaacks, Katherine Kearns, Jacob Kremer, Margaret A. Laws, Pattie Lenzen, Jiawen Liao, Amy Lovvorn, Jane Mbabazi, Julia N. McPeek, Rachel Meyers, J. Jaime Miranda, Erick Mollinedo, Libny Monroy, Krishnendu Mukhopadhyay, Bernard Mutariyani, Luke P. Naeher, Abidan Nambajimana, Durairaj Natesan, Azhar Nizam, Jean de Dieu Ntivuguruzwa, Ricardo Piedrahita, Naveen Puttaswamy, Elisa Puzzolo, Ashlinn Quinn, Karthikeyan D. Rajamani, Sarah Rajkumar, Rengaraj Ramasami, Alexander Ramirez, P. Barry Ryan, Sudhakar Saidam, Zoe Sakas, Sankar Sambandam, Jeremy Sarnat, Kirk Smith, Damien Swearing, Ashley Toenjes, Lindsay Underhill, Jean D Uwizeyimana, Viviane Valdes, Amit Verma, Megan Warnock, Wenlu Ye, Bonnie Young, Ashley Younger, Libny Y. Monroy-Alarcón, Adly Castañaza Gonzalez de Durante, Claudia López-Ortega, Maria F. Gonzalez, Lakshminarayanan Sowrirajan, Shanthi P. Paramanandam, K Shanmugavadivu, V Sudharsanan, Suresh Seshadri, Adhemir E. Yupanqui-Fredes, Mario Hancco-Gomez, Ronald Apaza, Juan F. Persivale-Calle, Elizabeth Quispe, Carlos Leon-Ponce, Victor Villar-Gonzales, Rebeca Andrade-Salas, Jhon E. Herrera, Luzdelia Ramos-Mamani, Yessica Lopez, Giovanna Quiza, Yadel Hinojosa, Madeluz Gomez-Quispe, Gery Frisancho-Parada, Danielle I. Mendoza-Apaza, Luz R. Quispe-Flores, Niyitegeka F Xavier, Grace Utfimana, Elie Tuzayisenga, Valens Nkurunziza

**Affiliations:** a Division of Pulmonary and Critical Care, School of Medicine, Johns Hopkins University, Baltimore, MD, USA; b Center for Global Non-Communicable Disease Research and Training, School of Medicine, Johns Hopkins University, Baltimore, MD, USA; c Global Program in Pediatric Respiratory Sciences, Eudowood Division of Pediatric Respiratory Sciences, School of Medicine, Johns Hopkins University, Baltimore, MD, USA; d Nell Hodgson Woodruff School of Nursing, Emory University, Atlanta, GA, USA; e Latin American Center of Excellence on Climate Change and Health, Universidad Peruana Cayetano Heredia, Lima, Peru; f Biomedical Research Unit, Asociación Benéfica PRISMA, Lima, Perú; g ICMR Center for Advanced Research on Air Quality, Climate and Health, Department of Environmental Health Engineering, Sri Ramachandra Institute for Higher Education and Research, Chennai, India; h Faculty of Infectious and Tropical Diseases, London School of Hygiene & Tropical Medicine, London, UK; i Centre for Maternal, Adolescent, Reproductive & Child Health, London School of Hygiene & Tropical Medicine, London, UK; j Eagle Research Center Limited, Kigali, Rwanda; k Epidemiology and Biostatistics Department, University of Georgia, Athens, GA, USA; l Center for Health Studies, Universidad del Valle de Guatemala, Guatemala City, Guatemala; m Hubert Department of Global Health, Rollins School of Public Health, Emory University, Atlanta, GA, USA; n Department of Biostatistics and Bioinformatics, Emory University, Atlanta, GA, USA; o Gangarosa Department of Environmental Health, Rollins School of Public Health, Emory University, Atlanta, GA, USA; p Department of Global Health and Population, Harvard T.H. Chan School of Public Health, Boston, MA, USA; q Berkeley Air Monitoring Group, Berkeley, CA, USA; r Division of Environmental Health Sciences, University of California at Berkeley, Berkeley, CA, USA; s Eudowood Division of Pediatric Respiratory Sciences, School of Medicine, Johns Hopkins University, Baltimore, MD, USA; t Nuffield Department of Women's & Reproductive Health, University of Oxford, John Radcliffe Hospital, Oxford, UK; u Cardiovascular Imaging and Clinical Research Core Laboratory, Department of Medicine, Washington University in St Louis, St Louis, MO, USA; v Division of Healthcare Delivery Research, MedStar Health Research Institute, Hyattsville, MD, USA; w Division of Pulmonary and Critical Care Medicine, Georgetown University, Washington, DC, USA; x Department of Environmental and Radiological Health Sciences, Colorado State University, Fort Collins, CO, USA

## Abstract

**Background:**

Household air pollution might lead to fetal growth restriction during pregnancy. We aimed to investigate whether a liquefied petroleum gas (LPG) intervention to reduce personal exposures to household air pollution during pregnancy would alter fetal growth.

**Methods:**

The Household Air Pollution Intervention Network (HAPIN) trial was an open-label randomised controlled trial conducted in ten resource-limited settings across Guatemala, India, Peru, and Rwanda. Pregnant women aged 18–34 years (9–19 weeks of gestation) were randomly assigned in a 1:1 ratio to receive an LPG stove, continuous fuel delivery, and behavioural messaging or to continue usual cooking with biomass for 18 months. We conducted ultrasound assessments at baseline, 24–28 weeks of gestation (the first pregnancy visit), and 32–36 weeks of gestation (the second pregnancy visit), to measure fetal size; we monitored 24 h personal exposures to household air pollutants during these visits; and we weighed children at birth. We conducted intention-to-treat analyses to estimate differences in fetal size between the intervention and control group, and exposure–response analyses to identify associations between household air pollutants and fetal size. This trial is registered with ClinicalTrials.gov (NCT02944682).

**Findings:**

Between May 7, 2018, and Feb 29, 2020, we randomly assigned 3200 pregnant women (1593 to the intervention group and 1607 to the control group). The mean gestational age was 14·5 (SD 3·0) weeks and mean maternal age was 25·6 (4·5) years. We obtained ultrasound assessments in 3147 (98·3%) women at baseline, 3052 (95·4%) women at the first pregnancy visit, and 2962 (92·6%) at the second pregnancy visit, through to Aug 25, 2020. Intervention adherence was high (the median proportion of days with biomass stove use was 0·0%, IQR 0·0–1·6) and pregnant women in the intervention group had lower mean exposures to particulate matter with a diameter less than 2·5 μm (PM_2·5_; 35·0 [SD 37·2] μg/m^3^*vs* 103·3 [97·9] μg/m^3^) than did women in the control group. We did not find differences in averaged post-randomisation Z scores for head circumference (0·30 *vs* 0·39; p=0·04), abdominal circumference (0·38 *vs* 0·39; p=0·99), femur length (0·44 *vs* 0·45; p=0·73), and estimated fetal weight or birthweight (–0·13 *vs* –0·12; p=0·70) between the intervention and control groups. Personal exposures to household air pollutants were not associated with fetal size.

**Interpretation:**

Although an LPG cooking intervention successfully reduced personal exposure to air pollution during pregnancy, it did not affect fetal size. Our findings do not support the use of unvented liquefied petroleum gas stoves as a strategy to increase fetal growth in settings were biomass fuels are used predominantly for cooking.

**Funding:**

US National Institutes of Health and Bill & Melinda Gates Foundation.

**Translations:**

For the Kinyarwanda, Spanish and Tamil translations of the abstract see Supplementary Materials section.

## Introduction

Biomass fuels such as wood, crop waste, and animal dung are commonly used for cooking and heating in approximately 36% of households worldwide.^1^ The incomplete combustion of these fuels results in household air pollution, which is estimated to be responsible for 2·3 million premature deaths and 91·5 million disability-adjusted life-years lost worldwide every year.^2^ Women, who are often the primary cooks at home, are the most exposed to and affected by household air pollution.^1^, ^2^ Exposure to household air pollution might adversely affect pregnancy outcomes, including higher rates of prematurity, fetal growth restriction, stillbirth, and low birthweight.^3^ A systematic review and meta-analysis conducted in 2014 estimated that exposure to household air pollution might increase the likelihood of stillbirth by 29% and reduce birthweight by an average of 86 g.^3^

Preterm birth and fetal growth restriction remain crucial problems in low-income and middle-income countries (LMICs). A systematic review estimated that 14·9 million infants were born preterm in 2010, and 60% of these preterm births were in south Asia and sub-Saharan Africa.^4^ An additional 5–10% of babies are born growth restricted globally.^5^ Preterm and growth-restricted infants are at an increased risk of neonatal death, stunting, impaired motor and neurological development, and chronic diseases in adulthood, such as type 2 diabetes, hypertension, and obesity.^4^ The extent of the effects of household air pollution on fetal growth and other maternal outcomes remains uncertain, partly due to the small number of studies conducted and inaccuracies in gestational age estimation. Major shortcomings of existing studies include inconsistent findings of an association between air pollution exposure and adverse maternal or neonatal outcomes across studies; that most data come from observational studies that are open to confounding; and that most studies measure birthweight rather than fetal growth, which might be affected differentially across gestation and levels of exposures to household air pollution. Interventions that address the primary risk factors for exposure to household air pollution might therefore hold promise for reducing the incidence of preterm labour and intrauterine growth restriction and improving the health outcomes of pregnant women and their infants in LMICs.

**Research in context**

**Evidence before this study**

About a third of households worldwide use biomass fuels for cooking and heating, resulting in household air pollution. Studies show a link between such pollution and adverse pregnancy outcomes, including fetal growth restriction and low birthweight. We reviewed PubMed, MEDLINE, Embase, and the Web of Science database on Jan 13, 2023, using search terms related to exposure (including “home air pollution”, “household air pollution”, “solid fuel”, “biomass”, and “cooking”) and pregnancy outcomes relating to fetal growth (including “adverse pregnancy outcomes”, “birthweight”, “small for gestational age”, “intrauterine growth restriction”, and “fetal growth restriction”) for studies published between Jan 1, 2000, and Jan 13, 2023. The available evidence suggests that exposure to household air pollution is associated with lower birthweight. However, there are three major shortcomings of existing studies. First, the results are not consistent across studies. Second, most studies have examined birthweight rather than fetal growth during pregnancy; because fetal growth is a dynamic process, it might be affected differentially across gestation and across levels of exposures to household air pollution. Finally, most data come from observational studies that are subject to confounding by socioeconomic or other factors.

**Added value of this study**

We assessed the effects of reducing personal exposures to household air pollution on fetal growth in a randomised controlled trial conducted in 3200 households (with one pregnant woman per household) in ten resource-poor settings in Jalapa (Guatemala), Tamil Nadu (India), Kayonza (Rwanda), and Puno (Peru). We implemented a multi-component intervention of a liquefied petroleum gas (LPG) stove, continuous and free fuel delivery, and behavioural messaging. We previously found that the intervention did not affect birthweight. Here, we assessed fetal growth throughout gestational age using standardised ultrasound measures to identify crucial windows of early development.

**Implications of all the available evidence**

Despite high adherence to the intervention during pregnancy and a large reduction in prenatal exposures to particulate matter less than 2·5 μm (PM_2·5_) in the intervention versus control groups, we did not find evidence of an effect on any fetal growth outcomes in the intention-to-treat analysis. Additionally, no strong associations were observed between personal exposure to pollutants and fetal growth outcomes in the exposure–response analyses. The findings of this randomised trial suggest that the associations between higher exposure to household air pollution and reduced fetal growth seen in previous studies were likely to be due to confounding. Use of LPG-based interventions as a strategy to specifically attenuate fetal growth restriction is not warranted.

We aimed to assess the health effects of a liquefied petroleum gas (LPG) cooking intervention aimed at reducing personal exposures to household air pollution.^6^ We previously reported that the intervention had no significant impact on birthweight.^7^ However, we also asserted that birthweight alone is a crude single measure of size at birth, and that concurrent longitudinal assessment of fetal growth patterns is useful to identify important windows of early development and relevant phenotypes during childhood growth and neurodevelopment.^8^ We therefore aimed to analyse fetal growth data collected by ultrasound, to evaluate the effects of a multi-component LPG cooking intervention as a strategy to mitigate household air pollution and reduce the risk of fetal growth restriction.

## Methods

### Study design and participants

The Household Air Pollution Intervention Network (HAPIN) trial was an open-label randomised controlled trial conducted across 3200 households in ten resource-limited settings in Jalapa, Guatemala (one setting); Tamil Nadu, India (two settings); Puno, Peru (six settings); and Kayonza, Rwanda (one setting). These ten settings constituted the randomisation strata and were purposefully selected to represent a diversity of characteristics expected to influence intervention effects, including altitude, population density, cooking practices, baseline pollution levels, and sources of pollution other than cooking.^6^ We selected countries across different continents with a high prevalence of biomass fuel use, with previous experience in conducting research studies on household air pollution, and with low ambient air pollution in rural settings.

All pregnant women were offered a gestational age assessment via ultrasound at enrolment; those with pregnancies at gestational ages of 9–19 weeks (baseline visit), based on either fetal crown rump length or head circumference and femur length, could be included in the trial. We calculated gestational age based on fetal crown rump length if this measurement was less than 85 mm^9^ or by fetal head circumference and femur length if crown rump length was 85 mm or longer.^10^ When the head circumference and femur length were used for dating, we also measured abdominal circumference. These dating scans were used to ensure the pregnancy was viable (presence of fetal heart rate and confirmation of an intrauterine pregnancy), to diagnose a multiple pregnancy, and to calculate gestational age.^11^

The study protocol was reviewed and approved by institutional review boards or ethics committees at Emory University (00089799), Johns Hopkins University (00007403), Sri Ramachandra Institute of Higher Education and Research (IEC-N1/16/JUL/54/49), the Indian Council of Medical Research – Health Ministry Screening Committee (5/8/4-30/[Env]/Indo-US/2016-NCD-I), Universidad del Valle de Guatemala (146-08-2016/11-2016), the Guatemalan Ministry of Health National Ethics Committee (11-2016), A.B. PRISMA (CE3571.16), the London School of Hygiene & Tropical Medicine (11664-5), the Rwanda National Ethics Committee (No. 357/RNEC/2018), and Washington University in St Louis (201611159). All participants provided written informed consent. Details of the trial have been published previously^6^ and the trial is registered on ClinicalTrials.gov (NCT02944682).

### Randomisation and masking

The intervention comprised distribution of an LPG stove, continuous and free fuel delivery, and behavioural messaging. Controls received periodic compensation, which varied by international research centres (IRCs), to offset the economic benefit of the intervention and were offered an LPG stove and 1 month of fuel or another household item at endline. Participants were randomly assigned in a 1:1 ratio stratified by setting (ten strata in total) in permuted blocks of 2 and 4 to either receive the intervention or continue cooking primarily with biomass fuels. The Data Management Core developed the randomisation scheme by use of an in-house computerised algorithm. Each of the four in-country international research centres recruited 800 pregnant women aged 18–34 years (total 3200 pregnant women) with singleton pregnancies at 9–19 weeks of gestation, as confirmed by ultrasound. Only one pregnant woman per household was allowed to participate.

The field team could not be masked to the intervention; however, the statistical plan was prepared before unmasking and analysts (SH and WC) were masked to the allocation group.

### Procedures

We used the same portable ultrasound (Edge, Fujifilm/SonoSite, Bothell, WA, USA) with curvilinear 5–2 MHz abdominal transducers at the four IRCs. Ultrasound teams consisted of three to four experienced sonographers in each IRC. Imaging criteria, methods, and quality control methods followed INTERGROWTH-21st guidelines.^12^, ^13^, ^14^ Representatives from each country went to Washington University in St Louis, Missouri, for a 10-day intensive training and standardisation programme,^15^ which has previously been shown to improve the consistency of measurements even for experienced sonographers.^14^ All sites also received an ultrasound simulation probe and programme for practice (SonoSim, Santa Monica, CA, USA). Sonographers received additional in-country training by physicians and were given comprehensive protocols, instructions on appropriate calliper placement, imaging checklists, and reference cards with sample images of obstetric examinations. Additional training on image transmission, identification, and storage was also provided by Trice, a cloud-based server and image management system (Trice Imaging, Del Mar, CA, USA).

A lead sonographer was selected for each site on the basis of their ultrasound skills and motivation to perform the research scans. Their primary role was to oversee local training and standardisation; they were also responsible for ensuring data upload and training staff locally. Each sonographer was required to perform 25 certification scans, which were reviewed by the Ultrasound Core Laboratory at Washington University in St Louis and Oxford University for quality control. The Ultrasound Core Laboratory checked each scan for protocol adherence and quality. Sonographers who successfully submitted 25 studies with a quality score greater than 80% were certified to scan for the trial. Those not meeting this criterion underwent retraining, and further scans of sufficient quality had to be submitted for the sonographer to be re-certified. If new sonographers were recruited to the team due to staffing changes, they underwent the same standardisation process as above, including completing training from a local sonographer, or, if required, from an external sonographer and then submitting 25 scans for review by the Ultrasound Core Laboratory before certification. When necessary, Ultrasound Core Laboratory members performed site visits to provide additional and refresher training. Sonographers received one-on-one support to help them address their individual needs, such as ensuring they obtained the correct image plane or manoeuvring the probe. Additionally, support was provided for the management of the images to ensure data were entered correctly and all data and images were uploaded to the database promptly.

Ultrasound data were transferred from the IRCs to Trice, and the associated measurements for each image were then entered. Once the sites completed their data entry, the images were moved to a folder that allowed data to be accessed by the Ultrasound Core Laboratory. All data were then transferred to Intelligent Ultrasound (Abingdon, Oxfordshire, UK), where quality assurance and analysis was performed with a system to automatically sort images into ultrasound views, which can then be assigned to expert sonographers for grading. The sonographer grading process was achieved with a secure website that allowed sonographers to view and grade images remotely.

An a-priori plan dictated that a varying proportion of images would be assessed for quality as the project evolved. At the beginning of the project, 100% of the images were reviewed to ensure quality images were being obtained. In July, 2019, the number of images reviewed was reduced to a random 10% of scans, as the team was confident that the sonographers could acquire good quality images. Of the 10% of images reviewed by the team of sonographers, a 10% sample was re-assessed by the HAPIN ultrasound team to ensure reviewing sonographers were conducting quality assessment correctly by assessing agreement.

Each set of scan data was reviewed following INTERGROWTH-21st methods.^13^, ^14^ Images had criteria that had to be achieved, with a score of 1 point given if a criterion was met and 0 if it was not met (total score of 5 for crown rump length and femur length, and total score of 6 for head circumference and abdominal circumference). An individual ultrasound scan's score was computed as the average score from all required images. If the mean scan score was 80% or higher, it was deemed adequate; if it was less than 80%, the scan was flagged for review. If the average score for an IRC fell below 80%, scores were stratified according to sonographer and failing sonographers were identified and retrained. To ensure protocol adherence, one passing image of each requested type was required for a scan to pass. If both images failed, it was marked as a protocol deviation. If any of the additional measures were missed, the sonographer was notified and monitored for protocol adherence. If a sonographer had more than three protocol deviations, they were asked to repeat training and be re-certified. For these analyses, we used data from all ultrasounds regardless of whether they were adequate or not.

We used temperature loggers as stove use monitors on all biomass-burning stoves of intervention households to monitor adherence to the intervention.^16^ We conducted additional home visits to deliver LPG fuel tanks to intervention homes and provided behavioural reinforcement in response to identified biomass stove use.^17^ We provide a detailed description of exposure assessment methods in appendix 4 (p 4). Briefly, we measured 24 h personal exposures to particulate matter with a diameter less than 2·5 μm (PM_2·5_) in aerodynamic diameter (Enhanced Children MicroPEM [ECM]; RTI International, Chapel Hill, NC, USA) and carbon monoxide gas (EL-USB-300; Lascar Electronics, Erie, PA, USA) using portable, battery-operated monitors. Black carbon was measured on the PM_2·5_ filters from the ECM by use of a SootScan Model OT21 transmissometer (Magee Scientific, Berkeley, CA, USA). We conducted exposure measurements three times during pregnancy—at the baseline, first pregnancy, and second pregnancy visit windows as defined above. Pregnant women wore the devices in a breathable pocket in clothing provided to them by our project team, either an apron or a lightweight vest, and were asked to hang the clothing near their beds while sleeping or bathing.

We followed up pregnant women and their fetuses until birth, as well as their infants for the first year of life to assess for health, growth, and development.

### Outcomes

Primary outcomes for this trial were birthweight, childhood stunting at 12 months of age, incidence of severe pneumonia in the first year of life, and blood pressure in older adult women.^6^ Details of how birthweight was measured in this trial are reported elsewhere.^7^ Fetal growth was a prespecified secondary outcome. Ultrasound scans were conducted at 24–28 weeks (first pregnancy visit) and 32–36 weeks (second pregnancy visit) to assess fetal growth.

We measured head circumference, abdominal circumference and femur length, placenta location, fetal presentation, and estimated fetal weight at the first pregnancy visit and second pregnancy visit. We calculated Z scores for head circumference, femur length, abdominal circumference, estimated fetal weight, and birthweight at the gestational age that the child was born with the INTERGROWTH-21st reference equations.^18^

### Statistical analysis

We sought to randomly assign 1600 participants to the intervention group and 1600 to the control group. The main analysis was done in the intention-to-treat population, with Z scores of fetal growth outcomes (head circumference, abdominal circumference, femur length, and estimated fetal weight or birthweight) modelled as continuous variables. As fetal growth was considered a secondary outcome, there was no formal sample size calculation. Given that we have four fetal growth outcomes, the threshold for statistical significance was set at 0·0125 and we presented 98·75% CIs to control the familywise type I error rate at 0·05. Estimated fetal weight and birthweight are thought to represent a continuum in the gestational age timescale.^19^ We therefore combined estimated fetal weight Z scores and birthweight-for-gestational-age Z scores into a single outcome variable in our regression models. We excluded fetal measurements of newborns with congenital abnormalities.

We used multivariable linear regressions with the post-randomisation Z scores as the outcome and the trial group assignment as the main covariate (with the control group as the reference), adjusted for randomisation strata. We first ran regressions to estimate the difference in fetal growth Z scores for the first pregnancy visit and second pregnancy visit separately and then ran regressions to estimate differences in the average Z scores of the first pregnancy visit and second pregnancy visit (if only one visit was available, we used the measurement in that visit as the average). We also ran regressions for birthweight-for-gestational-age Z scores at birth separately and for the averaged estimated fetal weight and birthweight-for-gestational-age Z scores between the first pregnancy visit and birth. We considered a difference in Z score of 0·2 SDs as a clinically significant difference based on the work by Sudfeld and colleagues,^20^ who showed that an effect size of 0·2 SDs is a commonly observed magnitude of change in anthropometric indices due to nutrition-specific and nutrition-sensitive interventions.

We also modelled the Z scores obtained at the baseline visit, first pregnancy visit, and second pregnancy visit (and at birth for estimated fetal weight Z scores and birthweight-for-gestational-age Z scores) using a generalised additive model^21^ with a tensor smooth for gestational age stratified by trial group assignment (with the control group as the reference) and time of randomisation above or below the median (ie, for this analysis, this corresponded to a gestational age <15·3 weeks or ≥15·3 weeks) and adjusted for randomisation strata. We estimated median differences in Z scores by gestational age between participants in the intervention and control group at 25 weeks of gestation (median gestational age for the first pregnancy visit) and 33 weeks of gestation (median gestational age for the second pregnancy visit) and at 40 weeks for estimated fetal weight, stratified by gestational age at randomisation. We accounted for heterogeneity across participants using random intercepts and slopes.

Finally, we also conducted exposure–response analyses using the longitudinal fetal growth Z scores as continuous variables and 24 h personal exposures to PM_2·5_, black carbon, and carbon monoxide as the main covariate, also continuous, obtained at the baseline visit, first pregnancy visit, and second pregnancy visit. For the exposure–response analyses, growth measurements obtained at the baseline visit were paired with pollutant concentrations measured at the baseline visit for regression; those obtained at the first pregnancy visit were paired with the average of pollutant concentrations obtained at the baseline visit and first pregnancy visit; those obtained at the second pregnancy visit were paired with the average of pollutant concentrations obtained at the baseline visit, first pregnancy visit, and second pregnancy visit; and birthweight-for-gestational-age Z scores were paired with average pollutant concentrations across the baseline visit, first pregnancy visit, and second pregnancy visit. Means and SDs of Z scores by visit and their paired pollutant concentrations in quartiles were tabulated. Generalised additive models were used with the Z scores as the outcome and the following covariates selected a priori and consistent with variables included in the exposure–response analysis for birthweight:^22^ tensor surface smooth for the interaction between pollutant and gestational age, principal components analysis-derived socioeconomic status index as described in appendix 4 (p 5), maternal age, nulliparity, minimum diet diversity for women categorised as either high (≥5) or low (<5),^7^ maternal education, altitude-adjusted maternal haemoglobin,^23^ and self-reported exposure to second-hand smoke. We accounted for heterogeneity across participants using random intercepts and slopes. To ensure comparability with the intention-to-treat analysis, we computed differences in Z scores at 25 and 33 weeks (and at 40 weeks for estimated fetal weight) across the interquartile differences of the paired pollutant concentrations and set the threshold for statistical significance at 0·0125.

We conducted statistical analyses in R (version 4.2.1; alias Funny-Looking Kid). We present our statistical analysis plan, code, and output in appendix 4 (pp 21–210).

### Role of the funding source

The sponsors did not have any role in study design; in the collection, analysis, or interpretation of data; in the writing of the report; or in the decision to submit the manuscript for publication.

## Results

**Figure 1:**
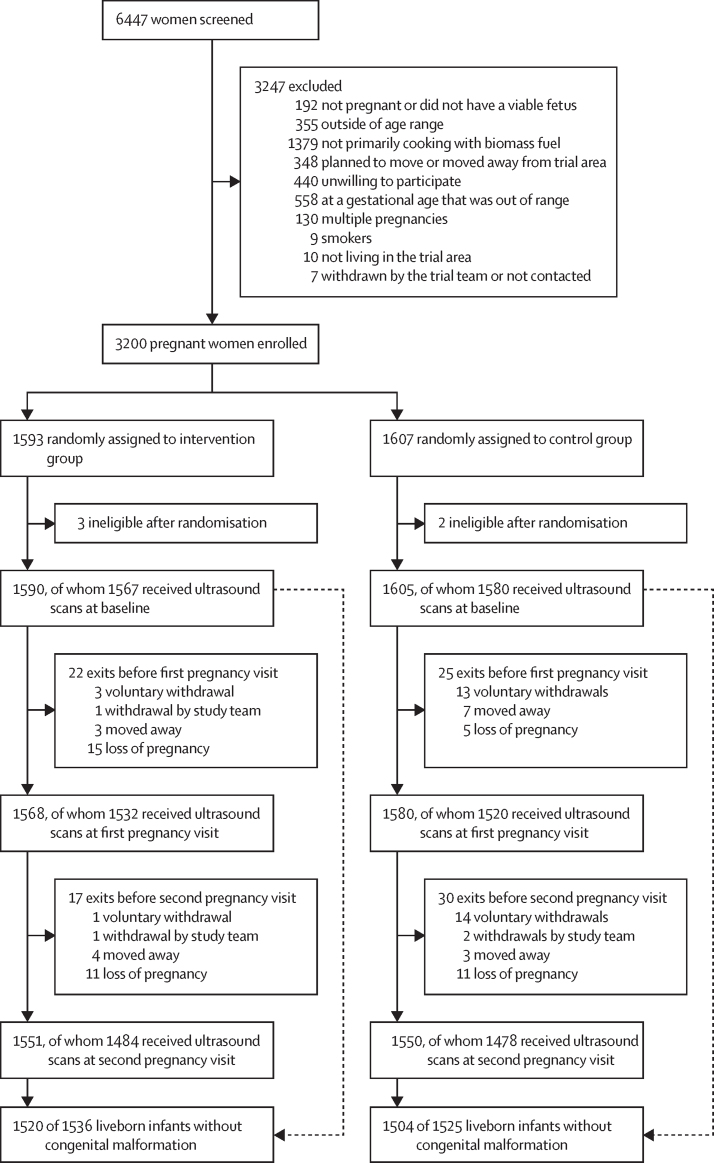
Trial profile Participants could have more than one reason for exclusion (ie, categories are not mutually exclusive).

Between May 7, 2018, and Feb 29, 2020, we randomly assigned 3200 pregnant women to the intervention or control group (figure 1); the mean gestational age was 14·5 (SD 3·0) weeks and mean maternal age was 25·6 (4·5) years. We randomly assigned 1593 pregnant women to the intervention group and 1607 pregnant women to the control group. Five participants were ineligible after randomisation. We also excluded 48 children with congenital malformations detected either during fetal ultrasound or at birth. We assessed 3147 (98·3%) pregnant women for fetal dating at baseline; we obtained fetal growth measurements in 3052 (95·4%) pregnant women at the first pregnancy visit and in 2962 (92·6%) at the second pregnancy visit. The last ultrasound for fetal growth was conducted on Aug 25, 2020. The percentage of fetal ultrasounds obtained at each exact week of gestational age and stratified by visit is presented in appendix 4 (p 7).

In total, 3024 children were born alive without congenital abnormalities and contributed data to birthweight-for-gestational-age Z scores. Among participants who had fetal growth measurements obtained at either the first pregnancy visit or second pregnancy visit, there were no differences in sociodemographic characteristics between pregnant women in the intervention and control groups (table 1). The mean gestational age at baseline was 14·7 (SD 3·0) weeks in the intervention group and 14·4 (3·1) weeks in the control group. Based on data on stove use monitors, the median proportion of days that intervention households used their biomass stove was 0·0% (IQR 0·0–1·6) during pregnancy. The intervention reduced prenatal, averaged, post-randomisation personal exposures to PM_2·5_, black carbon, and carbon monoxide (table 2).

**Table 1:** Baseline characteristics of 2811 participants with fetal growth measurements

		**Intervention group (n=1396)**	**Control group (n=1376)**
Demographics			
	Maternal age in years	25·5 (4·4)	25·6 (4·6)
	Maternal height in cm	152·4 (6·3)	152·2 (6·1)
Fetal sex			
	Female	684 (49·0%)	692 (50·3%)
	Male	712 (51·0%)	684 (49·7%)
Socioeconomic status indicators			
	Bank account	546 (39·1%)	462 (33·6%)
	Television	653 (46·8%)	625 (45·4%)
	Radio	686 (49·1%)	654 (47·5%)
	Cellular telephone	1215 (87·0%)	1187 (86·3%)
	Maternal education, number of years	8·0 (3·7)	7·8 (3·5)
	Number of people living in the house	4·3 (2·1)	4·3 (2·0)
	Socioeconomic wealth index	0·1 (1·0)	0·2 (1·0)
Pregnancy factors			
	Gestational age, weeks	14·7 (3·0)	14·4 (3·1)
	Nulliparous	526 (37·7%)	477 (34·7%)
	Hypertension during pregnancy	25 (1·8%)	28 (2·0%)
	Preterm birth	73 (5·2%)	68 (4·8%)
Minimum dietary diversity score for women			
	Low (<4)	740 (53·0%)	756 (54·9%)
	Medium (4–5)	459 (32·9%)	472 (34·3%)
	High (>5)	196 (14·0%)	147 (10·7%)
Household food insecurity			
	Food secure	788 (56·4%)	695 (50·5%)
	Mild (1–3)	383 (27·4%)	405 (29·4%)
	Moderate or severe (4–8)	205 (14·7%)	254 (18·5%)
Smoking in the household		99 (7·1%)	126 (9·2%)

Data are n (%) or mean (SD).

**Table 2:** Personal exposures to PM_2·5_, black carbon, and carbon monoxide measured over 24 h at baseline and after randomisation

	**Intervention group**		**Control group**	
	Mean (SD)	n	Mean (SD)	n
**Personal exposures to PM**_2·5_**(μg/m^3^)**				
Prenatal maternal exposures at baseline*	119·5 (133·6)	1352	111·9 (110·9)	1352
Average prenatal maternal exposures after randomisation†	35·0 (37·2)	1437	103·3 (97·9)	1409
**Personal exposures to black carbon (μg/m^3^)**				
Prenatal maternal exposures at baseline*	12·6 (10·9)	1223	12·4 (9·2)	1211
Average prenatal maternal exposures after randomisation†	4·1 (5·6)	1413	11·2 (9·3)	1376
**Personal exposures to carbon monoxide (ppm)**				
Prenatal maternal exposures at baseline*	2·7 (4·5)	1384	2·3 (4·0)	1375
Average prenatal maternal exposures after randomisation†	0·7 (1·2)	1450	2·2 (3·6)	1430

PM_2·5_=fine particulate matter.

* Obtained at enrolment in pregnant women participants before randomisation.

† Obtained in the first or second visits during pregnancy via a direct method (ie, pregnant women wore the monitors).

**Figure 2:**
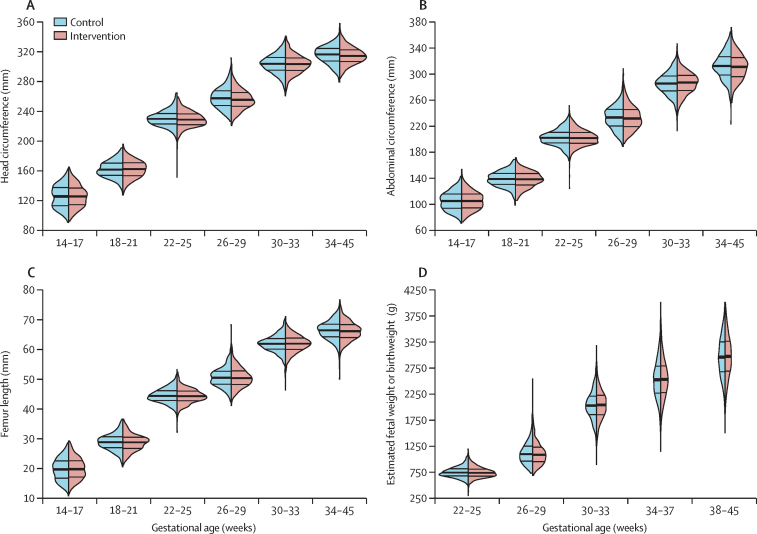
Violin plots for head circumference (A), abdominal circumference (B), femur length (C), and estimated fetal weight or birthweight (D) by categories of gestational age and intervention group Violin plots are hybrids of boxplots and density plots, and are used here to visualise the distribution of fetal growth outcomes. The thick band represents the 50th percentile, and the thinner bands represent the 25th and 75th percentiles.

**Figure 3:**
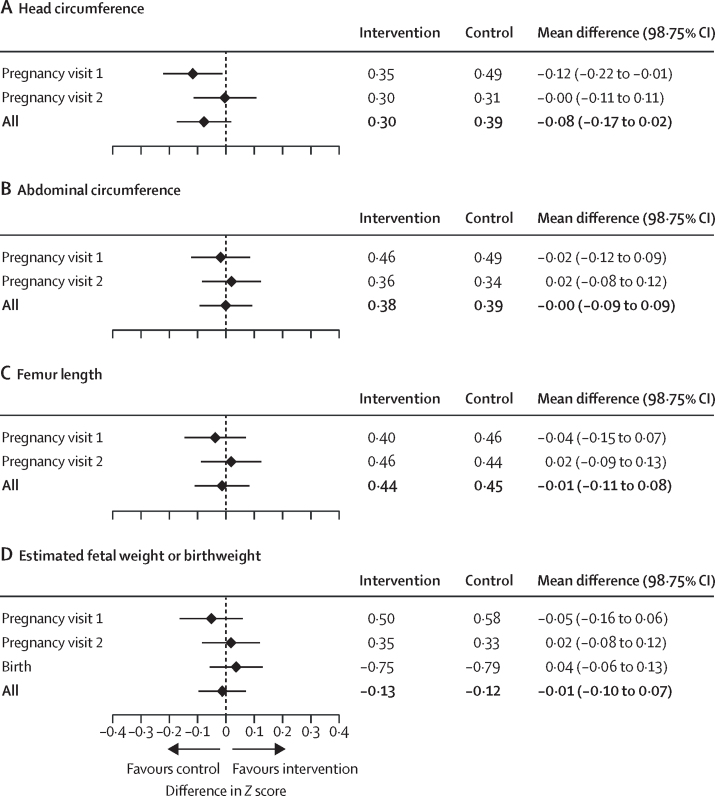
Results of intention-to-treat analyses for the differences in Z scores of head circumference (A), abdominal circumference (B), femur length (C), and estimated fetal weight or birthweight (D) by visit, displayed as a forest plot Stacked panels represent one of the four fetal growth outcomes. The difference was estimated from multiple linear regressions of fetal growth Z scores as a function of intervention group adjusted for randomisation strata at each visit, and multiple linear regressions of fetal growth Z score averages across visits as a function of intervention group adjusted for randomisation strata. Values lower than 0 indicate benefit to the control group, whereas values higher than 0 indicate benefit to the intervention group. The estimated averaged post-randomised means by trial group and the mean differences between groups (intervention minus control) are displayed with a diamond and the corresponding 98·75% CIs as horizontal lines. The first pregnancy visit was at 24–28 weeks of gestation; and the second pregnancy visit was at 32–36 weeks of gestation. We did not find differences in averaged post-randomisation Z scores between intervention participants and controls for head circumference (0·30 *vs* 0·39; p=0·04), abdominal circumference (0·38 *vs* 0·39; p=0·99), femur length (0·44 *vs* 0·45; p=0·73), and estimated fetal weight or birthweight (–0·13 *vs* –0·12; p=0·70).

We did not observe any differences between trial groups in fetal growth outcomes (figure 2) or averaged Z scores (appendix 4 p 8) at any gestational age category or averaged Z scores at any visit (appendix 4 p 6), except for a higher head circumference Z score at 26–29 weeks’ gestation and at the first pregnancy visit, favouring participants in the control group over those in the intervention group. We also did not observe any site-specific differences between trial groups for fetal growth by gestational age categories (appendix 4 pp 9–12) or averaged Z scores by gestational age categories (appendix 4 pp 13–16). We summarise the results of the intention-to-treat analyses by visit in figure 3. Specifically, we did not find any differences in fetal growth measurements between groups at any visit or when combined, except for a head circumference Z score that was –0·12 SDs smaller in participants in the intervention group than in those in the control group. All differences in Z scores between groups at any visit were less than 0·2 SD. We also plotted the estimated mean differences in fetal growth Z scores at 25 weeks and 33 weeks of gestation (and at 40 weeks for estimated fetal weight Z scores and birthweight-for-gestational-age Z scores) between participants in the intervention and control groups stratified by gestational age at randomisation (appendix 4 p 17). There were no differences in Z scores by gestational age at randomisation between groups at 25 weeks or 33 weeks of gestation.

**Figure 4:**
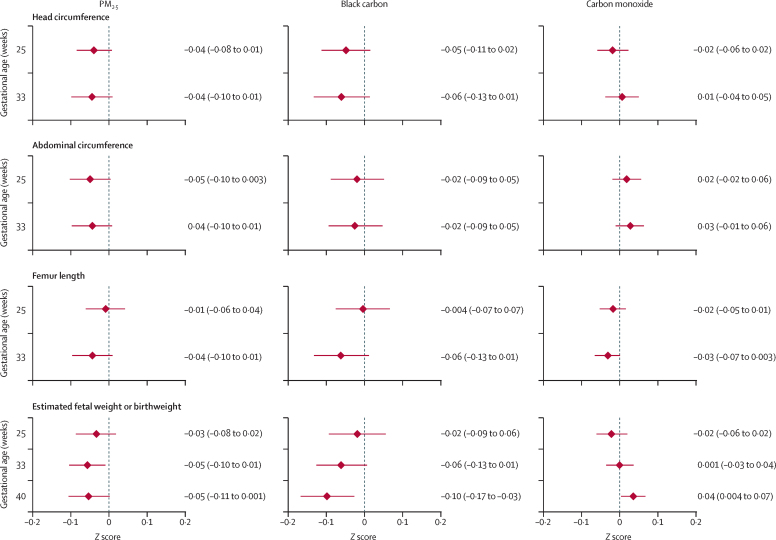
Results of exposure–response analyses of head circumference, abdominal circumference, femur length, and estimated fetal weight or birthweight Z scores scaled to the interquartile difference of PM_2·5_, black carbon, and carbon monoxide and estimated at 25, 33, and 40 weeks (for the estimated fetal weight Z score only) Differences were estimated from generalised additive mixed models (by pollutant) of the fetal growth Z scores as a function of a smooth surface comprising gestational age and pollutant concentrations and adjusted for potentially confounding variables (socioeconomic status, maternal age, nulliparity, diet diversity, maternal education, maternal haemoglobin, and second-hand smoke). Each of the 12 panels corresponds to the combination of fetal growth Z score and personal exposures to each pollutant. Values lower than 0 indicate a lower Z score for the interquartile difference of each pollutant (ie, household air pollution worsens fetal growth Z scores), whereas values higher than 0 indicate higher Z score for the interquartile difference of each pollutant. The estimated mean differences are displayed with a diamond and corresponding 98·75% CIs as horizontal lines. PM_2·5_=fine particulate matter.

We display the mean fetal growth Z scores by quartiles of personal exposures to pollutants (PM_2·5_, black carbon, and carbon monoxide) in appendix 4 (p 18). In the exposure–response analysis, we did not identify any consistent trends in Z scores for any of the fetal growth outcomes across quartiles for any of the measured pollutants, except for an association between lower Z scores of estimated fetal weight and higher personal exposures to PM_2·5_ (scaled to the interquartile difference and ranging from 23·3 μg/m^3^ to 98·4 μg/m^3^) at 33 weeks and black carbon at 40 weeks (figure 4). All differences in Z scores were less than 0·2 SD.

## Discussion

We conducted a multi-country randomised controlled trial to evaluate the impact of an LPG stove, continuous and free fuel distribution, and ongoing behavioural messaging intervention on gestational development during pregnancy. Our study found that adherence to the intervention during pregnancy was high^20^ and that the intervention reduced prenatal PM_2·5_ exposures by 66% (from 103·3 μg/m^3^ to 35·0 μg/m^3^)^24^ compared with those in the control group. However, despite this high adherence and concomitant reductions in prenatal household air pollution exposures, we did not find evidence of an effect of the intervention on any fetal growth outcomes in the intention-to-treat analysis, nor a consistent association between personal exposure to pollutants and fetal growth outcomes in the exposure–response analyses. Our results are consistent with previous analyses of birthweight (the primary outcome) in the HAPIN trial.^7^, ^22^

Although several observational studies have linked household air pollution to low birthweight and other adverse maternal outcomes,^25^, ^26^, ^27^ to the best of our knowledge no studies have examined the effect of household air pollution on fetal growth outcomes other than a randomised controlled trial of approximately 300 participants in Ibadan, Nigeria.^28^ Like our trial, the trial in Nigeria randomly assigned pregnant women between 16–18 weeks of gestation to receive a clean fuel stove (using ethanol instead of LPG) or continue with usual cooking practices. The trial in Nigeria also conducted serial fetal growth assessment at 13, 20, 26, 30, 34, and 38 weeks of gestation. The investigators did not find a difference in the intention-to-treat population for fetal growth or an association in the exposure–response analysis in the subset of around 20% of participants with a 72 h personal exposure to PM_2·5_ in the second or third trimesters.

We observed no noticeable difference in fetal growth outcomes between the trial groups, in line with our earlier findings that the intervention did not affect birthweight in an intention-to-treat analysis.^7^ However, the results of our exposure–response analyses differ from our previous findings of a small but statistically significant association between prenatal pollutant exposures and birthweight.^22^ Our analysis has the advantage of modelling serial measurements of fetal growth outcomes that allowed for a more comprehensive longitudinal analysis of this relationship. Interquartile differences in personal exposures to PM_2·5_ or black carbon yielded estimated fetal weight SDs in the range of 0·03 to 0·05, well below a clinically meaningful difference of 0·2 SDs.^20^

Our study has several strengths. First, we conducted a large, randomised controlled trial in four diverse, resource-poor settings around the world. Second, we ensured accurate estimation of gestational age for all participants. Third, we used a standardised protocol, using the same ultrasound devices across settings to measure fetal growth and ensuring high quality imaging throughout the study. Fourth, we conducted three fetal ultrasound measurements, allowing for longitudinal measurement of growth. Fifth, we conducted three high quality assessments of personal exposures to household air pollutants at the same timepoints as the fetal ultrasounds. Finally, our study was conducted in rural areas with low ambient air pollution and where daily use of biomass fuels could have the greatest effect on health outcomes. However, we also caution against assuming that our findings are generalisable to other regions around the world.

The absence of an observed effect might be attributed to a few potential shortcomings of our trial. First, the intervention might not have started early enough in pregnancy: a reduction in exposures to household air pollution might need to occur closer to, if not before, conception. With a median gestational age of enrolment at 14 weeks, in-utero growth trajectories might have already been established, given the importance of pre-conception health and epigenetic factors. Future studies should consider the effect of an LPG intervention on subsequent pregnancies. Second, although our trial participants showed high adherence to the intervention, and we observed an important exposure contrast between trial groups, the average prenatal exposures were still three times higher than current recommended guidelines for air quality.^29^ Third, LPG combustion byproducts, such as nitrogen dioxide, might also adversely affect fetal growth outcomes. Notably, nitrogen dioxide is also a byproduct of biomass combustion and although LPG stoves have lower nitrogen dioxide emissions when compared to biomass, if unvented they are higher than established safe guidelines.^30^ Fourth, our study might have been underpowered to detect small effects on fetal growth outcomes, especially at the 0·0125 level of significance. Nevertheless, the absence of any clinically meaningful differences in this large study suggests that the link between household air pollution and poor fetal growth might be less pronounced than previously thought, and it is possible that observational studies overestimated this link due to confounding with socioeconomic or other factors. Fifth, although our exposure–response analysis accounted for several confounders, there might still be unmeasured or residual confounding factors that could influence fetal growth outcomes. Finally, a formal sample size calculation for fetal growth was not done because this was a secondary outcome.

In summary, cooking with unvented LPG stoves did not affect fetal growth when compared with cooking with biomass fuel. We also did not find an association between fetal growth outcomes and personal exposures to specific household air pollutants. The adverse role of air pollution in poor birthweight outcomes is well established; however, the findings of this large trial carried out in populations living in resource-limited settings does not support use of unventilated LPG interventions as a strategy to reduce poor fetal growth.

## Data sharing

Datasets that enable others to reproduce the reported findings will be made openly available immediately after publication. To protect the confidentiality of study participants, identifiable information will be excluded from all datasets that will be made available. These will be accompanied by documentation necessary to understand the content (such as data dictionaries or metadata descriptions). All source datasets will be made available through the corresponding authors, subject to ethical, data protection, and other obligations being addressed. At the same time, they will be posted to the results section of the registry. Data will be publicly accessible and can be downloaded by anyone without restrictions.

## Declaration of interests

LW reports a grant from the US National Institute of Environmental Health Sciences. All other authors declare no competing interests.

## Supplementary Materials

Kinyarwanda translation of the abstract

Spanish translation of the abstract

Tamil translation of the abstract
