# Correction to *Lancet Glob Health* 2024; 12: e815–25

**DOI:** 10.1016/S2214-109X(24)00223-7

**Published:** 2024-05-29

*Checkley C, Thompson LM, Hossen S, et al. Cooking with liquefied petroleum gas or biomass and fetal growth outcomes: a multi-country randomised controlled trial.* Lancet Glob Health *2024; **12:** e815–25*—In figure 4 of this Article, for the exposure–response analysis of estimated fetal weight or birthweight and personal exposure to PM_2·5_, the 98·75% CI of the Z score at 33 weeks should be –0·10 to –0·01. And the following statement has been added after the first sentence of the Acknowledgments section: “This research represents the NIH's contribution to the Global Alliance for Chronic Diseases (GACD) coordinated call for research on prevention and management of chronic lung diseases for 2016.” These corrections have been made as of May 29, 2024.

