# Supplementary material for: Cooking with liquefied petroleum gas or biomass and fetal growth outcomes: a multi-country randomised controlled trial
Source: Lancet Glob Health. 2024 Apr 11;12(5):e815–25. doi: 10.1016/S2214-109X(24)00033-0 (PMC11027158; doi:10.1016/S2214-109X(24)00033-0)
Supplement: Kinyarwanda translation of the abstract [file mmc1.pdf]

THE LANCET
Global Health

Supplementary appendix 1

This translation in Kinyarwanda was submitted by the authors and we reproduce it as supplied. It has not been peer reviewed. *The Lancet*'s editorial processes have only been applied to the original in English, which should serve as reference for this manuscript.

Uku gushyirwa mu Kinyarwanda byatanzwe n'abanditsi kandi tubisubirwamo nk'uko byatanzwe. Ntabwo byasuzumwe ntabwo byarebewe hamwe. Ibikorwa by'ubwanditsi bwa Lancet byakoreshejwe gusa mu cyongereza cy'umwimerere, bigomba kugenderwaho muri iyi nyandiko (manuscript).

Supplement to: Checkley W, Thompson LM, Hossen S, et al. Cooking with liquefied petroleum gas or biomass and fetal growth outcomes: a multi-country randomised controlled trial. *Lancet Glob Health* 2024; **12:** e815–25.

Kinyarwanda Abstract

INCAMAKE

Intangiriro: Ihumana ry'umwuka wo mu rugo rishobora gutuma umwana adakura mu gihe cyo gutwita. Twagerageje kumenya niba ubufasha bwo gutanga gazi ikomoka kuri peteroli (LPG) mu kugabanya uguhura n'umwuka wanduye wo mu rugo mu gihe cyo gutwita niba bishobora kongera ubunini bw'umwana batwite.

Uburyo byakozwe:
Ubushakashatsi butanga inyunganizi bwa HAPIN yari amahitamo asesuye yakorewe ahantu icumi h'aba abafite ubishobozi buke muri muri Guatemala, u Buhinde, Peru no mu Rwanda. Abagore batwite bari hagati y'imyaka 18 na 34 (batwite ibyumweru 9-19) bashyizwe mu kigero cya 1:1 kugira ngo bahabwe ishyiga rya LPG, gukomeza guhabwa ibicanwa no guhabwa ubutumwa guhindura imyitwarire cyangwa gukomeza gukoresha ibicanwa bikomoka ku bimera mu gihe cy'amezi 18.Twakoze kandi ibizamini bya ekogarafi ku isura ry'ibanze, hagati y'ibyumweru 24 na 28 (Isura rya mbere mu gihe cyo gutwita) n'ibyumweru 32-36 (Isura rya kabiri mu gihe cyo gutwita) kugira ngo dupime ubunini bw'umwana uri munda, twagenzuye kwandura k'umwuka wo mu rugo ku muntu mu masaha 24 muri ayo masura kandi tunapima ibiro by'umwana ku munsi yavutseho. Twakoze isesengura rigamije gusuzuma itandukaniro ry'ubunini bw'umwana uri mu nda hagati y'abagize uruhare mu bushakashatsi bari mu itsinda rihabwa inyunganizi n'iry'igereranya; kandi ikigereranyo k'ibyo guhura n'umwotsi cyerekanye isano iri hagati y'ibyanduza umwuka n'ubunini bw'umwana uir munda. Iyi trial yanditswe muri ClinicalTrials.gov (NCT02944682)

Ibyavuye mu bushakashatsi: Hagati y'itariki ya 7 Gicuransi 2018 na 29 Gashyantare 2020, twashyize mu matsinda abagore 3200 batwite (1593 mu itsinda rihabwa inyunganizi na 1607 mu itsinda ry'igereranya). Impuzandengo y'ibyumweru byo gutwita 14.5 ± 3.0, impuzandengo y' imyaka y'ababyeyi ni 25 ± 4.5.Twakoze ibizamini bya ekogarafi ni 3147 (98.3%) ku isura ry'ibanzd, 3052 (95.4%) Isura rya mbere mu gihe cyo gutwita na 2962 (92.6%) ku isura rya kabiri mu gihe cyo gutwita, kugera tariki ya 25 Kanama 2020. Kubahiriza guhabwa inyunganizi byari biri hejuru (ijanisha ry'impuzandengo y'iminsi yo gukoresha amashyiga akoresha ibicanwa bikomoka ku bimera ryari 0%, interquartile range 0-1.6%) kandi abagore batwite bari mu itsinda rihabwa inyunganinzi bagize impuzandengo ntoya (± SD) guhura na PM2.5 (35.0 ± 37.2 vs. 103.3 ± 97.9 μg/m^3) ugereranyije n'abitsinda ry'igenzura. Ntabwo twabonye itandukaniro mu mpuzandengo ya Z-scores nyuma yo gushyira mu matsinda ku bipimo by' umuzenguruka w'umutwe (0.30 vs. 0.39; p=0.04), umuzenguruko wo munda (0.38 vs. 0.39; p=0.99), uburebure bw'igufwa rya femur (0.44 vs. 0.45; p=0.73) hamwe n'ikigereranyo cy'ibiro by'umwana munda/avuka (-0.13 vs. -0.12; p=0.70) hagati y'abari bari mu itsinda rihabwa inyunganizi n'igereranya. Guhura kw'ibyanduza umwotsi wo mu rugo n'umuntu ntabwo byagiranye isano n'ubunini bw'umwana uri munda.

Ibisobanuro: Nubwo inyunganizi yo guteka hakoreshejwe LPG byagabanyije guhura no kwandura k'umwuka mu gihe cyo gutwita, ntibyagize ingaruka ku bunini bw'umwana munda.Ibyavuye muri ubu bushakashatsi ntabwo byemeje gukoresha amashyiga ya gaze ikomoka kuri peteroli ari ingamba yo kongera ubunini bw'umwana batwite ahakoreshwa cyane ibicanwa bikomoka ku bimera mu guteka.

Umuterankunga: Ikigo cy'Igihugu gishinzwe Ubuzima muri Leta Zunze Ubumwe za Amerika, Bill & Melinda Gates Foundation.
