# Supplementary material for: Cooking with liquefied petroleum gas or biomass and fetal growth outcomes: a multi-country randomised controlled trial
Source: Lancet Glob Health. 2024 Apr 11;12(5):e815–25. doi: 10.1016/S2214-109X(24)00033-0 (PMC11027158; doi:10.1016/S2214-109X(24)00033-0)
Supplement: Spanish translation of the abstract [file mmc2.pdf]

THE LANCET
Global Health

Supplementary appendix 2

This translation in Spanish was submitted by the authors and we reproduce it as supplied. It has not been peer reviewed. *The Lancet*'s editorial processes have only been applied to the original in English, which should serve as reference for this manuscript.

Los autores nos proporcionaron esta traducción al español y la reproducimos tal como nos fue entregada. No la hemos revisado. Los procesos editoriales de *The Lancet* se han aplicado únicamente al original en inglés, que debe servir de referencia para este manuscrito.

Supplement to: Checkley W, Thompson LM, Hossen S, et al. Cooking with liquefied petroleum gas or biomass and fetal growth outcomes: a multi-country randomised controlled trial. *Lancet Glob Health* 2024; **12:** e815–25.

Resumen
Antecedentes: La contaminación del aire en el hogar puede conducir a la restricción del crecimiento fetal durante el embarazo. Nos propusimos investigar si una intervención con gas licuado de petróleo (GLP) para reducir la exposición personal a la contaminación del aire en el hogar durante el embarazo alteraría el crecimiento fetal.
Métodos: El ensayo de la Red de Intervención contra la Contaminación del Aire en los Hogares (HAPIN, por sus siglas en inglés) fue un ensayo aleatorizado controlado abierto realizado en 10 sitios de recursos limitados en Guatemala, India, Perú y Ruanda (NCT02944682). Mujeres embarazadas de 18 a 34 años (9-19 semanas de gestación) fueron asignadas aleatoriamente en una proporción de 1:1 para recibir una cocina de GLP, suministro continuo de combustible y mensajes de comportamiento o continuar cocinando de manera habitual con biomasa durante 18 meses. Realizamos evaluaciones ecográficas al inicio del estudio, a las 24-28 semanas de gestación (primera visita de embarazo) y a las 32-36 semanas de gestación (segunda visita de embarazo) para medir el tamaño fetal, monitoreamos las exposiciones personales a contaminantes del aire en el hogar durante 24 horas en estas visitas, y pesamos a los niños al nacer. Realizamos análisis por intención de tratar para estimar las diferencias en el tamaño fetal entre los participantes del grupo intervención y los de control; y análisis de exposición-respuesta para identificar asociaciones entre los contaminantes del aire doméstico y el tamaño fetal. Este ensayo está registrado en ClinicalTrials.gov (NCT02944682).
Hallazgos: Entre el 7 de mayo de 2018 y el 29 de febrero de 2020, asignamos aleatoriamente a 3200 mujeres embarazadas (1593 al grupo de intervención y 1607 al grupo de control). La edad gestacional media fue de 14,5 (DE 3,0) semanas y la edad materna media fue de 25,6 (4,5) años. Obtuvimos evaluaciones ecográficas en 3147 (98,3%) mujeres en la línea de base, 3052 (95,4%) mujeres en la primera visita de embarazo y 2962 (92,6%) en la segunda visita de embarazo, hasta el 25 de agosto de 2020. La adherencia a la intervención fue alta (la proporción mediana de días de uso cocinas de biomasa fue del 0,0%, RIC 0,0–1,6) y las mujeres embarazadas del grupo de intervención tuvieron exposiciones medias a $PM_{2,5}$ más bajas (35,0 [DE 37,2] μg / m³ vs 103,3 [97,9] μg/m³) que las mujeres del grupo de control. No encontramos diferencias en los puntajes Z post-aleatorización promediados para la circunferencia cefálica (0,30 vs 0,39; p = 0,04), la circunferencia abdominal (0,38 vs 0,39; p = 0,99), la longitud del fémur (0,44 vs 0,45; p = 0,73) y el peso fetal estimado o el peso al nacer (-0,13 vs -0,12; p = 0,70) entre los grupos de intervención y control. Las exposiciones personales a contaminantes del aire en el hogar no estuvieron asociadas con el tamaño fetal.
Interpretación: Aunque una intervención para cocinar con GLP redujo con éxito la exposición personal a la contaminación del aire durante el embarazo, esta no afectó al tamaño fetal. Nuestros hallazgos no respaldan el uso de cocinas de gas licuado de petróleo sin ventilación como estrategia para aumentar el crecimiento fetal en entornos donde se utilizan principalmente combustibles de biomasa para cocinar.
Financiamiento: Institutos Nacionales de Salud de los Estados Unidos, Fundación Bill y Melinda Gates
