# Supplementary material for: Cooking with liquefied petroleum gas or biomass and fetal growth outcomes: a multi-country randomised controlled trial
Source: Lancet Glob Health. 2024 Apr 11;12(5):e815–25. doi: 10.1016/S2214-109X(24)00033-0 (PMC11027158; doi:10.1016/S2214-109X(24)00033-0)
Supplement: Tamil translation of the abstract [file mmc3.pdf]

THE LANCET
Global Health

Supplementary appendix 3

This translation in Tamil was submitted by the authors and we reproduce it as supplied. It has not been peer reviewed. *The Lancet's* editorial processes have only been applied to the original in English, which should serve as reference for this manuscript.

தமிழில் இந்த மொழிபெயர்ப்பு ஆசிரியர்களால் சமர்ப்பிக்கப்பட்டது, நாங்கள் அதை வழங்கியபடி மீண்டும் உருவாக்குகிறோம்.இது மதிப்பாய்வு செய்யப்படவில்லை. லான்செட்டின் தலையங்க செயல்முறைகள் ஆங்கிலத்தில் அசலுக்கு மட்டுமே பயன்படுத்தப்பட்டுள்ளன, இது இந்த கையெழுத்துப் பிரதிக்கான குறிப்பாக செயல்பட வேண்டும்.

Supplement to: Checkley W, Thompson LM, Hossen S, et al. Cooking with liquefied petroleum gas or biomass and fetal growth outcomes: a multi-country randomised controlled trial. *Lancet Glob Health* 2024; **12:** e815–25.

ஆராய்ச்சி கட்டுரையின் சுருக்கம்

கர்ப்ப காலத்தில் விறகடுப்பை பயன்படுத்துவதால் ஏற்படும் புகையை சுவாசிப்பது கருவின் வளர்ச்சியை பாதிக்கலாம். எல்பிஜி அடுப்புகளை கர்ப்பிணி பெண்களுக்கு கொடுத்து, அவர்கள் சுவாசிக்கும் காற்றின் மாசுபாட்டை குறைப்பதால் கருவின் வளர்ச்சி மேம்படுமா என அறிவதற்கு இவ்வாய்வு மேற்கொள்ளப்பட்டது.

ஆய்வின் நடைமுறை: குவாத்தமாலா, இந்தியா, பெரு மற்றும் ருவாண்டா ஆகிய 4 நாடுகளில் ஆய்வு நடத்தபட்டது. (ஆய்வு எண்: NCT02944682). 9-19 வார கர்ப்பகாலத்தில் உள்ள 18-34 சராசரி வயதுடைய கர்ப்பிணிப் பெண்களை தேர்ந்தெடுத்து தோராயமாக 1:1 விகிதத்தில் இரு குழுவாக பிரித்து, ஒரு குழுவினர்க்கு இலவசமாக எல்பிஜி அடுப்பு, ஆய்வு காலம் (18 மாதங்கள்) முழுவதும் எரிபொருள் விநியோகம் எல்பிஜி அடுப்பை மட்டுமே பயன்படுத்த சொல்லி வலியுறுத்தும் செய்திகளும் வழங்கப்பட்டது. மற்றொரு குழுவினர் தொடர்ந்து விறகடுப்பையே ஆய்வு காலம் முழுவதும் பயன்படுத்தினர். இரு சாராருக்கும் ஆய்வின் தொடக்கத்திலும், 24-28 வாரம் (P1) மற்றும் 32-36 வாரம் (P2) ஆகிய கர்ப்பகால கட்டங்களிலும் அல்ட்ராசவுண்ட் கருவி மூலம் கரு வளர்ச்சி அளவுகள் பெறப்பட்டது. அது போன்றே அவர்கள் சுவாசிக்கும் 24 மணி நேர காற்று மாசுபாட்டின் அளவுகளும் அந்த காலகட்டத்தில் அளக்கப்பட்டது.

ஆய்வின் முடிவுகள்: இவ்வாய்வில் மேற்கூறிய நான்கு நாடுகளின் 10 இடங்களிலிருந்து சராசரியாக 25·6 ± 4·5 வயதிலிருந்த 3200 கர்ப்பிணி பெண்கள், சராசரியாக தங்களின் 14·5 ± 3·0 கர்ப்ப வாரத்தில், ஆய்வில் இணைந்துகொண்டனர். மே 7, 2018 தொடங்கி பிப்ரவரி 29, 2020 வரை ஆய்வு நடைபெற்றது. கருவின் வளர்ச்சி அளவீடுகள் அல்ட்ராசவுண்ட் மூலம் 3147 (98·3%) கர்ப்பிணிகளுக்கு ஆய்வின் தொடக்ககாலத்திலும், 3052 (95·4%) கர்ப்பிணிகளுக்கு P1 காலத்திலும், 2962 (92·6%) கர்ப்பிணிகளுக்கு P2 காலத்திலும் அறியப்பட்டது. எல்பிஜி பயன்படுத்தும் பெண்கள் குழுவில் எல்பிஜி அடுப்பே பெரும்பாலும் பயன்படுத்தினர் (விறகடுப்பு பயன்பாடு - சராசரி %, இடைப்பட்ட வரம்பு நாட்கள் -0%, 0–1·6%) ஆகும். மேலும், இவர்கள் சுவாசிக்கும் சராசரி புகைமாசுபாடு $PM_{2·5}$ (35·0 ± 37·2 $\mu g/m^3$) விறகடுப்பு பயன்படுத்தும் குழுவில் உள்ள கர்ப்பிணிகளின் சராசரி அளவை (103·3 ± 97·9 $\mu g/m^3$) விட குறைவாக இருந்தது. கருவின் வளர்ச்சி அளவீடுகளான தலை சுற்றளவு (0·30 vs. 0·39; p=0·04), வயிற்று சுற்றளவு (0·38 vs 0·39; p=0·99), தொடைஎன்பு நீளம் (0·44 vs 0·45; p =0·73) மற்றும் அல்ட்ராசவுண்ட் மூலம் கணிக்கப்பட்ட பிறப்புகால எடை (-0·13 vs. -0·12; p =0·70) ஆகியவற்றின் சராசரி Z ஸ்கோர்களில் வேறுபாடுகள் இல்லை எனக் கண்டறியபட்டது.

விளக்கம்: கர்ப்ப காலத்தில் மட்டும் எல்பிஜி அடுப்பை பயன்படுத்துவதால், கர்ப்பிணி பெண்கள் சுவாசிக்கும் காற்றின் மாசுபாடுகளை வெற்றிகரமாக குறைத்தாலும், அது கருவின் வளர்ச்சி விகிதத்தில் மேம்பாட்டை ஏற்படுத்தவில்லை.

ஆய்வின் நிதிஉதவி : யுனைடெட் ஸ்டேட்ஸ் நேஷனல் இன்ஸ்டிடியூட் ஆப் ஹெல்த், பில் & ஆம்ப்; மெலிண்டா கேட்ஸ் அறக்கட்டளை.
