# Supplementary material for: Cooking with liquefied petroleum gas or biomass and fetal growth outcomes: a multi-country randomised controlled trial
Source: Lancet Glob Health. 2024 Apr 11;12(5):e815–25. doi: 10.1016/S2214-109X(24)00033-0 (PMC11027158; doi:10.1016/S2214-109X(24)00033-0)
Supplement: Supplementary appendix 4 [file mmc4.pdf]

THE LANCET
Global Health

Supplementary appendix 4

This appendix formed part of the original submission and has been peer reviewed. We post it as supplied by the authors.

Supplement to: Checkley W, Thompson LM, Hossen S, et al. Cooking with liquefied petroleum gas or biomass and fetal growth outcomes: a multi-country randomised controlled trial. *Lancet Glob Health* 2024; **12:** e815–25.

Online Supplement to: "Cooking with liquefied petroleum gas or biomass and fetal growth outcomes: A multi-country randomised controlled trial"

Table of Contents

INVESTIGATORS …………………………………………………………………………………… 3

SUPPLEMENTAL METHODS …………………………………………………………………………………… 4

SUPPLEMENTAL TABLE S1 …………………………………………………………………………………… 6
Means and standard deviations of fetal growth Z-scores by visit and intervention arm.

SUPPLEMENTAL FIGURE S1 …………………………………………………………………………………… 7
Percentage of fetal ultrasound visits and birthweights obtained for each exact week of gestational age and stratified by visit.

SUPPLEMENTAL FIGURE S2 …………………………………………………………………………………… 8
Forest plots for head circumference (HC), abdominal circumference (AC), femur length (FL) and estimated fetal weight/birthweight (EFW/BW) by categories of gestational age and intervention arm (control in blue and intervention in red).

SUPPLEMENTAL FIGURE S3 …………………………………………………………………………………… 9
Violin plots for head circumference (HC) in mm by categories of gestational age and intervention arm (control in blue and intervention in red) and stratified by international research center.

SUPPLEMENTAL FIGURE S4 …………………………………………………………………………………… 10
Violin plots for abdominal circumference (AC) in mm by categories of gestational age and intervention arm (control in blue and intervention in red) and stratified by international research center.

SUPPLEMENTAL FIGURE S5 …………………………………………………………………………………… 11
Violin plots for femur length (FL) in mm by categories of gestational age and intervention arm (control in blue and intervention in red) and stratified by international research center.

SUPPLEMENTAL FIGURE S6 …………………………………………………………………………………… 12
Violin plots for estimated fetal weight/birthweight (EFW/BW) in grams by categories of gestational age and intervention arm (control in blue and intervention in red) and stratified by international research center.

SUPPLEMENTAL FIGURE S7 …………………………………………………………………………………… 13
Forest plots for head circumference (HC) in Z-scores by categories of gestational age and intervention arm (control in blue and intervention in red).

SUPPLEMENTAL FIGURE S8 …………………………………………………………………………………… 14

Forest plots for abdominal circumference (AC) in Z-scores by categories of gestational age and intervention arm (control in blue and intervention in red).

SUPPLEMENTAL FIGURE S9 ………………………………………………………………………………… 15
Forest plots for femur length (FL) in Z-scores by categories of gestational age and intervention arm (control in blue and intervention in red).

SUPPLEMENTAL FIGURE S10 ………………………………………………………………………………… 16
Forest plots for estimated fetal weight (EFW) in Z-scores by categories of gestational age and intervention arm (control in blue and intervention in red

SUPPLEMENTAL FIGURE S11 ………………………………………………………………………………… 17
Results of intention-to-treat analyses for the differences in Z-scores of head circumference (HC), abdominal circumference (AC), femur length (FL) and estimated fetal weight/birthweight (EFW/BW) by visit (first pregnancy visit or P1, second pregnancy visit or P2 and birth) and timing of randomisation (for this analysis early corresponded to a GA <15.3 and it is plotted in red and late to ≥ 15.3 weeks and it is plotted in blue) as a function of continuous gestational age displayed as a forest plot.

SUPPLEMENTAL FIGURE S12 ………………………………………………………………………………… 18
Mean and standard deviation of head circumference (HC), abdominal circumference (AC), femur length (FL) and estimated fetal weight/birthweight (EFW/BW) Z-scores by quartiles of 24-hour personal exposures to fine particulate matter ($PM_{2.5}$), black carbon (BC) and carbon monoxide (CO) and visit (baseline visit between 9-19 weeks, first pregnancy visit or P1, second pregnancy visit or P2 and birth).

CONSORT Checklist ………………………………………………………………………………… 19

R MARKDOWN ………………………………………………………………………………… 21

REFERENCES ………………………………………………………………………………… 211

INVESTIGATORS

Authors: William Checkley, Lisa M Thompson, Shakir Hossen, Laura Nicolaou, Kendra N Williams, Stella M Hartinger, Marilú Chiang, Kalpana Balakrishnan, Sarada S Garg, Gurusamy Thangavel, Vigneswari Aravindalochanan, Ghislaine Rosa, Alexie Mukeshimana, Florien Ndagijimana, John P. McCracken, Anaité Diaz-Artiga, Sheela S Sinharoy, Lance Waller, Jiantong Wang, Shirin Jabbarzadeh, Yunyun Chen, Kyle Steenland, Miles A Kirby, Usha Ramakrishnan, Michael Johnson, Ajay Pillarisetti, Eric D McCollum, Rachel Craik, Eric Ohuma, Victor G. Dávila-Román, Suzanne M Simkovich, Jennifer L Peel, Thomas F Clasen, Aris T Papageorghiou; Household Air Pollution Intervention Network (HAPIN) Investigators.

HAPIN Investigators: Gloriose Bankundiye, Dana Boyd Barr, Vanessa Burrowes, Alejandra Bussalleu, Devan Campbell, Eduardo Canuz, Adly Castañaza, Maggie L. Clark, Mary Crocker, Oscar De León, Ephrem Dusabimana, Lisa Elon, Juan G. Espinoza, Irma Sayury Pineda Fuentes, Ahana Ghosh, Dina Goodman, Savannah Gupton, Sarah Hamid, Steven A. Harvey, Mayari Hengstermann, Ian Hennessee, Phabiola Herrera, Marjorie Howard, Penelope P. Howards, Lindsay Jaacks, Katherine Kearns, Jacob Kremer, Margaret A. Laws, Pattie Lenzen, Jiawen Liao, Amy E. Lovvorn, Jane Mbabazi, Julia N. McPeek, Rachel Meyers, J. Jaime Miranda, Erick Mollinedo, Libny Monroy, Krishnendu Mukhopadhyay, Bernard Mutariyani, Luke Naeher, Abidan Nambajimana, Durairaj Natesan, Azhar Nizam, Jean de Dieu Ntivuguruzwa, Ricardo Piedrahita, Naveen Puttaswamy, Elisa Puzzolo, Ashlinn Quinn, Karthikeyan Dharmapuri Rajamani, Sarah Rajkumar, Rengaraj Ramasami, Alexander Ramirez, P. Barry Ryan, Sudhakar Saidam, Zoe Sakas, Sankar Sambandam, Jeremy A. Sarnat, Kirk R. Smith, Damien Swearing, Ashley Toenjes, Lindsay Underhill, Jean Damascene Uwizeyimana, Viviane Valdes, Amit Verma, Jiantong Wang, Megan Warnock, Wenlu Ye, Bonnie N. Young, Ashley Younger. We would also like to thank all sonographers with whom this work would have not been possible: Libny Y. Monroy-Alarcón, Adly Castañaza Gonzalez de Durante, Claudia López-Ortega, Maria F Gonzalez, Lakshminarayanan Sowrirajan, Shanthi P. Paramanandam, K Shanmugavadivu, V Sudharsanan, Suresh Seshadri, Adhemir E. Yupanqui-Fredes, Mario Hancco-Gomez, Ronald Apaza, Juan F. Persivale-Calle, Elizabeth Quispe, Carlos Leon-Ponce, Victor Villar-Gonzales, Rebeca Andrade-Salas, Jhon E Herrera, Luzdelia Ramos-Mamani, Yessica Lopez, Giovanna Quiza, Yadel Hinojosa, Madeluz Gomez-Quispe, Gery Frisancho-Parada, Danielle I Mendoza-Apaza, Luz R Quispe-Flores, Niyitegeka F Xavier, Grace Utfimana, Elie Tuzayisenga, Valens Nkurunziza.

US National Institutes of Heart, Lung, Blood Institute Data Safety Monitoring Board: Catherine J Karr (Chair), Nancy R Cook, Stephen Hecht, Joseph Millum, Nalini Sathiakumar (deceased), Paul K Whelton, Thomas Croxton (Executive Secretary).

Program Coordination: Gail Rodgers, Bill & Melinda Gates Foundation; Claudia L Thompson, National Institute of Environmental Health Sciences; Mark J Parascandola, National Cancer Institute; Marion Koso-Thomas, Eunice Kennedy Shriver National Institute of Child Health and Human Development; Joshua P Rosenthal, Fogarty International Center; Concepcion R Nierras, NIH Office of Strategic Coordination, The Common Fund; Katherine Kavounis, Dong-Yun Kim, Antonello Punturieri, and Barry S Schmetter (deceased), National Heart, Lung, and Blood Institute.

Advisory Committee: The investigators would like to thank the Drs. Patrick Breysse, Donna Spiegelman, and Joel Kaufman for their valuable insight and guidance throughout the implementation of the trial. We also wish to acknowledge all the research staff and study participants for their dedication to and participation in this important trial.

SUPPLEMENTAL METHODS

Assessment of personal exposures to air pollution

We assessed 24-hour personal exposures to fine particulate matter (<2.5 µm in aerodynamic diameter, $PM_{2.5}$) using the Enhanced Children's MicroPEM (ECM, RTI International, Durham, NC, USA) and to carbon monoxide (CO) using the Lascar EL-USB-300 (Lascar Electronics, Erie, PA, USA). Exposures were estimated at three timepoints during pregnancy at baseline (9-19 weeks of gestation) before randomization, at 24-28 weeks and 32-36 weeks of gestation (1, 2).

The ECM is a combined gravimetric and nephelometric sampler that weighs approximately 150 g and can operate continuously at 0.3 L/min. It collects gravimetric samples on 15 mm polytetrafluoroethylene filters (Measurement Technology Laboratories, Minneapolis, MN, USA). Changes in filter mass pre- and post-sampling were assessed using 1 µg resolution microbalances (Sartorius Cubis, MSA6.6s-000-DF) at the University of Georgia (filters for Guatemala, Rwanda, and Peru) and at Sri Ramachandra Institute for Higher Education and Research (filters for India). We evaluated pre- and post-sample flow rates with a flowmeter and removed samples outside of expected ranges. We also invalidated samples with damaged filters, and removed data that did not meet criteria for sample duration (24 ± 4h), flow rate (300 ± 100 mL/min, measured by the internal flow sensor), and inlet pressure (95th percentile less than <5 inches H_2O). We used instrument-specific nephelometric $PM_{2.5}$ concentrations normalized to field-based filter samples in instances where gravimetric samples were invalid (2). We collected field blanks at a rate of 4 per 100 sample filters and calculated the limit of detection (LOD) separately for each Intervention Research Center (IRC) as three times the standard deviation of the blank mass depositions. Sample depositions below the LOD were replaced with $LOD/\sqrt{2}$. We also deployed duplicate ECMs on a subset of samples to assess between-monitor performance.

Black carbon (BC) concentrations were estimated for $PM_{2.5}$ filter samples using the SootScan Model OT-21 Optical Transmissometers (Magee Scientific, Berkeley, CA), either at the University of Georgia (Athens, GA, USA) for samples collected in Guatemala, Peru, and Rwanda or at Sri Ramachandra Institute for Higher Education and Research (Chennai, India) for samples collected in India.

CO data quality assurance procedures included calibrations with zero air and CO span gas (ranging between 40 and 80 ppm), automated, server-based quality assurance checks and a manual, visual rating system. We calibrated CO loggers every 1–3 months using the temporally closest calibration coefficient. We removed data that did not meet criteria sampling duration (24h ± 4h), and visually rated the CO times series data to remove samples that displayed response artifacts. We deployed duplicate CO monitors on a subset of samples to assess between-monitor performance.

Stove use monitoring

We used Geocene (Geocene Inc., Vallejo, CA, USA) thermocouple stove use monitors (SUMs) to assess traditional stove use in intervention households. This decision was driven by the evidence that traditional stove use must be nearly eliminated (<1 hour per week) to achieve reductions in HAP that may translate into improved health outcomes. We additionally monitored the LPG stove in a subset of intervention homes and the traditional stove in a subset of control homes. SUMs measured cookstove temperatures using a K-type thermocouple. We set SUMs to record temperature every five minutes throughout the duration of the trial and were downloaded every two weeks. Temperature readings were used to flag cooking events based on temperature increases above identified thresholds that lasted at least five minutes. Details on SUMs data collection and quality control are described in detail elsewhere (1, 4).

Construction of a socioeconomic status index

We used a principal component analysis (PCA) to construct an socioeconomic status index based on ownership of 24 selected household assets, water and sanitation quality, access to electricity, number of people in the household, food insecurity, participant's education level, and floor, wall, and roofing material at baseline. We conducted multiple imputation in chained equations for missing data in the PCA (3). No variable was missing for more than 4% of observations.

Table S1. Means and standard deviations of fetal growth Z-scores by visit and intervention arm.

	Z-scores, mean (SD)			
	P1		P2	
Fetal assessment	Intervention	Control	Intervention	Control
Head circumference	0·35 (1·12)	0·49 (1·09)	0·30 (1·19)	0·31 (1·20)
Abdominal circumference	0·46 (1·18)	0·49 (1·17)	0·36 (1·11)	0·34 (1·13)
Femur length	0·40 (1·16)	0·46 (1·11)	0·46 (1·06)	0·44 (1·11)
Estimated fetal weight	0·50 (1·24)	0·58 (1·16)	0·35 (1·07)	0·33 (1·11)

Figure S1. Percentage of fetal ultrasound visits and birthweights obtained for each exact week of gestational age and stratified by visit.

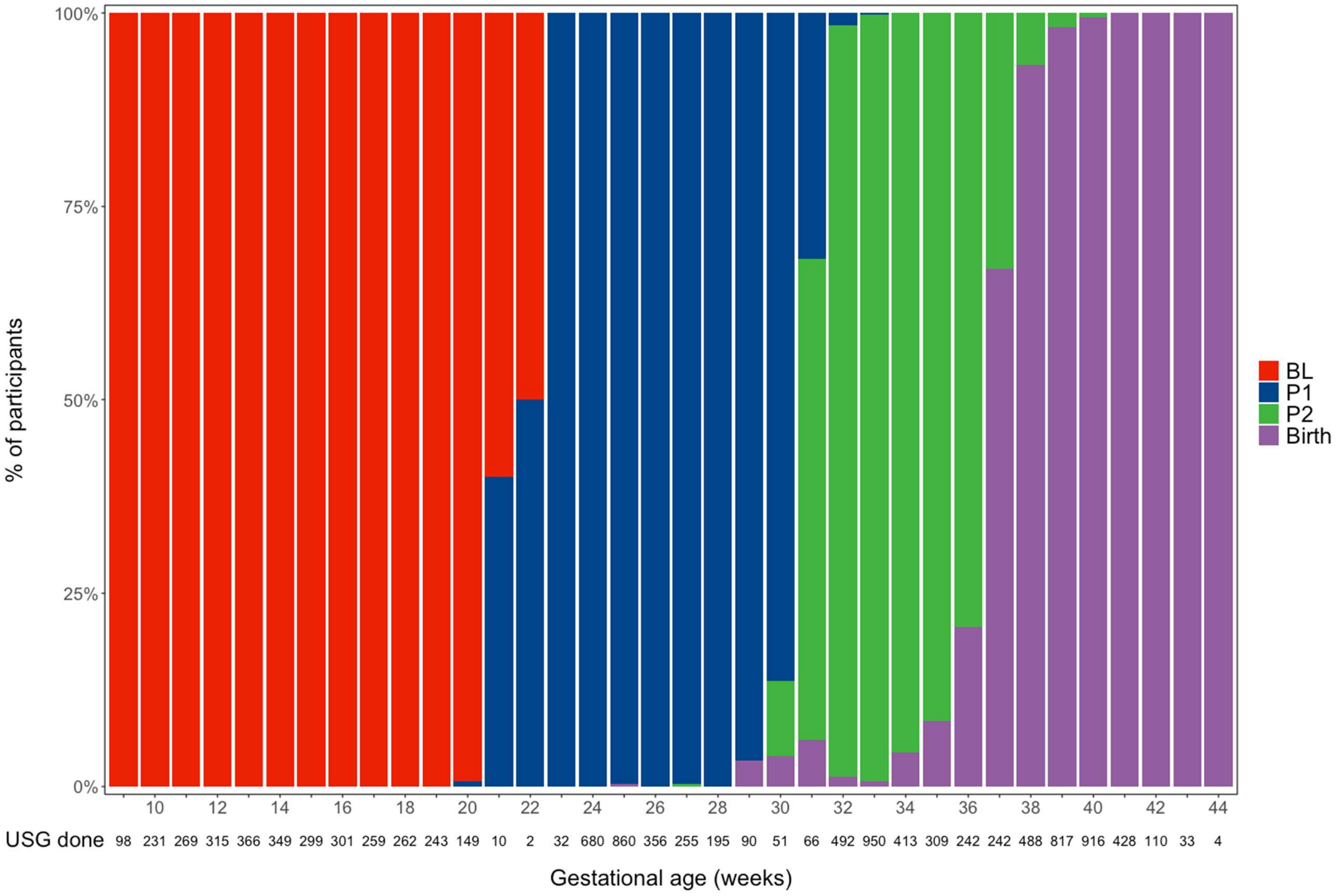

Figure S2. Forest plots for head circumference (HC), abdominal circumference (AC), femur length (FL) and estimated fetal weight/birthweight (EFW/BW) by categories of gestational age and intervention arm (control in blue and intervention in red). Each panel plots the means as diamonds and corresponding 95% confidence intervals as horizontal lines. We also display the numerical means and standard deviation (SD) for each row on the right side of the panels.

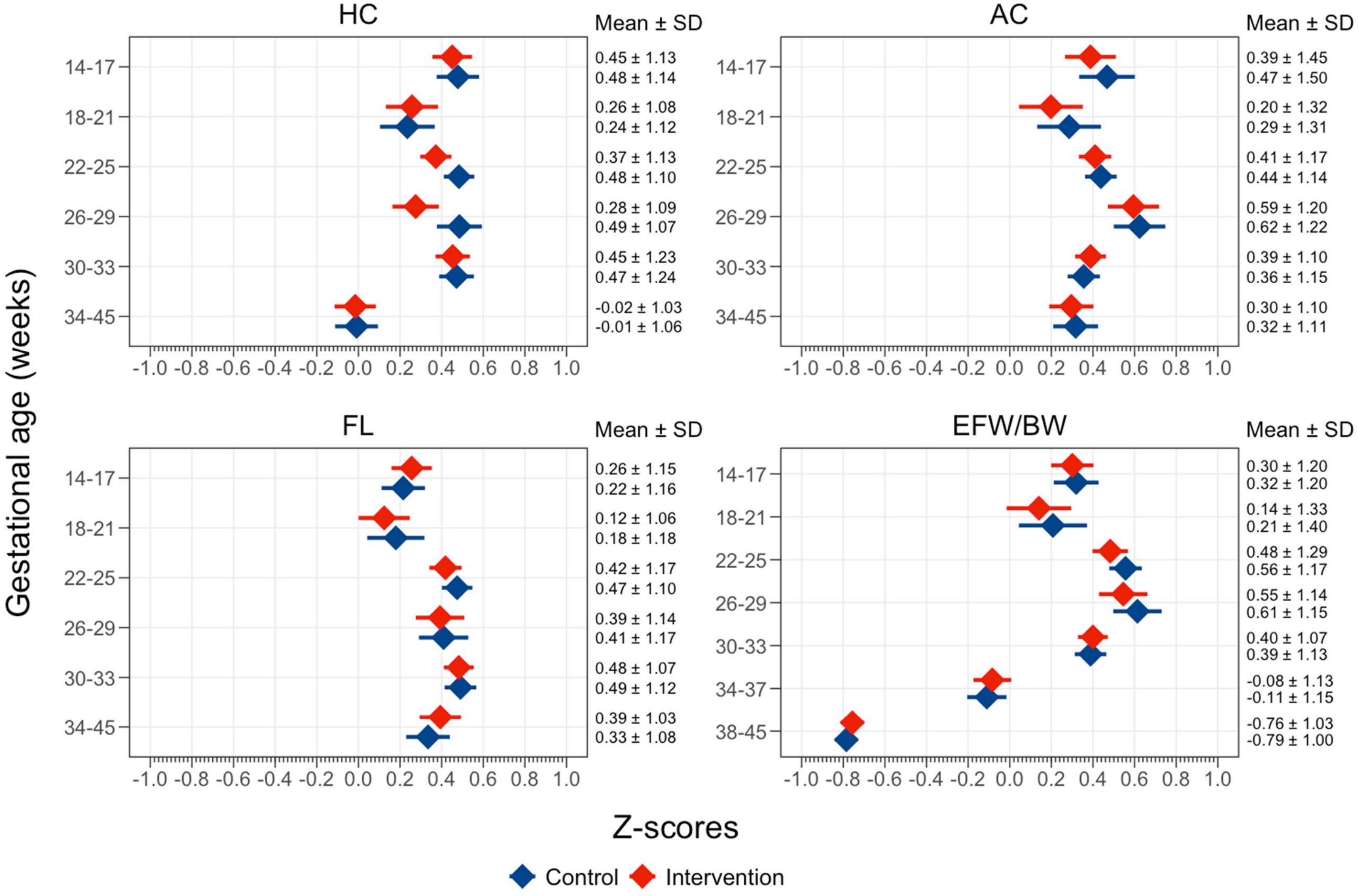

Figure S3. Violin plots for head circumference (HC) in mm by categories of gestational age and intervention arm (control in blue and intervention in red) and stratified by international research center. Violin plots are hybrids of boxplots and density plots, and we use them here to visualize the distribution of fetal growth outcomes. International research centres include Jalapa, Guatemala; Tamil Nadu, India; Puno, Peru; and Kayonza, Rwanda. The thick white band represents the 50th percentile, and the thinner white bands represent the 25th and 75th percentiles.

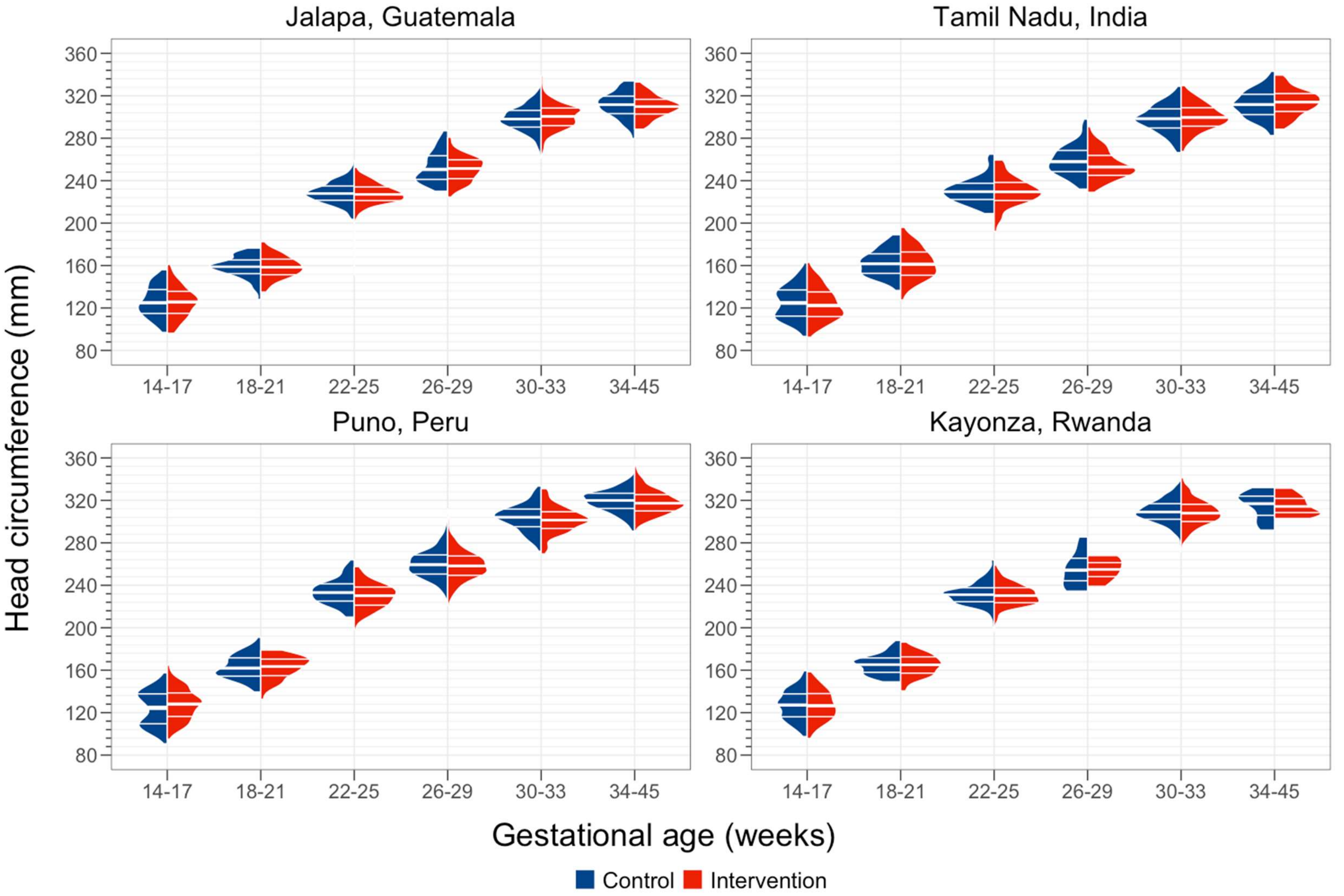

Figure S4. Violin plots for abdominal circumference (AC) in mm by categories of gestational age and intervention arm (control in blue and intervention in red) and stratified by international research center. Violin plots are hybrids of boxplots and density plots, and we use them here to visualize the distribution of fetal growth outcomes. International research centres include Jalapa, Guatemala; Tamil Nadu, India; Puno, Peru; and Kayonza, Rwanda. The thick white band represents the 50th percentile, and the thinner white bands represent the 25th and 75th percentiles.

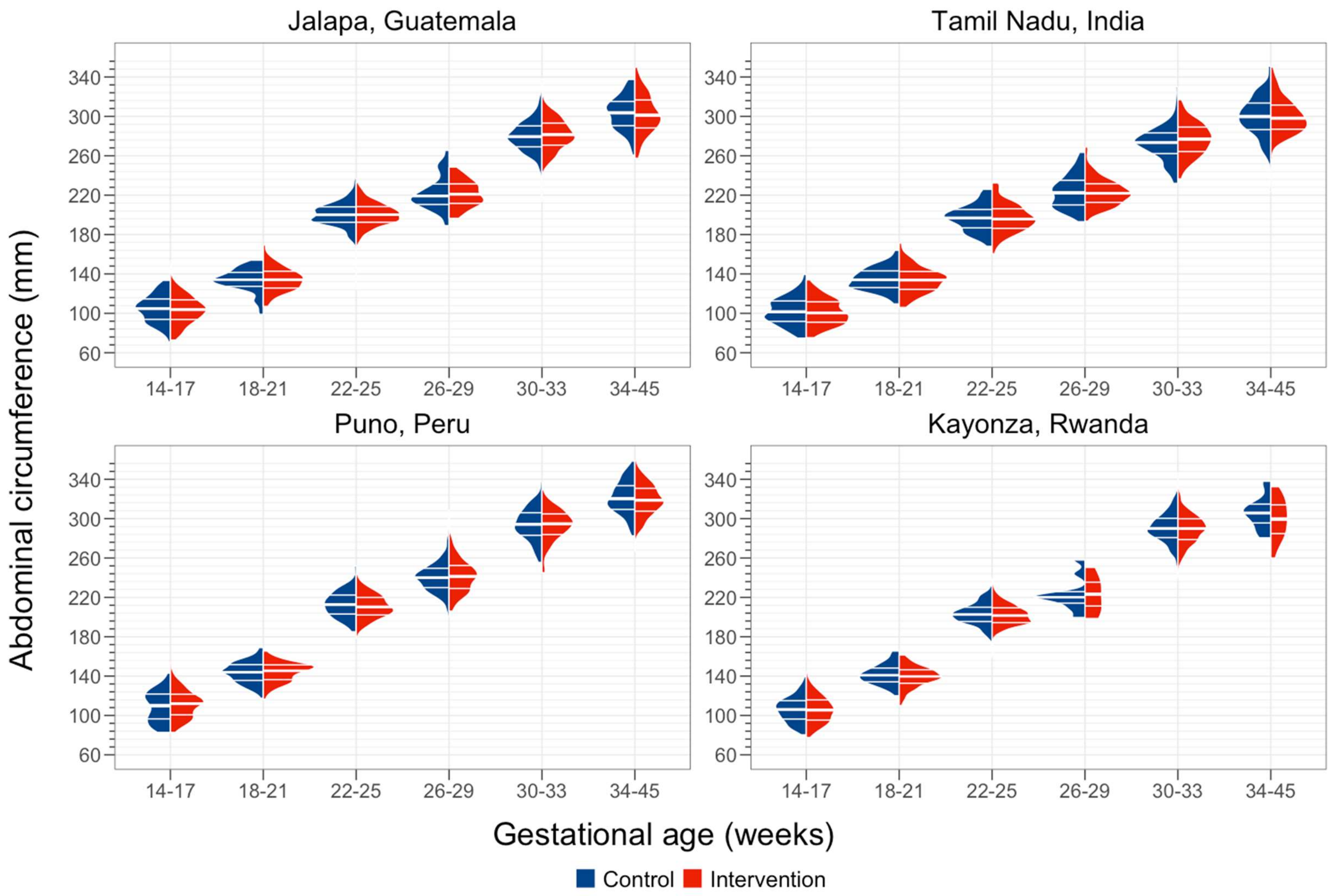

Figure S5. Violin plots for femur length (FL) in mm by categories of gestational age and intervention arm (control in blue and intervention in red) and stratified by international research center. Violin plots are hybrids of boxplots and density plots, and we use them here to visualize the distribution of fetal growth outcomes. International research centres include Jalapa, Guatemala; Tamil Nadu, India; Puno, Peru; and Kayonza, Rwanda. The thick white band represents the 50th percentile, and the thinner white bands represent the 25th and 75th percentiles.

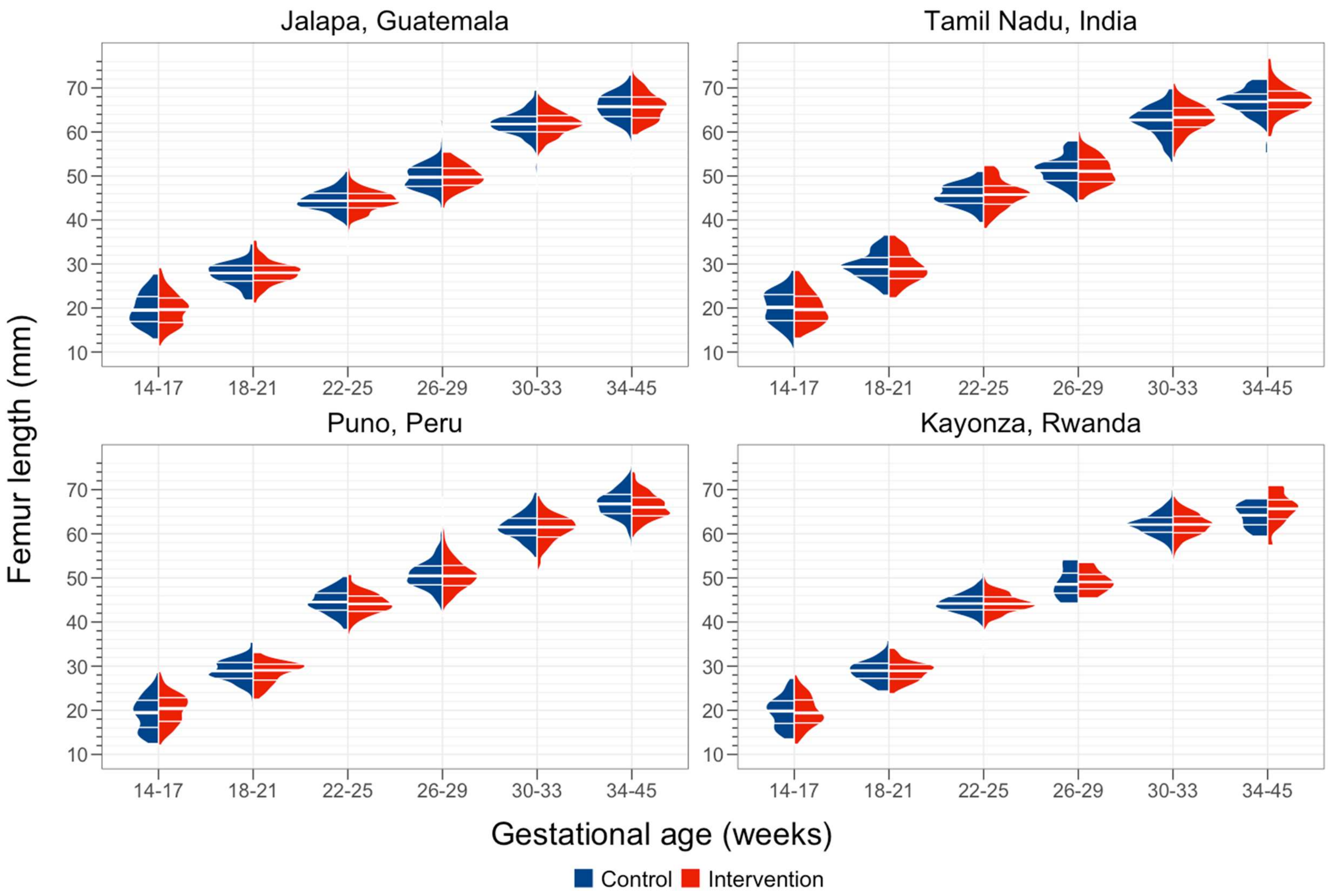

Figure S6. Violin plots for estimated fetal weight/birthweight (EFW/BW) in grams by categories of gestational age and intervention arm (control in blue and intervention in red) and stratified by international research center. Violin plots are hybrids of boxplots and density plots, and we use them here to visualize the distribution of fetal growth outcomes. International research centres include Jalapa, Guatemala; Tamil Nadu, India; Puno, Peru; and Kayonza, Rwanda. The thick white band represents the 50th percentile, and the thinner white bands represent the 25th and 75th percentiles.

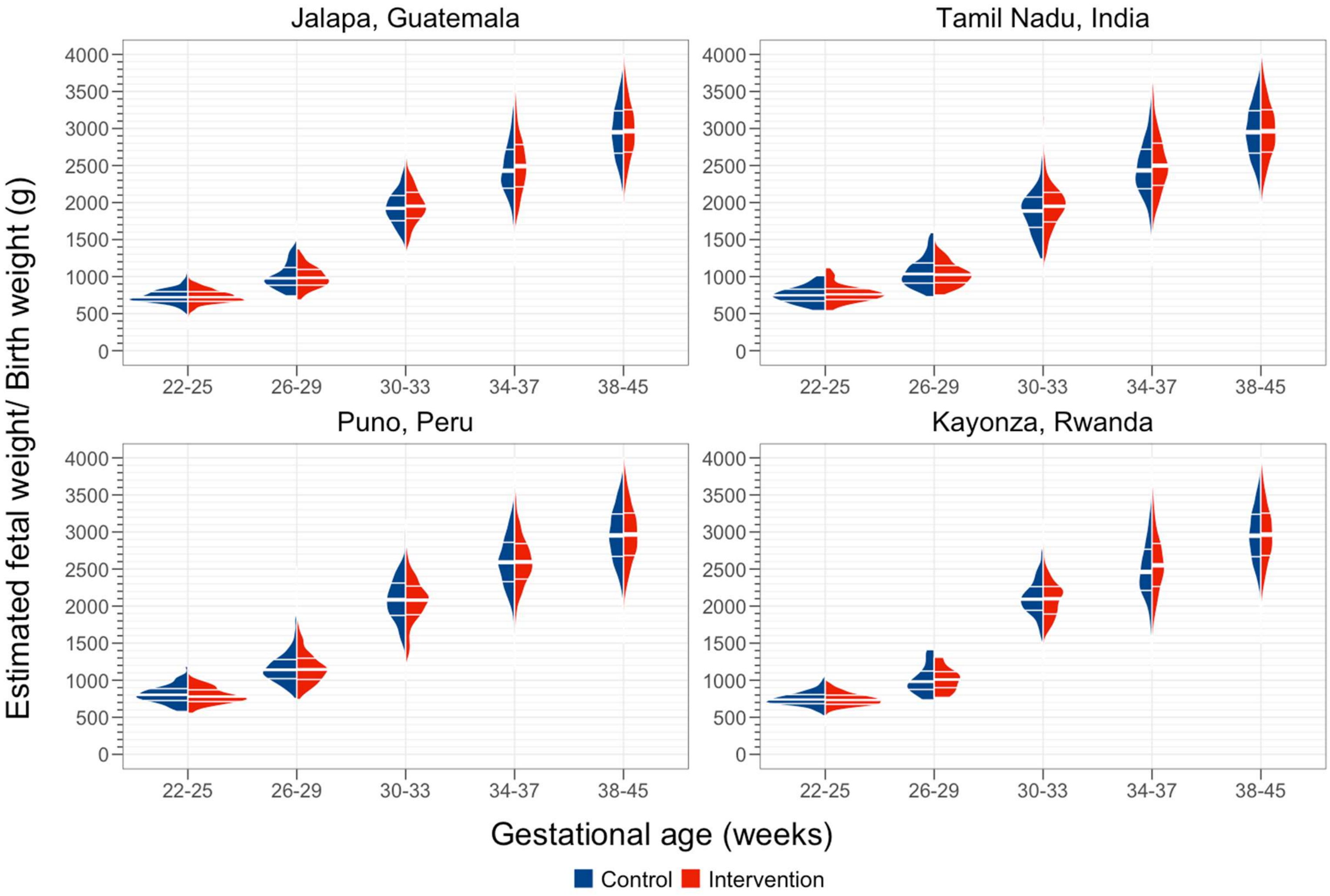

Figure S7. Forest plots for head circumference (HC) in Z-scores by categories of gestational age and intervention arm (control in blue and intervention in red). Each panel plots the means as diamonds and corresponding 95% confidence intervals as horizontal lines. International research centres include Jalapa, Guatemala; Tamil Nadu, India; Puno, Peru; and Kayonza, Rwanda. We also display the numerical means and standard deviation (SD) for each row on the right side of the panels.

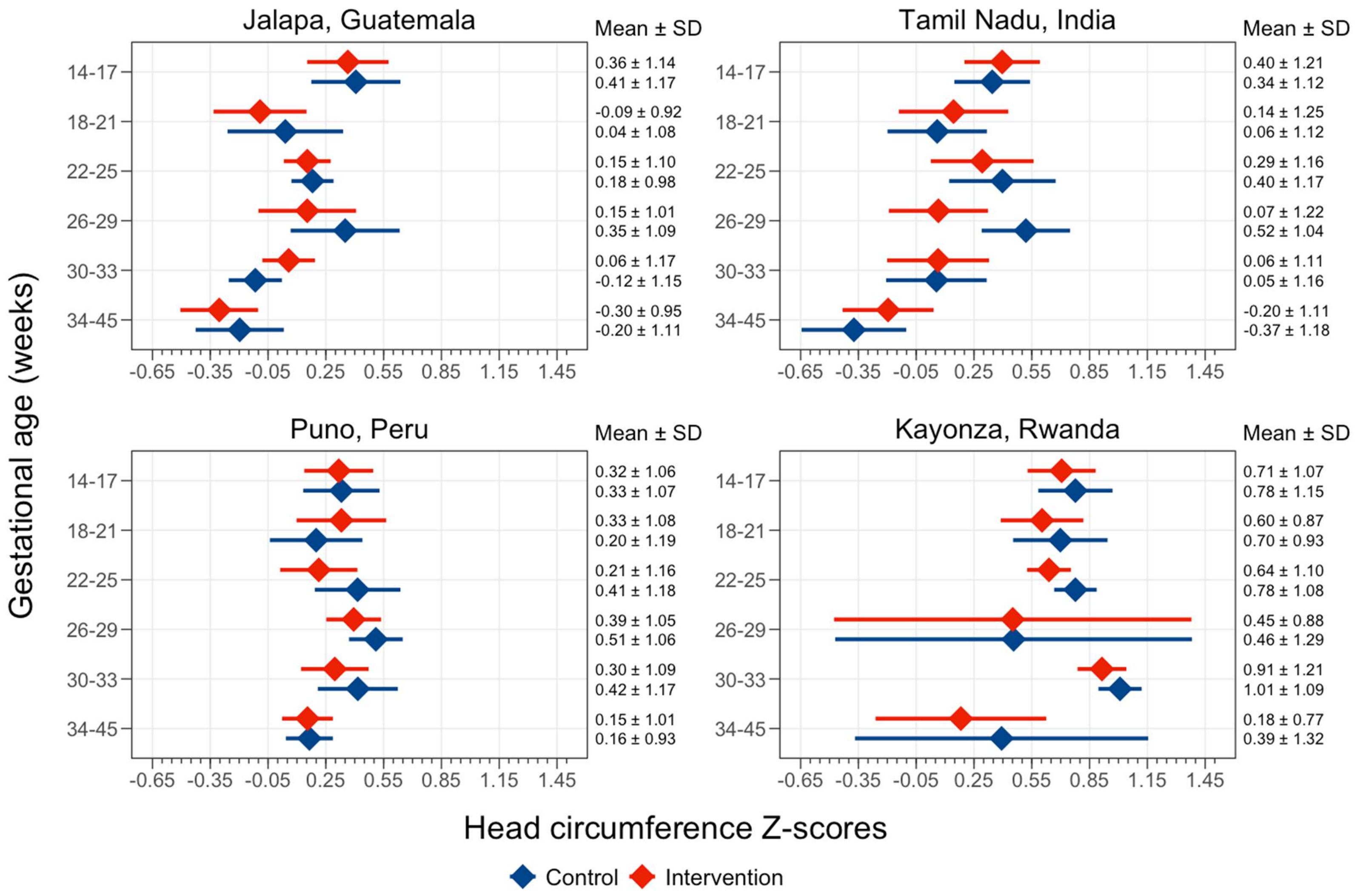

Figure S8. Forest plots for abdominal circumference (AC) in Z-scores by categories of gestational age and intervention arm (control in blue and intervention in red). Each panel plots the means as diamonds and corresponding 95% confidence intervals as horizontal lines. International research centres include Jalapa, Guatemala; Tamil Nadu, India; Puno, Peru; and Kayonza, Rwanda. We also display the numerical means and standard deviation (SD) for each row on the right side of the panels.

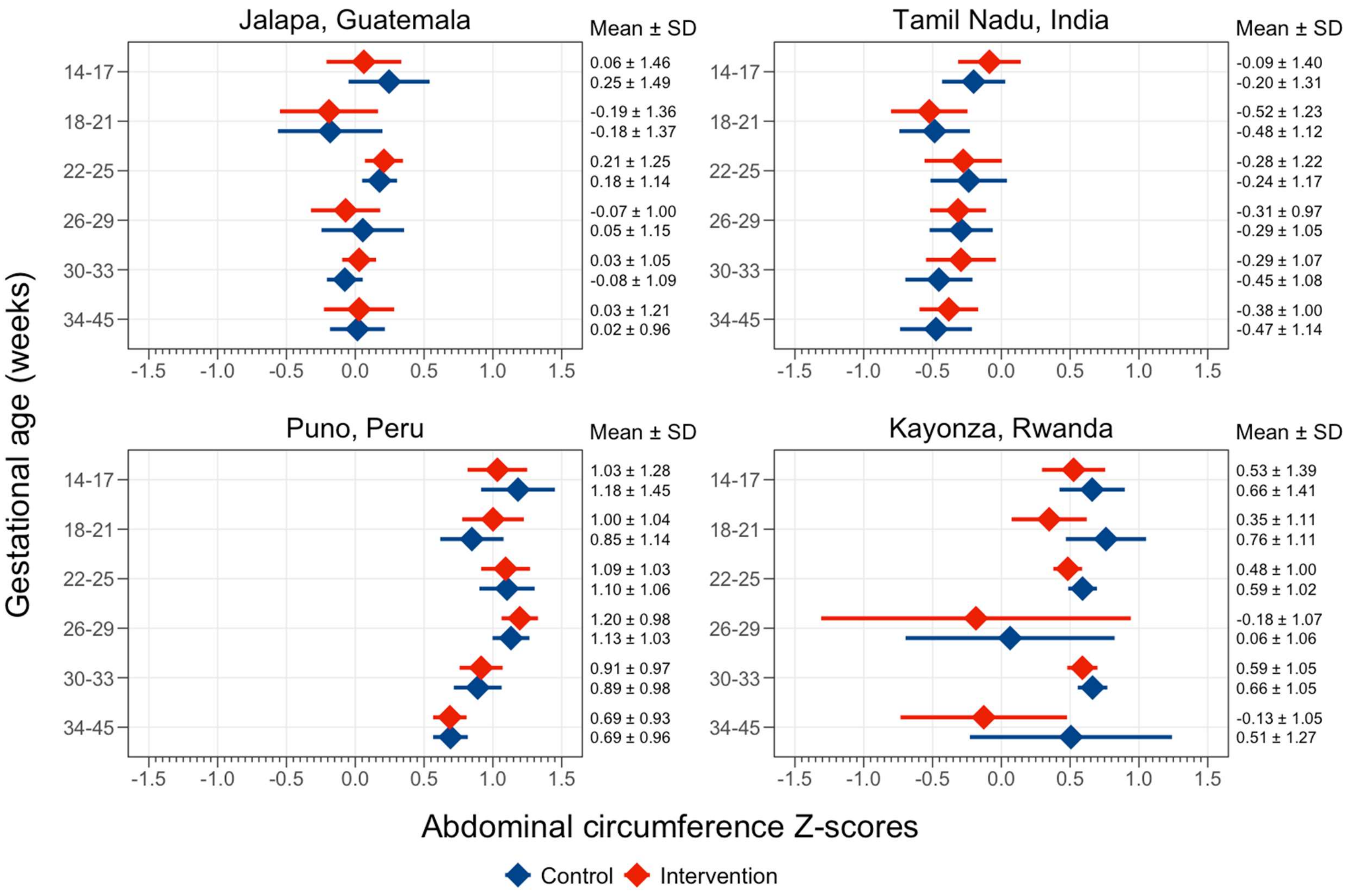

Figure S9. Forest plots for femur length (FL) in Z-scores by categories of gestational age and intervention arm (control in blue and intervention in red). Each panel plots the means as diamonds and corresponding 95% confidence intervals as horizontal lines. International research centres include Jalapa, Guatemala; Tamil Nadu, India; Puno, Peru; and Kayonza, Rwanda. We also display the numerical means and standard deviation (SD) for each row on the right side of the panels.

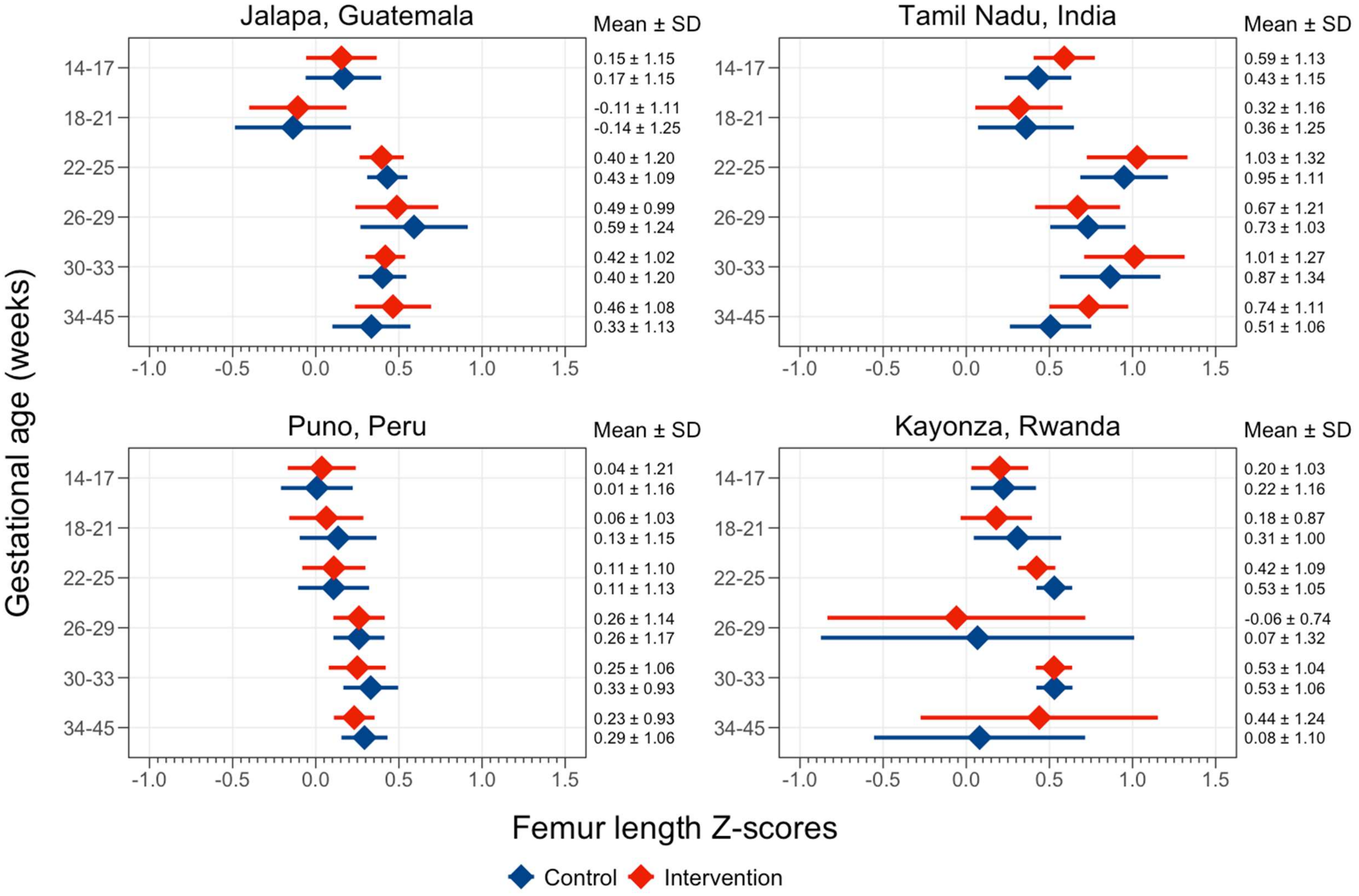

Figure S10. Forest plots for estimated fetal weight (EFW) in Z-scores by categories of gestational age and intervention arm (control in blue and intervention in red). Each panel plots the means as diamonds and corresponding 95% confidence intervals as horizontal lines. International research centres include Jalapa, Guatemala; Tamil Nadu, India; Puno, Peru; and Kayonza, Rwanda. We also display the numerical means and standard deviation (SD) for each row on the right side of the panels.

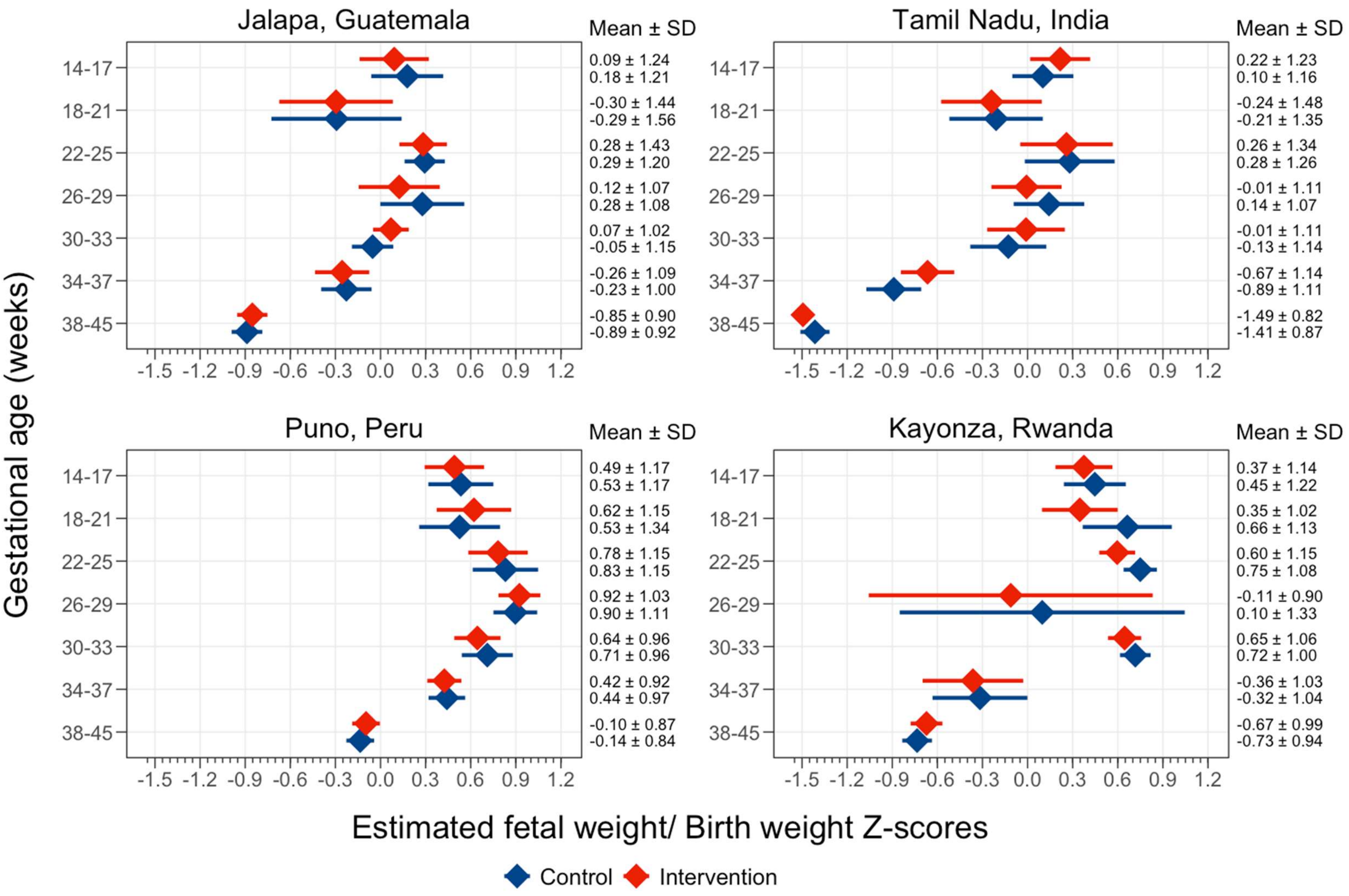

Figure S11. Results of intention-to-treat analyses for the differences in Z-scores of head circumference (HC), abdominal circumference (AC), femur length (FL) and estimated fetal weight/birthweight (EFW/BW) by visit (first pregnancy visit or P1, second pregnancy visit or P2 and birth) and timing of randomisation (for this analysis early corresponded to a GA <15.3 and it is plotted in red and late to ≥ 15.3 weeks and it is plotted in blue) as a function of continuous gestational age displayed as a forest plot. Stacked panels represent one of the four fetal growth outcomes. Differences were estimated from generalized additive mixed models of the fetal growth Z-scores as a function of tensor smooths of gestational age stratified by trial groups (control, intervention with early timing of randomisation, and intervention with late timing of randomisation) adjusted for randomisation strata. We estimated differences at 25, 33 and 40 weeks (for EFW Z-score only). Values below 0 indicate benefit to the control arm, whereas values above 0 indicate benefit to the intervention arm. The estimated averaged post-randomized means by trial arm and the mean differences between arms (intervention – control) are displayed with a diamond and corresponding 98·75% confidence intervals as horizontal lines. We also display the numerical mean differences and 98·75% confidence intervals for each row on the right side of the panels.

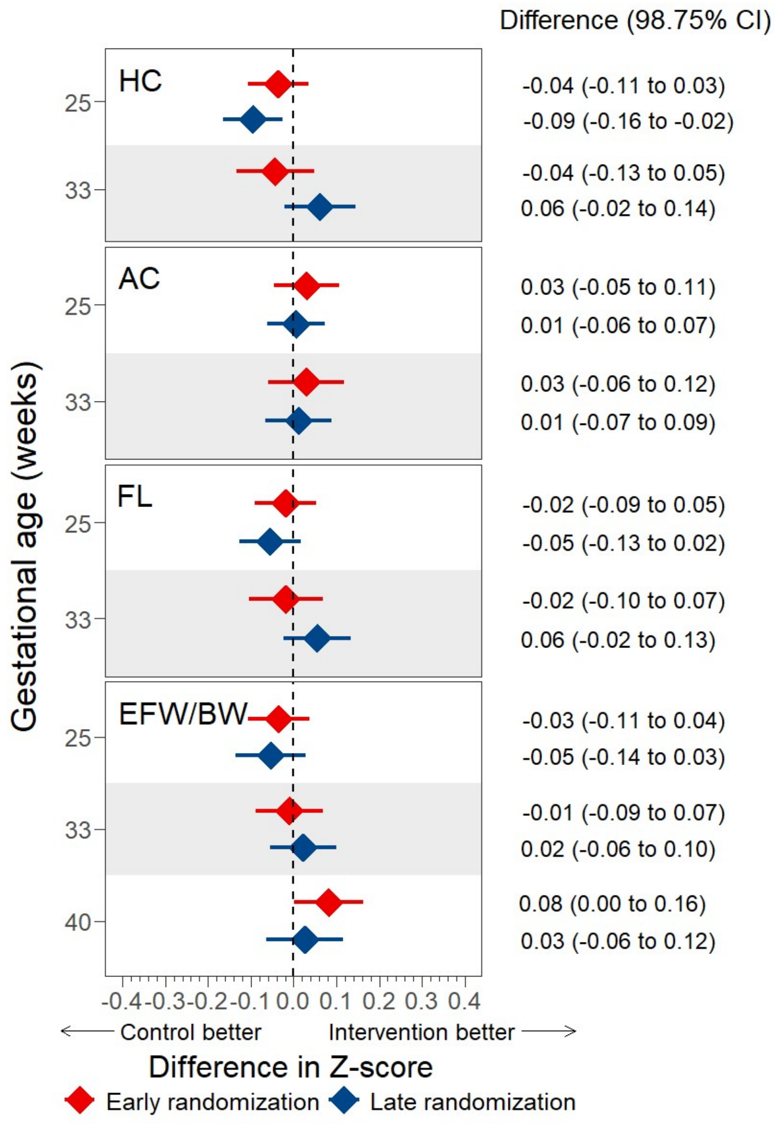

Figure S12. Mean and standard deviation of head circumference (HC), abdominal circumference (AC), femur length (FL) and estimated fetal weight/birthweight (EFW/BW) Z-scores by quartiles of 24-hour personal exposures to fine particulate matter ($PM_{2.5}$), black carbon (BC) and carbon monoxide (CO) and visit (baseline visit between 9-19 weeks, first pregnancy visit or P1, second pregnancy visit or P2 and birth). Means are displayed as diamonds and corresponding 95% confidence intervals as vertical lines. Means and 95% confidence intervals are plotted in a grey scale where the darkening denotes a higher quartile.

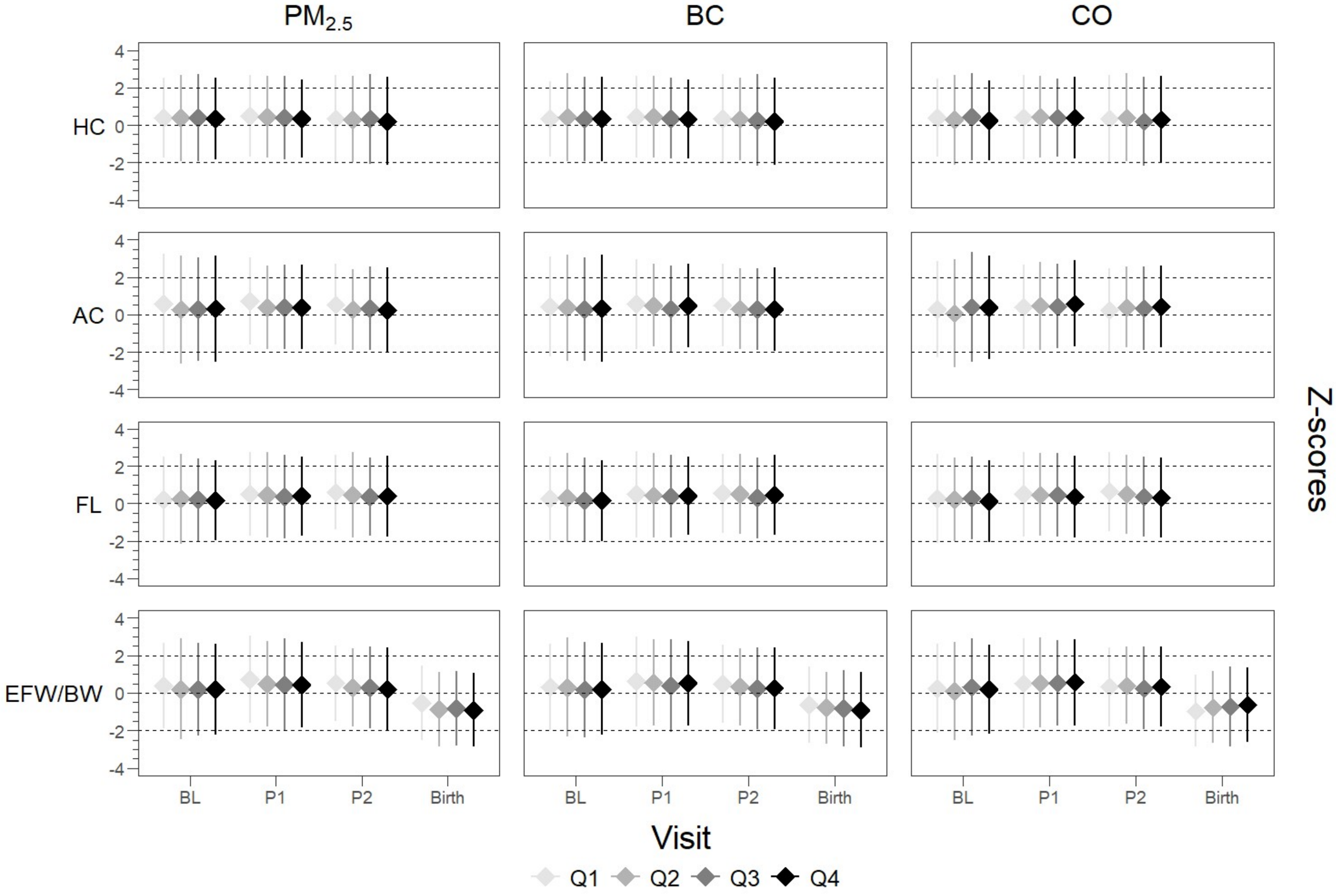

CONSORT 2010 checklist of information to include when reporting a randomised trial

Section/Topic	Item No	Checklist item	Reported on page No
**Title and abstract**			
	1a	Identification as a randomised trial in the title	1
	1b	Structured summary of trial design, methods, results, and conclusions (for specific guidance see CONSORT for abstracts)	4
**Introduction**			
Background and objectives	2a	Scientific background and explanation of rationale	5
	2b	Specific objectives or hypotheses	5
**Methods**			
Trial design	3a	Description of trial design (such as parallel, factorial) including allocation ratio	6
	3b	Important changes to methods after trial commencement (such as eligibility criteria), with reasons	5
Participants	4a	Eligibility criteria for participants	6
	4b	Settings and locations where the data were collected	5
Interventions	5	The interventions for each group with sufficient details to allow replication, including how and when they were actually administered	5-6
Outcomes	6a	Completely defined pre-specified primary and secondary outcome measures, including how and when they were assessed	8
	6b	Any changes to trial outcomes after the trial commenced, with reasons	N/A
Sample size	7a	How sample size was determined	8
	7b	When applicable, explanation of any interim analyses and stopping guidelines	N/A
Randomisation:			
Sequence generation	8a	Method used to generate the random allocation sequence	8
	8b	Type of randomisation; details of any restriction (such as blocking and block size)	8
Allocation concealment mechanism	9	Mechanism used to implement the random allocation sequence (such as sequentially numbered containers), describing any steps taken to conceal the sequence until interventions were assigned	8
Implementation	10	Who generated the random allocation sequence, who enrolled participants, and who assigned participants to interventions	8
Blinding	11a	If done, who was blinded after assignment to interventions (for example, participants, care providers, those assessing outcomes) and how	8
	11b	If relevant, description of the similarity of interventions	5
Statistical methods	12a	Statistical methods used to compare groups for primary and secondary outcomes	8

	12b	Methods for additional analyses, such as subgroup analyses and adjusted analyses	8
**Results**			
Participant flow (a diagram is strongly recommended)	13a	For each group, the numbers of participants who were randomly assigned, received intended treatment, and were analysed for the primary outcome	6
	13b	For each group, losses and exclusions after randomisation, together with reasons	Figure S1
Recruitment	14a	Dates defining the periods of recruitment and follow-up	Abstract, 10
	14b	Why the trial ended or was stopped	10
Baseline data	15	A table showing baseline demographic and clinical characteristics for each group	Table 1
Numbers analysed	16	For each group, number of participants (denominator) included in each analysis and whether the analysis was by original assigned groups	Figure S1, 10
Outcomes and estimation	17a	For each primary and secondary outcome, results for each group, and the estimated effect size and its precision (such as 95% confidence interval)	Figure 2
	17b	For binary outcomes, presentation of both absolute and relative effect sizes is recommended	N/A
Ancillary analyses	18	Results of any other analyses performed, including subgroup analyses and adjusted analyses, distinguishing pre-specified from exploratory	10
Harms	19	All important harms or unintended effects in each group (for specific guidance see CONSORT for harms)	10
**Discussion**			
Limitations	20	Trial limitations, addressing sources of potential bias, imprecision, and, if relevant, multiplicity of analyses	11-12
Generalisability	21	Generalisability (external validity, applicability) of the trial findings	11
Interpretation	22	Interpretation consistent with results, balancing benefits and harms, and considering other relevant evidence	11-12
**Other information**			
Registration	23	Registration number and name of trial registry	6
Protocol	24	Where the full trial protocol can be accessed, if available	Abstract
Funding	25	Sources of funding and other support (such as supply of drugs), role of funders	

Cooking with liquefied petroleum gas or biomass and fetal growth outcomes: A multi-country randomised controlled trial

SUPPLEMENT

Data management for this analysis

```
rm(list = ls())

library(dplyr)
library(tidyverse)
library(knitr)
library(gridExtra)
library(cowplot)
library(dtplyr)
library(haven)
library(kableExtra)
library(ggpubr)
library(doit4me)
library(janitor)
library(broom)
library(Rmisc)
library(nlme)
library(splines)
library(gmodels)
library(data.table)
library(Rmisc)
library(mice)
library(mgcv)
library(gamm4)
library(biostat3)
```

```r
library(grid)
library(ggh4x)
library(here)
set.seed(443527)


### Functions

fun.efw = function(HC, AC, FL) {
    efw = 10^(1.326 + 0.0107 * HC + 0.0438 *
        AC + 0.158 * FL - 0.00326 * AC *
        FL)
    return(efw)
}

efw_zsore = function(EFW, GA) {
    skewness = 9.43643 + 9.41579 * ((GA/10)^(-2)) -
        83.5422 * log(GA/10) * ((GA/10)^(-2))
    mean = -2.42272 + 1.86478 * (GA^0.5) -
        1.93299e-05 * (GA^3)
    cov = 0.0193557 + 0.0310716 * ((GA/10)^(-2)) -
        0.0657587 * log(GA/10) * ((GA/10)^(-2))
    Y = log(EFW)

    zscore = ifelse(skewness == 0, ((cov)^(-1)) *
        log(Y/mean), ifelse(skewness != 0,
        ((cov * skewness)^(-1)) * (((Y/mean)^skewness) -
            1), NA))
    return(zscore)
}
```

Codebook

Variables	Code
hhid	Household id
irc	India, Peru, Guatemala, Rwanda
strata	1, . . . , 10
lpg	1=intervention, 0=control
ga	Gestional age at visit
gadays	Gestional age at visit.
visit	BL, P1, P2, BIRTH
dob	Date of birth
usdate	Date of ultrasonogram
fl	Femur length
hc	Head circumference
ac	Abdominal circumference
momage	Maternal age at enrollment
tv	1 yes, 0 no
mobile	1 yes, 0 no
radio	1 yes, 0 no
bicycle	1 yes, 0 no
bankacct	1 yes, 0 no
childhh	Number of children in household
sleephouse	Number of people that sleep in house
nullparous	1 yes, 0 no
x2handsmoke	1 someone other than pregnant woman smokes in house, 0 otherwise
momeduc	No formal education, some primary school, completed primary school, secondary school or higher
momhb	Maternal hemoglobin at enrollment
momhbalt	Altitude adjusted maternal hemoglobin at enrollment
foodinsecure	Food insecurity score
fiescat	Food insecurity score categories
momht	Maternal height
mommdd	Maternal diet diversity (1 yes, 0 no)
mommddcat	Maternal diet diversity score (low, medium, high)
garandom	Gestational age at randomization
flz	Femur length z-score
hcz	Head circumference z-score
acz	Abdominal circumference z-score
efwz	Estimated birthweight z-score
efw	Estimated fetal weight
pcassets	Principal component analysis of all asset variables
co	Personal exposure to carbon monoxide obtained during permitted window for visits
bc	Personal exposure to black carbon obtained during permitted window for visits
pm25	Personal exposure to PM2.5 obtained during permitted window for visits
birthspace	Spacing from last child
afi	Amniotic fluid index (cm)
fhr	Fetal heart rate (beats/min)

Data

```
df = fread("HAPIN_HE6_ITT_unbld_20230331_fmt.csv")

df.he1 <- fread("HAPIN_HE1_ITT_full_20220720_fmt.csv")

df.stunt = fread("HAPIN_HE4_ITT_unbld_20230705_unfmt.csv")

df.fhr <- fread("Abnormal_FHR_RC.csv")
df.afi <- fread("AFI data_ RC.csv")
df.alt <- fread("Altitude_Guatemala+Rwanda.csv")

df.stunt <- df.stunt %>%
    dplyr::filter(timepoint == "BL") %>%
    dplyr::rename(hhid = HHID) %>%
    dplyr::select(hhid, hypertension_pregnancy_P1,
        hypertension_pregnancy_P2, preeclampsia_reported)

df.stunt$htn <- ifelse(df.stunt$hypertension_pregnancy_P1 ==
    1 | df.stunt$hypertension_pregnancy_P2 ==
    1 | df.stunt$preeclampsia_reported ==
    1, 1, 0)

df.stunt$htn <- replace(df.stunt$htn, is.na(df.stunt$htn),
    0)

### Recoding/generating variables

dd2 = df %>%
    dplyr::select(HHID, rc_GA_at_birth_inDays,
        rc_GA_at_birth_inWks, Birthwt_by_protocol,
        timepoint, HHID, s6_arm, WeightZScore,
        Cong_Anom) %>%
    dplyr::filter(timepoint == "BL") %>%
    dplyr::filter(Cong_Anom != "Yes") %>%
    dplyr::select(-Cong_Anom) %>%
    dplyr::rename(hhid = HHID, visit = timepoint,
        lpg = s6_arm, efw = Birthwt_by_protocol,
        efwz = WeightZScore, ga = rc_GA_at_birth_inWks,
        gadays = rc_GA_at_birth_inDays) %>%
    dplyr::mutate(visit = "Birth") %>%
```

```r
    dplyr::filter(!is.na(ga))


df$strata = ifelse(df$s6_site == "", 1, ifelse(df$s6_site ==
    "Azangaro", 2, ifelse(df$s6_site == "Chucuito/Juli",
    3, ifelse(df$s6_site == "El Collao",
        4, ifelse(df$s6_site == "Huancane",
            5, ifelse(df$s6_site == "JALAPA",
                6, ifelse(df$s6_site == "Nagapattinam",
                  7, ifelse(df$s6_site ==
                    "Puno", 8, ifelse(df$s6_site ==
                    "San Roman", 9, ifelse(df$s6_site ==
                    "Villupuram", 10, NA))))))))))


### Socioeconomic index data
df.ses <- fread("df.ses.csv")


### Combining datasets

df <- df %>%
    dplyr::group_by(HHID) %>%
    dplyr::mutate(m17_RC_m17_date = as.Date(m17_RC_m17_date,
        format = "%d%b%Y"), DATE_FETAL_ULTRASOUND = as.Date(DATE_FETAL_ULTRASOUND,
        format = "%d%b%Y")) %>%
    fill(m17_rc_GA_at_screening, .direction = c("down")) %>%
    fill(m17_RC_m17_date, .direction = c("down")) %>%
    ungroup() %>%
    dplyr::mutate(gadays = as.numeric(m17_rc_GA_at_screening +
        (DATE_FETAL_ULTRASOUND - m17_RC_m17_date)),
        ga = as.numeric(gadays/7)) %>%
    dplyr::filter(Cong_Anom != "Yes") %>%
    dplyr::rename(hhid = HHID, irc = IRC,
        lpg = s6_arm, dob = c30_dob, ma1fe2 = c30_sex,
        visit = timepoint, usdate = DATE_FETAL_ULTRASOUND,
        gabirthdays = rc_GA_at_birth_inDays,
        gabirthweeks = rc_GA_at_birth_inWks,
        fl = FL_AVGc, hc = HC_AVGc, ac = AC_AVGc,
        tv = m10_color_tv, mobile = m10_phone,
        radio = m10_radio, bicycle = m10_bicycle,
```

```
        bankacct = m10_bank, childhh = m13_child,
        momage = MAyears_at_baseline, sleephouse = m10_sleep,
        nullparous = nulliparity, x2handsmoke = h43_tobacco,
        momeduc = m10_educ_R, momeducyr = m10_educ_year,
        momhb = b10_tm_hg, foodinsecure = fies,
        momht = m14_ave_ht_BL, mommdd = diet_diverse,
        mommddcat = diet_diverse_cat, garandom = GAweeks_AT_baseline,
        fiescat = fies_cat, flz = FL_zscore,
        hcz = HC_zscore, acz = AC_zscore,
        pwdate = m17_RC_m17_date, afi = M17M18_AFI,
        fhr = M17M18_FHR) %>%
    dplyr::select(hhid, irc, strata, lpg,
        dob, visit, ma1fe2, pwdate, usdate,
        gadays, ga, fl, hc, ac, tv, mobile,
        radio, bicycle, bankacct, childhh,
        fiescat, preterm, momage, sleephouse,
        nullparous, x2handsmoke, momeduc,
        momeducyr, momhb, foodinsecure, momht,
        mommdd, mommddcat, garandom, flz,
        hcz, acz, usdate, gabirthweeks, afi,
        fhr, Duration_screening_to_DOB) %>%
    dplyr::left_join(df.ses[, c("hhid", "pcassets")],
        by = c("hhid")) %>%
    dplyr::left_join(df.stunt[, c("hhid",
        "htn")])

# Create EFW and EFWZ variables
df$efw = fun.efw(HC = df$hc/10, AC = df$ac/10,
    FL = df$fl/10)
df$efwz = efw_zsore(df$efw, df$ga)


# varlist1 <- names(df)[!(names(df)
# %in% names(dd2))] dd2 <-
# left_join(dd2, df[df$visit ==
# 'BL',c('hhid', varlist1)], by =
# 'hhid') dd2$fl <- dd2$ac <- dd2$hc <-
# dd2$hcz <- dd2$acz <- dd2$flz <- NA

# join with dd2
df <- full_join(df, dd2, by = c("hhid", "visit",
```

```r
    "efw", "efwz", "lpg", "ga", "gadays")) %>%
    dplyr::mutate(dob = as.Date(dob), tv = ifelse(tv ==
        "Yes", 1, ifelse(tv == "No", 0, NA)),
        mobile = ifelse(mobile == "Yes",
            1, ifelse(mobile == "No", 0,
                NA)), radio = ifelse(radio ==
            "Yes", 1, ifelse(radio == "No",
            0, NA)), bicycle = ifelse(bicycle ==
            "Yes", 1, ifelse(bicycle == "No",
            0, NA)), bankacct = ifelse(bankacct ==
            "Yes", 1, ifelse(bankacct ==
            "No", 0, NA)), nullparous = ifelse(nullparous ==
            "Yes", 1, ifelse(nullparous ==
            "No", 0, NA)), x2handsmoke = ifelse(x2handsmoke ==
            "Yes", 1, ifelse(x2handsmoke ==
            "No", 0, NA)), mommdd = ifelse(mommdd ==
            "Yes", 1, ifelse(mommdd == "No",
            0, NA))) %>%
    dplyr::group_by(hhid) %>%
    fill(irc, strata, dob, pwdate, tv, mobile,
        radio, bicycle, bankacct, childhh,
        momage, sleephouse, nullparous, x2handsmoke,
        momeduc, momhb, foodinsecure, momht,
        mommdd, garandom) %>%
    dplyr::ungroup()


df$lpg = ifelse(df$lpg == "Intervention",
    1, 0)

df$gacat = ifelse(df$ga >= 14 & df$ga < 18,
    "14-17", ifelse(df$ga >= 18 & df$ga <
        22, "18-21", ifelse(df$ga >= 22 &
        df$ga < 26, "22-25", ifelse(df$ga >=
        26 & df$ga < 30, "26-29", ifelse(df$ga >=
        30 & df$ga < 34, "30-33", ifelse(df$ga >=
        34 & df$ga < 38, "34-37", ifelse(df$ga >=
        38 & df$ga < 46, "38-45", NA)))))))

df$gacat2 = ifelse(df$ga >= 14 & df$ga <
    18, "14-17", ifelse(df$ga >= 18 & df$ga <
```

```r
    22, "18-21", ifelse(df$ga >= 22 & df$ga <
    26, "22-25", ifelse(df$ga >= 26 & df$ga <
    30, "26-29", ifelse(df$ga >= 30 & df$ga <
    34, "30-33", ifelse(df$ga >= 34, "34-45",
    NA))))))

df.alt <- df.alt %>%
    dplyr::rename(hhid = id)

df <- left_join(df, df.alt, by = c("hhid",
    "irc"))

df$altitude <- ifelse(df$irc == "Peru", 12556/1000,
    ifelse(df$irc %in% c("Rwanda", "Guatemala"),
        ((df$altitude * 3.28084)/1000), NA))

df$altitude <- ifelse(is.na(df$altitude) &
    df$irc == "Rwanda", mean(df$altitude[df$irc ==
    "Rwanda"], na.rm = TRUE), df$altitude)

df$alt_adjust <- ((-0.032) * df$altitude) +
    (0.022 * (df$altitude^2))

df$momhbalt = ifelse(df$irc == "India", df$momhb,
    ifelse(df$irc != "India" & df$alt_adjust >
        0, df$momhb - df$alt_adjust, NA))

### Cleaning AFI df$afi <-
### ifelse(df$afi > 50, NA, df$afi)
### df$fhr <- ifelse(df$fhr <100 |
### df$fhr>200, NA, df$fhr)


df.he1 <- df.he1 %>%
    dplyr::rename(hhid = HHID, co_BL = CO_avg_ppm_M_BL,
        co_P1 = CO_avg_ppm_M_P1, co_P2 = CO_avg_ppm_M_P2,
        bc_BL = ECM_bc_conc_M_BL, bc_P1 = ECM_bc_conc_M_P1,
        bc_P2 = ECM_bc_conc_M_P2, pm_BL = ECM_grav_neph_conc_M_BL,
        pm_P1 = ECM_grav_neph_conc_M_P1,
        pm_P2 = ECM_grav_neph_conc_M_P2,
        hapdate_BL = ecm_date_start_m_BL,
```

```
        hapdate_P1 = ecm_date_start_m_P1,
        hapdate_P2 = ecm_date_start_m_P2) %>%
    dplyr::select(hhid, co_BL, co_P1, co_P2,
        bc_BL, bc_P1, bc_P2, pm_BL, pm_P1,
        pm_P2, hapdate_BL, hapdate_P1, hapdate_P2)

df.he1 <- data.frame(df.he1)


df.he1_co <- df.he1 %>%
    dplyr::select(hhid, contains("co_")) %>%
    pivot_longer(cols = starts_with("co_"),
        names_to = "visit", names_prefix = "co_",
        values_to = "co", values_drop_na = TRUE)

df.he1_bc <- df.he1 %>%
    dplyr::select(hhid, contains("bc_")) %>%
    pivot_longer(cols = starts_with("bc_"),
        names_to = "visit", names_prefix = "bc_",
        values_to = "bc", values_drop_na = TRUE)

df.he1_pm <- df.he1 %>%
    dplyr::select(hhid, contains("pm_")) %>%
    pivot_longer(cols = starts_with("pm_"),
        names_to = "visit", names_prefix = "pm_",
        values_to = "pm", values_drop_na = TRUE)

df.he1_hapdate <- df.he1 %>%
    dplyr::select(hhid, contains("hapdate_")) %>%
    pivot_longer(cols = starts_with("hapdate_"),
        names_to = "visit", names_prefix = "hapdate_",
        values_to = "hapdate", values_drop_na = TRUE) %>%
    dplyr::mutate(hapdate = as.Date(hapdate,
        format = "%d%B%Y"))

df.he1x <- full_join(df.he1_co, df.he1_bc,
    by = c("hhid", "visit")) %>%
    full_join(df.he1_pm, by = c("hhid", "visit")) %>%
    full_join(df.he1_hapdate, by = c("hhid",
        "visit"))
```

```r
df <- as.data.frame(left_join(df, df.he1x,
    by = c("hhid", "visit")))


df.fhr <- df.fhr %>%
    dplyr::select(HHID, timepoint, Correction) %>%
    dplyr::rename(hhid = HHID, visit = timepoint,
        fhr_c = Correction) %>%
    dplyr::mutate(fhr_c = as.numeric(fhr_c))

df <- left_join(df, df.fhr, by = c("hhid",
    "visit"))

df.afi <- df.afi %>%
    dplyr::select(HHID, timepoint, Correction) %>%
    dplyr::rename(hhid = HHID, visit = timepoint,
        afi_c = Correction) %>%
    dplyr::mutate(afi_c = as.numeric(afi_c))

df <- left_join(df, df.afi, by = c("hhid",
    "visit"))

df$fhr <- ifelse(!is.na(df$fhr_c), df$fhr_c,
    df$fhr)
df$afi <- ifelse(!is.na(df$afi_c), df$afi_c,
    df$afi)
df$afi <- ifelse(df$afi > 100, NA, df$afi)


tab.exp <- df %>%
    dplyr::filter(!is.na(hc) | !is.na(ac) |
        !is.na(fl) | !is.na(efw)) %>%
    dplyr::filter(visit == "P1" | visit ==
        "P2") %>%
    dplyr::group_by(lpg) %>%
    dplyr::summarise(n = n(), mean_pm = mean(pm,
        na.rm = TRUE), sd_pm = sd(pm, na.rm = TRUE),
        mean_bc = mean(bc, na.rm = TRUE),
        sd_bc = sd(bc, na.rm = TRUE), mean_co = mean(co,
            na.rm = TRUE), sd_co = sd(co,
            na.rm = TRUE), lab_pm = paste0(round(mean_pm,
```

```
            1), " (", round(sd_pm, 1), ")"),
        lab_bc = paste0(round(mean_bc, 1),
            " (", round(sd_bc, 1), ")"),
        lab_co = paste0(round(mean_co, 1),
            " (", round(sd_co, 1), ")"))


write.csv(df, "Fetal_growth_03-07-2022.csv")
write.csv(df.he1x, "he1_clean_03-24-2022.csv")
```

Statistical code for the analysis

The main analysis was intention-to-treat (ITT) with Z-scores of foetal growth outcomes (HC, AC, FL and EFW/birthweight) modelled as continuous variables. As foetal growth outcomes were considered secondary outcomes, there was no formal sample size calculation. Given that we have four foetal growth outcomes, the threshold for statistical significance was set at $0 \cdot 0125$ and we present $98 \cdot 75\%$ CIs to control the familywise Type I error rate at $0 \cdot 05$. EFW and birthweight are thought to represent a continuum in the gestational age time scale [24, 25]. We therefore combined EFW Z-scores and birthweight-for-gestational-age (BAZ) into a single outcome variable (EFWZ/BAZ) in our regression models.

We used multivariable linear regressions with the post-randomisation Z-scores as the outcome and the intervention arm as the main covariate, adjusted for randomisation strata. We first ran regressions to estimate difference in foetal growth Z-scores for P1 and P2 separately and then ran regressions to estimate differences in the average Z-scores of P1 and P2 (if only one visit was available, we used the measurement in that visit as the average). We also ran regressions for BAZ at birth separately and for the averaged EFWZ/BAZ between P1 and birth. We considered a difference in Z-score of $0 \cdot 2$ SDs as a clinically significant difference based on the work by Sudfeld et al. [26], who showed that an effect size of $0 \cdot 2$ SDs is a commonly observed magnitude of change in anthropometric indices due to nutrition-specific and nutrition-sensitive interventions.

We also modelled the Z-scores obtained at the BL, P1 and P2 visits (and at birth for EFWZ/BAZ) using a generalised additive model (GAM) [27] with a tensor smooth for GA stratified by intervention arm and time of randomisation above or below the median (i.e., for this analysis, this corresponded to a GA $<15 \cdot 3$ or $\geq 15 \cdot 3$ weeks) and adjusted for randomisation strata. We estimated median differences in Z-scores by GA between intervention and control participants at 25 and 33 weeks of gestation (median GA for P1 and P2 visits, respectively) and at 40 weeks for EFW, stratified by gestational age at randomisation. We accounted for heterogeneity across participants using a random intercept and a random slope.

Finally, we also conducted exposure-response (E-R) analyses using the longitudinal foetal growth Z-scores as continuous variables and 24-hour personal exposures to $PM2 \cdot 5$, BC, and CO as the main covariate, also continuous, obtained at the BL, P1 and P2 visits (and at birth for EFWZ/BAZ). For the E-R analyses, growth measurements obtained at BL were paired with pollutant concentrations measured at BL for regression, those obtained at P1 were paired with the average of pollutant concentrations obtained at BL and P1, those obtained at P2 were paired with the average of pollutant concentrations obtained at BL, P1 and P2, and BAZ was paired with average pollutant concentrations across BL, P1, and P2. Means and standard deviation of Z-scores by visit and their paired pollutant concentrations in quartiles were tabulated. GAMs were used with the Z-scores as the outcome and the following covariates selected a priori and consistent with variables included in the exposure-response analysis for birthweight [28]: tensor surface smooth for the interaction between pollutant and GA, principal components analysis derived socioeconomic status index [29], maternal age, nulliparity, minimum diet diversity for

women categorised as either high (>5) or low (<5) [8], maternal education, altitude-adjusted maternal haemoglobin [30], and self-reported exposure to second-hand smoke. We accounted for heterogeneity across participants using a random intercept. To ensure comparability with the ITT analysis, we computed differences in Z-scores at 25 and 33 weeks (and at 40 weeks for EFW) across the interquartile differences of the paired pollutant concentrations and set the threshold for statistical significance at $0 \cdot 0125$.

```
### Setting data df2 removes missing
### data and creates categories for LPG

# df2 = subset(df,
# select=c(hhid,ga,visit,
# lpg,garandom,flz,hcz,acz,efwz,strata))

df2 <- df %>%
    dplyr::select(hhid, ga, visit, lpg, garandom,
        flz, hcz, acz, efwz, strata) %>%
    dplyr::filter(!is.na(ga)) %>%
    dplyr::filter(!is.na(efwz)) %>%
    dplyr::filter(!(visit %in% c("BL", "P1",
        "P2") & is.na(flz))) %>%
    dplyr::filter(!(visit %in% c("BL", "P1",
        "P2") & is.na(hcz))) %>%
    dplyr::filter(!(visit %in% c("BL", "P1",
        "P2") & is.na(acz)))

# dim(df2) colSums(is.na(df2))
# table(df2$visit)


# df2 = df2[is.na(df2$ga)==F,] df2 =
# df2[is.na(df2$flz)==F,] df2 =
# df2[is.na(df2$hcz)==F,] df2 =
# df2[is.na(df2$acz)==F,] df2 =
# df2[is.na(df2$efwz)==F,] df2 =
# df2[is.na(df2$garandom)==F,] df2 =
# df2[is.na(df2$hhid)==F,]
df2$lpg3 = ifelse(df2$lpg == 1, ifelse(df2$garandom >=
    16.1, 2, 1), 0)
df2$lpg3 = factor(df2$lpg3)
df2 = df2[substr(rownames(df2), 1, 2) !=
    "NA", ]
```

```r
df3 = df2 %>%
    # dplyr::filter(visit != 'Birth')
    # %>% dplyr::distinct(hhid, visit,
    # .keep_all = TRUE) %>%
dplyr::select(hhid, flz, hcz, acz, efwz,
    ga, lpg3, strata, visit) %>%
    dplyr::mutate(hhid = factor(hhid))

# df3 = subset(df2, select=c(hhid, flz,
# hcz, acz, efwz, ga, lpg3,strata))
# df3$hhid = factor(df3$hhid)

### Remove outliers less than -6s or
### more than 6

df2 = df2[df2$hcz > (-6) & df2$hcz < 6 |
    is.na(df2$hcz), ]
df2 = df2[df2$acz > (-6) & df2$acz < 6 |
    is.na(df2$acz), ]
df2 = df2[df2$flz > (-6) & df2$flz < 6 |
    is.na(df2$flz), ]
df2 = df2[df2$efwz > (-6) & df2$efwz < 6 |
    is.na(df2$efwz), ]


### Establishing median gestational age
### times
median(df2$ga[df2$visit == "P1"], na.rm = T)
```

```
[1] 25
```

```r
median(df2$ga[df2$visit == "P2"], na.rm = T)
```

```
[1] 33.14286
```

```r
### Models and dataset for Figure-4

tab4 <- data.frame(matrix(ncol = 13, nrow = 4))
colnames(tab4) <- c("visits", "hc", "hc.lb",
```

```r
    "hc.ub", "ac", "ac.lb", "ac.ub", "fl",
    "fl.lb", "fl.ub", "efw", "efw.lb", "efw.ub")

tab4$visits <- c("P1", "P2", "Birth", "All")
rownames(tab4) <- c("P1", "P2", "Birth",
    "All")

fun.tab4 <- function(model, visit, var) {
    tab4[[var]][which(tab4$visits == visit)] <- lincom(model,
        c("lpg"), level = 0.9875)[["Estimate"]]
    tab4[[paste0(var, ".lb")]][which(tab4$visits ==
        visit)] <- lincom(model, c("lpg"),
        level = 0.9875)[["0.625 %"]]
    tab4[[paste0(var, ".ub")]][which(tab4$visits ==
        visit)] <- lincom(model, c("lpg"),
        level = 0.9875)[["99.375 %"]]
    return(tab4)
}

### Initial models Separate by visit, 9
### models
model.flz.p1 = lm(flz ~ lpg + factor(strata),
    data = df2, subset = visit == "P1")
summary(model.flz.p1)
```

```
Call:
lm(formula = flz ~ lpg + factor(strata), data = df2, subset = visit ==
    "P1")

Residuals:
    Min      1Q  Median      3Q     Max
-5.1647 -0.7242 -0.0305  0.7092  4.2970

Coefficients:
                 Estimate Std. Error t value Pr(>|t|)
(Intercept)       0.49475    0.04540  10.899  < 2e-16 ***
lpg              -0.03769    0.04341  -0.868  0.38533
factor(strata)2  -0.01494    0.12862  -0.116  0.90751
factor(strata)3  -0.14403    0.13490  -1.068  0.28578
factor(strata)4  -0.19585    0.14668  -1.335  0.18195
factor(strata)5  -0.32416    0.13324  -2.433  0.01505 *
```

```
factor(strata)6  -0.03222    0.05652  -0.570  0.56863
factor(strata)7   0.59579    0.08450   7.051 2.29e-12 ***
factor(strata)8  -0.33053    0.07478  -4.420 1.03e-05 ***
factor(strata)9  -0.40153    0.10425  -3.851  0.00012 ***
factor(strata)10 -0.09867    0.11211  -0.880  0.37887
---
Signif. codes:  0 '***' 0.001 '**' 0.01 '*' 0.05 '.' 0.1 ' ' 1

Residual standard error: 1.093 on 2525 degrees of freedom
Multiple R-squared:  0.04444,   Adjusted R-squared:  0.04066
F-statistic: 11.74 on 10 and 2525 DF,  p-value: < 2.2e-16
```

```
lincom(model.flz.p1, c("lpg"), level = 0.9875)
```

```
       Estimate    0.625 %   99.375 % Df Sum of Sq
lpg -0.03769443 -0.1461289 0.07074004  1 0.9004196
```

```
tab4 <- fun.tab4(model.flz.p1, "P1", "fl")

model.flz.p2 = lm(flz ~ lpg + factor(strata),
    data = df2, subset = visit == "P2")
summary(model.flz.p2)
```

```
Call:
lm(formula = flz ~ lpg + factor(strata), data = df2, subset = visit ==
    "P2")

Residuals:
    Min      1Q  Median      3Q     Max
-5.5980 -0.6727  0.0090  0.6923  3.5343

Coefficients:
                 Estimate Std. Error t value Pr(>|t|)
(Intercept)       0.50797    0.04413  11.510  < 2e-16 ***
lpg               0.01909    0.04281   0.446  0.65562
factor(strata)2  -0.17909    0.12225  -1.465  0.14308
factor(strata)3  -0.27010    0.13838  -1.952  0.05106 .
factor(strata)4  -0.17968    0.14367  -1.251  0.21118
factor(strata)5  -0.26735    0.12868  -2.078  0.03784 *
```

```
factor(strata)6  -0.09953    0.05540  -1.797  0.07250 .
factor(strata)7   0.63441    0.09050   7.010 3.07e-12 ***
factor(strata)8  -0.21926    0.07387  -2.968  0.00303 **
factor(strata)9  -0.35165    0.10486  -3.354  0.00081 ***
factor(strata)10 -0.21160    0.09843  -2.150  0.03166 *
---
Signif. codes:  0 '***' 0.001 '**' 0.01 '*' 0.05 '.' 0.1 ' ' 1

Residual standard error: 1.062 on 2455 degrees of freedom
Multiple R-squared:  0.03956,   Adjusted R-squared:  0.03564
F-statistic: 10.11 on 10 and 2455 DF,  p-value: < 2.2e-16
```

```r
lincom(model.flz.p2, c("lpg"), level = 0.9875)
```

```
      Estimate     0.625 %  99.375 % Df Sum of Sq
lpg 0.01909249 -0.08782442 0.1260094  1 0.2245358
```

```r
tab4 <- fun.tab4(model.flz.p2, "P2", "fl")


model.acz.p1 = lm(acz ~ lpg + factor(strata),
    data = df2, subset = visit == "P1")
summary(model.acz.p1)
```

```
Call:
lm(formula = acz ~ lpg + factor(strata), data = df2, subset = visit ==
    "P1")

Residuals:
    Min      1Q  Median      3Q     Max
-3.5682 -0.7040  0.0093  0.6760  3.7217

Coefficients:
                 Estimate Std. Error t value Pr(>|t|)
(Intercept)       0.54188    0.04372  12.395  < 2e-16 ***
lpg              -0.01822    0.04181  -0.436 0.663068
factor(strata)2   0.86267    0.12386   6.965 4.18e-12 ***
factor(strata)3   0.40626    0.12992   3.127 0.001786 **
factor(strata)4   0.74655    0.14126   5.285 1.37e-07 ***
```

```
factor(strata)5   0.49655    0.12831   3.870 0.000112 ***
factor(strata)6  -0.35324    0.05443  -6.490 1.03e-10 ***
factor(strata)7  -0.79680    0.08138  -9.791  < 2e-16 ***
factor(strata)8   0.51482    0.07201   7.149 1.14e-12 ***
factor(strata)9   0.58659    0.10040   5.842 5.80e-09 ***
factor(strata)10 -0.87037    0.10797  -8.062 1.15e-15 ***
---
Signif. codes:  0 '***' 0.001 '**' 0.01 '*' 0.05 '.' 0.1 ' ' 1

Residual standard error: 1.052 on 2525 degrees of freedom
Multiple R-squared:  0.1656,    Adjusted R-squared:  0.1623
F-statistic:  50.1 on 10 and 2525 DF,  p-value: < 2.2e-16
```

```r
lincom(model.acz.p1, c("lpg"), level = 0.9875)
```

```
       Estimate    0.625 %   99.375 % Df Sum of Sq
lpg -0.01821755 -0.1226442 0.08620907  1 0.2103152
```

```r
tab4 <- fun.tab4(model.acz.p1, "P1", "ac")

model.acz.p2 = lm(acz ~ lpg + factor(strata),
    data = df2, subset = visit == "P2")
summary(model.acz.p2)
```

```
Call:
lm(formula = acz ~ lpg + factor(strata), data = df2, subset = visit ==
    "P2")

Residuals:
    Min      1Q  Median      3Q     Max
-4.5818 -0.6633 -0.0092  0.6836  3.4279

Coefficients:
                 Estimate Std. Error t value Pr(>|t|)
(Intercept)       0.60076    0.04288  14.010   <2e-16 ***
lpg               0.01969    0.04159   0.473   0.6360
factor(strata)2   0.20565    0.11879   1.731   0.0835 .
factor(strata)3  -0.03829    0.13445  -0.285   0.7758
factor(strata)4   0.30610    0.13959   2.193   0.0284 *
```

```
factor(strata)5   0.22022    0.12503   1.761   0.0783 .
factor(strata)6  -0.61794    0.05383 -11.480   <2e-16 ***
factor(strata)7  -0.86193    0.08793  -9.802   <2e-16 ***
factor(strata)8   0.15512    0.07178   2.161   0.0308 *
factor(strata)9   0.18467    0.10189   1.813   0.0700 .
factor(strata)10 -1.18007    0.09564 -12.339   <2e-16 ***
---
Signif. codes:  0 '***' 0.001 '**' 0.01 '*' 0.05 '.' 0.1 ' ' 1

Residual standard error: 1.032 on 2455 degrees of freedom
Multiple R-squared:  0.1473,    Adjusted R-squared:  0.1438
F-statistic: 42.41 on 10 and 2455 DF,  p-value: < 2.2e-16
```

```
lincom(model.acz.p2, c("lpg"), level = 0.9875)
```

```
      Estimate     0.625 %  99.375 % Df Sum of Sq
lpg 0.01968545 -0.08420076 0.1235717  1 0.2386993
```

```
tab4 <- fun.tab4(model.acz.p2, "P2", "ac")

model.hcz.p1 = lm(hcz ~ lpg + factor(strata),
    data = df2, subset = visit == "P1")
summary(model.hcz.p1)
```

```
Call:
lm(formula = hcz ~ lpg + factor(strata), data = df2, subset = visit ==
    "P1")

Residuals:
    Min      1Q  Median      3Q     Max
-4.4580 -0.6819 -0.0407  0.6717  3.9730

Coefficients:
                 Estimate Std. Error t value Pr(>|t|)
(Intercept)       0.76459    0.04394  17.402  < 2e-16 ***
lpg              -0.11710    0.04202  -2.787  0.00536 **
factor(strata)2  -0.15352    0.12448  -1.233  0.21761
factor(strata)3  -0.25282    0.13057  -1.936  0.05294 .
factor(strata)4  -0.03837    0.14197  -0.270  0.78696
```

```
factor(strata)5  -0.65842    0.12896  -5.106 3.54e-07 ***
factor(strata)6  -0.51194    0.05470  -9.359  < 2e-16 ***
factor(strata)7  -0.16603    0.08179  -2.030  0.04246 *
factor(strata)8  -0.31353    0.07238  -4.332 1.54e-05 ***
factor(strata)9  -0.29817    0.10090  -2.955  0.00316 **
factor(strata)10 -0.79068    0.10851  -7.287 4.22e-13 ***
---
Signif. codes:  0 '***' 0.001 '**' 0.01 '*' 0.05 '.' 0.1 ' ' 1

Residual standard error: 1.058 on 2525 degrees of freedom
Multiple R-squared:  0.0521,    Adjusted R-squared:  0.04835
F-statistic: 13.88 on 10 and 2525 DF,  p-value: < 2.2e-16
```

```
lincom(model.hcz.p1, c("lpg"), level = 0.9875)
```

```
     Estimate    0.625 %    99.375 % Df Sum of Sq
lpg -0.117103 -0.2220544 -0.01215167  1  8.690145
```

```
tab4 <- fun.tab4(model.hcz.p1, "P1", "hc")

model.hcz.p2 = lm(hcz ~ lpg + factor(strata),
    data = df2, subset = visit == "P2")
summary(model.hcz.p2)
```

```
Call:
lm(formula = hcz ~ lpg + factor(strata), data = df2, subset = visit ==
    "P2")

Residuals:
    Min      1Q  Median      3Q     Max
-3.6585 -0.7370 -0.0211  0.7166  4.1655

Coefficients:
                  Estimate Std. Error t value Pr(>|t|)
(Intercept)       0.937590   0.045756  20.491  < 2e-16 ***
lpg              -0.003226   0.044381  -0.073    0.942
factor(strata)2  -0.734664   0.126751  -5.796 7.66e-09 ***
factor(strata)3  -0.856603   0.143466  -5.971 2.70e-09 ***
factor(strata)4  -0.219497   0.148951  -1.474    0.141
```

```
factor(strata)5  -0.867563   0.133412  -6.503 9.51e-11 ***
factor(strata)6  -1.012188   0.057433 -17.624  < 2e-16 ***
factor(strata)7  -0.722379   0.093828  -7.699 1.97e-14 ***
factor(strata)8  -0.773429   0.076591 -10.098  < 2e-16 ***
factor(strata)9  -0.683529   0.108717  -6.287 3.81e-10 ***
factor(strata)10 -1.488038   0.102048 -14.582  < 2e-16 ***
---
Signif. codes:  0 '***' 0.001 '**' 0.01 '*' 0.05 '.' 0.1 ' ' 1

Residual standard error: 1.101 on 2455 degrees of freedom
Multiple R-squared:  0.1511,    Adjusted R-squared:  0.1477
F-statistic: 43.71 on 10 and 2455 DF,  p-value: < 2.2e-16
```

```r
lincom(model.hcz.p2, c("lpg"), level = 0.9875)
```

```
       Estimate    0.625 %  99.375 % Df   Sum of Sq
lpg -0.00322625 -0.1140766 0.1076241  1 0.006411453
```

```r
tab4 <- fun.tab4(model.hcz.p2, "P2", "hc")

model.efwz.p1 = lm(efwz ~ lpg + factor(strata),
    data = df2, subset = visit == "P1")
summary(model.efwz.p1)
```

```
Call:
lm(formula = efwz ~ lpg + factor(strata), data = df2, subset = visit ==
    "P1")

Residuals:
    Min      1Q  Median      3Q     Max
-4.7081 -0.6922  0.0177  0.7114  4.0612

Coefficients:
                 Estimate Std. Error t value Pr(>|t|)
(Intercept)       0.69475    0.04650  14.942  < 2e-16 ***
lpg              -0.05170    0.04447  -1.163   0.2451
factor(strata)2   0.51728    0.13173   3.927 8.84e-05 ***
factor(strata)3   0.12861    0.13817   0.931   0.3521
factor(strata)4   0.38188    0.15024   2.542   0.0111 *
```

```
factor(strata)5   0.03300    0.13647   0.242   0.8089    
factor(strata)6  -0.36813    0.05789  -6.360 2.39e-10 ***
factor(strata)7  -0.34572    0.08655  -3.995 6.67e-05 ***
factor(strata)8   0.11692    0.07659   1.527   0.1270    
factor(strata)9   0.13648    0.10678   1.278   0.2013    
factor(strata)10 -0.82340    0.11483  -7.171 9.74e-13 ***
---
Signif. codes:  0 '***' 0.001 '**' 0.01 '*' 0.05 '.' 0.1 ' ' 1

Residual standard error: 1.119 on 2525 degrees of freedom
Multiple R-squared:  0.06027,   Adjusted R-squared:  0.05654 
F-statistic: 16.19 on 10 and 2525 DF,  p-value: < 2.2e-16
```

```
lincom(model.efwz.p1, c("lpg"), level = 0.9875)
```

```
       Estimate     0.625 %   99.375 % Df Sum of Sq
lpg -0.05170036 -0.1627626 0.05936184  1  1.693861
```

```
tab4 <- fun.tab4(model.efwz.p1, "P1", "efw")

model.efwz.p2 = lm(efwz ~ lpg + factor(strata),
    data = df2, subset = visit == "P2")
summary(model.efwz.p2)
```

```
Call:
lm(formula = efwz ~ lpg + factor(strata), data = df2, subset = visit == 
    "P2")

Residuals:
    Min      1Q  Median      3Q     Max 
-5.5070 -0.6337  0.0189  0.6456  3.3221 

Coefficients:
                 Estimate Std. Error t value Pr(>|t|)    
(Intercept)       0.65921    0.04222  15.612  < 2e-16 ***
lpg               0.01768    0.04096   0.432   0.6659    
factor(strata)2  -0.06403    0.11697  -0.547   0.5841    
factor(strata)3  -0.28496    0.13239  -2.152   0.0315 *  
factor(strata)4   0.11483    0.13745   0.835   0.4036    
```

```
factor(strata)5  -0.10860    0.12311  -0.882   0.3778    
factor(strata)6  -0.66809    0.05300 -12.605  < 2e-16 ***
factor(strata)7  -0.54112    0.08659  -6.250 4.84e-10 ***
factor(strata)8  -0.11482    0.07068  -1.625   0.1044    
factor(strata)9  -0.11188    0.10033  -1.115   0.2649    
factor(strata)10 -1.19919    0.09417 -12.734  < 2e-16 ***
---
Signif. codes:  0 '***' 0.001 '**' 0.01 '*' 0.05 '.' 0.1 ' ' 1

Residual standard error: 1.016 on 2455 degrees of freedom
Multiple R-squared:  0.1109,    Adjusted R-squared:  0.1073 
F-statistic: 30.63 on 10 and 2455 DF,  p-value: < 2.2e-16
```

```r
lincom(model.efwz.p2, c("lpg"), level = 0.9875)
```

```
      Estimate     0.625 %  99.375 % Df Sum of Sq
lpg 0.01768418 -0.08460989 0.1199782  1 0.1926327
```

```r
tab4 <- fun.tab4(model.efwz.p2, "P2", "efw")

# model.efwz.birth = lm ( efwz ~ lpg +
# factor(strata), data = df2,
# subset=visit=='Birth' )
# summary(model.efwz.birth) lincom
# (model.efwz.birth,
# c('lpg'),level=0.9875)

### Can do it more elegantly in 4
### models
model.flz.p1p2 = lm(flz ~ lpg * factor(visit) +
    factor(strata), data = df2, subset = visit ==
    "P1" | visit == "P2")
summary(model.flz.p1p2)
```

```
Call:
lm(formula = flz ~ lpg * factor(visit) + factor(strata), data = df2, 
    subset = visit == "P1" | visit == "P2")

Residuals:
```

```
    Min      1Q  Median      3Q     Max 
-5.6251 -0.6967 -0.0078  0.6971  4.3233 

Coefficients:
                      Estimate Std. Error t value Pr(>|t|)    
(Intercept)          0.5016238  0.0382647  13.109  < 2e-16 ***
lpg                 -0.0376838  0.0428002  -0.880  0.37865    
factor(visit)P2     -0.0006981  0.0432952  -0.016  0.98714    
factor(strata)2     -0.0988804  0.0886565  -1.115  0.26477    
factor(strata)3     -0.2037971  0.0965331  -2.111  0.03481 *  
factor(strata)4     -0.1879185  0.1026490  -1.831  0.06721 .  
factor(strata)5     -0.2955501  0.0925887  -3.192  0.00142 ** 
factor(strata)6     -0.0653647  0.0395637  -1.652  0.09857 .  
factor(strata)7      0.6118679  0.0616421   9.926  < 2e-16 ***
factor(strata)8     -0.2764239  0.0525504  -5.260 1.50e-07 ***
factor(strata)9     -0.3779046  0.0739068  -5.113 3.28e-07 ***
factor(strata)10    -0.1609911  0.0740499  -2.174  0.02975 *  
lpg:factor(visit)P2  0.0572104  0.0609557   0.939  0.34800    
---
Signif. codes:  0 '***' 0.001 '**' 0.01 '*' 0.05 '.' 0.1 ' ' 1

Residual standard error: 1.078 on 4989 degrees of freedom
Multiple R-squared:  0.04116,   Adjusted R-squared:  0.03886 
F-statistic: 17.85 on 12 and 4989 DF,  p-value: < 2.2e-16
```

```
lincom(model.flz.p1p2, c("lpg", "lpg+lpg:factor(visit)P2"),
    level = 0.9875)
```

```
                        Estimate    0.625 %    99.375 %   Df Sum of Sq
lpg                     -0.03768382 -0.1445861 0.06921844 1  0.9001081
lpg+lpg:factor(visit)P2 0.01952655  -0.0888951 0.1279482  1  0.2349513
```

```
model.acz.p1p2 = lm(acz ~ lpg * factor(visit) +
    factor(strata), data = df2, subset = visit ==
    "P1" | visit == "P2")
lincom(model.acz.p1p2, c("lpg", "lpg+lpg:factor(visit)P2"),
    level = 0.9875)
```

```
                        Estimate   0.625 %     99.375 %   Df Sum of Sq
lpg                     -0.0157391 -0.1194347  0.08795654 1  0.1570163
lpg+lpg:factor(visit)P2 0.0170885  -0.08808096 0.122258   1  0.1799429
```

```
model.hcz.p1p2 = lm(hcz ~ lpg * factor(visit) +
    factor(strata), data = df2, subset = visit ==
    "P1" | visit == "P2")
lincom(model.hcz.p1p2, c("lpg", "lpg+lpg:factor(visit)P2"),
    level = 0.9875)
```

```
                        Estimate     0.625 %    99.375 %     Df Sum of Sq
lpg                     -0.1133197   -0.2209941 -0.005645247 1  8.139458
lpg+lpg:factor(visit)P2 -0.008076766 -0.1172816 0.101128     1  0.04019773
```

```
df2$visit <- factor(df2$visit, levels = c("BL",
    "P1", "P2", "Birth"))

model.efwz.p1p2 = lm(efwz ~ lpg * factor(visit) +
    factor(strata), data = df2, subset = visit ==
    "P1" | visit == "P2" | visit == "Birth")
lincom(model.efwz.p1p2, c("lpg", "lpg+lpg:factor(visit)P2",
    "lpg+lpg:factor(visit)Birth"), level = 0.9875)
```

```
                           Estimate    0.625 %     99.375 %   Df Sum of Sq
lpg                        -0.05061054 -0.1515674  0.05034635 1  1.623684
lpg+lpg:factor(visit)P2    0.01267535  -0.08971352 0.1150642  1  0.09901614
lpg+lpg:factor(visit)Birth 0.03610224  -0.05723767 0.1294421  1  0.9665518
```

```
efw.lincom <- data.frame(lincom(model.efwz.p1p2,
    c("lpg", "lpg+lpg:factor(visit)P2", "lpg+lpg:factor(visit)Birth"),
    level = 0.9875))

tab4["Birth", "efw"] <- efw.lincom["lpg+lpg:factor(visit)Birth",
    "Estimate"]
tab4["Birth", "efw.lb"] <- efw.lincom["lpg+lpg:factor(visit)Birth",
    "X0.625.."]
tab4["Birth", "efw.ub"] <- efw.lincom["lpg+lpg:factor(visit)Birth",
    "X99.375.."]


### Overall, average of P1 and P2
df.flz = aggregate(flz ~ hhid + lpg + strata,
    data = df, FUN = mean, subset = visit ==
```

```
        "P1" | visit == "P2")
df.acz = aggregate(acz ~ hhid + lpg + strata,
    data = df, FUN = mean, subset = visit ==
        "P1" | visit == "P2")
df.hcz = aggregate(hcz ~ hhid + lpg + strata,
    data = df, FUN = mean, subset = visit ==
        "P1" | visit == "P2")
df.efwz = aggregate(efwz ~ hhid + lpg + strata,
    data = df, FUN = mean, subset = visit ==
        "P1" | visit == "P2" | visit == "Birth")

### FLZ Confirm that aggregate
### adequately calculates mean if one
### value missing, just the other value
df.flz.p1 = aggregate(flz ~ hhid + lpg +
    strata, data = df, FUN = mean, subset = visit ==
    "P1")
names(df.flz.p1)[4] = "flz1"
df.flz.p2 = aggregate(flz ~ hhid + lpg +
    strata, data = df, FUN = mean, subset = visit ==
    "P2")
names(df.flz.p2)[4] = "flz2"

df.flz.p1p2 = full_join(df.flz.p1, df.flz.p2[,
    c("hhid", "lpg", "flz2")], by = c("hhid",
    "lpg"))

df.flz.p1p2$avgflz = rowMeans(df.flz.p1p2[,
    c(4, 5)], na.rm = T)


### HCZ
df.hcz.p1 = aggregate(hcz ~ hhid + lpg +
    strata, data = df, FUN = mean, subset = visit ==
    "P1")
names(df.hcz.p1)[4] = "hcz1"
df.hcz.p2 = aggregate(hcz ~ hhid + lpg +
    strata, data = df, FUN = mean, subset = visit ==
    "P2")
names(df.hcz.p2)[4] = "hcz2"
```

```r
df.hcz.p1p2 = full_join(df.hcz.p1, df.hcz.p2[,
    c("hhid", "lpg", "hcz2")], by = c("hhid",
    "lpg"))

df.hcz.p1p2$avghcz = rowMeans(df.hcz.p1p2[,
    c(4, 5)], na.rm = T)

### ACZ
df.acz.p1 = aggregate(acz ~ hhid + lpg +
    strata, data = df, FUN = mean, subset = visit ==
    "P1")
names(df.acz.p1)[4] = "acz1"
df.acz.p2 = aggregate(acz ~ hhid + lpg +
    strata, data = df, FUN = mean, subset = visit ==
    "P2")
names(df.acz.p2)[4] = "acz2"

df.acz.p1p2 = full_join(df.acz.p1, df.acz.p2[,
    c("hhid", "lpg", "acz2")], by = c("hhid",
    "lpg"))

df.acz.p1p2$avgacz = rowMeans(df.acz.p1p2[,
    c(4, 5)], na.rm = T)


### EFWZ
df.efwz.p1 = aggregate(efwz ~ hhid + lpg +
    strata, data = df, FUN = mean, subset = visit ==
    "P1")
names(df.efwz.p1)[4] = "efwz1"
df.efwz.p2 = aggregate(efwz ~ hhid + lpg +
    strata, data = df, FUN = mean, subset = visit ==
    "P2")
names(df.efwz.p2)[4] = "efwz2"

df.efwz.birth = aggregate(efwz ~ hhid + lpg +
    strata, data = df, FUN = mean, subset = visit ==
    "Birth")
names(df.efwz.birth)[4] = "efwzbirth"

df.efwz.p1p2 = full_join(df.efwz.p1, df.efwz.p2[,
```

```
    c("hhid", "lpg", "efwz2")], by = c("hhid",
    "lpg"))
df.efwz.p1p2 = full_join(df.efwz.p1p2, df.efwz.birth[,
    c("hhid", "lpg", "efwzbirth")], by = c("hhid",
    "lpg"))

df.efwz.p1p2$avgefwz = rowMeans(df.efwz.p1p2[,
    c(4, 5, 6)], na.rm = T)

### Check
mean(df.flz$flz[df.flz$lpg == 1], na.rm = TRUE)
```

```
[1] 0.4375507
```

```
mean(df.flz.p1p2$avgflz[df.flz.p1p2$lpg ==
    1], na.rm = TRUE)
```

```
[1] 0.4375507
```

```
mean(df.flz$flz[df.flz$lpg == 0], na.rm = TRUE)
```

```
[1] 0.448439
```

```
mean(df.flz.p1p2$avgflz[df.flz.p1p2$lpg ==
    0], na.rm = TRUE)
```

```
[1] 0.448439
```

```
tab4["P1", "hcz.int"] <- mean(df.hcz.p1p2$hcz1[df.hcz.p1p2$lpg ==
    1], na.rm = TRUE)
tab4["P2", "hcz.int"] <- mean(df.hcz.p1p2$hcz2[df.hcz.p1p2$lpg ==
    1], na.rm = TRUE)
tab4["All", "hcz.int"] <- mean(df.hcz.p1p2$avghcz[df.hcz.p1p2$lpg ==
    1], na.rm = TRUE)

tab4["P1", "hcz.cont"] <- mean(df.hcz.p1p2$hcz1[df.hcz.p1p2$lpg ==
```

```
    0], na.rm = TRUE)
tab4["P2", "hcz.cont"] <- mean(df.hcz.p1p2$hcz2[df.hcz.p1p2$lpg ==
    0], na.rm = TRUE)
tab4["All", "hcz.cont"] <- mean(df.hcz.p1p2$avghcz[df.hcz.p1p2$lpg ==
    0], na.rm = TRUE)

tab4["P1", "acz.int"] <- mean(df.acz.p1p2$acz1[df.acz.p1p2$lpg ==
    1], na.rm = TRUE)
tab4["P2", "acz.int"] <- mean(df.acz.p1p2$acz2[df.acz.p1p2$lpg ==
    1], na.rm = TRUE)
tab4["All", "acz.int"] <- mean(df.acz.p1p2$avgacz[df.acz.p1p2$lpg ==
    1], na.rm = TRUE)

tab4["P1", "acz.cont"] <- mean(df.acz.p1p2$acz1[df.acz.p1p2$lpg ==
    0], na.rm = TRUE)
tab4["P2", "acz.cont"] <- mean(df.acz.p1p2$acz2[df.acz.p1p2$lpg ==
    0], na.rm = TRUE)
tab4["All", "acz.cont"] <- mean(df.acz.p1p2$avgacz[df.acz.p1p2$lpg ==
    0], na.rm = TRUE)

tab4["P1", "flz.int"] <- mean(df.flz.p1p2$flz1[df.flz.p1p2$lpg ==
    1], na.rm = TRUE)
tab4["P2", "flz.int"] <- mean(df.flz.p1p2$flz2[df.flz.p1p2$lpg ==
    1], na.rm = TRUE)
tab4["All", "flz.int"] <- mean(df.flz.p1p2$avgflz[df.flz.p1p2$lpg ==
    1], na.rm = TRUE)

tab4["P1", "flz.cont"] <- mean(df.flz.p1p2$flz1[df.flz.p1p2$lpg ==
    0], na.rm = TRUE)
tab4["P2", "flz.cont"] <- mean(df.flz.p1p2$flz2[df.flz.p1p2$lpg ==
    0], na.rm = TRUE)
tab4["All", "flz.cont"] <- mean(df.flz.p1p2$avgflz[df.flz.p1p2$lpg ==
    0], na.rm = TRUE)

tab4["P1", "efwz.int"] <- mean(df.efwz.p1p2$efwz1[df.efwz.p1p2$lpg ==
    1], na.rm = TRUE)
tab4["P2", "efwz.int"] <- mean(df.efwz.p1p2$efwz2[df.efwz.p1p2$lpg ==
    1], na.rm = TRUE)
tab4["All", "efwz.int"] <- mean(df.efwz.p1p2$avgefwz[df.efwz.p1p2$lpg ==
    1], na.rm = TRUE)
tab4["Birth", "efwz.int"] <- mean(df.efwz.p1p2$efwzbirth[df.efwz.p1p2$lpg ==
```

```
    1], na.rm = TRUE)

tab4["P1", "efwz.cont"] <- mean(df.efwz.p1p2$efwz1[df.efwz.p1p2$lpg ==
    0], na.rm = TRUE)
tab4["P2", "efwz.cont"] <- mean(df.efwz.p1p2$efwz2[df.efwz.p1p2$lpg ==
    0], na.rm = TRUE)
tab4["All", "efwz.cont"] <- mean(df.efwz.p1p2$avgefwz[df.efwz.p1p2$lpg ==
    0], na.rm = TRUE)
tab4["Birth", "efwz.cont"] <- mean(df.efwz.p1p2$efwzbirth[df.efwz.p1p2$lpg ==
    0], na.rm = TRUE)

for (k in c("hcz.int", "hcz.cont", "acz.int",
    "acz.cont", "flz.int", "flz.cont", "efwz.int",
    "efwz.cont")) {

    tab4[[k]] <- ifelse(tab4[[k]] == " NA",
        NA, as.numeric(tab4[[k]]))
    tab4[[k]] <- sprintf("%3.2f", round(as.numeric(tab4[[k]]),
        2))
    tab4[[k]] <- ifelse(tab4[[k]] == " NA",
        NA, tab4[[k]])
}

#
model.flz.overall = lm(flz ~ lpg + factor(strata),
    data = df.flz)
summary(model.flz.overall)
```

```
Call:
lm(formula = flz ~ lpg + factor(strata), data = df.flz)

Residuals:
    Min      1Q  Median      3Q     Max
-6.4270 -0.6349  0.0163  0.6628  5.7702

Coefficients:
                 Estimate Std. Error t value Pr(>|t|)
(Intercept)       0.49152    0.04136  11.885  < 2e-16 ***
lpg              -0.01337    0.03890  -0.344 0.731017
factor(strata)2  -0.11208    0.11632  -0.964 0.335363
factor(strata)3  -0.16136    0.12531  -1.288 0.197960
```

```
factor(strata)4  -0.06799    0.13312  -0.511 0.609549
factor(strata)5  -0.29486    0.11885  -2.481 0.013165 *
factor(strata)6  -0.06470    0.05213  -1.241 0.214704
factor(strata)7   0.58279    0.07152   8.148 5.52e-16 ***
factor(strata)8  -0.28871    0.06804  -4.243 2.28e-05 ***
factor(strata)9  -0.36481    0.09667  -3.774 0.000164 ***
factor(strata)10 -0.19781    0.08180  -2.418 0.015663 *
---
Signif. codes:  0 '***' 0.001 '**' 0.01 '*' 0.05 '.' 0.1 ' ' 1

Residual standard error: 1.024 on 2761 degrees of freedom
Multiple R-squared:  0.05082,   Adjusted R-squared:  0.04738
F-statistic: 14.78 on 10 and 2761 DF,  p-value: < 2.2e-16
```

```
lincom(model.flz.overall, c("lpg"), level = 0.9875)
```

```
       Estimate  0.625 %   99.375 % Df Sum of Sq
lpg -0.01337354 -0.11053 0.0837829  1 0.1238915
```

```
tab4 <- fun.tab4(model.flz.overall, "All",
    "fl")

model.acz.overall = lm(acz ~ lpg + factor(strata),
    data = df.acz)
summary(model.acz.overall)
```

```
Call:
lm(formula = acz ~ lpg + factor(strata), data = df.acz)

Residuals:
    Min      1Q  Median      3Q     Max
-5.8431 -0.6286 -0.0092  0.6342  3.7871

Coefficients:
                  Estimate Std. Error t value Pr(>|t|)
(Intercept)       0.565789   0.039587  14.292  < 2e-16 ***
lpg              -0.000329   0.037231  -0.009  0.99295
factor(strata)2   0.512904   0.111323   4.607 4.26e-06 ***
factor(strata)3   0.224373   0.119918   1.871  0.06144 .
```

```
factor(strata)4   0.639394   0.127394   5.019 5.52e-07 ***
factor(strata)5   0.359487   0.113744   3.160  0.00159 **
factor(strata)6  -0.486296   0.049903  -9.745  < 2e-16 ***
factor(strata)7  -0.829531   0.068454 -12.118  < 2e-16 ***
factor(strata)8   0.351523   0.065126   5.398 7.33e-08 ***
factor(strata)9   0.426378   0.092518   4.609 4.24e-06 ***
factor(strata)10 -1.057460   0.078290 -13.507  < 2e-16 ***
---
Signif. codes:  0 '***' 0.001 '**' 0.01 '*' 0.05 '.' 0.1 ' ' 1

Residual standard error: 0.9797 on 2760 degrees of freedom
Multiple R-squared:  0.1905,    Adjusted R-squared:  0.1875
F-statistic: 64.94 on 10 and 2760 DF,  p-value: < 2.2e-16
```

```
lincom(model.acz.overall, c("lpg"), level = 0.9875)
```

```
         Estimate     0.625 %   99.375 % Df    Sum of Sq
lpg -0.0003289599 -0.09332004 0.09266212  1 7.493299e-05
```

```
tab4 <- fun.tab4(model.acz.overall, "All",
    "ac")

model.hcz.overall = lm(hcz ~ lpg + factor(strata),
    data = df.hcz)
summary(model.hcz.overall)
```

```
Call:
lm(formula = hcz ~ lpg + factor(strata), data = df.hcz)

Residuals:
    Min      1Q  Median      3Q     Max
-9.1210 -0.6429 -0.0188  0.6203  4.0448

Coefficients:
                 Estimate Std. Error t value Pr(>|t|)
(Intercept)       0.86004    0.04079  21.085  < 2e-16 ***
lpg              -0.07761    0.03836  -2.023   0.0432 *
factor(strata)2  -0.45891    0.11472  -4.000 6.50e-05 ***
factor(strata)3  -0.53152    0.12358  -4.301 1.76e-05 ***
```

```
factor(strata)4  -0.07216    0.13129  -0.550   0.5826
factor(strata)5  -0.76587    0.11722  -6.534 7.62e-11 ***
factor(strata)6  -0.77271    0.05141 -15.029  < 2e-16 ***
factor(strata)7  -0.40545    0.07054  -5.748 1.00e-08 ***
factor(strata)8  -0.54675    0.06711  -8.147 5.57e-16 ***
factor(strata)9  -0.48006    0.09534  -5.035 5.08e-07 ***
factor(strata)10 -1.20527    0.08068 -14.939  < 2e-16 ***
---
Signif. codes:  0 '***' 0.001 '**' 0.01 '*' 0.05 '.' 0.1 ' ' 1

Residual standard error: 1.01 on 2761 degrees of freedom
Multiple R-squared:  0.1172,    Adjusted R-squared:  0.114
F-statistic: 36.66 on 10 and 2761 DF,  p-value: < 2.2e-16
```

```r
lincom(model.hcz.overall, c("lpg"), level = 0.9875)
```

```
       Estimate    0.625 %   99.375 % Df Sum of Sq
lpg -0.07760579 -0.1734265 0.01821495  1  4.171931
```

```r
tab4 <- fun.tab4(model.hcz.overall, "All",
    "hc")

model.efwz.overall = lm(efwz ~ lpg + factor(strata),
    data = df.efwz)
summary(model.efwz.overall)
```

```
Call:
lm(formula = efwz ~ lpg + factor(strata), data = df.efwz)

Residuals:
     Min       1Q   Median       3Q      Max
-10.6007  -0.5786   0.0042   0.5882   4.3418

Coefficients:
                 Estimate Std. Error t value Pr(>|t|)
(Intercept)       0.21546    0.03718   5.794 7.55e-09 ***
lpg              -0.01301    0.03358  -0.387  0.69860
factor(strata)2   0.27229    0.10526   2.587  0.00973 **
factor(strata)3   0.12320    0.11328   1.088  0.27686
```

```
factor(strata)4   0.54187    0.12109   4.475 7.92e-06 ***
factor(strata)5   0.13535    0.10811   1.252  0.21071
factor(strata)6  -0.39728    0.04732  -8.396  < 2e-16 ***
factor(strata)7  -0.83550    0.05789 -14.433  < 2e-16 ***
factor(strata)8   0.19592    0.06181   3.170  0.00154 **
factor(strata)9   0.23730    0.08793   2.699  0.00700 **
factor(strata)10 -1.50805    0.05814 -25.938  < 2e-16 ***
---
Signif. codes:  0 '***' 0.001 '**' 0.01 '*' 0.05 '.' 0.1 ' ' 1

Residual standard error: 0.9314 on 3066 degrees of freedom
Multiple R-squared:  0.2655,    Adjusted R-squared:  0.2631
F-statistic: 110.8 on 10 and 3066 DF,  p-value: < 2.2e-16
```

```
lincom(model.efwz.overall, c("lpg"), level = 0.9875)
```

```
       Estimate     0.625 %   99.375 % Df Sum of Sq
lpg -0.01300525 -0.09688853 0.07087803  1 0.1300833
```

```
tab4 <- fun.tab4(model.efwz.overall, "All",
    "efw")

(mean.hc.int <- round(mean(df.hcz$hcz[df.hcz$lpg ==
    1], na.rm = TRUE), 2))
```

```
[1] 0.3
```

```
(mean.hc.cont <- round(mean(df.hcz$hcz[df.hcz$lpg ==
    0], na.rm = TRUE), 2))
```

```
[1] 0.39
```

```
td.hc <- tidy(model.hcz.overall)
p_hc <- round(td.hc$p.value[td.hc$term ==
    "lpg"], 2)

(mean.ac.int <- round(mean(df.acz$acz[df.acz$lpg ==
```

```r
    1], na.rm = TRUE), 2))
```

```
[1] 0.38
```

```r
(mean.ac.cont <- round(mean(df.acz$acz[df.acz$lpg ==
    0], na.rm = TRUE), 2))
```

```
[1] 0.39
```

```r
td.ac <- tidy(model.acz.overall)
p_ac <- round(td.ac$p.value[td.ac$term ==
    "lpg"], 2)

(mean.fl.int <- round(mean(df.flz$flz[df.flz$lpg ==
    1], na.rm = TRUE), 2))
```

```
[1] 0.44
```

```r
(mean.fl.cont <- round(mean(df.flz$flz[df.flz$lpg ==
    0], na.rm = TRUE), 2))
```

```
[1] 0.45
```

```r
td.fl <- tidy(model.flz.overall)
p_fl <- round(td.fl$p.value[td.fl$term ==
    "lpg"], 2)

(mean.efw.int <- round(mean(df.efwz$efwz[df.efwz$lpg ==
    1], na.rm = TRUE), 2))
```

```
[1] -0.13
```

```r
(mean.efw.cont <- round(mean(df.efwz$efwz[df.efwz$lpg ==
    0], na.rm = TRUE), 2))
```

```
[1] -0.12
```

```r
td.efw <- tidy(model.efwz.overall)
p_efw <- round(td.efw$p.value[td.efw$term ==
    "lpg"], 2)

tab4$fl.lab <- paste0(sprintf("%3.2f", round(tab4$fl,
    2)), " (", sprintf("%3.2f", round(tab4$fl.lb,
    2)), " to ", sprintf("%3.2f", round(tab4$fl.ub,
    2)), ")")

tab4$ac.lab <- paste0(sprintf("%3.2f", round(tab4$ac,
    2)), " (", sprintf("%3.2f", round(tab4$ac.lb,
    2)), " to ", sprintf("%3.2f", round(tab4$ac.ub,
    2)), ")")

tab4$hc.lab <- paste0(sprintf("%3.2f", round(tab4$hc,
    2)), " (", sprintf("%3.2f", round(tab4$hc.lb,
    2)), " to ", sprintf("%3.2f", round(tab4$hc.ub,
    2)), ")")

tab4$efw.lab <- paste0(sprintf("%3.2f", round(tab4$efw,
    2)), " (", sprintf("%3.2f", round(tab4$efw.lb,
    2)), " to ", sprintf("%3.2f", round(tab4$efw.ub,
    2)), ")")

tab4["Birth", c("fl.lab", "ac.lab", "hc.lab")] <- NA


### Figure-4

tab4$visits <- factor(tab4$visits, levels = c("All",
    "Birth", "P2", "P1"))
```

FLZ 25-weeks

```r
tab5 <- data.frame(matrix(ncol = 14, nrow = 4))
colnames(tab5) <- c("weeks", "groups", "hc",
    "hc.lb", "hc.ub", "ac", "ac.lb", "ac.ub",
    "fl", "fl.lb", "fl.ub", "efw", "efw.lb",
    "efw.ub")
```

```r
tab5$weeks <- c(25, 25, 33, 33)
tab5$groups <- c(1, 2, 1, 2)
rownames(tab5) <- c("25_1", "25_2", "33_1",
    "33_2")

### flz
model.galpg.flz = gamm4(flz ~ t2(ga, by = lpg3) +
    factor(strata), data = df3[df3$visit !=
    "Birth", ], random = ~(1 + ga | hhid))
summary(model.galpg.flz$gam)
```

```
Family: gaussian
Link function: identity

Formula:
flz ~ t2(ga, by = lpg3) + factor(strata)

Parametric coefficients:
                 Estimate Std. Error t value Pr(>|t|)
(Intercept)       0.43204    0.03456  12.502  < 2e-16 ***
factor(strata)2  -0.06087    0.10818  -0.563 0.573655
factor(strata)3  -0.21254    0.11614  -1.830 0.067303 .
factor(strata)4  -0.08882    0.12506  -0.710 0.477601
factor(strata)5  -0.31038    0.11153  -2.783 0.005402 **
factor(strata)6  -0.06343    0.04879  -1.300 0.193695
factor(strata)7   0.47610    0.06430   7.405 1.48e-13 ***
factor(strata)8  -0.26881    0.06399  -4.201 2.69e-05 ***
factor(strata)9  -0.34199    0.09060  -3.775 0.000162 ***
factor(strata)10 -0.09591    0.07258  -1.321 0.186398
---
Signif. codes:  0 '***' 0.001 '**' 0.01 '*' 0.05 '.' 0.1 ' ' 1

Approximate significance of smooth terms:
               edf Ref.df      F  p-value
t2(ga):lpg30 3.605  3.605  9.004 6.77e-05 ***
t2(ga):lpg31 3.000  3.000  3.657  0.01192 *
t2(ga):lpg32 3.427  3.427 12.187  0.00289 **
---
Signif. codes:  0 '***' 0.001 '**' 0.01 '*' 0.05 '.' 0.1 ' ' 1

R-sq.(adj) =  0.0358
```

```
lmer.REML =  18635  Scale est. = 0.41899   n = 6613
```

```r
### estimating differences
predict(model.galpg.flz$gam, newdata = list(ga = 25,
    lpg3 = 2, hhid = 13001, strata = 1)) -
    predict(model.galpg.flz$gam, newdata = list(ga = 25,
        lpg3 = 0, hhid = 13001, strata = 1))
```

```
          1
-0.05488864
```

```r
predict(model.galpg.flz$gam, newdata = list(ga = 25,
    lpg3 = 1, hhid = 13001, strata = 1)) -
    predict(model.galpg.flz$gam, newdata = list(ga = 25,
        lpg3 = 0, hhid = 13001, strata = 1))
```

```
          1
-0.01831529
```

```r
### diff and 95% CI
beta = coef(model.galpg.flz$gam)
V = vcov(model.galpg.flz$gam)

## 25 wk 2 vs 0
df_pred = data.frame(lpg3 = c(2, 0), ga = 25,
    hhid = 13001, strata = 1)
phi_mat <- predict(model.galpg.flz$gam, type = "lpmatrix",
    newdata = df_pred)
dX <- beta[19:22] %*% phi_mat[1, 19:22] -
    beta[11:14] %*% phi_mat[2, 11:14]

vc <- V[c(11:14, 19:22), c(11:14, 19:22)]
a <- phi_mat[2, c(11:14, 19:22)] - phi_mat[1,
    c(11:14, 19:22)]
vX <- (t(a) %*% vc %*% a)

c(dX, dX - qnorm(0.99375) * sqrt(vX), dX +
    qnorm(0.99375) * sqrt(vX))
```

```
[1] -0.05488864 -0.12581699  0.01603971
```

```
tab5["25_2", c("fl", "fl.lb", "fl.ub")] <- c(dX,
    dX - qnorm(0.99375) * sqrt(vX), dX +
        qnorm(0.99375) * sqrt(vX))

# 1 vs. 0
df_pred = data.frame(lpg3 = c(1, 0), ga = 25,
    hhid = 13001, strata = 1)
phi_mat <- predict(model.galpg.flz$gam, type = "lpmatrix",
    newdata = df_pred)
dX <- beta[15:18] %*% phi_mat[1, 15:18] -
    beta[11:14] %*% phi_mat[2, 11:14]

vc <- V[c(11:18), c(11:18)]
a <- phi_mat[2, c(11:18)] - phi_mat[1, c(11:18)]
vX <- (t(a) %*% vc %*% a)

c(dX, dX - qnorm(0.99375) * sqrt(vX), dX +
    qnorm(0.99375) * sqrt(vX))
```

```
[1] -0.01831529 -0.09002309  0.05339252
```

```
tab5["25_1", c("fl", "fl.lb", "fl.ub")] <- c(dX,
    dX - qnorm(0.99375) * sqrt(vX), dX +
        qnorm(0.99375) * sqrt(vX))
```

FLZ 33-weeks

```
# 33 wk

predict(model.galpg.flz$gam, newdata = list(ga = 33,
    lpg3 = 2, hhid = 13001, strata = 1)) -
    predict(model.galpg.flz$gam, newdata = list(ga = 33,
        lpg3 = 0, hhid = 13001, strata = 1))
```

```
         1
0.05536268
```

```r
predict(model.galpg.flz$gam, newdata = list(ga = 33,
    lpg3 = 1, hhid = 13001, strata = 1)) -
    predict(model.galpg.flz$gam, newdata = list(ga = 33,
        lpg3 = 0, hhid = 13001, strata = 1))
```

```
         1
-0.0178795
```

```r
### diff and 95% CI
beta = coef(model.galpg.flz$gam)
V = vcov(model.galpg.flz$gam)

# 2 vs 0
df_pred = data.frame(lpg3 = c(2, 0), ga = 33,
    hhid = 13001, strata = 1)
phi_mat <- predict(model.galpg.flz$gam, type = "lpmatrix",
    newdata = df_pred)
dX <- beta[19:22] %*% phi_mat[1, 19:22] -
    beta[11:14] %*% phi_mat[2, 11:14]

vc <- V[c(11:14, 19:22), c(11:14, 19:22)]
a <- phi_mat[2, c(11:14, 19:22)] - phi_mat[1,
    c(11:14, 19:22)]
vX <- (t(a) %*% vc %*% a)

c(dX, dX - qnorm(0.99375) * sqrt(vX), dX +
    qnorm(0.99375) * sqrt(vX))
```

```
[1]  0.05536268 -0.02332063  0.13404598
```

```r
tab5["33_2", c("fl", "fl.lb", "fl.ub")] <- c(dX,
    dX - qnorm(0.99375) * sqrt(vX), dX +
        qnorm(0.99375) * sqrt(vX))


# 1 vs. 0
df_pred = data.frame(lpg3 = c(1, 0), ga = 33,
    hhid = 13001, strata = 1)
phi_mat <- predict(model.galpg.flz$gam, type = "lpmatrix",
```

```
    newdata = df_pred)
dX <- beta[15:18] %*% phi_mat[1, 15:18] -
    beta[11:14] %*% phi_mat[2, 11:14]

vc <- V[c(11:18), c(11:18)]
a <- phi_mat[2, c(11:18)] - phi_mat[1, c(11:18)]
vX <- (t(a) %*% vc %*% a)

c(dX, dX - qnorm(0.99375) * sqrt(vX), dX +
    qnorm(0.99375) * sqrt(vX))
```

```
[1] -0.01787950 -0.10446625  0.06870726
```

```
tab5["33_1", c("fl", "fl.lb", "fl.ub")] <- c(dX,
    dX - qnorm(0.99375) * sqrt(vX), dX +
        qnorm(0.99375) * sqrt(vX))
```

HCZ 25-weeks

```
### hcz
model.galpg.hcz = gamm4(hcz ~ t2(ga, by = lpg3) +
    factor(strata), data = df3[df3$visit !=
    "Birth", ], random = ~(1 + ga | hhid))

summary(model.galpg.hcz$gam)
```

```
Family: gaussian
Link function: identity

Formula:
hcz ~ t2(ga, by = lpg3) + factor(strata)

Parametric coefficients:
                 Estimate Std. Error t value Pr(>|t|)
(Intercept)       0.78532    0.03378  23.245  < 2e-16 ***
factor(strata)2  -0.38021    0.10617  -3.581 0.000345 ***
factor(strata)3  -0.47918    0.11388  -4.208 2.61e-05 ***
factor(strata)4  -0.07354    0.12295  -0.598 0.549786
```

```
factor(strata)5  -0.67793    0.10948  -6.192 6.29e-10 ***
factor(strata)6  -0.69197    0.04773 -14.497  < 2e-16 ***
factor(strata)7  -0.34295    0.06288  -5.454 5.11e-08 ***
factor(strata)8  -0.46222    0.06282  -7.357 2.11e-13 ***
factor(strata)9  -0.45143    0.08890  -5.078 3.92e-07 ***
factor(strata)10 -1.05014    0.07140 -14.707  < 2e-16 ***
---
Signif. codes:  0 '***' 0.001 '**' 0.01 '*' 0.05 '.' 0.1 ' ' 1

Approximate significance of smooth terms:
               edf Ref.df     F  p-value    
t2(ga):lpg30 3.800  3.800 22.54  < 2e-16 ***
t2(ga):lpg31 3.410  3.410 13.30  < 2e-16 ***
t2(ga):lpg32 3.686  3.686  6.38 0.000121 ***
---
Signif. codes:  0 '***' 0.001 '**' 0.01 '*' 0.05 '.' 0.1 ' ' 1

R-sq.(adj) =  0.0906   
lmer.REML =  18603  Scale est. = 0.37817   n = 6613
```

```r
predict(model.galpg.hcz$gam, newdata = list(ga = 25,
    lpg3 = 2, hhid = 13001, strata = 1)) -
    predict(model.galpg.hcz$gam, newdata = list(ga = 25,
        lpg3 = 0, hhid = 13001, strata = 1))
```

```
          1 
-0.09484861 
```

```r
predict(model.galpg.hcz$gam, newdata = list(ga = 25,
    lpg3 = 1, hhid = 13001, strata = 1)) -
    predict(model.galpg.hcz$gam, newdata = list(ga = 25,
        lpg3 = 0, hhid = 13001, strata = 1))
```

```
          1 
-0.03626975 
```

```r
predict(model.galpg.hcz$gam, newdata = list(ga = 33,
    lpg3 = 2, hhid = 13001, strata = 1)) -
    predict(model.galpg.hcz$gam, newdata = list(ga = 33,
```

```r
        lpg3 = 0, hhid = 13001, strata = 1))
```

```
         1
0.06110174
```

```r
predict(model.galpg.hcz$gam, newdata = list(ga = 33,
    lpg3 = 1, hhid = 13001, strata = 1)) -
    predict(model.galpg.hcz$gam, newdata = list(ga = 33,
        lpg3 = 0, hhid = 13001, strata = 1))
```

```
          1
-0.04329499
```

```r
### diff and 95% CI
beta = coef(model.galpg.hcz$gam)
V = vcov(model.galpg.hcz$gam)

## 25 wk 2 vs 0
df_pred = data.frame(lpg3 = c(2, 0), ga = 25,
    hhid = 13001, strata = 1)
phi_mat <- predict(model.galpg.hcz$gam, type = "lpmatrix",
    newdata = df_pred)
dX <- beta[19:22] %*% phi_mat[1, 19:22] -
    beta[11:14] %*% phi_mat[2, 11:14]

vc <- V[c(11:14, 19:22), c(11:14, 19:22)]
a <- phi_mat[2, c(11:14, 19:22)] - phi_mat[1,
    c(11:14, 19:22)]
vX <- (t(a) %*% vc %*% a)

c(dX, dX - qnorm(0.99375) * sqrt(vX), dX +
    qnorm(0.99375) * sqrt(vX))
```

```
[1] -0.09484861 -0.16493933 -0.02475790
```

```r
tab5["25_2", c("hc", "hc.lb", "hc.ub")] <- c(dX,
    dX - qnorm(0.99375) * sqrt(vX), dX +
        qnorm(0.99375) * sqrt(vX))
```

```r
# 1 vs. 0
df_pred = data.frame(lpg3 = c(1, 0), ga = 25,
    hhid = 13001, strata = 1)
phi_mat <- predict(model.galpg.hcz$gam, type = "lpmatrix",
    newdata = df_pred)
dX <- beta[15:18] %*% phi_mat[1, 15:18] -
    beta[11:14] %*% phi_mat[2, 11:14]

vc <- V[c(11:18), c(11:18)]
a <- phi_mat[2, c(11:18)] - phi_mat[1, c(11:18)]
vX <- (t(a) %*% vc %*% a)

c(dX, dX - qnorm(0.99375) * sqrt(vX), dX +
    qnorm(0.99375) * sqrt(vX))
```

```
[1] -0.03626975 -0.10732003  0.03478053
```

```r
tab5["25_1", c("hc", "hc.lb", "hc.ub")] <- c(dX,
    dX - qnorm(0.99375) * sqrt(vX), dX +
        qnorm(0.99375) * sqrt(vX))
```

HCZ 33-weeks

```r
# 33 wk

predict(model.galpg.hcz$gam, newdata = list(ga = 33,
    lpg3 = 2, hhid = 13001, strata = 1)) -
    predict(model.galpg.hcz$gam, newdata = list(ga = 33,
        lpg3 = 0, hhid = 13001, strata = 1))
```

```
         1
0.06110174
```

```r
predict(model.galpg.hcz$gam, newdata = list(ga = 33,
    lpg3 = 1, hhid = 13001, strata = 1)) -
    predict(model.galpg.hcz$gam, newdata = list(ga = 33,
        lpg3 = 0, hhid = 13001, strata = 1))
```

```
          1
-0.04329499
```

```r
### diff and 95% CI
beta = coef(model.galpg.hcz$gam)
V = vcov(model.galpg.hcz$gam)

# 2 vs 0
df_pred = data.frame(lpg3 = c(2, 0), ga = 33,
    hhid = 13001, strata = 1)
phi_mat <- predict(model.galpg.hcz$gam, type = "lpmatrix",
    newdata = df_pred)
dX <- beta[19:22] %*% phi_mat[1, 19:22] -
    beta[11:14] %*% phi_mat[2, 11:14]

vc <- V[c(11:14, 19:22), c(11:14, 19:22)]
a <- phi_mat[2, c(11:14, 19:22)] - phi_mat[1,
    c(11:14, 19:22)]
vX <- (t(a) %*% vc %*% a)

c(dX, dX - qnorm(0.99375) * sqrt(vX), dX +
    qnorm(0.99375) * sqrt(vX))
```

```
[1]  0.06110174 -0.02198294  0.14418642
```

```r
tab5["33_2", c("hc", "hc.lb", "hc.ub")] <- c(dX,
    dX - qnorm(0.99375) * sqrt(vX), dX +
        qnorm(0.99375) * sqrt(vX))



# 1 vs. 0
df_pred = data.frame(lpg3 = c(1, 0), ga = 33,
    hhid = 13001, strata = 1)
phi_mat <- predict(model.galpg.hcz$gam, type = "lpmatrix",
    newdata = df_pred)
dX <- beta[15:18] %*% phi_mat[1, 15:18] -
    beta[11:14] %*% phi_mat[2, 11:14]

vc <- V[c(11:18), c(11:18)]
a <- phi_mat[2, c(11:18)] - phi_mat[1, c(11:18)]
```

```r
vX <- (t(a) %*% vc %*% a)

c(dX, dX - qnorm(0.99375) * sqrt(vX), dX +
    qnorm(0.99375) * sqrt(vX))
```

```
[1] -0.04329499 -0.13432797  0.04773798
```

```r
tab5["33_1", c("hc", "hc.lb", "hc.ub")] <- c(dX,
    dX - qnorm(0.99375) * sqrt(vX), dX +
        qnorm(0.99375) * sqrt(vX))
```

ACZ 25-weeks

```r
### acz
model.galpg.acz = gamm4(acz ~ t2(ga, by = lpg3) +
    factor(strata), data = df3[df3$visit !=
    "Birth", ], random = ~(1 + ga | hhid))

summary(model.galpg.acz$gam)
```

```
Family: gaussian
Link function: identity

Formula:
acz ~ t2(ga, by = lpg3) + factor(strata)

Parametric coefficients:
                  Estimate Std. Error t value Pr(>|t|)
(Intercept)        0.56561    0.03397  16.652  < 2e-16 ***
factor(strata)2    0.56129    0.10458   5.367 8.28e-08 ***
factor(strata)3    0.20453    0.11284   1.813   0.0699 .
factor(strata)4    0.62634    0.12084   5.183 2.24e-07 ***
factor(strata)5    0.35887    0.10805   3.321   0.0009 ***
factor(strata)6   -0.49596    0.04750 -10.440  < 2e-16 ***
factor(strata)7   -0.82240    0.06408 -12.834  < 2e-16 ***
factor(strata)8    0.38953    0.06212   6.270 3.83e-10 ***
factor(strata)9    0.43718    0.08767   4.987 6.30e-07 ***
factor(strata)10  -1.00476    0.07235 -13.887  < 2e-16 ***
```

```
---
Signif. codes:  0 '***' 0.001 '**' 0.01 '*' 0.05 '.' 0.1 ' ' 1

Approximate significance of smooth terms:
                edf Ref.df     F p-value
t2(ga):lpg30 3.584  3.584 14.79  <2e-16 ***
t2(ga):lpg31 3.418  3.418 12.92  <2e-16 ***
t2(ga):lpg32 2.343  2.343  1.92   0.085 .
---
Signif. codes:  0 '***' 0.001 '**' 0.01 '*' 0.05 '.' 0.1 ' ' 1

R-sq.(adj) =  0.149
lmer.REML =  19015  Scale est. = 0.45227   n = 6613
```

```r
predict(model.galpg.acz$gam, newdata = list(ga = 25,
    lpg3 = 2, hhid = 13001, strata = 1)) -
    predict(model.galpg.acz$gam, newdata = list(ga = 25,
        lpg3 = 0, hhid = 13001, strata = 1))
```

```
          1
0.006060866
```

```r
predict(model.galpg.acz$gam, newdata = list(ga = 25,
    lpg3 = 1, hhid = 13001, strata = 1)) -
    predict(model.galpg.acz$gam, newdata = list(ga = 25,
        lpg3 = 0, hhid = 13001, strata = 1))
```

```
         1
0.03043939
```

```r
predict(model.galpg.acz$gam, newdata = list(ga = 33,
    lpg3 = 2, hhid = 13001, strata = 1)) -
    predict(model.galpg.acz$gam, newdata = list(ga = 33,
        lpg3 = 0, hhid = 13001, strata = 1))
```

```
         1
0.01214357
```

```r
predict(model.galpg.acz$gam, newdata = list(ga = 33,
    lpg3 = 1, hhid = 13001, strata = 1)) -
    predict(model.galpg.acz$gam, newdata = list(ga = 33,
        lpg3 = 0, hhid = 13001, strata = 1))
```

```
        1
0.0297738
```

```r
### diff and 95% CI
beta = coef(model.galpg.acz$gam)
V = vcov(model.galpg.acz$gam)

## 25 wk 2 vs 0
df_pred = data.frame(lpg3 = c(2, 0), ga = 25,
    hhid = 13001, strata = 1)
phi_mat <- predict(model.galpg.acz$gam, type = "lpmatrix",
    newdata = df_pred)
dX <- beta[19:22] %*% phi_mat[1, 19:22] -
    beta[11:14] %*% phi_mat[2, 11:14]

vc <- V[c(11:14, 19:22), c(11:14, 19:22)]
a <- phi_mat[2, c(11:14, 19:22)] - phi_mat[1,
    c(11:14, 19:22)]
vX <- (t(a) %*% vc %*% a)

c(dX, dX - qnorm(0.99375) * sqrt(vX), dX +
    qnorm(0.99375) * sqrt(vX))
```

```
[1]  0.006060866 -0.062079869  0.074201601
```

```r
tab5["25_2", c("ac", "ac.lb", "ac.ub")] <- c(dX,
    dX - qnorm(0.99375) * sqrt(vX), dX +
        qnorm(0.99375) * sqrt(vX))


# 1 vs. 0
df_pred = data.frame(lpg3 = c(1, 0), ga = 25,
    hhid = 13001, strata = 1)
phi_mat <- predict(model.galpg.acz$gam, type = "lpmatrix",
```

```
    newdata = df_pred)
dX <- beta[15:18] %*% phi_mat[1, 15:18] -
    beta[11:14] %*% phi_mat[2, 11:14]

vc <- V[c(11:18), c(11:18)]
a <- phi_mat[2, c(11:18)] - phi_mat[1, c(11:18)]
vX <- (t(a) %*% vc %*% a)

c(dX, dX - qnorm(0.99375) * sqrt(vX), dX +
    qnorm(0.99375) * sqrt(vX))
```

```
[1]  0.03043939 -0.04647175  0.10735053
```

```
tab5["25_1", c("ac", "ac.lb", "ac.ub")] <- c(dX,
    dX - qnorm(0.99375) * sqrt(vX), dX +
        qnorm(0.99375) * sqrt(vX))
```

ACZ 33-weeks

```
# 33 wk

predict(model.galpg.acz$gam, newdata = list(ga = 33,
    lpg3 = 2, hhid = 13001, strata = 1)) -
    predict(model.galpg.acz$gam, newdata = list(ga = 33,
        lpg3 = 0, hhid = 13001, strata = 1))
```

```
         1
0.01214357
```

```
predict(model.galpg.acz$gam, newdata = list(ga = 33,
    lpg3 = 1, hhid = 13001, strata = 1)) -
    predict(model.galpg.acz$gam, newdata = list(ga = 33,
        lpg3 = 0, hhid = 13001, strata = 1))
```

```
        1
0.0297738
```

```
### diff and 95% CI
beta = coef(model.galpg.acz$gam)
V = vcov(model.galpg.acz$gam)

# 2 vs 0
df_pred = data.frame(lpg3 = c(2, 0), ga = 33,
    hhid = 13001, strata = 1)
phi_mat <- predict(model.galpg.acz$gam, type = "lpmatrix",
    newdata = df_pred)
dX <- beta[19:22] %*% phi_mat[1, 19:22] -
    beta[11:14] %*% phi_mat[2, 11:14]

vc <- V[c(11:14, 19:22), c(11:14, 19:22)]
a <- phi_mat[2, c(11:14, 19:22)] - phi_mat[1,
    c(11:14, 19:22)]
vX <- (t(a) %*% vc %*% a)

c(dX, dX - qnorm(0.99375) * sqrt(vX), dX +
    qnorm(0.99375) * sqrt(vX))
```

```
[1]  0.01214357 -0.06560574  0.08989287
```

```
tab5["33_2", c("ac", "ac.lb", "ac.ub")] <- c(dX,
    dX - qnorm(0.99375) * sqrt(vX), dX +
        qnorm(0.99375) * sqrt(vX))


# 1 vs. 0
df_pred = data.frame(lpg3 = c(1, 0), ga = 33,
    hhid = 13001, strata = 1)
phi_mat <- predict(model.galpg.acz$gam, type = "lpmatrix",
    newdata = df_pred)
dX <- beta[15:18] %*% phi_mat[1, 15:18] -
    beta[11:14] %*% phi_mat[2, 11:14]

vc <- V[c(11:18), c(11:18)]
a <- phi_mat[2, c(11:18)] - phi_mat[1, c(11:18)]
vX <- (t(a) %*% vc %*% a)

c(dX, dX - qnorm(0.99375) * sqrt(vX), dX +
    qnorm(0.99375) * sqrt(vX))
```

```
[1]  0.02977380 -0.05901273  0.11856033
```

```
tab5["33_1", c("ac", "ac.lb", "ac.ub")] <- c(dX,
    dX - qnorm(0.99375) * sqrt(vX), dX +
        qnorm(0.99375) * sqrt(vX))
```

EFWZ 25-weeks

```
### efwz

model.galpg.efwz = gamm4(efwz ~ t2(ga, by = lpg3) +
    factor(strata), data = df3, random = ~(1 +
    ga | hhid))

summary(model.galpg.efwz$gam)
```

```
Family: gaussian
Link function: identity

Formula:
efwz ~ t2(ga, by = lpg3) + factor(strata)

Parametric coefficients:
                  Estimate Std. Error t value Pr(>|t|)
(Intercept)        0.20211    0.02909   6.948 3.94e-12 ***
factor(strata)2    0.35385    0.09288   3.810 0.000140 ***
factor(strata)3    0.19808    0.09998   1.981 0.047592 *
factor(strata)4    0.54685    0.10841   5.044 4.64e-07 ***
factor(strata)5    0.21794    0.09662   2.256 0.024112 *
factor(strata)6   -0.39752    0.04232  -9.393  < 2e-16 ***
factor(strata)7   -0.56850    0.05122 -11.100  < 2e-16 ***
factor(strata)8    0.29677    0.05569   5.329 1.01e-07 ***
factor(strata)9    0.28560    0.07644   3.736 0.000188 ***
factor(strata)10 -1.08793    0.05955 -18.269  < 2e-16 ***
---
Signif. codes:  0 '***' 0.001 '**' 0.01 '*' 0.05 '.' 0.1 ' ' 1

Approximate significance of smooth terms:
               edf Ref.df     F p-value
```

```
t2(ga):lpg30 3.794  3.794 340.8  <2e-16 ***
t2(ga):lpg31 3.782  3.782 202.8  <2e-16 ***
t2(ga):lpg32 3.677  3.677 178.2  <2e-16 ***
---
Signif. codes:  0 '***' 0.001 '**' 0.01 '*' 0.05 '.' 0.1 ' ' 1

R-sq.(adj) =  0.265
lmer.REML =  25537  Scale est. = 0.35777   n = 9580
```

```r
predict(model.galpg.efwz$gam, newdata = list(ga = 25,
    lpg3 = 2, hhid = 13001, strata = 1)) -
    predict(model.galpg.efwz$gam, newdata = list(ga = 25,
        lpg3 = 0, hhid = 13001, strata = 1))
```

```
          1
-0.05312037
```

```r
predict(model.galpg.efwz$gam, newdata = list(ga = 25,
    lpg3 = 1, hhid = 13001, strata = 1)) -
    predict(model.galpg.efwz$gam, newdata = list(ga = 25,
        lpg3 = 0, hhid = 13001, strata = 1))
```

```
          1
-0.03425069
```

```r
predict(model.galpg.efwz$gam, newdata = list(ga = 33,
    lpg3 = 2, hhid = 13001, strata = 1)) -
    predict(model.galpg.efwz$gam, newdata = list(ga = 33,
        lpg3 = 0, hhid = 13001, strata = 1))
```

```
         1
0.02212593
```

```r
predict(model.galpg.efwz$gam, newdata = list(ga = 33,
    lpg3 = 1, hhid = 13001, strata = 1)) -
    predict(model.galpg.efwz$gam, newdata = list(ga = 33,
        lpg3 = 0, hhid = 13001, strata = 1))
```

```
         1
-0.01011948
```

```
### diff and 95% CI
beta = coef(model.galpg.efwz$gam)
V = vcov(model.galpg.efwz$gam)

## 25 wk 2 vs 0
df_pred = data.frame(lpg3 = c(2, 0), ga = 25,
    hhid = 13001, strata = 1)
phi_mat <- predict(model.galpg.efwz$gam,
    type = "lpmatrix", newdata = df_pred)
dX <- beta[19:22] %*% phi_mat[1, 19:22] -
    beta[11:14] %*% phi_mat[2, 11:14]

vc <- V[c(11:14, 19:22), c(11:14, 19:22)]
a <- phi_mat[2, c(11:14, 19:22)] - phi_mat[1,
    c(11:14, 19:22)]
vX <- (t(a) %*% vc %*% a)

c(dX, dX - qnorm(0.99375) * sqrt(vX), dX +
    qnorm(0.99375) * sqrt(vX))
```

```
[1] -0.05312037 -0.13536305  0.02912231
```

```
tab5["25_2", c("efw", "efw.lb", "efw.ub")] <- c(dX,
    dX - qnorm(0.99375) * sqrt(vX), dX +
        qnorm(0.99375) * sqrt(vX))


# 1 vs. 0
df_pred = data.frame(lpg3 = c(1, 0), ga = 25,
    hhid = 13001, strata = 1)
phi_mat <- predict(model.galpg.efwz$gam,
    type = "lpmatrix", newdata = df_pred)
dX <- beta[15:18] %*% phi_mat[1, 15:18] -
    beta[11:14] %*% phi_mat[2, 11:14]

vc <- V[c(11:18), c(11:18)]
a <- phi_mat[2, c(11:18)] - phi_mat[1, c(11:18)]
vX <- (t(a) %*% vc %*% a)
```

```r
c(dX, dX - qnorm(0.99375) * sqrt(vX), dX +
    qnorm(0.99375) * sqrt(vX))
```

```
[1] -0.03425069 -0.10640203  0.03790066
```

```r
tab5["25_1", c("efw", "efw.lb", "efw.ub")] <- c(dX,
    dX - qnorm(0.99375) * sqrt(vX), dX +
        qnorm(0.99375) * sqrt(vX))

# 0.033351886 0.001182427 0.065521346
```

EFWZ 33-weeks

```r
# 33 wk

predict(model.galpg.efwz$gam, newdata = list(ga = 33,
    lpg3 = 2, hhid = 13001, strata = 1)) -
    predict(model.galpg.efwz$gam, newdata = list(ga = 33,
        lpg3 = 0, hhid = 13001, strata = 1))
```

```
         1
0.02212593
```

```r
predict(model.galpg.efwz$gam, newdata = list(ga = 33,
    lpg3 = 1, hhid = 13001, strata = 1)) -
    predict(model.galpg.efwz$gam, newdata = list(ga = 33,
        lpg3 = 0, hhid = 13001, strata = 1))
```

```
          1
-0.01011948
```

```r
### diff and 95% CI
beta = coef(model.galpg.efwz$gam)
V = vcov(model.galpg.efwz$gam)

# 2 vs 0
df_pred = data.frame(lpg3 = c(2, 0), ga = 33,
```

```r
    hhid = 13001, strata = 1)
phi_mat <- predict(model.galpg.efwz$gam,
    type = "lpmatrix", newdata = df_pred)
dX <- beta[19:22] %*% phi_mat[1, 19:22] -
    beta[11:14] %*% phi_mat[2, 11:14]

vc <- V[c(11:14, 19:22), c(11:14, 19:22)]
a <- phi_mat[2, c(11:14, 19:22)] - phi_mat[1,
    c(11:14, 19:22)]
vX <- (t(a) %*% vc %*% a)

c(dX, dX - qnorm(0.99375) * sqrt(vX), dX +
    qnorm(0.99375) * sqrt(vX))
```

```
[1]  0.02212593 -0.05569554  0.09994741
```

```r
tab5["33_2", c("efw", "efw.lb", "efw.ub")] <- c(dX,
    dX - qnorm(0.99375) * sqrt(vX), dX +
        qnorm(0.99375) * sqrt(vX))


# 1 vs. 0
df_pred = data.frame(lpg3 = c(1, 0), ga = 33,
    hhid = 13001, strata = 1)
phi_mat <- predict(model.galpg.efwz$gam,
    type = "lpmatrix", newdata = df_pred)
dX <- beta[15:18] %*% phi_mat[1, 15:18] -
    beta[11:14] %*% phi_mat[2, 11:14]

vc <- V[c(11:18), c(11:18)]
a <- phi_mat[2, c(11:18)] - phi_mat[1, c(11:18)]
vX <- (t(a) %*% vc %*% a)

c(dX, dX - qnorm(0.99375) * sqrt(vX), dX +
    qnorm(0.99375) * sqrt(vX))
```

```
[1] -0.01011948 -0.08906702  0.06882806
```

```
tab5["33_1", c("efw", "efw.lb", "efw.ub")] <- c(dX,
    dX - qnorm(0.99375) * sqrt(vX), dX +
        qnorm(0.99375) * sqrt(vX))
```

EFWZ 40-weeks

```
df.temp <- data.frame(matrix(ncol = dim(tab5)[2],
    nrow = 2))
colnames(df.temp) <- names(tab5)
df.temp$weeks <- c(40, 40)
df.temp$groups <- 1:2

# 40 wk

predict(model.galpg.efwz$gam, newdata = list(ga = 40,
    lpg3 = 2, hhid = 13001, strata = 1)) -
    predict(model.galpg.efwz$gam, newdata = list(ga = 40,
        lpg3 = 0, hhid = 13001, strata = 1))
```

```
         1
0.02662971
```

```
predict(model.galpg.efwz$gam, newdata = list(ga = 40,
    lpg3 = 1, hhid = 13001, strata = 1)) -
    predict(model.galpg.efwz$gam, newdata = list(ga = 40,
        lpg3 = 0, hhid = 13001, strata = 1))
```

```
         1
0.08171514
```

```
### diff and 95% CI
beta = coef(model.galpg.efwz$gam)
V = vcov(model.galpg.efwz$gam)

# 2 vs 0
df_pred = data.frame(lpg3 = c(2, 0), ga = 40,
    hhid = 13001, strata = 1)
phi_mat <- predict(model.galpg.efwz$gam,
```

```
    type = "lpmatrix", newdata = df_pred)
dX <- beta[19:22] %*% phi_mat[1, 19:22] -
    beta[11:14] %*% phi_mat[2, 11:14]

vc <- V[c(11:14, 19:22), c(11:14, 19:22)]
a <- phi_mat[2, c(11:14, 19:22)] - phi_mat[1,
    c(11:14, 19:22)]
vX <- (t(a) %*% vc %*% a)

c(dX, dX - qnorm(0.99375) * sqrt(vX), dX +
    qnorm(0.99375) * sqrt(vX))
```

```
[1]  0.02662971 -0.06285511  0.11611453
```

```
df.temp[2, c("efw", "efw.lb", "efw.ub")] <- c(dX,
    dX - qnorm(0.99375) * sqrt(vX), dX +
        qnorm(0.99375) * sqrt(vX))


# 1 vs. 0
df_pred = data.frame(lpg3 = c(1, 0), ga = 40,
    hhid = 13001, strata = 1)
phi_mat <- predict(model.galpg.efwz$gam,
    type = "lpmatrix", newdata = df_pred)
dX <- beta[15:18] %*% phi_mat[1, 15:18] -
    beta[11:14] %*% phi_mat[2, 11:14]

vc <- V[c(11:18), c(11:18)]
a <- phi_mat[2, c(11:18)] - phi_mat[1, c(11:18)]
vX <- (t(a) %*% vc %*% a)

c(dX, dX - qnorm(0.99375) * sqrt(vX), dX +
    qnorm(0.99375) * sqrt(vX))
```

```
[1] 0.0817151389 0.0004636175 0.1629666602
```

```
df.temp[1, c("efw", "efw.lb", "efw.ub")] <- c(dX,
    dX - qnorm(0.99375) * sqrt(vX), dX +
        qnorm(0.99375) * sqrt(vX))
```

```
# df3$birth <- ifelse(df3$visit ==
# 'Birth', 1, 0) model = gamm4 ( efwz ~
# t2(ga,by=lpg3) + birth +
# factor(strata) , data=df3,
# random=~(1+ga|hhid))
# predict(model$gam,newdata=list(ga=40,
# birth=1, lpg3=2,hhid=13001,strata=1))
# -
# predict(model$gam,newdata=list(ga=40,
# birth=1, lpg3=0,hhid=13001,strata=1))
# predict(model$gam,newdata=list(ga=40,
# birth=1, lpg3=1,hhid=13001,strata=1))
# -
# predict(model$gam,newdata=list(ga=40,
# birth=1, lpg3=0,hhid=13001,strata=1))
# beta=coef(model$gam)
# V=vcov(model$gam) # 2 vs 0 df_pred =
# data.frame(lpg3=c(2,0),ga=40,
# birth=1,hhid=13001,strata=1) phi_mat
# <- predict(model$gam,
# type='lpmatrix', newdata=df_pred) #dX
# <- beta[20:23]%*%phi_mat[1,20:23] -
# beta[12:15]%*%phi_mat[2,12:15] dX <-
# beta[c(2,20:23)]%*%phi_mat[1,c(2,20:23)]
# -
# beta[c(2,12:15)]%*%phi_mat[2,c(2,12:15)]
# vc <-
# V[c(2,12:15,20:23),c(2,12:15,20:23)]
# a <- phi_mat[2,c(2,12:15,20:23)] -
# phi_mat[1,c(2,12:15,20:23)] vX <-
# (t(a) %*% vc %*% a)

# c(dX, dX - qnorm(0.99375)*sqrt(vX),
# dX + qnorm(0.99375)*sqrt(vX))
# df.temp[2,c('efw', 'efw.lb',
# 'efw.ub')] <- c(dX, dX -
# qnorm(0.99375)*sqrt(vX), dX +
# qnorm(0.99375)*sqrt(vX)) # 1 vs. 0
# df_pred =
# data.frame(lpg3=c(1,0),ga=40,
```

```
# birth=1,hhid=13001,strata=1) phi_mat
# <- predict(model$gam,
# type='lpmatrix', newdata=df_pred) dX
# <- beta[16:19]%*%phi_mat[1,16:19] -
# beta[12:15]%*%phi_mat[2,12:15] vc <-
# V[c(12:15,16:19),c(12:15,16:19)] a <-
# phi_mat[2,c(12:15,16:19)] -
# phi_mat[1,c(12:15,16:19)] vX <- (t(a)
# %*% vc %*% a) c(dX, dX -
# qnorm(0.99375)*sqrt(vX), dX +
# qnorm(0.99375)*sqrt(vX))

# With birth as Random effect 0.1357868
# -0.1650929 0.4366665

# Without birth as Random effect
# 0.142159 -0.158553 0.442871

# -0.1320815 -0.4532441 0.1890811 #
# with -0.1247898 -0.4468833 0.1973038
# # without
```

```r
## New variables GA cutoffs, at 14-17,
## 18-21, 22-25, 26-29, 30-33, 34-41
df$gacut = cut(df$ga, breaks = c(14, 18,
    22, 26, 30, 34, 41), right = F)


### Create new database with paired
### pollution creating average and
### lagged exposure data

pm = aggregate(pm ~ hhid + visit, data = df,
    FUN = mean)
bc = aggregate(bc ~ hhid + visit, data = df,
    FUN = mean)
co = aggregate(co ~ hhid + visit, data = df,
    FUN = mean)

pm$xvisit = 0
pm$xvisit = ifelse(pm$visit == "P1", 1, pm$xvisit)
pm$xvisit = ifelse(pm$visit == "P2", 2, pm$xvisit)
pm$xvisit = ifelse(pm$visit == "Birth", 3,
    pm$xvisit)

pm$id = pm$hhid * 100 + pm$xvisit

bc$xvisit = 0
bc$xvisit = ifelse(bc$visit == "P1", 1, bc$xvisit)
bc$xvisit = ifelse(bc$visit == "P2", 2, bc$xvisit)
bc$xvisit = ifelse(bc$visit == "Birth", 3,
    bc$xvisit)

bc$id = bc$hhid * 100 + bc$xvisit

co$xvisit = 0
co$xvisit = ifelse(co$visit == "P1", 1, co$xvisit)
co$xvisit = ifelse(co$visit == "P2", 2, co$xvisit)
co$xvisit = ifelse(co$visit == "Birth", 3,
    co$xvisit)

co$id = co$hhid * 100 + co$xvisit
```

```
uid = unique(df$hhid)
visit = c("BL", "P1", "P2", "Birth")

hap = expand.grid(hhid = uid, visit = visit)

hap = hap[order(hap$hhid), ]

hap$xvisit = 0
hap$xvisit = ifelse(hap$visit == "P1", 1,
    hap$xvisit)
hap$xvisit = ifelse(hap$visit == "P2", 2,
    hap$xvisit)
hap$xvisit = ifelse(hap$visit == "Birth",
    3, hap$xvisit)

hap$id = hap$hhid * 100 + hap$xvisit

hap = merge(hap, pm[, -c(1, 2, 4)], by = "id",
    all = T)
hap = merge(hap, bc[, -c(1, 2, 4)], by = "id",
    all = T)
hap = merge(hap, co[, -c(1, 2, 4)], by = "id",
    all = T)

for (i in 1:dim(hap)[1]) {
    yid = hap$hhid[i]
    yvisit = hap$xvisit[i]
    if (yvisit == 0) {
        hap$avgpm[i] = ifelse(length(pm$pm[pm$hhid ==
            yid & pm$visit == "BL"]) == 0,
            NA, pm$pm[pm$hhid == yid & pm$visit ==
                "BL"])
    }
    if (yvisit == 1) {
        hap$avgpm[i] = mean(c(pm$pm[pm$hhid ==
            yid & pm$visit == "BL"], pm$pm[pm$hhid ==
            yid & pm$visit == "P1"]), na.rm = T)
    }
    if (yvisit == 2) {
        hap$avgpm[i] = mean(c(pm$pm[pm$hhid ==
            yid & pm$visit == "BL"], pm$pm[pm$hhid ==
```

```
        yid & pm$visit == "P1"], pm$pm[pm$hhid ==
        yid & pm$visit == "P2"]), na.rm = T)
}
if (yvisit == 3) {
    hap$avgpm[i] = mean(c(pm$pm[pm$hhid ==
        yid & pm$visit == "BL"], pm$pm[pm$hhid ==
        yid & pm$visit == "P1"], pm$pm[pm$hhid ==
        yid & pm$visit == "P2"]), na.rm = T)
}

if (yvisit == 0) {
    hap$avgbc[i] = ifelse(length(bc$bc[bc$hhid ==
        yid & bc$visit == "BL"]) == 0,
        NA, bc$bc[bc$hhid == yid & bc$visit ==
            "BL"])
}
if (yvisit == 1) {
    hap$avgbc[i] = mean(c(bc$bc[bc$hhid ==
        yid & bc$visit == "BL"], bc$bc[bc$hhid ==
        yid & bc$visit == "P1"]), na.rm = T)
}
if (yvisit == 2) {
    hap$avgbc[i] = mean(c(bc$bc[bc$hhid ==
        yid & bc$visit == "BL"], bc$bc[bc$hhid ==
        yid & bc$visit == "P1"], bc$bc[bc$hhid ==
        yid & bc$visit == "P2"]), na.rm = T)
}
if (yvisit == 3) {
    hap$avgbc[i] = mean(c(bc$bc[bc$hhid ==
        yid & bc$visit == "BL"], bc$bc[bc$hhid ==
        yid & bc$visit == "P1"], bc$bc[bc$hhid ==
        yid & bc$visit == "P2"]), na.rm = T)
}

if (yvisit == 0) {
    hap$avgco[i] = ifelse(length(co$co[bc$hhid ==
        yid & co$visit == "BL"]) == 0,
        NA, co$co[co$hhid == yid & co$visit ==
            "BL"])
}
if (yvisit == 1) {
```

```
        hap$avgco[i] = mean(c(co$co[co$hhid ==
            yid & co$visit == "BL"], co$co[co$hhid ==
            yid & co$visit == "P1"]), na.rm = T)
    }
    if (yvisit == 2) {
        hap$avgco[i] = mean(c(co$co[co$hhid ==
            yid & co$visit == "BL"], co$co[co$hhid ==
            yid & co$visit == "P1"], co$co[co$hhid ==
            yid & co$visit == "P2"]), na.rm = T)
    }
    if (yvisit == 3) {
        hap$avgco[i] = mean(c(co$co[co$hhid ==
            yid & co$visit == "BL"], co$co[co$hhid ==
            yid & co$visit == "P1"], co$co[co$hhid ==
            yid & co$visit == "P2"]), na.rm = T)
    }

    if (yvisit == 0) {
        hap$lagpm[i] = ifelse(length(pm$pm[pm$hhid ==
            yid & pm$visit == "BL"]) == 0,
            NA, pm$pm[pm$hhid == yid & pm$visit ==
                "BL"])
    }
    if (yvisit == 1) {
        hap$lagpm[i] = mean(c(pm$pm[pm$hhid ==
            yid & pm$visit == "BL"], pm$pm[pm$hhid ==
            yid & pm$visit == "P1"]), na.rm = T)
    }
    if (yvisit == 2) {
        hap$lagpm[i] = mean(c(pm$pm[pm$hhid ==
            yid & pm$visit == "P1"], pm$pm[pm$hhid ==
            yid & pm$visit == "P2"]), na.rm = T)
    }
    if (yvisit == 3) {
        hap$lagpm[i] = ifelse(length(pm$pm[pm$hhid ==
            yid & pm$visit == "P2"]) == 0,
            NA, pm$pm[pm$hhid == yid & pm$visit ==
                "P2"])
    }

    if (yvisit == 0) {
```

```
        hap$lagbc[i] = ifelse(length(bc$bc[bc$hhid ==
            yid & bc$visit == "BL"]) == 0,
            NA, bc$bc[bc$hhid == yid & bc$visit ==
                "BL"])
    }
    if (yvisit == 1) {
        hap$lagbc[i] = mean(c(bc$bc[bc$hhid ==
            yid & bc$visit == "BL"], bc$bc[bc$hhid ==
            yid & bc$visit == "P1"]), na.rm = T)
    }
    if (yvisit == 2) {
        hap$lagbc[i] = mean(c(bc$bc[bc$hhid ==
            yid & bc$visit == "P1"], bc$bc[bc$hhid ==
            yid & bc$visit == "P2"]), na.rm = T)
    }
    if (yvisit == 3) {
        hap$lagbc[i] = ifelse(length(bc$bc[bc$hhid ==
            yid & bc$visit == "BL"]) == 0,
            NA, bc$bc[bc$hhid == yid & bc$visit ==
                "BL"])
    }

    if (yvisit == 0) {
        hap$lagco[i] = ifelse(length(co$co[bc$hhid ==
            yid & co$visit == "BL"]) == 0,
            NA, co$co[co$hhid == yid & co$visit ==
                "BL"])
    }
    if (yvisit == 1) {
        hap$lagco[i] = mean(c(co$co[co$hhid ==
            yid & co$visit == "BL"], co$co[co$hhid ==
            yid & co$visit == "P1"]), na.rm = T)
    }
    if (yvisit == 2) {
        hap$lagco[i] = mean(c(co$co[co$hhid ==
            yid & co$visit == "P1"], co$co[co$hhid ==
            yid & co$visit == "P2"]), na.rm = T)
    }
    if (yvisit == 3) {
        hap$lagco[i] = ifelse(length(co$co[bc$hhid ==
            yid & co$visit == "BL"]) == 0,
```

```
            NA, co$co[co$hhid == yid & co$visit ==
                "BL"])
    }

}

df4 = subset(df, select = c(hhid, visit,
    ga, garandom, hcz, acz, flz, efwz))

df4$xvisit = 0
df4$xvisit = ifelse(df4$visit == "P1", 1,
    df4$xvisit)
df4$xvisit = ifelse(df4$visit == "P2", 2,
    df4$xvisit)
df4$xvisit = ifelse(df4$visit == "Birth",
    3, df4$xvisit)

df4$id = df4$hhid * 100 + df4$xvisit
hap = merge(hap, df4[, -c(1, 2, 9)], by = "id",
    all = T)
hap2 = hap[is.na(hap$efwz) == F, ]
```

PM

```
## Extracting confounders from fetal
confounders = distinct(subset(df[df$visit ==
    "BL", ], select = c(hhid, tv, mobile,
    radio, bicycle, bankacct, childhh, momage,
    sleephouse, nullparous, x2handsmoke,
    momeduc, momhbalt, foodinsecure, momht,
    mommdd, pcassets)))

mydata = unique(confounders)
confounders = mydata[is.na(mydata$momage) ==
    F, ]

# hap3 <- full_join(hap2, confounders,
# by='hhid')

# Merging HAP, fetal outcome and
# confounders
hap3 = merge(hap2, confounders, by = "hhid",
    all = T)

hap3 = hap3[is.na(hap3$efwz) == F, ]

hap3$loglagpm = log(hap3$lagpm)

# remove outliers
hap4 = hap3

hap4 = hap4[(hap4$hcz > (-6) & hap4$hcz <
    6) | is.na(hap4$hcz), ]
hap4 = hap4[(hap4$flz > (-6) & hap4$flz <
    6) | is.na(hap4$flz), ]
hap4 = hap4[(hap4$acz > (-6) & hap4$acz <
    6) | is.na(hap4$acz), ]
hap4 = hap4[(hap4$efwz > (-6) & hap4$efwz <
    6) | is.na(hap4$efwz), ]

hap4$nullparous <- hap4$x2handsmoke <- hap4$momeduc <- hap4$mommdd <- NULL

hap4 <- full_join(hap4, df[df$visit == "BL",
```

```
    c("hhid", "nullparous", "x2handsmoke",
        "momeduc", "mommdd", "gabirthweeks")],
    by = "hhid") %>%
    dplyr::rename(nullparity = nullparous,
        x2handsmoke01 = x2handsmoke, mommdd01 = mommdd) %>%
    dplyr::mutate(catmomeduc = ifelse(momeduc ==
        "No formal education or Primary school incomplete",
        1, ifelse(momeduc == "Primary school complete or Secondary school incomplete",
            2, ifelse(momeduc == "Secondary school complete or Vocational or Some college
                3, NA))))


## Trim data to 97.5% of PM
quantile(sort(hap4$avgpm), 0.975)
```

```
  97.5%
324.817
```

```
hap5 = hap4[hap4$avgpm < 338, ]
hap5 = hap5[is.na(hap5$avgpm) == F, ]

### E-R analyses with averaged
### exposures BL fetal outcomes paired
### with BL exposure P1 fetal outcomes
### paired with avg of BL and P1
### exposure P2 fetal outcomes paired
### with avg of BL, P1 and P2 exposure
### Birth weight paired with with avg
### of BL, P1 and P2 exposure Need to
### create similar models for BC and CO

hap4$id = factor(hap4$hhid)
hap5$id <- hap5$hhid

hap4$catmomeduc = factor(hap4$catmomeduc)
hap5$catmomeduc = factor(hap5$catmomeduc)

hap4$birth <- ifelse(hap4$visit == "Birth",
    1, 0)
hap5$birth <- ifelse(hap5$visit == "Birth",
    1, 0)
```

```r
tab7 <- data.frame(matrix(ncol = 37, nrow = 3))
tab7.trunc <- data.frame(matrix(ncol = 37,
    nrow = 3))
tab7.lag <- data.frame(matrix(ncol = 37,
    nrow = 3))

colnames(tab7) <- colnames(tab7.trunc) <- colnames(tab7.lag) <- c("group",
    "flz_pm", "acz_pm", "hcz_pm", "efwz_pm",
    paste0(c("flz_pm", "acz_pm", "hcz_pm",
        "efwz_pm"), "_lb"), paste0(c("flz_pm",
        "acz_pm", "hcz_pm", "efwz_pm"), "_ub"),
    "flz_bc", "acz_bc", "hcz_bc", "efwz_bc",
    paste0(c("flz_bc", "acz_bc", "hcz_bc",
        "efwz_bc"), "_lb"), paste0(c("flz_bc",
        "acz_bc", "hcz_bc", "efwz_bc"), "_ub"),
    "flz_co", "acz_co", "hcz_co", "efwz_co",
    paste0(c("flz_co", "acz_co", "hcz_co",
        "efwz_co"), "_lb"), paste0(c("flz_co",
        "acz_co", "hcz_co", "efwz_co"), "_ub"))

rownames(tab7) <- tab7$group <- c("25", "33",
    "40")
rownames(tab7.trunc) <- tab7.trunc$group <- c("25",
    "33", "40")
rownames(tab7.lag) <- tab7.lag$group <- c("25",
    "33", "40")

### PM
hap.x <- hap4 %>%
    dplyr::filter(!is.na(hcz) | !is.na(acz) |
        !is.na(flz) | !is.na(efwz))

pm.quant.75 <- quantile(hap.x$pm, na.rm = T)[["75%"]]
pm.quant.25 <- quantile(hap.x$pm, na.rm = T)[["25%"]]


write.csv(hap4, "hap4.csv")
write.csv(df3, "fetal3.csv")
```

FLZ 25-weeks

```r
start.time <- Sys.time()
############## flz
model.gamm = gamm4(flz ~ t2(ga, avgpm) +
    pcassets + momht + momage + nullparity +
    mommdd01 + momhbalt + x2handsmoke01 +
    catmomeduc, data = hap4, random = ~(1 +
    ga | id), na.action = na.omit)

summary(model.gamm$gam)
```

```
Family: gaussian
Link function: identity

Formula:
flz ~ t2(ga, avgpm) + pcassets + momht + momage + nullparity +
    mommdd01 + momhbalt + x2handsmoke01 + catmomeduc

Parametric coefficients:
               Estimate Std. Error t value Pr(>|t|)
(Intercept)   -3.026993   0.481352  -6.289 3.42e-10 ***
pcassets      -0.128530   0.025366  -5.067 4.16e-07 ***
momht          0.021399   0.003027   7.069 1.73e-12 ***
momage         0.019208   0.004589   4.185 2.89e-05 ***
nullparity    -0.003891   0.043895  -0.089 0.929359
mommdd01      -0.165142   0.043097  -3.832 0.000128 ***
momhbalt      -0.013860   0.012972  -1.068 0.285342
x2handsmoke01  0.169890   0.066820   2.543 0.011030 *
catmomeduc2   -0.150043   0.048457  -3.096 0.001967 **
catmomeduc3   -0.201955   0.063768  -3.167 0.001547 **
---
Signif. codes:  0 '***' 0.001 '**' 0.01 '*' 0.05 '.' 0.1 ' ' 1

Approximate significance of smooth terms:
               edf Ref.df     F p-value
t2(ga,avgpm) 5.446  5.446 3.228 0.00743 **
---
Signif. codes:  0 '***' 0.001 '**' 0.01 '*' 0.05 '.' 0.1 ' ' 1

R-sq.(adj) =  0.0355
lmer.REML =  17501  Scale est. = 0.41262   n = 6234
```

```
##############
end.time <- Sys.time()
time.taken <- end.time - start.time
time.taken
```

```
Time difference of 11.28144 secs
```

```
predict(model.gamm$gam, newdata = list(ga = 25,
    avgpm = pm.quant.75, hhid = 13001, pcassets = 0.12,
    momht = 152.34, momage = 25.6, nullparity = 1,
    mommdd01 = 1, momhbalt = 12.6, x2handsmoke01 = 1,
    catmomeduc = 1)) - predict(model.gamm$gam,
    newdata = list(ga = 25, avgpm = pm.quant.25,
        hhid = 13001, pcassets = 0.12, momht = 152.34,
        momage = 25.6, nullparity = 1, mommdd01 = 1,
        momhbalt = 12.6, x2handsmoke01 = 1,
        catmomeduc = 1))
```

```
           1
-0.008119804
```

```
predict(model.gamm$gam, newdata = list(ga = 25,
    avgpm = pm.quant.75, hhid = 13001, pcassets = 0.12,
    momht = 152.34, momage = 25.6, nullparity = 1,
    mommdd01 = 1, momhbalt = 12.6, x2handsmoke01 = 1,
    catmomeduc = 1)) - predict(model.gamm$gam,
    newdata = list(ga = 25, avgpm = pm.quant.25,
        hhid = 13001, pcassets = 0.12, momht = 152.34,
        momage = 25.6, nullparity = 1, mommdd01 = 1,
        momhbalt = 12.6, x2handsmoke01 = 1,
        catmomeduc = 1))
```

```
           1
-0.008119804
```

```
# calc diffs and 95% CIs
beta = coef(model.gamm$gam)
V = vcov(model.gamm$gam)
# at 25 weeks
```

```r
df_pred = data.frame(avgpm = c(pm.quant.75,
    pm.quant.25), ga = 25, hhid = 13001,
    pcassets = 0.12, momht = 152.34, momage = 25.6,
    nullparity = 1, mommdd01 = 1, momhbalt = 12.6,
    x2handsmoke01 = 1, catmomeduc = 1)
phi_mat <- predict(model.gamm$gam, type = "lpmatrix",
    newdata = df_pred)
dX <- beta[11:34] %*% phi_mat[1, 11:34] -
    beta[11:34] %*% phi_mat[2, 11:34]

vc <- V[c(11:34), c(11:34)]
a <- phi_mat[2, 11:34] - phi_mat[1, 11:34]
vX <- (t(a) %*% vc %*% a)

c(dX, dX - qnorm(0.99375) * sqrt(vX), dX +
    qnorm(0.99375) * sqrt(vX))
```

```
[1] -0.008119804 -0.059188708  0.042949100
```

```r
tab7["25", c("flz_pm", "flz_pm_lb", "flz_pm_ub")] <- c(dX,
    dX - qnorm(0.99375) * sqrt(vX), dX +
        qnorm(0.99375) * sqrt(vX))
```

FLZ 33-weeks

```r
# at 33 weeks
df_pred = data.frame(avgpm = c(pm.quant.75,
    pm.quant.25), ga = 33, hhid = 13001,
    pcassets = 0.12, momht = 152.34, momage = 25.6,
    nullparity = 1, mommdd01 = 1, momhbalt = 12.6,
    x2handsmoke01 = 1, catmomeduc = 1)
phi_mat <- predict(model.gamm$gam, type = "lpmatrix",
    newdata = df_pred)
dX <- beta[11:34] %*% phi_mat[1, 11:34] -
    beta[11:34] %*% phi_mat[2, 11:34]

vc <- V[c(11:34), c(11:34)]
a <- phi_mat[2, 11:34] - phi_mat[1, 11:34]
vX <- (t(a) %*% vc %*% a)
```

```r
c(dX, dX - qnorm(0.99375) * sqrt(vX), dX +
    qnorm(0.99375) * sqrt(vX))
```

```
[1] -0.04174175 -0.09565492  0.01217141
```

```r
tab7["33", c("flz_pm", "flz_pm_lb", "flz_pm_ub")] <- c(dX,
    dX - qnorm(0.99375) * sqrt(vX), dX +
        qnorm(0.99375) * sqrt(vX))
```

HCZ 25-weeks

```r
### hcz

model.gamm = gamm4(hcz ~ t2(ga, avgpm) +
    pcassets + momht + momage + nullparity +
    mommdd01 + momhbalt + x2handsmoke01 +
    catmomeduc, data = hap4, random = ~(1 +
    ga | id), na.action = na.omit)

summary(model.gamm$gam)
```

```
Family: gaussian
Link function: identity

Formula:
hcz ~ t2(ga, avgpm) + pcassets + momht + momage + nullparity +
    mommdd01 + momhbalt + x2handsmoke01 + catmomeduc

Parametric coefficients:
               Estimate Std. Error t value Pr(>|t|)
(Intercept)   -5.698870   0.474498 -12.010  < 2e-16 ***
pcassets       0.125500   0.025033   5.013  5.5e-07 ***
momht          0.033494   0.002985  11.221  < 2e-16 ***
momage         0.039055   0.004527   8.627  < 2e-16 ***
nullparity     0.050899   0.043320   1.175  0.24006
mommdd01       0.008095   0.042565   0.190  0.84917
momhbalt      -0.015309   0.012794  -1.197  0.23150
x2handsmoke01  0.006647   0.065859   0.101  0.91961
```

```
catmomeduc2    0.110190   0.047757   2.307  0.02107 *
catmomeduc3    0.201728   0.062906   3.207  0.00135 **
---
Signif. codes:  0 '***' 0.001 '**' 0.01 '*' 0.05 '.' 0.1 ' ' 1

Approximate significance of smooth terms:
               edf Ref.df     F p-value
t2(ga,avgpm) 7.457  7.457 13.61  <2e-16 ***
---
Signif. codes:  0 '***' 0.001 '**' 0.01 '*' 0.05 '.' 0.1 ' ' 1

R-sq.(adj) =  0.0859
lmer.REML =  17579  Scale est. = 0.38445   n = 6234
```

```r
predict(model.gamm$gam, newdata = list(ga = 25,
    avgpm = pm.quant.75, hhid = 13001, pcassets = 0.12,
    momht = 152.34, momage = 25.6, nullparity = 1,
    mommdd01 = 1, momhbalt = 12.6, x2handsmoke01 = 1,
    catmomeduc = 1)) - predict(model.gamm$gam,
    newdata = list(ga = 25, avgpm = pm.quant.25,
        hhid = 13001, pcassets = 0.12, momht = 152.34,
        momage = 25.6, nullparity = 1, mommdd01 = 1,
        momhbalt = 12.6, x2handsmoke01 = 1,
        catmomeduc = 1))
```

```
         1
-0.0383945
```

```r
predict(model.gamm$gam, newdata = list(ga = 25,
    avgpm = pm.quant.75, hhid = 13001, pcassets = 0.12,
    momht = 152.34, momage = 25.6, nullparity = 1,
    mommdd01 = 1, momhbalt = 12.6, x2handsmoke01 = 1,
    catmomeduc = 1)) - predict(model.gamm$gam,
    newdata = list(ga = 25, avgpm = pm.quant.25,
        hhid = 13001, pcassets = 0.12, momht = 152.34,
        momage = 25.6, nullparity = 1, mommdd01 = 1,
        momhbalt = 12.6, x2handsmoke01 = 1,
        catmomeduc = 1))
```

```
         1
-0.0383945
```

```
# calc diffs and 95% CIs
beta = coef(model.gamm$gam)
V = vcov(model.gamm$gam)
# at 25 weeks
df_pred = data.frame(avgpm = c(pm.quant.75,
    pm.quant.25), ga = 25, hhid = 13001,
    pcassets = 0.12, momht = 152.34, momage = 25.6,
    nullparity = 1, mommdd01 = 1, momhbalt = 12.6,
    x2handsmoke01 = 1, catmomeduc = 1)
phi_mat <- predict(model.gamm$gam, type = "lpmatrix",
    newdata = df_pred)
dX <- beta[11:34] %*% phi_mat[1, 11:34] -
    beta[11:34] %*% phi_mat[2, 11:34]

vc <- V[c(11:34), c(11:34)]
a <- phi_mat[2, 11:34] - phi_mat[1, 11:34]
vX <- (t(a) %*% vc %*% a)

c(dX, dX - qnorm(0.99375) * sqrt(vX), dX +
    qnorm(0.99375) * sqrt(vX))
```

```
[1] -0.03839450 -0.08390912  0.00712011
```

```
tab7["25", c("hcz_pm", "hcz_pm_lb", "hcz_pm_ub")] <- c(dX,
    dX - qnorm(0.99375) * sqrt(vX), dX +
        qnorm(0.99375) * sqrt(vX))
```

HCZ 33-weeks

```
# at 33 weeks
df_pred = data.frame(avgpm = c(pm.quant.75,
    pm.quant.25), ga = 33, hhid = 13001,
    pcassets = 0.12, momht = 152.34, momage = 25.6,
    nullparity = 1, mommdd01 = 1, momhbalt = 12.6,
    x2handsmoke01 = 1, catmomeduc = 1)
phi_mat <- predict(model.gamm$gam, type = "lpmatrix",
    newdata = df_pred)
dX <- beta[11:34] %*% phi_mat[1, 11:34] -
    beta[11:34] %*% phi_mat[2, 11:34]
```

```r
vc <- V[c(11:34), c(11:34)]
a <- phi_mat[2, 11:34] - phi_mat[1, 11:34]
vX <- (t(a) %*% vc %*% a)

c(dX, dX - qnorm(0.99375) * sqrt(vX), dX +
    qnorm(0.99375) * sqrt(vX))
```

```
[1] -0.04222766 -0.09755476  0.01309944
```

```r
tab7["33", c("hcz_pm", "hcz_pm_lb", "hcz_pm_ub")] <- c(dX,
    dX - qnorm(0.99375) * sqrt(vX), dX +
        qnorm(0.99375) * sqrt(vX))
```

ACZ 25-weeks

```r
### acz

model.gamm = gamm4(acz ~ t2(ga, avgpm) +
    pcassets + momht + momage + nullparity +
    mommdd01 + momhbalt + x2handsmoke01 +
    catmomeduc, data = hap4, random = ~(1 +
    ga | id), na.action = na.omit)

summary(model.gamm$gam)
```

```
Family: gaussian
Link function: identity

Formula:
acz ~ t2(ga, avgpm) + pcassets + momht + momage + nullparity +
    mommdd01 + momhbalt + x2handsmoke01 + catmomeduc

Parametric coefficients:
               Estimate Std. Error t value Pr(>|t|)
(Intercept)   -3.552840   0.487987  -7.281 3.73e-13 ***
pcassets       0.216042   0.025825   8.366  < 2e-16 ***
momht          0.019210   0.003067   6.263 4.03e-10 ***
momage         0.030108   0.004648   6.477 1.00e-10 ***
```

```
nullparity    -0.151648   0.044535  -3.405 0.000665 ***
mommdd01       0.446120   0.043615  10.229  < 2e-16 ***
momhbalt      -0.006689   0.013172  -0.508 0.611582    
x2handsmoke01 -0.253869   0.068336  -3.715 0.000205 ***
catmomeduc2    0.234710   0.049230   4.768 1.91e-06 ***
catmomeduc3    0.494931   0.064842   7.633 2.64e-14 ***
---
Signif. codes:  0 '***' 0.001 '**' 0.01 '*' 0.05 '.' 0.1 ' ' 1

Approximate significance of smooth terms:
               edf Ref.df     F  p-value    
t2(ga,avgpm) 5.893  5.893 4.282 0.000289 ***
---
Signif. codes:  0 '***' 0.001 '**' 0.01 '*' 0.05 '.' 0.1 ' ' 1

R-sq.(adj) =  0.0973   
lmer.REML =  18041  Scale est. = 0.4418    n = 6234
```

```r
predict(model.gamm$gam, newdata = list(ga = 25,
    avgpm = pm.quant.75, hhid = 13001, pcassets = 0.12,
    momht = 152.34, momage = 25.6, nullparity = 1,
    mommdd01 = 1, momhbalt = 12.6, x2handsmoke01 = 1,
    catmomeduc = 1)) - predict(model.gamm$gam,
    newdata = list(ga = 25, avgpm = pm.quant.25,
        hhid = 13001, pcassets = 0.12, momht = 152.34,
        momage = 25.6, nullparity = 1, mommdd01 = 1,
        momhbalt = 12.6, x2handsmoke01 = 1,
        catmomeduc = 1))
```

```
          1 
-0.04878645 
```

```r
predict(model.gamm$gam, newdata = list(ga = 25,
    avgpm = pm.quant.75, hhid = 13001, pcassets = 0.12,
    momht = 152.34, momage = 25.6, nullparity = 1,
    mommdd01 = 1, momhbalt = 12.6, x2handsmoke01 = 1,
    catmomeduc = 1)) - predict(model.gamm$gam,
    newdata = list(ga = 25, avgpm = pm.quant.25,
        hhid = 13001, pcassets = 0.12, momht = 152.34,
        momage = 25.6, nullparity = 1, mommdd01 = 1,
        momhbalt = 12.6, x2handsmoke01 = 1,
```

```
        catmomeduc = 1))
```

```
          1
-0.04878645
```

```
# calc diffs and 95% CIs
beta = coef(model.gamm$gam)
V = vcov(model.gamm$gam)
# at 25 weeks
df_pred = data.frame(avgpm = c(pm.quant.75,
    pm.quant.25), ga = 25, hhid = 13001,
    pcassets = 0.12, momht = 152.34, momage = 25.6,
    nullparity = 1, mommdd01 = 1, momhbalt = 12.6,
    x2handsmoke01 = 1, catmomeduc = 1)
phi_mat <- predict(model.gamm$gam, type = "lpmatrix",
    newdata = df_pred)
dX <- beta[11:34] %*% phi_mat[1, 11:34] -
    beta[11:34] %*% phi_mat[2, 11:34]

vc <- V[c(11:34), c(11:34)]
a <- phi_mat[2, 11:34] - phi_mat[1, 11:34]
vX <- (t(a) %*% vc %*% a)

c(dX, dX - qnorm(0.99375) * sqrt(vX), dX +
    qnorm(0.99375) * sqrt(vX))
```

```
[1] -0.048786449 -0.100452729  0.002879831
```

```
tab7["25", c("acz_pm", "acz_pm_lb", "acz_pm_ub")] <- c(dX,
    dX - qnorm(0.99375) * sqrt(vX), dX +
        qnorm(0.99375) * sqrt(vX))
```

ACZ 33-weeks

```
# at 33 weeks
df_pred = data.frame(avgpm = c(pm.quant.75,
    pm.quant.25), ga = 33, hhid = 13001,
    pcassets = 0.12, momht = 152.34, momage = 25.6,
```

```
    nullparity = 1, mommdd01 = 1, momhbalt = 12.6,
    x2handsmoke01 = 1, catmomeduc = 1)
phi_mat <- predict(model.gamm$gam, type = "lpmatrix",
    newdata = df_pred)
dX <- beta[11:34] %*% phi_mat[1, 11:34] -
    beta[11:34] %*% phi_mat[2, 11:34]

vc <- V[c(11:34), c(11:34)]
a <- phi_mat[2, 11:34] - phi_mat[1, 11:34]
vX <- (t(a) %*% vc %*% a)

c(dX, dX - qnorm(0.99375) * sqrt(vX), dX +
    qnorm(0.99375) * sqrt(vX))
```

```
[1] -0.04210241 -0.09621209  0.01200726
```

```
tab7["33", c("acz_pm", "acz_pm_lb", "acz_pm_ub")] <- c(dX,
    dX - qnorm(0.99375) * sqrt(vX), dX +
        qnorm(0.99375) * sqrt(vX))
```

EFWZ 25-weeks

```
### efwz
model.gamm = gamm4(efwz ~ t2(ga, avgpm) +
    pcassets + momht + momage + nullparity +
    mommdd01 + momhbalt + x2handsmoke01 +
    catmomeduc, data = hap4, random = ~(1 +
    ga | id), na.action = na.omit)

summary(model.gamm$gam)
```

```
Family: gaussian
Link function: identity

Formula:
efwz ~ t2(ga, avgpm) + pcassets + momht + momage + nullparity +
    mommdd01 + momhbalt + x2handsmoke01 + catmomeduc
```

```
Parametric coefficients:
              Estimate Std. Error t value Pr(>|t|)
(Intercept)   -5.622598   0.415851 -13.521  < 2e-16 ***
pcassets       0.141524   0.021411   6.610 4.06e-11 ***
momht          0.028760   0.002614  11.003  < 2e-16 ***
momage         0.030274   0.003979   7.609 3.03e-14 ***
nullparity    -0.195475   0.037629  -5.195 2.09e-07 ***
mommdd01       0.344904   0.037456   9.208  < 2e-16 ***
momhbalt       0.021472   0.011199   1.917  0.05524 .
x2handsmoke01 -0.176226   0.054271  -3.247  0.00117 **
catmomeduc2    0.189095   0.041312   4.577 4.77e-06 ***
catmomeduc3    0.418765   0.053763   7.789 7.49e-15 ***
---
Signif. codes:  0 '***' 0.001 '**' 0.01 '*' 0.05 '.' 0.1 ' ' 1

Approximate significance of smooth terms:
               edf Ref.df    F p-value
t2(ga,avgpm) 8.963  8.963 96.5  <2e-16 ***
---
Signif. codes:  0 '***' 0.001 '**' 0.01 '*' 0.05 '.' 0.1 ' ' 1

R-sq.(adj) =  0.266
lmer.REML =  24192  Scale est. = 0.35156   n = 9123
```

```r
# calc diffs and 95% CIs
beta = coef(model.gamm$gam)
V = vcov(model.gamm$gam)
# at 25 weeks
df_pred = data.frame(avgpm = c(pm.quant.75,
    pm.quant.25), ga = 25, hhid = 13001,
    pcassets = 0.12, momht = 152.34, momage = 25.6,
    nullparity = 1, mommdd01 = 1, momhbalt = 12.6,
    x2handsmoke01 = 1, catmomeduc = 1)
phi_mat <- predict(model.gamm$gam, type = "lpmatrix",
    newdata = df_pred)
dX <- beta[11:34] %*% phi_mat[1, 11:34] -
    beta[11:34] %*% phi_mat[2, 11:34]

vc <- V[c(11:34), c(11:34)]
a <- phi_mat[2, 11:34] - phi_mat[1, 11:34]
vX <- (t(a) %*% vc %*% a)
```

```
c(dX, dX - qnorm(0.99375) * sqrt(vX), dX +
    qnorm(0.99375) * sqrt(vX))
```

```
[1] -0.03266810 -0.08473238  0.01939619
```

```
tab7["25", c("efwz_pm", "efwz_pm_lb", "efwz_pm_ub")] <- c(dX,
    dX - qnorm(0.99375) * sqrt(vX), dX +
        qnorm(0.99375) * sqrt(vX))
```

EFWZ 33-weeks

```
# at 33 weeks
df_pred = data.frame(avgpm = c(pm.quant.75,
    pm.quant.25), ga = 33, hhid = 13001,
    pcassets = 0.12, momht = 152.34, momage = 25.6,
    nullparity = 1, mommdd01 = 1, momhbalt = 12.6,
    x2handsmoke01 = 1, catmomeduc = 1)
phi_mat <- predict(model.gamm$gam, type = "lpmatrix",
    newdata = df_pred)
dX <- beta[11:34] %*% phi_mat[1, 11:34] -
    beta[11:34] %*% phi_mat[2, 11:34]

vc <- V[c(11:34), c(11:34)]
a <- phi_mat[2, 11:34] - phi_mat[1, 11:34]
vX <- (t(a) %*% vc %*% a)

c(dX, dX - qnorm(0.99375) * sqrt(vX), dX +
    qnorm(0.99375) * sqrt(vX))
```

```
[1] -0.05474133 -0.10330194 -0.00618071
```

```
tab7["33", c("efwz_pm", "efwz_pm_lb", "efwz_pm_ub")] <- c(dX,
    dX - qnorm(0.99375) * sqrt(vX), dX +
        qnorm(0.99375) * sqrt(vX))
```

EFWZ 40-weeks

```r
# at Birth
df_pred = data.frame(avgpm = c(pm.quant.75,
    pm.quant.25), ga = 40, birth = 1, hhid = 13001,
    pcassets = 0.12, momht = 152.34, momage = 25.6,
    nullparity = 1, mommdd01 = 1, momhbalt = 12.6,
    x2handsmoke01 = 1, catmomeduc = 1)

phi_mat <- predict(model.gamm$gam, type = "lpmatrix",
    newdata = df_pred)
dX <- beta[c(11:34)] %*% phi_mat[1, c(11:34)] -
    beta[c(11:34)] %*% phi_mat[2, c(11:34)]

vc <- V[c(11:34), c(11:34)]
a <- phi_mat[2, c(11:34)] - phi_mat[1, c(11:34)]
vX <- (t(a) %*% vc %*% a)

c(dX, dX - qnorm(0.99375) * sqrt(vX), dX +
    qnorm(0.99375) * sqrt(vX))
```

```
[1] -0.0525838035 -0.1057986786  0.0006310717
```

```r
tab7["40", c("efwz_pm", "efwz_pm_lb", "efwz_pm_ub")] <- c(dX,
    dX - qnorm(0.99375) * sqrt(vX), dX +
        qnorm(0.99375) * sqrt(vX))
```

BC

FLZ 25-week

```r
bc.quant.75 <- quantile(hap.x$bc, na.rm = T)[["75%"]]
bc.quant.25 <- quantile(hap.x$bc, na.rm = T)[["25%"]]

### flz
model.gamm = gamm4(flz ~ t2(ga, avgbc) +
    pcassets + momht + momage + nullparity +
    mommdd01 + momhbalt + x2handsmoke01 +
    catmomeduc, data = hap4, random = ~(1 +
    ga | id), na.action = na.omit)

summary(model.gamm$gam)
```

```
Family: gaussian
Link function: identity

Formula:
flz ~ t2(ga, avgbc) + pcassets + momht + momage + nullparity +
    mommdd01 + momhbalt + x2handsmoke01 + catmomeduc

Parametric coefficients:
               Estimate Std. Error t value Pr(>|t|)
(Intercept)   -3.003879   0.486887  -6.170 7.30e-10 ***
pcassets      -0.129740   0.025599  -5.068 4.14e-07 ***
momht          0.021484   0.003056   7.029 2.31e-12 ***
momage         0.018970   0.004635   4.093 4.32e-05 ***
nullparity    -0.012137   0.044392  -0.273 0.784550
mommdd01      -0.162991   0.043523  -3.745 0.000182 ***
momhbalt      -0.015769   0.013135  -1.201 0.229983
x2handsmoke01  0.183153   0.067252   2.723 0.006480 **
catmomeduc2   -0.152768   0.049007  -3.117 0.001834 **
catmomeduc3   -0.206101   0.064415  -3.200 0.001383 **
---
Signif. codes:  0 '***' 0.001 '**' 0.01 '*' 0.05 '.' 0.1 ' ' 1

Approximate significance of smooth terms:
               edf Ref.df     F p-value
t2(ga,avgbc) 5.539  5.539 2.853 0.00756 **
---
Signif. codes:  0 '***' 0.001 '**' 0.01 '*' 0.05 '.' 0.1 ' ' 1

R-sq.(adj) =  0.0358
lmer.REML =  16805  Scale est. = 0.41902   n = 5971
```

```r
predict(model.gamm$gam, newdata = list(ga = 25,
    avgbc = bc.quant.75, hhid = 13001, pcassets = 0.12,
    momht = 152.34, momage = 25.6, nullparity = 1,
    mommdd01 = 1, momhbalt = 12.6, x2handsmoke01 = 1,
    catmomeduc = 1)) - predict(model.gamm$gam,
    newdata = list(ga = 25, avgbc = bc.quant.25,
        hhid = 13001, pcassets = 0.12, momht = 152.34,
        momage = 25.6, nullparity = 1, mommdd01 = 1,
        momhbalt = 12.6, x2handsmoke01 = 1,
        catmomeduc = 1))
```

```
           1
-0.003709864
```

```
predict(model.gamm$gam, newdata = list(ga = 25,
    avgbc = bc.quant.75, hhid = 13001, pcassets = 0.12,
    momht = 152.34, momage = 25.6, nullparity = 1,
    mommdd01 = 1, momhbalt = 12.6, x2handsmoke01 = 1,
    catmomeduc = 1)) - predict(model.gamm$gam,
    newdata = list(ga = 25, avgbc = bc.quant.25,
        hhid = 13001, pcassets = 0.12, momht = 152.34,
        momage = 25.6, nullparity = 1, mommdd01 = 1,
        momhbalt = 12.6, x2handsmoke01 = 1,
        catmomeduc = 1))
```

```
           1
-0.003709864
```

```
# calc diffs and 95% CIs
beta = coef(model.gamm$gam)
V = vcov(model.gamm$gam)
# at 25 weeks
df_pred = data.frame(avgbc = c(bc.quant.75,
    bc.quant.25), ga = 25, hhid = 13001,
    pcassets = 0.12, momht = 152.34, momage = 25.6,
    nullparity = 1, mommdd01 = 1, momhbalt = 12.6,
    x2handsmoke01 = 1, catmomeduc = 1)
phi_mat <- predict(model.gamm$gam, type = "lpmatrix",
    newdata = df_pred)
dX <- beta[11:34] %*% phi_mat[1, 11:34] -
    beta[11:34] %*% phi_mat[2, 11:34]

vc <- V[c(11:34), c(11:34)]
a <- phi_mat[2, 11:34] - phi_mat[1, 11:34]
vX <- (t(a) %*% vc %*% a)

c(dX, dX - qnorm(0.99375) * sqrt(vX), dX +
    qnorm(0.99375) * sqrt(vX))
```

```
[1] -0.003709864 -0.073259894  0.065840167
```

```
tab7["25", c("flz_bc", "flz_bc_lb", "flz_bc_ub")] <- c(dX,
    dX - qnorm(0.99375) * sqrt(vX), dX +
        qnorm(0.99375) * sqrt(vX))
```

FLZ 33-weeks

```
# at 33 weeks
df_pred = data.frame(avgbc = c(bc.quant.75,
    bc.quant.25), ga = 33, hhid = 13001,
    pcassets = 0.12, momht = 152.34, momage = 25.6,
    nullparity = 1, mommdd01 = 1, momhbalt = 12.6,
    x2handsmoke01 = 1, catmomeduc = 1)
phi_mat <- predict(model.gamm$gam, type = "lpmatrix",
    newdata = df_pred)
dX <- beta[11:34] %*% phi_mat[1, 11:34] -
    beta[11:34] %*% phi_mat[2, 11:34]

vc <- V[c(11:34), c(11:34)]
a <- phi_mat[2, 11:34] - phi_mat[1, 11:34]
vX <- (t(a) %*% vc %*% a)

c(dX, dX - qnorm(0.99375) * sqrt(vX), dX +
    qnorm(0.99375) * sqrt(vX))
```

```
[1] -0.06027148 -0.13233364  0.01179068
```

```
tab7["33", c("flz_bc", "flz_bc_lb", "flz_bc_ub")] <- c(dX,
    dX - qnorm(0.99375) * sqrt(vX), dX +
        qnorm(0.99375) * sqrt(vX))
```

HCZ 25-week

```
model.gamm = gamm4(hcz ~ t2(ga, avgbc) +
    pcassets + momht + momage + nullparity +
    mommdd01 + momhbalt + x2handsmoke01 +
    catmomeduc, data = hap4, random = ~(1 +
    ga | id), na.action = na.omit)

summary(model.gamm$gam)
```

```
Family: gaussian
Link function: identity

Formula:
hcz ~ t2(ga, avgbc) + pcassets + momht + momage + nullparity +
    mommdd01 + momhbalt + x2handsmoke01 + catmomeduc

Parametric coefficients:
               Estimate Std. Error t value Pr(>|t|)
(Intercept)   -5.813459   0.479912 -12.114  < 2e-16 ***
pcassets       0.123864   0.025260   4.904 9.66e-07 ***
momht          0.034129   0.003013  11.328  < 2e-16 ***
momage         0.039365   0.004569   8.616  < 2e-16 ***
nullparity     0.031385   0.043782   0.717  0.47350
mommdd01       0.005065   0.042944   0.118  0.90612
momhbalt      -0.014776   0.012954  -1.141  0.25405
x2handsmoke01  0.017525   0.066301   0.264  0.79153
catmomeduc2    0.107401   0.048281   2.224  0.02615 *
catmomeduc3    0.208506   0.063526   3.282  0.00104 **
---
Signif. codes:  0 '***' 0.001 '**' 0.01 '*' 0.05 '.' 0.1 ' ' 1

Approximate significance of smooth terms:
               edf Ref.df     F p-value
t2(ga,avgbc) 6.563  6.563 9.304  <2e-16 ***
---
Signif. codes:  0 '***' 0.001 '**' 0.01 '*' 0.05 '.' 0.1 ' ' 1

R-sq.(adj) =  0.0832
lmer.REML =  16877  Scale est. = 0.38903   n = 5971
```

```
predict(model.gamm$gam, newdata = list(ga = 25,
    avgbc = bc.quant.75, hhid = 13001, pcassets = 0.12,
    momht = 152.34, momage = 25.6, nullparity = 1,
    mommdd01 = 1, momhbalt = 12.6, x2handsmoke01 = 1,
    catmomeduc = 1)) - predict(model.gamm$gam,
    newdata = list(ga = 25, avgbc = bc.quant.25,
        hhid = 13001, pcassets = 0.12, momht = 152.34,
        momage = 25.6, nullparity = 1, mommdd01 = 1,
        momhbalt = 12.6, x2handsmoke01 = 1,
        catmomeduc = 1))
```

```
        1
-0.0476779
```

```
predict(model.gamm$gam, newdata = list(ga = 25,
    avgbc = bc.quant.75, hhid = 13001, pcassets = 0.12,
    momht = 152.34, momage = 25.6, nullparity = 1,
    mommdd01 = 1, momhbalt = 12.6, x2handsmoke01 = 1,
    catmomeduc = 1)) - predict(model.gamm$gam,
    newdata = list(ga = 25, avgbc = bc.quant.25,
        hhid = 13001, pcassets = 0.12, momht = 152.34,
        momage = 25.6, nullparity = 1, mommdd01 = 1,
        momhbalt = 12.6, x2handsmoke01 = 1,
        catmomeduc = 1))
```

```
        1
-0.0476779
```

```
# calc diffs and 95% CIs
beta = coef(model.gamm$gam)
V = vcov(model.gamm$gam)
# at 25 weeks
df_pred = data.frame(avgbc = c(bc.quant.75,
    bc.quant.25), ga = 25, hhid = 13001,
    pcassets = 0.12, momht = 152.34, momage = 25.6,
    nullparity = 1, mommdd01 = 1, momhbalt = 12.6,
    x2handsmoke01 = 1, catmomeduc = 1)
phi_mat <- predict(model.gamm$gam, type = "lpmatrix",
    newdata = df_pred)
dX <- beta[11:34] %*% phi_mat[1, 11:34] -
    beta[11:34] %*% phi_mat[2, 11:34]

vc <- V[c(11:34), c(11:34)]
a <- phi_mat[2, 11:34] - phi_mat[1, 11:34]
vX <- (t(a) %*% vc %*% a)

c(dX, dX - qnorm(0.99375) * sqrt(vX), dX +
    qnorm(0.99375) * sqrt(vX))
```

```
[1] -0.04767790 -0.11120289  0.01584708
```

```
tab7["25", c("hcz_bc", "hcz_bc_lb", "hcz_bc_ub")] <- c(dX,
    dX - qnorm(0.99375) * sqrt(vX), dX +
        qnorm(0.99375) * sqrt(vX))
```

HCZ 33-week

```
# at 33 weeks
df_pred = data.frame(avgbc = c(bc.quant.75,
    bc.quant.25), ga = 33, hhid = 13001,
    pcassets = 0.12, momht = 152.34, momage = 25.6,
    nullparity = 1, mommdd01 = 1, momhbalt = 12.6,
    x2handsmoke01 = 1, catmomeduc = 1)
phi_mat <- predict(model.gamm$gam, type = "lpmatrix",
    newdata = df_pred)
dX <- beta[11:34] %*% phi_mat[1, 11:34] -
    beta[11:34] %*% phi_mat[2, 11:34]

vc <- V[c(11:34), c(11:34)]
a <- phi_mat[2, 11:34] - phi_mat[1, 11:34]
vX <- (t(a) %*% vc %*% a)

c(dX, dX - qnorm(0.99375) * sqrt(vX), dX +
    qnorm(0.99375) * sqrt(vX))
```

```
[1] -0.05882783 -0.13147663  0.01382098
```

```
tab7["33", c("hcz_bc", "hcz_bc_lb", "hcz_bc_ub")] <- c(dX,
    dX - qnorm(0.99375) * sqrt(vX), dX +
        qnorm(0.99375) * sqrt(vX))
```

ACZ 25-week

```
### acz

model.gamm = gamm4(acz ~ t2(ga, avgbc) +
    pcassets + momht + momage + nullparity +
    mommdd01 + momhbalt + x2handsmoke01 +
    catmomeduc, data = hap4, random = ~(1 +
    ga | id), na.action = na.omit)
```

```r
summary(model.gamm$gam)
```

```

Family: gaussian
Link function: identity

Formula:
acz ~ t2(ga, avgbc) + pcassets + momht + momage + nullparity +
    mommdd01 + momhbalt + x2handsmoke01 + catmomeduc

Parametric coefficients:
               Estimate Std. Error t value Pr(>|t|)
(Intercept)   -3.723762   0.493614  -7.544 5.25e-14 ***
pcassets       0.210984   0.026080   8.090 7.18e-16 ***
momht          0.020100   0.003097   6.489 9.32e-11 ***
momage         0.031280   0.004697   6.660 2.99e-11 ***
nullparity    -0.161754   0.045061  -3.590 0.000334 ***
mommdd01       0.463229   0.044108  10.502  < 2e-16 ***
momhbalt      -0.006519   0.013337  -0.489 0.625035
x2handsmoke01 -0.254650   0.068777  -3.703 0.000215 ***
catmomeduc2    0.236898   0.049791   4.758 2.00e-06 ***
catmomeduc3    0.499621   0.065521   7.625 2.82e-14 ***
---
Signif. codes:  0 '***' 0.001 '**' 0.01 '*' 0.05 '.' 0.1 ' ' 1

Approximate significance of smooth terms:
               edf Ref.df     F  p-value
t2(ga,avgbc) 7.379  7.379 6.196 3.04e-06 ***
---
Signif. codes:  0 '***' 0.001 '**' 0.01 '*' 0.05 '.' 0.1 ' ' 1

R-sq.(adj) =  0.103
lmer.REML =  17239  Scale est. = 0.43401   n = 5971
```

```r
predict(model.gamm$gam, newdata = list(ga = 25,
    avgbc = bc.quant.75, hhid = 13001, pcassets = 0.12,
    momht = 152.34, momage = 25.6, nullparity = 1,
    mommdd01 = 1, momhbalt = 12.6, x2handsmoke01 = 1,
    catmomeduc = 1)) - predict(model.gamm$gam,
    newdata = list(ga = 25, avgbc = bc.quant.25,
```

```
        hhid = 13001, pcassets = 0.12, momht = 152.34,
        momage = 25.6, nullparity = 1, mommdd01 = 1,
        momhbalt = 12.6, x2handsmoke01 = 1,
        catmomeduc = 1))
```

```
          1
-0.01844812
```

```
predict(model.gamm$gam, newdata = list(ga = 25,
    avgbc = bc.quant.75, hhid = 13001, pcassets = 0.12,
    momht = 152.34, momage = 25.6, nullparity = 1,
    mommdd01 = 1, momhbalt = 12.6, x2handsmoke01 = 1,
    catmomeduc = 1)) - predict(model.gamm$gam,
    newdata = list(ga = 25, avgbc = bc.quant.25,
        hhid = 13001, pcassets = 0.12, momht = 152.34,
        momage = 25.6, nullparity = 1, mommdd01 = 1,
        momhbalt = 12.6, x2handsmoke01 = 1,
        catmomeduc = 1))
```

```
          1
-0.01844812
```

```
# calc diffs and 95% CIs
beta = coef(model.gamm$gam)
V = vcov(model.gamm$gam)
# at 25 weeks
df_pred = data.frame(avgbc = c(bc.quant.75,
    bc.quant.25), ga = 25, hhid = 13001,
    pcassets = 0.12, momht = 152.34, momage = 25.6,
    nullparity = 1, mommdd01 = 1, momhbalt = 12.6,
    x2handsmoke01 = 1, catmomeduc = 1)
phi_mat <- predict(model.gamm$gam, type = "lpmatrix",
    newdata = df_pred)
dX <- beta[11:34] %*% phi_mat[1, 11:34] -
    beta[11:34] %*% phi_mat[2, 11:34]

vc <- V[c(11:34), c(11:34)]
a <- phi_mat[2, 11:34] - phi_mat[1, 11:34]
vX <- (t(a) %*% vc %*% a)
```

```r
c(dX, dX - qnorm(0.99375) * sqrt(vX), dX +
    qnorm(0.99375) * sqrt(vX))
```

```
[1] -0.01844812 -0.08845351  0.05155727
```

```r
tab7["25", c("acz_bc", "acz_bc_lb", "acz_bc_ub")] <- c(dX,
    dX - qnorm(0.99375) * sqrt(vX), dX +
        qnorm(0.99375) * sqrt(vX))
```

ACZ 33-weeks

```r
# at 33 weeks
df_pred = data.frame(avgbc = c(bc.quant.75,
    bc.quant.25), ga = 33, hhid = 13001,
    pcassets = 0.12, momht = 152.34, momage = 25.6,
    nullparity = 1, mommdd01 = 1, momhbalt = 12.6,
    x2handsmoke01 = 1, catmomeduc = 1)
phi_mat <- predict(model.gamm$gam, type = "lpmatrix",
    newdata = df_pred)
dX <- beta[11:34] %*% phi_mat[1, 11:34] -
    beta[11:34] %*% phi_mat[2, 11:34]

vc <- V[c(11:34), c(11:34)]
a <- phi_mat[2, 11:34] - phi_mat[1, 11:34]
vX <- (t(a) %*% vc %*% a)

c(dX, dX - qnorm(0.99375) * sqrt(vX), dX +
    qnorm(0.99375) * sqrt(vX))
```

```
[1] -0.02322903 -0.09355163  0.04709357
```

```r
tab7["33", c("acz_bc", "acz_bc_lb", "acz_bc_ub")] <- c(dX,
    dX - qnorm(0.99375) * sqrt(vX), dX +
        qnorm(0.99375) * sqrt(vX))
```

EFWZ 25-week

```r
### efwz

model.gamm = gamm4(efwz ~ t2(ga, avgbc) +
    pcassets + momht + momage + nullparity +
    mommdd01 + momhbalt + x2handsmoke01 +
    catmomeduc, data = hap4, random = ~(1 +
    ga | id), na.action = na.omit)

summary(model.gamm$gam)
```

```
Family: gaussian
Link function: identity

Formula:
efwz ~ t2(ga, avgbc) + pcassets + momht + momage + nullparity +
    mommdd01 + momhbalt + x2handsmoke01 + catmomeduc

Parametric coefficients:
               Estimate Std. Error t value Pr(>|t|)
(Intercept)   -5.712352   0.418818 -13.639  < 2e-16 ***
pcassets       0.140877   0.021553   6.536 6.66e-11 ***
momht          0.029253   0.002630  11.124  < 2e-16 ***
momage         0.029964   0.004013   7.466 9.04e-14 ***
nullparity    -0.207267   0.037999  -5.454 5.05e-08 ***
mommdd01       0.355538   0.037750   9.418  < 2e-16 ***
momhbalt       0.022737   0.011303   2.012  0.04430 *
x2handsmoke01 -0.175114   0.054514  -3.212  0.00132 **
catmomeduc2    0.190238   0.041717   4.560 5.18e-06 ***
catmomeduc3    0.426016   0.054226   7.856 4.42e-15 ***
---
Signif. codes:  0 '***' 0.001 '**' 0.01 '*' 0.05 '.' 0.1 ' ' 1

Approximate significance of smooth terms:
               edf Ref.df     F p-value
t2(ga,avgbc) 9.402  9.402 89.39  <2e-16 ***
---
Signif. codes:  0 '***' 0.001 '**' 0.01 '*' 0.05 '.' 0.1 ' ' 1

R-sq.(adj) =  0.269
lmer.REML =  23376  Scale est. = 0.34937   n = 8825
```

```
# predict(model.gamm$gam, newdata =
# list(ga = 25, avgbc = bc.quant.75,
# hhid = 13001, pcassets = 0.12, momht
# = 152.34, momage = 25.6, nullparity =
# 1, mommdd01 = 1, momhbalt = 12.6,
# x2handsmoke01 = 1, catmomeduc = 1)) -
# predict(model.gamm$gam, newdata =
# list(ga = 25, avgbc = bc.quant.25,
# hhid = 13001, pcassets = 0.12, momht
# = 152.34, momage = 25.6, nullparity =
# 1, mommdd01 = 1, momhbalt = 12.6,
# x2handsmoke01 = 1, catmomeduc = 1))
# predict(model.gamm$gam, newdata =
# list(ga = 25, avgbc = bc.quant.75,
# hhid = 13001, pcassets = 0.12, momht
# = 152.34, momage = 25.6, nullparity =
# 1, mommdd01 = 1, momhbalt = 12.6,
# x2handsmoke01 = 1, catmomeduc = 1)) -
# predict(model.gamm$gam, newdata =
# list(ga = 25, avgbc = bc.quant.25,
# hhid = 13001, pcassets = 0.12, momht
# = 152.34, momage = 25.6, nullparity =
# 1, mommdd01 = 1, momhbalt = 12.6,
# x2handsmoke01 = 1, catmomeduc = 1))

# calc diffs and 95% CIs
beta = coef(model.gamm$gam)
V = vcov(model.gamm$gam)
# at 25 weeks
df_pred = data.frame(avgbc = c(bc.quant.75,
    bc.quant.25), ga = 25, hhid = 13001,
    pcassets = 0.12, momht = 152.34, momage = 25.6,
    nullparity = 1, mommdd01 = 1, momhbalt = 12.6,
    x2handsmoke01 = 1, catmomeduc = 1)
phi_mat <- predict(model.gamm$gam, type = "lpmatrix",
    newdata = df_pred)
dX <- beta[11:34] %*% phi_mat[1, 11:34] -
    beta[11:34] %*% phi_mat[2, 11:34]

vc <- V[c(11:34), c(11:34)]
a <- phi_mat[2, 11:34] - phi_mat[1, 11:34]
```

```
vX <- (t(a) %*% vc %*% a)

c(dX, dX - qnorm(0.99375) * sqrt(vX), dX +
    qnorm(0.99375) * sqrt(vX))
```

```
[1] -0.01843920 -0.09256287  0.05568447
```

```
tab7["25", c("efwz_bc", "efwz_bc_lb", "efwz_bc_ub")] <- c(dX,
    dX - qnorm(0.99375) * sqrt(vX), dX +
        qnorm(0.99375) * sqrt(vX))
```

EFWZ 33-weeks

```
# at 33 weeks
df_pred = data.frame(avgbc = c(bc.quant.75,
    bc.quant.25), ga = 33, hhid = 13001,
    pcassets = 0.12, momht = 152.34, momage = 25.6,
    nullparity = 1, mommdd01 = 1, momhbalt = 12.6,
    x2handsmoke01 = 1, catmomeduc = 1)
phi_mat <- predict(model.gamm$gam, type = "lpmatrix",
    newdata = df_pred)
dX <- beta[11:34] %*% phi_mat[1, 11:34] -
    beta[11:34] %*% phi_mat[2, 11:34]

vc <- V[c(11:34), c(11:34)]
a <- phi_mat[2, 11:34] - phi_mat[1, 11:34]
vX <- (t(a) %*% vc %*% a)

c(dX, dX - qnorm(0.99375) * sqrt(vX), dX +
    qnorm(0.99375) * sqrt(vX))
```

```
[1] -0.059485588 -0.125940657  0.006969482
```

```
tab7["33", c("efwz_bc", "efwz_bc_lb", "efwz_bc_ub")] <- c(dX,
    dX - qnorm(0.99375) * sqrt(vX), dX +
        qnorm(0.99375) * sqrt(vX))
```

EFWZ 40-weeks

```
# at Birth
df_pred = data.frame(avgbc = c(bc.quant.75,
    bc.quant.25), ga = 40, hhid = 13001,
    pcassets = 0.12, momht = 152.34, momage = 25.6,
    nullparity = 1, mommdd01 = 1, momhbalt = 12.6,
    x2handsmoke01 = 1, catmomeduc = 1)
phi_mat <- predict(model.gamm$gam, type = "lpmatrix",
    newdata = df_pred)
dX <- beta[c(11:34)] %*% phi_mat[1, c(11:34)] -
    beta[c(11:34)] %*% phi_mat[2, c(11:34)]

vc <- V[c(c(11:34)), c(c(11:34))]
a <- phi_mat[2, c(11:34)] - phi_mat[1, c(11:34)]
vX <- (t(a) %*% vc %*% a)

c(dX, dX - qnorm(0.99375) * sqrt(vX), dX +
    qnorm(0.99375) * sqrt(vX))
```

```
[1] -0.09813757 -0.16781366 -0.02846148
```

```
tab7["40", c("efwz_bc", "efwz_bc_lb", "efwz_bc_ub")] <- c(dX,
    dX - qnorm(0.99375) * sqrt(vX), dX +
        qnorm(0.99375) * sqrt(vX))
```

CO

FLZ 25-weeks

```
co.quant.75 <- quantile(hap.x$co, na.rm = T)[["75%"]]
co.quant.25 <- quantile(hap.x$co, na.rm = T)[["25%"]]

### flz
model.gamm = gamm4(flz ~ t2(ga, avgco) +
    pcassets + momht + momage + nullparity +
    mommdd01 + momhbalt + x2handsmoke01 +
    catmomeduc, data = hap4, random = ~(1 +
    ga | id), na.action = na.omit)

summary(model.gamm$gam)
```

```
Family: gaussian
Link function: identity

Formula:
flz ~ t2(ga, avgco) + pcassets + momht + momage + nullparity +
    mommdd01 + momhbalt + x2handsmoke01 + catmomeduc

Parametric coefficients:
               Estimate Std. Error t value Pr(>|t|)
(Intercept)   -3.226386   0.480553  -6.714 2.07e-11 ***
pcassets      -0.125139   0.025295  -4.947 7.74e-07 ***
momht          0.022431   0.003022   7.422 1.31e-13 ***
momage         0.019899   0.004589   4.336 1.48e-05 ***
nullparity     0.001426   0.043969   0.032 0.974133
mommdd01      -0.153324   0.043269  -3.544 0.000398 ***
momhbalt      -0.011861   0.012988  -0.913 0.361164
x2handsmoke01  0.178119   0.067289   2.647 0.008140 **
catmomeduc2   -0.144468   0.048565  -2.975 0.002944 **
catmomeduc3   -0.176435   0.063998  -2.757 0.005853 **
---
Signif. codes:  0 '***' 0.001 '**' 0.01 '*' 0.05 '.' 0.1 ' ' 1

Approximate significance of smooth terms:
             edf Ref.df     F  p-value
t2(ga,avgco) 4.8    4.8 4.221 0.000716 ***
---
Signif. codes:  0 '***' 0.001 '**' 0.01 '*' 0.05 '.' 0.1 ' ' 1

R-sq.(adj) =  0.0366
lmer.REML =  16849  Scale est. = 0.42658   n = 5985
```

```r
predict(model.gamm$gam, newdata = list(ga = 25,
    avgco = co.quant.75, hhid = 13001, pcassets = 0.12,
    momht = 152.34, momage = 25.6, nullparity = 1,
    mommdd01 = 1, momhbalt = 12.6, x2handsmoke01 = 1,
    catmomeduc = 1)) - predict(model.gamm$gam,
    newdata = list(ga = 25, avgco = co.quant.25,
        hhid = 13001, pcassets = 0.12, momht = 152.34,
        momage = 25.6, nullparity = 1, mommdd01 = 1,
        momhbalt = 12.6, x2handsmoke01 = 1,
        catmomeduc = 1))
```

```
         1
-0.01958802
```

```
predict(model.gamm$gam, newdata = list(ga = 25,
    avgco = co.quant.75, hhid = 13001, pcassets = 0.12,
    momht = 152.34, momage = 25.6, nullparity = 1,
    mommdd01 = 1, momhbalt = 12.6, x2handsmoke01 = 1,
    catmomeduc = 1)) - predict(model.gamm$gam,
    newdata = list(ga = 25, avgco = co.quant.25,
        hhid = 13001, pcassets = 0.12, momht = 152.34,
        momage = 25.6, nullparity = 1, mommdd01 = 1,
        momhbalt = 12.6, x2handsmoke01 = 1,
        catmomeduc = 1))
```

```
         1
-0.01958802
```

```
# calc diffs and 95% CIs
beta = coef(model.gamm$gam)
V = vcov(model.gamm$gam)
# at 25 weeks
df_pred = data.frame(avgco = c(co.quant.75,
    co.quant.25), ga = 25, hhid = 13001,
    pcassets = 0.12, momht = 152.34, momage = 25.6,
    nullparity = 1, mommdd01 = 1, momhbalt = 12.6,
    x2handsmoke01 = 1, catmomeduc = 1)
phi_mat <- predict(model.gamm$gam, type = "lpmatrix",
    newdata = df_pred)
dX <- beta[11:34] %*% phi_mat[1, 11:34] -
    beta[11:34] %*% phi_mat[2, 11:34]

vc <- V[c(11:34), c(11:34)]
a <- phi_mat[2, 11:34] - phi_mat[1, 11:34]
vX <- (t(a) %*% vc %*% a)

c(dX, dX - qnorm(0.99375) * sqrt(vX), dX +
    qnorm(0.99375) * sqrt(vX))
```

```
[1] -0.01958802 -0.05362903  0.01445300
```

```r
tab7["25", c("flz_co", "flz_co_lb", "flz_co_ub")] <- c(dX,
    dX - qnorm(0.99375) * sqrt(vX), dX +
        qnorm(0.99375) * sqrt(vX))
```

FLZ 33-weeks

```r
# at 33 weeks
df_pred = data.frame(avgco = c(co.quant.75,
    co.quant.25), ga = 33, hhid = 13001,
    pcassets = 0.12, momht = 152.34, momage = 25.6,
    nullparity = 1, mommdd01 = 1, momhbalt = 12.6,
    x2handsmoke01 = 1, catmomeduc = 1)
phi_mat <- predict(model.gamm$gam, type = "lpmatrix",
    newdata = df_pred)
dX <- beta[11:34] %*% phi_mat[1, 11:34] -
    beta[11:34] %*% phi_mat[2, 11:34]

vc <- V[c(11:34), c(11:34)]
a <- phi_mat[2, 11:34] - phi_mat[1, 11:34]
vX <- (t(a) %*% vc %*% a)

c(dX, dX - qnorm(0.99375) * sqrt(vX), dX +
    qnorm(0.99375) * sqrt(vX))
```

```
[1] -0.031447176 -0.065862534  0.002968182
```

```r
tab7["33", c("flz_co", "flz_co_lb", "flz_co_ub")] <- c(dX,
    dX - qnorm(0.99375) * sqrt(vX), dX +
        qnorm(0.99375) * sqrt(vX))
```

HCZ 25-weeks

```r
### hcz

model.gamm = gamm4(hcz ~ t2(ga, avgco) +
    pcassets + momht + momage + nullparity +
    mommdd01 + momhbalt + x2handsmoke01 +
    catmomeduc, data = hap4, random = ~(1 +
    ga | id), na.action = na.omit)
```

```r
summary(model.gamm$gam)
```

```

Family: gaussian
Link function: identity

Formula:
hcz ~ t2(ga, avgco) + pcassets + momht + momage + nullparity +
    mommdd01 + momhbalt + x2handsmoke01 + catmomeduc

Parametric coefficients:
               Estimate Std. Error t value Pr(>|t|)
(Intercept)   -6.070362   0.478211 -12.694  < 2e-16 ***
pcassets       0.121106   0.025216   4.803  1.6e-06 ***
momht          0.035401   0.003007  11.773  < 2e-16 ***
momage         0.040498   0.004566   8.869  < 2e-16 ***
nullparity     0.050543   0.043775   1.155  0.24830
mommdd01       0.025694   0.043127   0.596  0.55134
momhbalt      -0.011728   0.012930  -0.907  0.36441
x2handsmoke01 -0.008654   0.066896  -0.129  0.89707
catmomeduc2    0.102410   0.048289   2.121  0.03398 *
catmomeduc3    0.209091   0.063737   3.281  0.00104 **
---
Signif. codes:  0 '***' 0.001 '**' 0.01 '*' 0.05 '.' 0.1 ' ' 1

Approximate significance of smooth terms:
               edf Ref.df    F p-value
t2(ga,avgco) 7.967  7.967 8.64  <2e-16 ***
---
Signif. codes:  0 '***' 0.001 '**' 0.01 '*' 0.05 '.' 0.1 ' ' 1

R-sq.(adj) =  0.0865
lmer.REML =  16950  Scale est. = 0.3833    n = 5985
```

```r
predict(model.gamm$gam, newdata = list(ga = 25,
    avgco = co.quant.75, hhid = 13001, pcassets = 0.12,
    momht = 152.34, momage = 25.6, nullparity = 1,
    mommdd01 = 1, momhbalt = 12.6, x2handsmoke01 = 1,
    catmomeduc = 1)) - predict(model.gamm$gam,
    newdata = list(ga = 25, avgco = co.quant.25,
```

```
        hhid = 13001, pcassets = 0.12, momht = 152.34,
        momage = 25.6, nullparity = 1, mommdd01 = 1,
        momhbalt = 12.6, x2handsmoke01 = 1,
        catmomeduc = 1))
```

```
          1
-0.01804646
```

```
predict(model.gamm$gam, newdata = list(ga = 25,
    avgco = co.quant.75, hhid = 13001, pcassets = 0.12,
    momht = 152.34, momage = 25.6, nullparity = 1,
    mommdd01 = 1, momhbalt = 12.6, x2handsmoke01 = 1,
    catmomeduc = 1)) - predict(model.gamm$gam,
    newdata = list(ga = 25, avgco = co.quant.25,
        hhid = 13001, pcassets = 0.12, momht = 152.34,
        momage = 25.6, nullparity = 1, mommdd01 = 1,
        momhbalt = 12.6, x2handsmoke01 = 1,
        catmomeduc = 1))
```

```
          1
-0.01804646
```

```
# calc diffs and 95% CIs
beta = coef(model.gamm$gam)
V = vcov(model.gamm$gam)
# at 25 weeks
df_pred = data.frame(avgco = c(co.quant.75,
    co.quant.25), ga = 25, hhid = 13001,
    pcassets = 0.12, momht = 152.34, momage = 25.6,
    nullparity = 1, mommdd01 = 1, momhbalt = 12.6,
    x2handsmoke01 = 1, catmomeduc = 1)
phi_mat <- predict(model.gamm$gam, type = "lpmatrix",
    newdata = df_pred)
dX <- beta[11:34] %*% phi_mat[1, 11:34] -
    beta[11:34] %*% phi_mat[2, 11:34]

vc <- V[c(11:34), c(11:34)]
a <- phi_mat[2, 11:34] - phi_mat[1, 11:34]
vX <- (t(a) %*% vc %*% a)
```

```r
c(dX, dX - qnorm(0.99375) * sqrt(vX), dX +
    qnorm(0.99375) * sqrt(vX))
```

```
[1] -0.01804646 -0.05847064  0.02237772
```

```r
tab7["25", c("hcz_co", "hcz_co_lb", "hcz_co_ub")] <- c(dX,
    dX - qnorm(0.99375) * sqrt(vX), dX +
        qnorm(0.99375) * sqrt(vX))
```

HCZ 33-weeks

```r
# at 33 weeks
df_pred = data.frame(avgco = c(co.quant.75,
    co.quant.25), ga = 33, hhid = 13001,
    pcassets = 0.12, momht = 152.34, momage = 25.6,
    nullparity = 1, mommdd01 = 1, momhbalt = 12.6,
    x2handsmoke01 = 1, catmomeduc = 1)
phi_mat <- predict(model.gamm$gam, type = "lpmatrix",
    newdata = df_pred)
dX <- beta[11:34] %*% phi_mat[1, 11:34] -
    beta[11:34] %*% phi_mat[2, 11:34]

vc <- V[c(11:34), c(11:34)]
a <- phi_mat[2, 11:34] - phi_mat[1, 11:34]
vX <- (t(a) %*% vc %*% a)

c(dX, dX - qnorm(0.99375) * sqrt(vX), dX +
    qnorm(0.99375) * sqrt(vX))
```

```
[1]  0.006616763 -0.036605218  0.049838744
```

```r
tab7["33", c("hcz_co", "hcz_co_lb", "hcz_co_ub")] <- c(dX,
    dX - qnorm(0.99375) * sqrt(vX), dX +
        qnorm(0.99375) * sqrt(vX))
```

ACZ 25-weeks

```r
### acz

model.gamm = gamm4(acz ~ t2(ga, avgco) +
    pcassets + momht + momage + nullparity +
    mommdd01 + momhbalt + x2handsmoke01 +
    catmomeduc, data = hap4, random = ~(1 +
    ga | id), na.action = na.omit)

summary(model.gamm$gam)
```

```
Family: gaussian
Link function: identity

Formula:
acz ~ t2(ga, avgco) + pcassets + momht + momage + nullparity +
    mommdd01 + momhbalt + x2handsmoke01 + catmomeduc

Parametric coefficients:
               Estimate Std. Error t value Pr(>|t|)
(Intercept)   -3.632527   0.487416  -7.453 1.05e-13 ***
pcassets       0.211696   0.025774   8.214 2.61e-16 ***
momht          0.019851   0.003064   6.478 1.00e-10 ***
momage         0.030770   0.004654   6.611 4.15e-11 ***
nullparity    -0.156706   0.044651  -3.510 0.000452 ***
mommdd01       0.454467   0.043846  10.365  < 2e-16 ***
momhbalt      -0.009162   0.013193  -0.694 0.487439
x2handsmoke01 -0.262266   0.068809  -3.812 0.000140 ***
catmomeduc2    0.221848   0.049377   4.493 7.16e-06 ***
catmomeduc3    0.501965   0.065124   7.708 1.49e-14 ***
---
Signif. codes:  0 '***' 0.001 '**' 0.01 '*' 0.05 '.' 0.1 ' ' 1

Approximate significance of smooth terms:
               edf Ref.df     F  p-value
t2(ga,avgco) 6.349  6.349 6.185 6.61e-07 ***
---
Signif. codes:  0 '***' 0.001 '**' 0.01 '*' 0.05 '.' 0.1 ' ' 1

R-sq.(adj) =  0.102
lmer.REML =  17259  Scale est. = 0.44075   n = 5985
```

```r
predict(model.gamm$gam, newdata = list(ga = 25,
    avgco = co.quant.75, hhid = 13001, pcassets = 0.12,
    momht = 152.34, momage = 25.6, nullparity = 1,
    mommdd01 = 1, momhbalt = 12.6, x2handsmoke01 = 1,
    catmomeduc = 1)) - predict(model.gamm$gam,
    newdata = list(ga = 25, avgco = co.quant.25,
        hhid = 13001, pcassets = 0.12, momht = 152.34,
        momage = 25.6, nullparity = 1, mommdd01 = 1,
        momhbalt = 12.6, x2handsmoke01 = 1,
        catmomeduc = 1))
```

```
         1
0.01918811
```

```r
predict(model.gamm$gam, newdata = list(ga = 25,
    avgco = co.quant.75, hhid = 13001, pcassets = 0.12,
    momht = 152.34, momage = 25.6, nullparity = 1,
    mommdd01 = 1, momhbalt = 12.6, x2handsmoke01 = 1,
    catmomeduc = 1)) - predict(model.gamm$gam,
    newdata = list(ga = 25, avgco = co.quant.25,
        hhid = 13001, pcassets = 0.12, momht = 152.34,
        momage = 25.6, nullparity = 1, mommdd01 = 1,
        momhbalt = 12.6, x2handsmoke01 = 1,
        catmomeduc = 1))
```

```
         1
0.01918811
```

```r
# calc diffs and 95% CIs
beta = coef(model.gamm$gam)
V = vcov(model.gamm$gam)
# at 25 weeks
df_pred = data.frame(avgco = c(co.quant.75,
    co.quant.25), ga = 25, hhid = 13001,
    pcassets = 0.12, momht = 152.34, momage = 25.6,
    nullparity = 1, mommdd01 = 1, momhbalt = 12.6,
    x2handsmoke01 = 1, catmomeduc = 1)
phi_mat <- predict(model.gamm$gam, type = "lpmatrix",
    newdata = df_pred)
dX <- beta[11:34] %*% phi_mat[1, 11:34] -
```

```
    beta[11:34] %*% phi_mat[2, 11:34]

vc <- V[c(11:34), c(11:34)]
a <- phi_mat[2, 11:34] - phi_mat[1, 11:34]
vX <- (t(a) %*% vc %*% a)

c(dX, dX - qnorm(0.99375) * sqrt(vX), dX +
    qnorm(0.99375) * sqrt(vX))
```

```
[1]  0.01918811 -0.01770537  0.05608159
```

```
tab7["25", c("acz_co", "acz_co_lb", "acz_co_ub")] <- c(dX,
    dX - qnorm(0.99375) * sqrt(vX), dX +
        qnorm(0.99375) * sqrt(vX))
```

ACZ 33-weeks

```
# at 33 weeks
df_pred = data.frame(avgco = c(co.quant.75,
    co.quant.25), ga = 33, hhid = 13001,
    pcassets = 0.12, momht = 152.34, momage = 25.6,
    nullparity = 1, mommdd01 = 1, momhbalt = 12.6,
    x2handsmoke01 = 1, catmomeduc = 1)
phi_mat <- predict(model.gamm$gam, type = "lpmatrix",
    newdata = df_pred)
dX <- beta[11:34] %*% phi_mat[1, 11:34] -
    beta[11:34] %*% phi_mat[2, 11:34]

vc <- V[c(11:34), c(11:34)]
a <- phi_mat[2, 11:34] - phi_mat[1, 11:34]
vX <- (t(a) %*% vc %*% a)

c(dX, dX - qnorm(0.99375) * sqrt(vX), dX +
    qnorm(0.99375) * sqrt(vX))
```

```
[1]  0.027538990 -0.008681255  0.063759234
```

```
tab7["33", c("acz_co", "acz_co_lb", "acz_co_ub")] <- c(dX,
    dX - qnorm(0.99375) * sqrt(vX), dX +
        qnorm(0.99375) * sqrt(vX))
```

EFWZ 25-weeks

```
### efwz

model.gamm = gamm4(efwz ~ t2(ga, avgco) +
    pcassets + momht + momage + nullparity +
    mommdd01 + momhbalt + x2handsmoke01 +
    catmomeduc, data = hap4, random = ~(1 +
    ga | id), na.action = na.omit)

summary(model.gamm$gam)
```

```
Family: gaussian
Link function: identity

Formula:
efwz ~ t2(ga, avgco) + pcassets + momht + momage + nullparity +
    mommdd01 + momhbalt + x2handsmoke01 + catmomeduc

Parametric coefficients:
               Estimate Std. Error t value Pr(>|t|)
(Intercept)   -5.774709   0.413654 -13.960  < 2e-16 ***
pcassets       0.137206   0.021343   6.428 1.36e-10 ***
momht          0.029690   0.002603  11.406  < 2e-16 ***
momage         0.030458   0.003979   7.655 2.14e-14 ***
nullparity    -0.194628   0.037649  -5.170 2.40e-07 ***
mommdd01       0.359923   0.037523   9.592  < 2e-16 ***
momhbalt       0.021197   0.011177   1.896 0.057938 .
x2handsmoke01 -0.190782   0.054405  -3.507 0.000456 ***
catmomeduc2    0.191085   0.041329   4.624 3.83e-06 ***
catmomeduc3    0.428363   0.053835   7.957 1.98e-15 ***
---
Signif. codes:  0 '***' 0.001 '**' 0.01 '*' 0.05 '.' 0.1 ' ' 1

Approximate significance of smooth terms:
               edf Ref.df     F p-value
```

```
t2(ga,avgco) 9.213  9.213 132.8  <2e-16 ***
---
Signif. codes:  0 '***' 0.001 '**' 0.01 '*' 0.05 '.' 0.1 ' ' 1

R-sq.(adj) =  0.273
lmer.REML =  23526  Scale est. = 0.3462    n = 8885
```

```
# predict(model.gamm$gam, newdata =
# list(ga = 25, avgco = co.quant.75,
# hhid = 13001, pcassets = 0.12, momht
# = 152.34, momage = 25.6, nullparity =
# 1, mommdd01 = 1, momhbalt = 12.6,
# x2handsmoke01 = 1, catmomeduc = 1)) -
# predict(model.gamm$gam, newdata =
# list(ga = 25, avgco = co.quant.25,
# hhid = 13001, pcassets = 0.12, momht
# = 152.34, momage = 25.6, nullparity =
# 1, mommdd01 = 1, momhbalt = 12.6,
# x2handsmoke01 = 1, catmomeduc = 1))
# predict(model.gamm$gam, newdata =
# list(ga = 25, avgco = co.quant.75,
# hhid = 13001, pcassets = 0.12, momht
# = 152.34, momage = 25.6, nullparity =
# 1, mommdd01 = 1, momhbalt = 12.6,
# x2handsmoke01 = 1, catmomeduc = 1)) -
# predict(model.gamm$gam, newdata =
# list(ga = 25, avgco = co.quant.25,
# hhid = 13001, pcassets = 0.12, momht
# = 152.34, momage = 25.6, nullparity =
# 1, mommdd01 = 1, momhbalt = 12.6,
# x2handsmoke01 = 1, catmomeduc = 1))

# calc diffs and 95% CIs
beta = coef(model.gamm$gam)
V = vcov(model.gamm$gam)
# at 25 weeks
df_pred = data.frame(avgco = c(co.quant.75,
    co.quant.25), ga = 25, hhid = 13001,
    pcassets = 0.12, momht = 152.34, momage = 25.6,
    nullparity = 1, mommdd01 = 1, momhbalt = 12.6,
    x2handsmoke01 = 1, catmomeduc = 1)
phi_mat <- predict(model.gamm$gam, type = "lpmatrix",
```

```
    newdata = df_pred)
dX <- beta[11:34] %*% phi_mat[1, 11:34] -
    beta[11:34] %*% phi_mat[2, 11:34]

vc <- V[c(11:34), c(11:34)]
a <- phi_mat[2, 11:34] - phi_mat[1, 11:34]
vX <- (t(a) %*% vc %*% a)

c(dX, dX - qnorm(0.99375) * sqrt(vX), dX +
    qnorm(0.99375) * sqrt(vX))
```

```
[1] -0.02155277 -0.06044060  0.01733507
```

```
tab7["25", c("efwz_co", "efwz_co_lb", "efwz_co_ub")] <- c(dX,
    dX - qnorm(0.99375) * sqrt(vX), dX +
        qnorm(0.99375) * sqrt(vX))
```

EFWZ 33-weeks

```
# at 33 weeks
df_pred = data.frame(avgco = c(co.quant.75,
    co.quant.25), ga = 33, hhid = 13001,
    pcassets = 0.12, momht = 152.34, momage = 25.6,
    nullparity = 1, mommdd01 = 1, momhbalt = 12.6,
    x2handsmoke01 = 1, catmomeduc = 1)
phi_mat <- predict(model.gamm$gam, type = "lpmatrix",
    newdata = df_pred)
dX <- beta[11:34] %*% phi_mat[1, 11:34] -
    beta[11:34] %*% phi_mat[2, 11:34]

vc <- V[c(11:34), c(11:34)]
a <- phi_mat[2, 11:34] - phi_mat[1, 11:34]
vX <- (t(a) %*% vc %*% a)

c(dX, dX - qnorm(0.99375) * sqrt(vX), dX +
    qnorm(0.99375) * sqrt(vX))
```

```
[1]  0.0009507659 -0.0340151341  0.0359166658
```

```
tab7["33", c("efwz_co", "efwz_co_lb", "efwz_co_ub")] <- c(dX,
    dX - qnorm(0.99375) * sqrt(vX), dX +
        qnorm(0.99375) * sqrt(vX))
```

EFWZ 40-weeks

```
# at birth
df_pred = data.frame(avgco = c(co.quant.75,
    co.quant.25), ga = 40, hhid = 13001,
    pcassets = 0.12, momht = 152.34, momage = 25.6,
    nullparity = 1, mommdd01 = 1, momhbalt = 12.6,
    x2handsmoke01 = 1, catmomeduc = 1)
phi_mat <- predict(model.gamm$gam, type = "lpmatrix",
    newdata = df_pred)
dX <- beta[c(11:34)] %*% phi_mat[1, c(11:34)] -
    beta[c(11:34)] %*% phi_mat[2, c(11:34)]

vc <- V[c(c(11:34)), c(c(11:34))]
a <- phi_mat[2, c(11:34)] - phi_mat[1, c(11:34)]
vX <- (t(a) %*% vc %*% a)

c(dX, dX - qnorm(0.99375) * sqrt(vX), dX +
    qnorm(0.99375) * sqrt(vX))
```

```
[1] 0.035714407 0.004085839 0.067342976
```

```
tab7["40", c("efwz_co", "efwz_co_lb", "efwz_co_ub")] <- c(dX,
    dX - qnorm(0.99375) * sqrt(vX), dX +
        qnorm(0.99375) * sqrt(vX))
```

```
### what if you truncate / trim data to
### 97.5% of CO
quantile(sort(hap4$avgco), 0.975)
hap5 = hap4[hap4$avgco < quantile(sort(hap4$avgco),
    0.975), ]
hap5 = hap5[is.na(hap5$avgco) == F, ]
hap5$id <- hap5$hhid
hap5$catmomeduc = factor(hap5$catmomeduc)
```

```r
### flz
model.gamm = gamm4(flz ~ t2(ga, avgco) +
    pcassets + momht + momage + nullparity +
    mommdd01 + momhbalt + x2handsmoke01 +
    catmomeduc, data = hap5, random = ~(1 +
    ga | id), na.action = na.omit)

summary(model.gamm$gam)

predict(model.gamm$gam, newdata = list(ga = 25,
    avgco = co.quant.75, hhid = 13001, pcassets = 0.12,
    momht = 152.34, momage = 25.6, nullparity = 1,
    mommdd01 = 1, momhbalt = 12.6, x2handsmoke01 = 1,
    catmomeduc = 1)) - predict(model.gamm$gam,
    newdata = list(ga = 25, avgco = co.quant.25,
        hhid = 13001, pcassets = 0.12, momht = 152.34,
        momage = 25.6, nullparity = 1, mommdd01 = 1,
        momhbalt = 12.6, x2handsmoke01 = 1,
        catmomeduc = 1))
predict(model.gamm$gam, newdata = list(ga = 25,
    avgco = co.quant.75, hhid = 13001, pcassets = 0.12,
    momht = 152.34, momage = 25.6, nullparity = 1,
    mommdd01 = 1, momhbalt = 12.6, x2handsmoke01 = 1,
    catmomeduc = 1)) - predict(model.gamm$gam,
    newdata = list(ga = 25, avgco = co.quant.25,
        hhid = 13001, pcassets = 0.12, momht = 152.34,
        momage = 25.6, nullparity = 1, mommdd01 = 1,
        momhbalt = 12.6, x2handsmoke01 = 1,
        catmomeduc = 1))

# calc diffs and 95% CIs
beta = coef(model.gamm$gam)
V = vcov(model.gamm$gam)
# at 25 weeks
df_pred = data.frame(avgco = c(co.quant.75,
    co.quant.25), ga = 25, hhid = 13001,
    pcassets = 0.12, momht = 152.34, momage = 25.6,
    nullparity = 1, mommdd01 = 1, momhbalt = 12.6,
    x2handsmoke01 = 1, catmomeduc = 1)
phi_mat <- predict(model.gamm$gam, type = "lpmatrix",
```

```
    newdata = df_pred)
dX <- beta[11:34] %*% phi_mat[1, 11:34] -
    beta[11:34] %*% phi_mat[2, 11:34]

vc <- V[c(11:34), c(11:34)]
a <- phi_mat[2, 11:34] - phi_mat[1, 11:34]
vX <- (t(a) %*% vc %*% a)

c(dX, dX - qnorm(0.99375) * sqrt(vX), dX +
    qnorm(0.99375) * sqrt(vX))

tab7.trunc["25", c("flz_co", "flz_co_lb",
    "flz_co_ub")] <- c(dX, dX - qnorm(0.99375) *
    sqrt(vX), dX + qnorm(0.99375) * sqrt(vX))

# at 33 weeks
df_pred = data.frame(avgco = c(co.quant.75,
    co.quant.25), ga = 33, hhid = 13001,
    pcassets = 0.12, momht = 152.34, momage = 25.6,
    nullparity = 1, mommdd01 = 1, momhbalt = 12.6,
    x2handsmoke01 = 1, catmomeduc = 1)
phi_mat <- predict(model.gamm$gam, type = "lpmatrix",
    newdata = df_pred)
dX <- beta[11:34] %*% phi_mat[1, 11:34] -
    beta[11:34] %*% phi_mat[2, 11:34]

vc <- V[c(11:34), c(11:34)]
a <- phi_mat[2, 11:34] - phi_mat[1, 11:34]
vX <- (t(a) %*% vc %*% a)

c(dX, dX - qnorm(0.99375) * sqrt(vX), dX +
    qnorm(0.99375) * sqrt(vX))

tab7.trunc["33", c("flz_co", "flz_co_lb",
    "flz_co_ub")] <- c(dX, dX - qnorm(0.99375) *
    sqrt(vX), dX + qnorm(0.99375) * sqrt(vX))


### hcz

model.gamm = gamm4(hcz ~ t2(ga, avgco) +
```

```
    pcassets + momht + momage + nullparity +
    mommdd01 + momhbalt + x2handsmoke01 +
    catmomeduc, data = hap5, random = ~(1 +
    ga | id), na.action = na.omit)

summary(model.gamm$gam)

predict(model.gamm$gam, newdata = list(ga = 25,
    avgco = co.quant.75, hhid = 13001, pcassets = 0.12,
    momht = 152.34, momage = 25.6, nullparity = 1,
    mommdd01 = 1, momhbalt = 12.6, x2handsmoke01 = 1,
    catmomeduc = 1)) - predict(model.gamm$gam,
    newdata = list(ga = 25, avgco = co.quant.25,
        hhid = 13001, pcassets = 0.12, momht = 152.34,
        momage = 25.6, nullparity = 1, mommdd01 = 1,
        momhbalt = 12.6, x2handsmoke01 = 1,
        catmomeduc = 1))
predict(model.gamm$gam, newdata = list(ga = 25,
    avgco = co.quant.75, hhid = 13001, pcassets = 0.12,
    momht = 152.34, momage = 25.6, nullparity = 1,
    mommdd01 = 1, momhbalt = 12.6, x2handsmoke01 = 1,
    catmomeduc = 1)) - predict(model.gamm$gam,
    newdata = list(ga = 25, avgco = co.quant.25,
        hhid = 13001, pcassets = 0.12, momht = 152.34,
        momage = 25.6, nullparity = 1, mommdd01 = 1,
        momhbalt = 12.6, x2handsmoke01 = 1,
        catmomeduc = 1))

# calc diffs and 95% CIs
beta = coef(model.gamm$gam)
V = vcov(model.gamm$gam)
# at 25 weeks
df_pred = data.frame(avgco = c(co.quant.75,
    co.quant.25), ga = 25, hhid = 13001,
    pcassets = 0.12, momht = 152.34, momage = 25.6,
    nullparity = 1, mommdd01 = 1, momhbalt = 12.6,
    x2handsmoke01 = 1, catmomeduc = 1)
phi_mat <- predict(model.gamm$gam, type = "lpmatrix",
    newdata = df_pred)
dX <- beta[11:34] %*% phi_mat[1, 11:34] -
    beta[11:34] %*% phi_mat[2, 11:34]
```

```
vc <- V[c(11:34), c(11:34)]
a <- phi_mat[2, 11:34] - phi_mat[1, 11:34]
vX <- (t(a) %*% vc %*% a)

c(dX, dX - qnorm(0.99375) * sqrt(vX), dX +
    qnorm(0.99375) * sqrt(vX))

tab7.trunc["25", c("hcz_co", "hcz_co_lb",
    "hcz_co_ub")] <- c(dX, dX - qnorm(0.99375) *
    sqrt(vX), dX + qnorm(0.99375) * sqrt(vX))


# at 33 weeks
df_pred = data.frame(avgco = c(co.quant.75,
    co.quant.25), ga = 33, hhid = 13001,
    pcassets = 0.12, momht = 152.34, momage = 25.6,
    nullparity = 1, mommdd01 = 1, momhbalt = 12.6,
    x2handsmoke01 = 1, catmomeduc = 1)
phi_mat <- predict(model.gamm$gam, type = "lpmatrix",
    newdata = df_pred)
dX <- beta[11:34] %*% phi_mat[1, 11:34] -
    beta[11:34] %*% phi_mat[2, 11:34]

vc <- V[c(11:34), c(11:34)]
a <- phi_mat[2, 11:34] - phi_mat[1, 11:34]
vX <- (t(a) %*% vc %*% a)

c(dX, dX - qnorm(0.99375) * sqrt(vX), dX +
    qnorm(0.99375) * sqrt(vX))

tab7.trunc["33", c("hcz_co", "hcz_co_lb",
    "hcz_co_ub")] <- c(dX, dX - qnorm(0.99375) *
    sqrt(vX), dX + qnorm(0.99375) * sqrt(vX))


### acz

model.gamm = gamm4(acz ~ t2(ga, avgco) +
    pcassets + momht + momage + nullparity +
    mommdd01 + momhbalt + x2handsmoke01 +
    catmomeduc, data = hap5, random = ~(1 +
```

```
    ga | id), na.action = na.omit)

summary(model.gamm$gam)

predict(model.gamm$gam, newdata = list(ga = 25,
    avgco = co.quant.75, hhid = 13001, pcassets = 0.12,
    momht = 152.34, momage = 25.6, nullparity = 1,
    mommdd01 = 1, momhbalt = 12.6, x2handsmoke01 = 1,
    catmomeduc = 1)) - predict(model.gamm$gam,
    newdata = list(ga = 25, avgco = co.quant.25,
        hhid = 13001, pcassets = 0.12, momht = 152.34,
        momage = 25.6, nullparity = 1, mommdd01 = 1,
        momhbalt = 12.6, x2handsmoke01 = 1,
        catmomeduc = 1))
predict(model.gamm$gam, newdata = list(ga = 25,
    avgco = co.quant.75, hhid = 13001, pcassets = 0.12,
    momht = 152.34, momage = 25.6, nullparity = 1,
    mommdd01 = 1, momhbalt = 12.6, x2handsmoke01 = 1,
    catmomeduc = 1)) - predict(model.gamm$gam,
    newdata = list(ga = 25, avgco = co.quant.25,
        hhid = 13001, pcassets = 0.12, momht = 152.34,
        momage = 25.6, nullparity = 1, mommdd01 = 1,
        momhbalt = 12.6, x2handsmoke01 = 1,
        catmomeduc = 1))

# calc diffs and 95% CIs
beta = coef(model.gamm$gam)
V = vcov(model.gamm$gam)
# at 25 weeks
df_pred = data.frame(avgco = c(co.quant.75,
    co.quant.25), ga = 25, hhid = 13001,
    pcassets = 0.12, momht = 152.34, momage = 25.6,
    nullparity = 1, mommdd01 = 1, momhbalt = 12.6,
    x2handsmoke01 = 1, catmomeduc = 1)
phi_mat <- predict(model.gamm$gam, type = "lpmatrix",
    newdata = df_pred)
dX <- beta[11:34] %*% phi_mat[1, 11:34] -
    beta[11:34] %*% phi_mat[2, 11:34]

vc <- V[c(11:34), c(11:34)]
a <- phi_mat[2, 11:34] - phi_mat[1, 11:34]
```

```r
vX <- (t(a) %*% vc %*% a)

c(dX, dX - qnorm(0.99375) * sqrt(vX), dX +
    qnorm(0.99375) * sqrt(vX))

tab7.trunc["25", c("acz_co", "acz_co_lb",
    "acz_co_ub")] <- c(dX, dX - qnorm(0.99375) *
    sqrt(vX), dX + qnorm(0.99375) * sqrt(vX))


# at 33 weeks
df_pred = data.frame(avgco = c(co.quant.75,
    co.quant.25), ga = 33, hhid = 13001,
    pcassets = 0.12, momht = 152.34, momage = 25.6,
    nullparity = 1, mommdd01 = 1, momhbalt = 12.6,
    x2handsmoke01 = 1, catmomeduc = 1)
phi_mat <- predict(model.gamm$gam, type = "lpmatrix",
    newdata = df_pred)
dX <- beta[11:34] %*% phi_mat[1, 11:34] -
    beta[11:34] %*% phi_mat[2, 11:34]

vc <- V[c(11:34), c(11:34)]
a <- phi_mat[2, 11:34] - phi_mat[1, 11:34]
vX <- (t(a) %*% vc %*% a)

c(dX, dX - qnorm(0.99375) * sqrt(vX), dX +
    qnorm(0.99375) * sqrt(vX))

tab7.trunc["33", c("acz_co", "acz_co_lb",
    "acz_co_ub")] <- c(dX, dX - qnorm(0.99375) *
    sqrt(vX), dX + qnorm(0.99375) * sqrt(vX))

### efwz

model.gamm = gamm4(efwz ~ t2(ga, avgco) +
    pcassets + momht + momage + nullparity +
    mommdd01 + momhbalt + x2handsmoke01 +
    catmomeduc, data = hap5, random = ~(1 +
    ga | id), na.action = na.omit)

summary(model.gamm$gam)
```

```r
predict(model.gamm$gam, newdata = list(ga = 25,
    avgco = co.quant.75, hhid = 13001, pcassets = 0.12,
    momht = 152.34, momage = 25.6, nullparity = 1,
    mommdd01 = 1, momhbalt = 12.6, x2handsmoke01 = 1,
    catmomeduc = 1)) - predict(model.gamm$gam,
    newdata = list(ga = 25, avgco = co.quant.25,
        hhid = 13001, pcassets = 0.12, momht = 152.34,
        momage = 25.6, nullparity = 1, mommdd01 = 1,
        momhbalt = 12.6, x2handsmoke01 = 1,
        catmomeduc = 1))
predict(model.gamm$gam, newdata = list(ga = 25,
    avgco = co.quant.75, hhid = 13001, pcassets = 0.12,
    momht = 152.34, momage = 25.6, nullparity = 1,
    mommdd01 = 1, momhbalt = 12.6, x2handsmoke01 = 1,
    catmomeduc = 1)) - predict(model.gamm$gam,
    newdata = list(ga = 25, avgco = co.quant.25,
        hhid = 13001, pcassets = 0.12, momht = 152.34,
        momage = 25.6, nullparity = 1, mommdd01 = 1,
        momhbalt = 12.6, x2handsmoke01 = 1,
        catmomeduc = 1))

# calc diffs and 95% CIs
beta = coef(model.gamm$gam)
V = vcov(model.gamm$gam)
# at 25 weeks
df_pred = data.frame(avgco = c(co.quant.75,
    co.quant.25), ga = 25, hhid = 13001,
    pcassets = 0.12, momht = 152.34, momage = 25.6,
    nullparity = 1, mommdd01 = 1, momhbalt = 12.6,
    x2handsmoke01 = 1, catmomeduc = 1)
phi_mat <- predict(model.gamm$gam, type = "lpmatrix",
    newdata = df_pred)
dX <- beta[11:34] %*% phi_mat[1, 11:34] -
    beta[11:34] %*% phi_mat[2, 11:34]

vc <- V[c(11:34), c(11:34)]
a <- phi_mat[2, 11:34] - phi_mat[1, 11:34]
vX <- (t(a) %*% vc %*% a)

c(dX, dX - qnorm(0.99375) * sqrt(vX), dX +
    qnorm(0.99375) * sqrt(vX))
```

```r
tab7.trunc["25", c("efwz_co", "efwz_co_lb",
    "efwz_co_ub")] <- c(dX, dX - qnorm(0.99375) *
    sqrt(vX), dX + qnorm(0.99375) * sqrt(vX))


# at 33 weeks
df_pred = data.frame(avgco = c(co.quant.75,
    co.quant.25), ga = 33, hhid = 13001,
    pcassets = 0.12, momht = 152.34, momage = 25.6,
    nullparity = 1, mommdd01 = 1, momhbalt = 12.6,
    x2handsmoke01 = 1, catmomeduc = 1)
phi_mat <- predict(model.gamm$gam, type = "lpmatrix",
    newdata = df_pred)
dX <- beta[11:34] %*% phi_mat[1, 11:34] -
    beta[11:34] %*% phi_mat[2, 11:34]

vc <- V[c(11:34), c(11:34)]
a <- phi_mat[2, 11:34] - phi_mat[1, 11:34]
vX <- (t(a) %*% vc %*% a)

c(dX, dX - qnorm(0.99375) * sqrt(vX), dX +
    qnorm(0.99375) * sqrt(vX))


tab7.trunc["33", c("efwz_co", "efwz_co_lb",
    "efwz_co_ub")] <- c(dX, dX - qnorm(0.99375) *
    sqrt(vX), dX + qnorm(0.99375) * sqrt(vX))


### E-R analysis with lagged exposure
### rather than averaged exposure BL
### fetal outcomes paired with BL
### exposure P1 fetal outcomes paired
### with avg of BL and P1 exposure P2
### fetal outcomes paired with avg of
### P1 and P2 exposure Birth weight
### paired with with avg of P1 and P2
### exposure

### flz
model.gamm = gamm4(flz ~ t2(ga, lagpm) +
```

```
    pcassets + momht + momage + nullparity +
    mommdd01 + momhbalt + x2handsmoke01 +
    catmomeduc, data = hap4, random = ~(1 +
    ga | id), na.action = na.omit)

summary(model.gamm$gam)

predict(model.gamm$gam, newdata = list(ga = 25,
    lagpm = co.quant.75, hhid = 13001, pcassets = 0.12,
    momht = 152.34, momage = 25.6, nullparity = 1,
    mommdd01 = 1, momhbalt = 12.6, x2handsmoke01 = 1,
    catmomeduc = 1)) - predict(model.gamm$gam,
    newdata = list(ga = 25, lagpm = co.quant.25,
        hhid = 13001, pcassets = 0.12, momht = 152.34,
        momage = 25.6, nullparity = 1, mommdd01 = 1,
        momhbalt = 12.6, x2handsmoke01 = 1,
        catmomeduc = 1))
predict(model.gamm$gam, newdata = list(ga = 25,
    lagpm = co.quant.75, hhid = 13001, pcassets = 0.12,
    momht = 152.34, momage = 25.6, nullparity = 1,
    mommdd01 = 1, momhbalt = 12.6, x2handsmoke01 = 1,
    catmomeduc = 1)) - predict(model.gamm$gam,
    newdata = list(ga = 25, lagpm = co.quant.25,
        hhid = 13001, pcassets = 0.12, momht = 152.34,
        momage = 25.6, nullparity = 1, mommdd01 = 1,
        momhbalt = 12.6, x2handsmoke01 = 1,
        catmomeduc = 1))

# calc diffs and 95% CIs
beta = coef(model.gamm$gam)
V = vcov(model.gamm$gam)
# at 25 weeks
df_pred = data.frame(lagpm = c(co.quant.75,
    co.quant.25), ga = 25, hhid = 13001,
    pcassets = 0.12, momht = 152.34, momage = 25.6,
    nullparity = 1, mommdd01 = 1, momhbalt = 12.6,
    x2handsmoke01 = 1, catmomeduc = 1)
phi_mat <- predict(model.gamm$gam, type = "lpmatrix",
    newdata = df_pred)
dX <- beta[11:34] %*% phi_mat[1, 11:34] -
    beta[11:34] %*% phi_mat[2, 11:34]
```

```
vc <- V[c(11:34), c(11:34)]
a <- phi_mat[2, 11:34] - phi_mat[1, 11:34]
vX <- (t(a) %*% vc %*% a)

c(dX, dX - qnorm(0.99375) * sqrt(vX), dX +
    qnorm(0.99375) * sqrt(vX))

tab7.lag["25", c("flz_co", "flz_co_lb", "flz_co_ub")] <- c(dX,
    dX - qnorm(0.99375) * sqrt(vX), dX +
        qnorm(0.99375) * sqrt(vX))


# at 33 weeks
df_pred = data.frame(lagpm = c(co.quant.75,
    co.quant.25), ga = 33, hhid = 13001,
    pcassets = 0.12, momht = 152.34, momage = 25.6,
    nullparity = 1, mommdd01 = 1, momhbalt = 12.6,
    x2handsmoke01 = 1, catmomeduc = 1)
phi_mat <- predict(model.gamm$gam, type = "lpmatrix",
    newdata = df_pred)
dX <- beta[11:34] %*% phi_mat[1, 11:34] -
    beta[11:34] %*% phi_mat[2, 11:34]

vc <- V[c(11:34), c(11:34)]
a <- phi_mat[2, 11:34] - phi_mat[1, 11:34]
vX <- (t(a) %*% vc %*% a)

c(dX, dX - qnorm(0.99375) * sqrt(vX), dX +
    qnorm(0.99375) * sqrt(vX))

tab7.lag["33", c("flz_co", "flz_co_lb", "flz_co_ub")] <- c(dX,
    dX - qnorm(0.99375) * sqrt(vX), dX +
        qnorm(0.99375) * sqrt(vX))

### hcz

model.gamm = gamm4(hcz ~ t2(ga, lagpm) +
    pcassets + momht + momage + nullparity +
    mommdd01 + momhbalt + x2handsmoke01 +
    catmomeduc, data = hap4, random = ~(1 +
    ga | id), na.action = na.omit)
```

```r
summary(model.gamm$gam)

predict(model.gamm$gam, newdata = list(ga = 25,
    lagpm = co.quant.75, hhid = 13001, pcassets = 0.12,
    momht = 152.34, momage = 25.6, nullparity = 1,
    mommdd01 = 1, momhbalt = 12.6, x2handsmoke01 = 1,
    catmomeduc = 1)) - predict(model.gamm$gam,
    newdata = list(ga = 25, lagpm = co.quant.25,
        hhid = 13001, pcassets = 0.12, momht = 152.34,
        momage = 25.6, nullparity = 1, mommdd01 = 1,
        momhbalt = 12.6, x2handsmoke01 = 1,
        catmomeduc = 1))
predict(model.gamm$gam, newdata = list(ga = 25,
    lagpm = co.quant.75, hhid = 13001, pcassets = 0.12,
    momht = 152.34, momage = 25.6, nullparity = 1,
    mommdd01 = 1, momhbalt = 12.6, x2handsmoke01 = 1,
    catmomeduc = 1)) - predict(model.gamm$gam,
    newdata = list(ga = 25, lagpm = co.quant.25,
        hhid = 13001, pcassets = 0.12, momht = 152.34,
        momage = 25.6, nullparity = 1, mommdd01 = 1,
        momhbalt = 12.6, x2handsmoke01 = 1,
        catmomeduc = 1))

# calc diffs and 95% CIs
beta = coef(model.gamm$gam)
V = vcov(model.gamm$gam)
# at 25 weeks
df_pred = data.frame(lagpm = c(co.quant.75,
    co.quant.25), ga = 25, hhid = 13001,
    pcassets = 0.12, momht = 152.34, momage = 25.6,
    nullparity = 1, mommdd01 = 1, momhbalt = 12.6,
    x2handsmoke01 = 1, catmomeduc = 1)
phi_mat <- predict(model.gamm$gam, type = "lpmatrix",
    newdata = df_pred)
dX <- beta[11:34] %*% phi_mat[1, 11:34] -
    beta[11:34] %*% phi_mat[2, 11:34]

vc <- V[c(11:34), c(11:34)]
a <- phi_mat[2, 11:34] - phi_mat[1, 11:34]
vX <- (t(a) %*% vc %*% a)
```

```
c(dX, dX - qnorm(0.99375) * sqrt(vX), dX +
    qnorm(0.99375) * sqrt(vX))

tab7.lag["25", c("hcz_co", "hcz_co_lb", "hcz_co_ub")] <- c(dX,
    dX - qnorm(0.99375) * sqrt(vX), dX +
        qnorm(0.99375) * sqrt(vX))


# at 33 weeks
df_pred = data.frame(lagpm = c(co.quant.75,
    co.quant.25), ga = 33, hhid = 13001,
    pcassets = 0.12, momht = 152.34, momage = 25.6,
    nullparity = 1, mommdd01 = 1, momhbalt = 12.6,
    x2handsmoke01 = 1, catmomeduc = 1)
phi_mat <- predict(model.gamm$gam, type = "lpmatrix",
    newdata = df_pred)
dX <- beta[11:34] %*% phi_mat[1, 11:34] -
    beta[11:34] %*% phi_mat[2, 11:34]

vc <- V[c(11:34), c(11:34)]
a <- phi_mat[2, 11:34] - phi_mat[1, 11:34]
vX <- (t(a) %*% vc %*% a)

c(dX, dX - qnorm(0.99375) * sqrt(vX), dX +
    qnorm(0.99375) * sqrt(vX))

tab7.lag["33", c("hcz_co", "hcz_co_lb", "hcz_co_ub")] <- c(dX,
    dX - qnorm(0.99375) * sqrt(vX), dX +
        qnorm(0.99375) * sqrt(vX))


### acz

model.gamm = gamm4(acz ~ t2(ga, lagpm) +
    pcassets + momht + momage + nullparity +
    mommdd01 + momhbalt + x2handsmoke01 +
    catmomeduc, data = hap4, random = ~(1 +
    ga | id), na.action = na.omit)

summary(model.gamm$gam)
```

```
predict(model.gamm$gam, newdata = list(ga = 25,
    lagpm = co.quant.75, hhid = 13001, pcassets = 0.12,
    momht = 152.34, momage = 25.6, nullparity = 1,
    mommdd01 = 1, momhbalt = 12.6, x2handsmoke01 = 1,
    catmomeduc = 1)) - predict(model.gamm$gam,
    newdata = list(ga = 25, lagpm = co.quant.25,
        hhid = 13001, pcassets = 0.12, momht = 152.34,
        momage = 25.6, nullparity = 1, mommdd01 = 1,
        momhbalt = 12.6, x2handsmoke01 = 1,
        catmomeduc = 1))
predict(model.gamm$gam, newdata = list(ga = 25,
    lagpm = co.quant.75, hhid = 13001, pcassets = 0.12,
    momht = 152.34, momage = 25.6, nullparity = 1,
    mommdd01 = 1, momhbalt = 12.6, x2handsmoke01 = 1,
    catmomeduc = 1)) - predict(model.gamm$gam,
    newdata = list(ga = 25, lagpm = co.quant.25,
        hhid = 13001, pcassets = 0.12, momht = 152.34,
        momage = 25.6, nullparity = 1, mommdd01 = 1,
        momhbalt = 12.6, x2handsmoke01 = 1,
        catmomeduc = 1))

# calc diffs and 95% CIs
beta = coef(model.gamm$gam)
V = vcov(model.gamm$gam)
# at 25 weeks
df_pred = data.frame(lagpm = c(co.quant.75,
    co.quant.25), ga = 25, hhid = 13001,
    pcassets = 0.12, momht = 152.34, momage = 25.6,
    nullparity = 1, mommdd01 = 1, momhbalt = 12.6,
    x2handsmoke01 = 1, catmomeduc = 1)
phi_mat <- predict(model.gamm$gam, type = "lpmatrix",
    newdata = df_pred)
dX <- beta[11:34] %*% phi_mat[1, 11:34] -
    beta[11:34] %*% phi_mat[2, 11:34]

vc <- V[c(11:34), c(11:34)]
a <- phi_mat[2, 11:34] - phi_mat[1, 11:34]
vX <- (t(a) %*% vc %*% a)

c(dX, dX - qnorm(0.99375) * sqrt(vX), dX +
    qnorm(0.99375) * sqrt(vX))
```

```r
tab7.lag["25", c("acz_co", "acz_co_lb", "acz_co_ub")] <- c(dX,
    dX - qnorm(0.99375) * sqrt(vX), dX +
        qnorm(0.99375) * sqrt(vX))



# at 33 weeks
df_pred = data.frame(lagpm = c(co.quant.75,
    co.quant.25), ga = 33, hhid = 13001,
    pcassets = 0.12, momht = 152.34, momage = 25.6,
    nullparity = 1, mommdd01 = 1, momhbalt = 12.6,
    x2handsmoke01 = 1, catmomeduc = 1)
phi_mat <- predict(model.gamm$gam, type = "lpmatrix",
    newdata = df_pred)
dX <- beta[11:34] %*% phi_mat[1, 11:34] -
    beta[11:34] %*% phi_mat[2, 11:34]

vc <- V[c(11:34), c(11:34)]
a <- phi_mat[2, 11:34] - phi_mat[1, 11:34]
vX <- (t(a) %*% vc %*% a)

c(dX, dX - qnorm(0.99375) * sqrt(vX), dX +
    qnorm(0.99375) * sqrt(vX))

tab7.lag["33", c("acz_co", "acz_co_lb", "acz_co_ub")] <- c(dX,
    dX - qnorm(0.99375) * sqrt(vX), dX +
        qnorm(0.99375) * sqrt(vX))

### efwz

model.gamm = gamm4(efwz ~ t2(ga, lagpm) +
    pcassets + momht + momage + nullparity +
    mommdd01 + momhbalt + x2handsmoke01 +
    catmomeduc, data = hap4, random = ~(1 +
    ga | id), na.action = na.omit)

summary(model.gamm$gam)

predict(model.gamm$gam, newdata = list(ga = 25,
    lagpm = co.quant.75, hhid = 13001, pcassets = 0.12,
    momht = 152.34, momage = 25.6, nullparity = 1,
```

```
    mommdd01 = 1, momhbalt = 12.6, x2handsmoke01 = 1,
    catmomeduc = 1)) - predict(model.gamm$gam,
    newdata = list(ga = 25, lagpm = co.quant.25,
        hhid = 13001, pcassets = 0.12, momht = 152.34,
        momage = 25.6, nullparity = 1, mommdd01 = 1,
        momhbalt = 12.6, x2handsmoke01 = 1,
        catmomeduc = 1))
predict(model.gamm$gam, newdata = list(ga = 25,
    lagpm = co.quant.75, hhid = 13001, pcassets = 0.12,
    momht = 152.34, momage = 25.6, nullparity = 1,
    mommdd01 = 1, momhbalt = 12.6, x2handsmoke01 = 1,
    catmomeduc = 1)) - predict(model.gamm$gam,
    newdata = list(ga = 25, lagpm = co.quant.25,
        hhid = 13001, pcassets = 0.12, momht = 152.34,
        momage = 25.6, nullparity = 1, mommdd01 = 1,
        momhbalt = 12.6, x2handsmoke01 = 1,
        catmomeduc = 1))

# calc diffs and 95% CIs
beta = coef(model.gamm$gam)
V = vcov(model.gamm$gam)
# at 25 weeks
df_pred = data.frame(lagpm = c(co.quant.75,
    co.quant.25), ga = 25, hhid = 13001,
    pcassets = 0.12, momht = 152.34, momage = 25.6,
    nullparity = 1, mommdd01 = 1, momhbalt = 12.6,
    x2handsmoke01 = 1, catmomeduc = 1)
phi_mat <- predict(model.gamm$gam, type = "lpmatrix",
    newdata = df_pred)
dX <- beta[11:34] %*% phi_mat[1, 11:34] -
    beta[11:34] %*% phi_mat[2, 11:34]

vc <- V[c(11:34), c(11:34)]
a <- phi_mat[2, 11:34] - phi_mat[1, 11:34]
vX <- (t(a) %*% vc %*% a)

c(dX, dX - qnorm(0.99375) * sqrt(vX), dX +
    qnorm(0.99375) * sqrt(vX))

tab7.lag["25", c("efwz_co", "efwz_co_lb",
    "efwz_co_ub")] <- c(dX, dX - qnorm(0.99375) *
```

```
    sqrt(vX), dX + qnorm(0.99375) * sqrt(vX))


# at 33 weeks
df_pred = data.frame(lagpm = c(co.quant.75,
    co.quant.25), ga = 33, hhid = 13001,
    pcassets = 0.12, momht = 152.34, momage = 25.6,
    nullparity = 1, mommdd01 = 1, momhbalt = 12.6,
    x2handsmoke01 = 1, catmomeduc = 1)
phi_mat <- predict(model.gamm$gam, type = "lpmatrix",
    newdata = df_pred)
dX <- beta[11:34] %*% phi_mat[1, 11:34] -
    beta[11:34] %*% phi_mat[2, 11:34]

vc <- V[c(11:34), c(11:34)]
a <- phi_mat[2, 11:34] - phi_mat[1, 11:34]
vX <- (t(a) %*% vc %*% a)

c(dX, dX - qnorm(0.99375) * sqrt(vX), dX +
    qnorm(0.99375) * sqrt(vX))

tab7.lag["33", c("efwz_co", "efwz_co_lb",
    "efwz_co_ub")] <- c(dX, dX - qnorm(0.99375) *
    sqrt(vX), dX + qnorm(0.99375) * sqrt(vX))


# Need to create a variable called
# gabirth that has gestational age at
# birth for all rows of a participant
# After that we need to create a
# 'reverse' gestional age (revga) where
# revga = 0 at birth Can only use this
# for EFWZ estimate at birth
```

Statistical code to produce tables

Table-1

```
df <- fread("Fetal_growth_03-07-2022.csv")

df.m <- df %>%
    dplyr::filter(!is.na(hc) | !is.na(ac) |
        !is.na(fl) | !is.na(efw)) %>%
    dplyr::filter(visit == "P1" | visit ==
        "P2")

df.tab1 <- df %>%
    dplyr::filter(visit == "BL" & hhid %in%
        unique(df.m$hhid))


df.tab1$lpg <- ifelse(df.tab1$lpg == 1, "Intervention",
    "Control")

mean.ga <- round(mean(df.tab1$ga, na.rm = TRUE),
    1)
sd.ga <- round(sd(df.tab1$ga, na.rm = TRUE),
    1)
mean.ga.int <- round(mean(df.tab1$ga[df.tab1$lpg ==
    "Intervention"], na.rm = TRUE), 1)
mean.ga.cont <- round(mean(df.tab1$ga[df.tab1$lpg ==
    "Control"], na.rm = TRUE), 1)

sd.ga.int <- round(sd(df.tab1$ga[df.tab1$lpg ==
    "Intervention"], na.rm = TRUE), 1)
sd.ga.cont <- round(sd(df.tab1$ga[df.tab1$lpg ==
    "Control"], na.rm = TRUE), 1)

mean.momage <- round(mean(df.tab1$momage,
    na.rm = TRUE), 1)
sd.momage <- round(sd(df.tab1$momage, na.rm = TRUE),
    1)


for (vv in c("bankacct", "tv", "radio", "mobile",
```

```
    "momeduc", "nullparous", "preterm", "mommddcat",
    "fiescat", "x2handsmoke")) {
    df.tab1[[vv]] <- ifelse(df.tab1[[vv]] ==
        "", NA, df.tab1[[vv]])
}

df.tab1$fiescat <- factor(df.tab1$fiescat,
    levels = c("None (0)", "Mild (1-3)",
        "Moderate/Severe (4-8)"))

df.tab1$mommddcat <- factor(df.tab1$mommddcat,
    levels = c("Low", "Medium", "High"))

df.tab1$preterm <- ifelse(df.tab1$preterm ==
    "Yes", 1, ifelse(df.tab1$preterm == "No",
    0, NA))

tab1 <- doit4me::table1x(df.tab1, varlist = c("momage",
    "momht", "factor(ma1fe2)", "factor(bankacct)",
    "factor(tv)", "factor(radio)", "factor(mobile)",
    "momeducyr", "sleephouse", "pcassets",
    "ga", "factor(nullparous)", "factor(htn)",
    "factor(preterm)", "factor(mommddcat)",
    "factor(fiescat)", "factor(x2handsmoke)"),
    group = "lpg")
tab1 <- tab1[, c(2, 1)]

tab1 <- rowinsert(locations = which(rownames(tab1) %in%
    c("momage mean(SD)", "factor(bankacct) (1)",
        "ga mean(SD)", "factor(mommddcat) (Low)",
        "factor(fiescat) (None (0))")), rownm = c("Demographics",
    "Socioeconomic status indicators", "Pregnancy factors",
    "Minimum dietary diversity", "Household food insecurity"),
    tab = tab1)

rownames(tab1)[rownames(tab1) == "factor(fiescat)(None (0))"] <- "factor(fiescat) (None)"

tab1 <- tab1[str_detect(rownames(tab1), "\\b(0)\\b") ==
    "FALSE" & str_detect(rownames(tab1),
    "\\b(NA)\\b") == "FALSE" & str_detect(rownames(tab1),
    "factor\\(ma1fe2\\) \\(\\)") == "FALSE" &
```

```r
    str_detect(rownames(tab1), "factor\\(ma1fe2\\) \\(Female\\)") ==
        "FALSE", ]

rownames(tab1) <- c("Demographics", "Maternal age in years, mean (SD)",
    "Maternal height in cm, mean (SD)", "Fetal sex, males (%)",
    "Socioeconomic status indicators", "Bank account, n (%)",
    "Television, n (%)", "Radio, n (%)",
    "Cellular telephone, n (%)", "Maternal education, number of years (SD)",
    "Number of people living in the house, mean (SD)",
    "Socioeconomic wealth index, mean (SD)",
    "Pregnancy factors", "Gestational age, weeks (SD)",
    "Nulliparous, n (%)", "Hypertension during pregnancy, n (%)",
    "Pre-term birth, n (%)", "Minimum dietary diversity",
    "Low (<4), n (%)", "Medium (4-5), n (%)",
    "High (>5), n (%)", "Household food insecurity",
    "Food secure, n (%)", "Mild (1-3), n (%)",
    "Moderate or severe (4 - 8), n (%)",
    "Smoking in the household, n (%)")


indentlist <- which(!(rownames(tab1) %in%
    c("Demographics", "Socioeconomic status indicators",
        "Pregnancy factors", "Minimum dietary diversity",
        "Household food insecurity", "Smoking in the household, n (%)")))


tab1 <- tab1 %>%
    kable(caption = "Table 1.",
        col.names = c(paste0("Intervention (N = ",
            table(df.tab1$lpg)[2], ")"),
            paste0("Control (N = ", table(df.tab1$lpg)[1],
                ")"))) %>%
    kableExtra::kable_classic_2(latex_options = "HOLD_position") %>%
    add_indent(indentlist, level_of_indent = 1) %>%
    column_spec(1, bold = ifelse(rownames(tab1) %in%
        c("Demographics", "Socioeconomic status indicators",
            "Pregnancy factors", "Minimum dietary diversity",
            "Household food insecurity",
            "Smoking in the household, n (%)"),
        TRUE, FALSE))
```

```
tab1
```

Table 1. Baseline characteristics of participants with fetal growth measurement

	Intervention (N = 1396)	Control (N = 1376)
**Demographics**		
Maternal age in years, mean (SD)	25.5 (4.4)	25.6 (4.6)
Maternal height in cm, mean (SD)	152.4 (6.3)	152.2 (6.1)
Fetal sex, males (%)	712 (51.0%)	684 (49.7%)
**Socioeconomic status indicators**		
Bank account, n (%)	546 (39.1%)	462 (33.6%)
Television, n (%)	653 (46.8%)	625 (45.4%)
Radio, n (%)	686 (49.1%)	654 (47.5%)
Cellular telephone, n (%)	1,215 (87.0%)	1,187 (86.3%)
Maternal education, number of years (SD)	8.0 (3.7)	7.8 (3.5)
Number of people living in the house, mean (SD)	4.3 (2.1)	4.3 (2.0)
Socioeconomic wealth index, mean (SD)	0.0 (1.0)	0.2 (1.0)
**Pregnancy factors**		
Gestational age, weeks (SD)	14.7 (3.0)	14.4 (3.1)
Nulliparous, n (%)	526 (37.7%)	477 (34.7%)
Hypertension during pregnancy, n (%)	25 (1.8%)	28 (2.0%)
Pre-term birth, n (%)	73 (5.2%)	66 (4.8%)
**Minimum dietary diversity**		
Low (<4), n (%)	740 (53.0%)	756 (54.9%)
Medium (4-5), n (%)	459 (32.9%)	472 (34.3%)
High (>5), n (%)	196 (14.0%)	147 (10.7%)
**Household food insecurity**		
Food secure, n (%)	788 (56.4%)	695 (50.5%)
Mild (1–3), n (%)	383 (27.4%)	405 (29.4%)
Moderate or severe (4 – 8), n (%)	205 (14.7%)	254 (18.5%)
**Smoking in the household, n (%)**	99 (7.1%)	126 (9.2%)

Table-2

```
tab.s2 <- NULL
for (jj in c("hcz", "acz", "flz", "efwz",
    "afi", "fhr")) {
    p1.int <- paste0(sprintf("%3.2f", round(mean(df[[jj]][df$lpg ==
        1 & df$visit == "P1"], na.rm = TRUE),
        2)), " (", sprintf("%3.2f", round(sd(df[[jj]][df$lpg ==
        1 & df$visit == "P1"], na.rm = TRUE),
        2)), ")")

    p1.cont <- paste0(sprintf("%3.2f", round(mean(df[[jj]][df$lpg ==
        0 & df$visit == "P1"], na.rm = TRUE),
        2)), " (", sprintf("%3.2f", round(sd(df[[jj]][df$lpg ==
        0 & df$visit == "P1"], na.rm = TRUE),
        2)), ")")

    p1.pval <- round(t.test(df[[jj]][df$visit ==
        "P1"] ~ df$lpg[df$visit == "P1"])$p.value,
        3)

    p2.int <- paste0(sprintf("%3.2f", round(mean(df[[jj]][df$lpg ==
        1 & df$visit == "P2"], na.rm = TRUE),
        2)), " (", sprintf("%3.2f", round(sd(df[[jj]][df$lpg ==
        1 & df$visit == "P2"], na.rm = TRUE),
        2)), ")")

    p2.cont <- paste0(sprintf("%3.2f", round(mean(df[[jj]][df$lpg ==
        0 & df$visit == "P2"], na.rm = TRUE),
        2)), " (", sprintf("%3.2f", round(sd(df[[jj]][df$lpg ==
        0 & df$visit == "P2"], na.rm = TRUE),
        2)), ")")

    p2.pval <- round(t.test(df[[jj]][df$visit ==
        "P2"] ~ df$lpg[df$visit == "P2"])$p.value,
        3)

    tab.s2 <- rbind(tab.s2, c(p1.int, p1.cont,
        p1.pval, p2.int, p2.cont, p2.pval))

}
```

```r
tab.s2 <- tab.s2[c(1:4), c(1, 2, 4, 5)]
colnames(tab.s2) <- c("P1_intervention",
    "P1_control", "P2_intervention", "P2_control")
rownames(tab.s2) <- c("Head circumference",
    "Abdominal circumference", "Femur length",
    "Estimated fetal weight")

tab2 <- tab.s2 %>%
    kable(caption = "Table 2. ",
        col.names = c("Intervention", "Control",
            "Intervention", "Control")) %>%
    kableExtra::kable_classic_2(latex_options = "HOLD_position") %>%
    add_indent(1:4, level_of_indent = 1) %>%
    add_header_above(c("", P1 = 2, P2 = 2)) %>%
    add_header_above(c("", `Z-scores, mean (SD)` = 4))

tab2
```

Table 2. Means and standard deviations of fetal growth Z-scores by visit and intervention arm.

	Z-scores, mean (SD)			
	P1		P2	
	Intervention	Control	Intervention	Control
Head circumference	0.35 (1.12)	0.49 (1.09)	0.30 (1.19)	0.31 (1.20)
Abdominal circumference	0.46 (1.18)	0.49 (1.17)	0.36 (1.11)	0.34 (1.13)
Femur length	0.40 (1.16)	0.46 (1.11)	0.46 (1.06)	0.44 (1.11)
Estimated fetal weight	0.50 (1.24)	0.58 (1.16)	0.35 (1.07)	0.33 (1.11)

Statistical code to produce figures

Figure-1

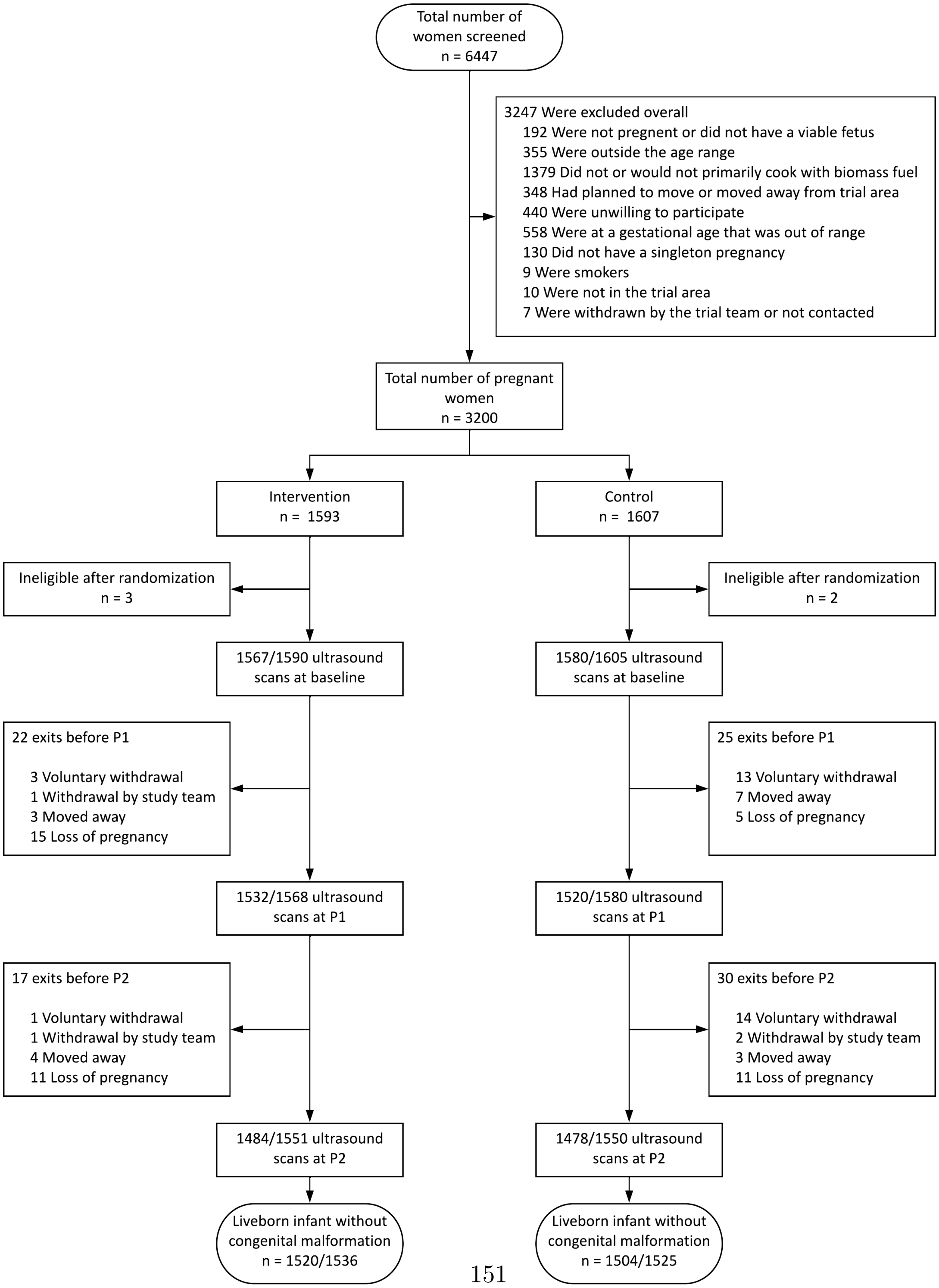

Figure-S1

```
df.s1 <- df %>%
    dplyr::select(hhid, visit, ga, usdate,
        lpg) %>%
    dplyr::filter(!is.na(ga)) %>%
    dplyr::mutate(ga = round(ga)) %>%
    dplyr::group_by(ga, visit) %>%
    dplyr::summarise(n = n())

df.s1$visit <- factor(df.s1$visit, levels = c("BL",
    "P1", "P2", "Birth"))

df.s2 <- df.s1 %>%
    dplyr::ungroup() %>%
    dplyr::group_by(ga) %>%
    dplyr::summarise(n = sum(n))

p.s1 <- ggplot() + geom_bar(aes(x = factor(df.s1$ga),
    y = df.s1$n, fill = factor(df.s1$visit)),
    position = "fill", stat = "identity") +
    scale_x_discrete("\n\nGestational age (weeks)",
        breaks = seq(9, 44, 1), labels = c("",
            "10", "", "12", "", "14", "",
            "16", "", "18", "", "20", "",
            "22", "", "24", "", "26", "",
            "28", "", "30", "", "32", "",
            "34", "", "36", "", "38", "",
            "40", "", "42", "", "44")) +
    geom_text(aes(x = factor(df.s2$ga), y = -Inf,
        label = df.s2$n), vjust = 5, size = 4) +
    scale_y_continuous("% of participants\n",
        labels = scales::percent, expand = c(0.005,
            0.005)) + scale_fill_manual(values = c("#ED0000FF",
    "#00468BFF", "#42B540FF", "#925E9FFF")) +
    theme_bw() + coord_cartesian(clip = "off") +
    theme(legend.title = element_blank(),
        panel.grid = element_blank(), axis.title = element_text(size = 18),
        axis.text = element_text(size = 14),
        legend.text = element_text(size = 18)) +
    annotate("text", x = -Inf, y = -Inf,
```

```
        label = "USG done", size = 6, hjust = 1,
        vjust = 3.4)



tiff("Figure_S1.tiff",
    units = "in", width = 15, height = 10,
    res = 100, compression = "lzw")
p.s1
invisible(dev.off())

setEPS()
postscript("Figure_S1.eps",
    width = 15, height = 10)
p.s1
invisible(dev.off())
```

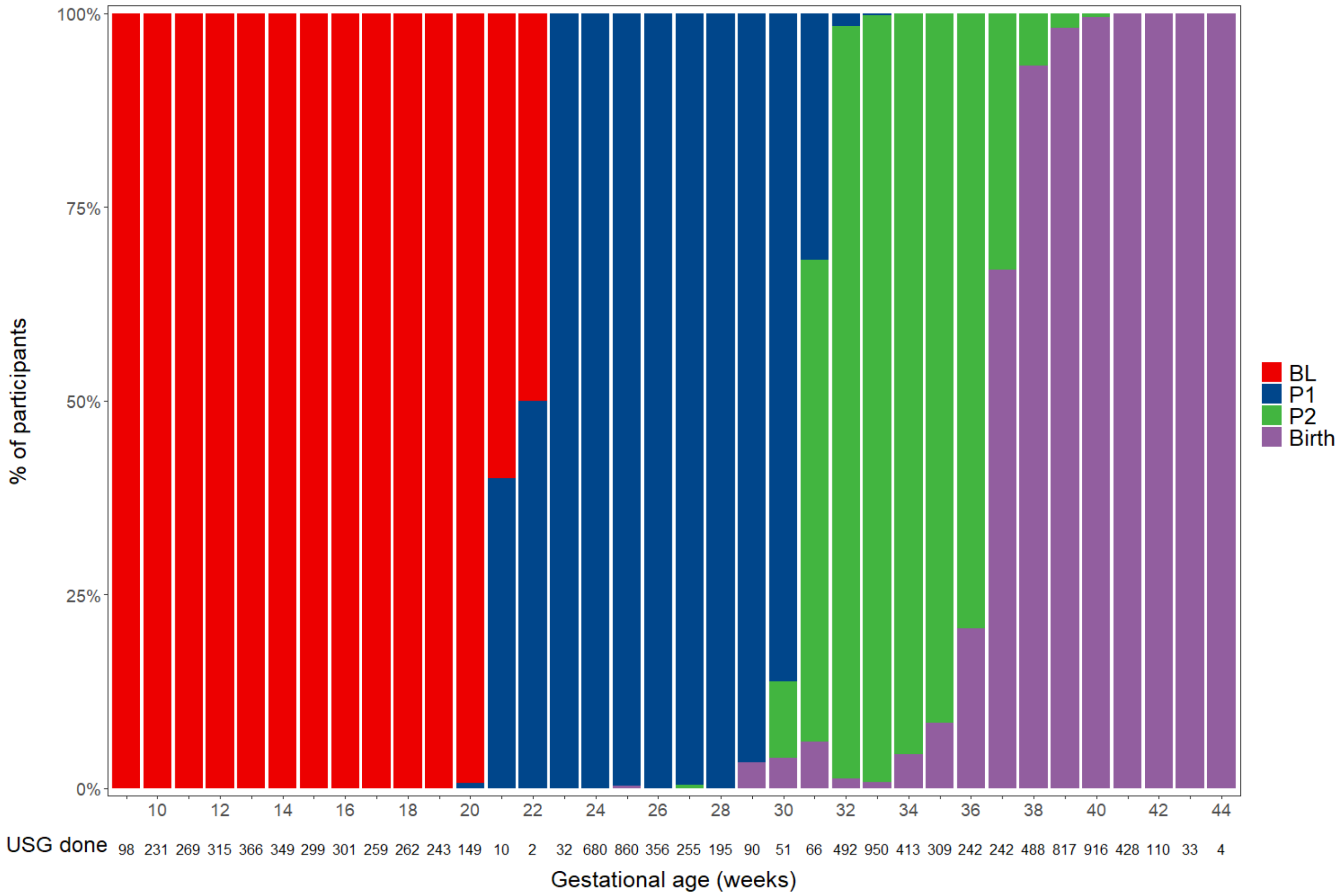

100%
75%
50%
25%
0%
% of participants
BL
P1
P2
Birth
10 12 14 16 18 20 22 24 26 28 30 32 34 36 38 40 42 44
USG done
98 231 269 315 366 349 299 301 259 262 243 149 10 2 32 680 860 356 255 195 90 51 66 492 950 413 309 242 242 488 817 916 428 110 33 4
Gestational age (weeks)

Figure-S1

Figure-2

```
GeomSplitViolin <- ggproto("GeomSplitViolin",
    GeomViolin, draw_group = function(self,
        data, ..., draw_quantiles = NULL) {
        # Original function by Jan
        # Gleixner (@jan-glx)
        # Adjustments by Wouter van der
        # Bijl (@Axeman)
        data <- transform(data, xminv = x -
            violinwidth * (x - xmin), xmaxv = x +
            violinwidth * (xmax - x))
        grp <- data[1, "group"]
        newdata <- plyr::arrange(transform(data,
            x = if (grp%%2 == 1)
                xminv else xmaxv), if (grp%%2 == 1)
            y else -y)
        newdata <- rbind(newdata[1, ], newdata,
            newdata[nrow(newdata), ], newdata[1,
                ])
        newdata[c(1, nrow(newdata) - 1, nrow(newdata)),
            "x"] <- round(newdata[1, "x"])
        if (length(draw_quantiles) > 0 &
            !scales::zero_range(range(data$y))) {
            stopifnot(all(draw_quantiles >=
                0), all(draw_quantiles <=
                1))
            quantiles <- create_quantile_segment_frame(data,
                draw_quantiles, split = TRUE,
                grp = grp)
            aesthetics <- data[rep(1, nrow(quantiles)),
                setdiff(names(data), c("x",
                  "y")), drop = FALSE]
            aesthetics$alpha <- rep(1, nrow(quantiles))
            aesthetics$size <- rep(1, nrow(quantiles))
            aesthetics$widths <- rep(1, nrow(quantiles))
            both <- cbind(quantiles, aesthetics)
            quantile_grob <- GeomPath$draw_panel(both,
                ...)
            ggplot2:::ggname("geom_split_violin",
                grid::grobTree(GeomPolygon$draw_panel(newdata,
```

```
                    ...), quantile_grob))
        } else {
            ggplot2:::ggname("geom_split_violin",
                GeomPolygon$draw_panel(newdata,
                  ...))
        }
    })

create_quantile_segment_frame <- function(data,
    draw_quantiles, split = FALSE, grp = NULL) {
    dens <- cumsum(data$density)/sum(data$density)
    ecdf <- stats::approxfun(dens, data$y)
    ys <- ecdf(draw_quantiles)
    violin.xminvs <- (stats::approxfun(data$y,
        data$xminv))(ys)
    violin.xmaxvs <- (stats::approxfun(data$y,
        data$xmaxv))(ys)
    violin.xs <- (stats::approxfun(data$y,
        data$x))(ys)
    if (grp%%2 == 0) {
        data.frame(x = ggplot2:::interleave(violin.xs,
            violin.xmaxvs), y = rep(ys, each = 2),
            group = rep(ys, each = 2))
    } else {
        data.frame(x = ggplot2:::interleave(violin.xminvs,
            violin.xs), y = rep(ys, each = 2),
            group = rep(ys, each = 2))
    }
}

geom_split_violin <- function(mapping = NULL,
    data = NULL, stat = "ydensity", position = "identity",
    ..., draw_quantiles = c(0.25, 0.5, 0.75),
    trim = TRUE, scale = "area", na.rm = FALSE,
    show.legend = NA, inherit.aes = TRUE) {
    layer(data = data, mapping = mapping,
        stat = stat, geom = GeomSplitViolin,
        position = position, show.legend = show.legend,
        inherit.aes = inherit.aes, params = list(trim = trim,
            scale = scale, draw_quantiles = draw_quantiles,
            na.rm = na.rm, ...))
```

```
}


# datain = df var = 'hc' x.group =
# 'lpg' y.group = 'gacat' ymin = 80
# ymax = 360 breaks = 40 title = 'HC
# (mm)' y.position = 'left' wd = 1.1
# birth = NULL


fun.violin = function(datain, var, x.group,
    y.group, ymin, ymax, breaks, xlab, wd,
    title, y.position, birth = NULL) {

    if (is.null(birth) == "FALSE") {
        datain <- datain %>%
            dplyr::filter(visit == "Birth" |
                !(is.na(gacat)))
        datain$gacat = ifelse(datain$visit ==
            "Birth", "Birth", datain$gacat)
    }

    datain <- datain %>%
        dplyr::filter(!is.na(gacat))

    p <- ggplot(data = datain, aes(y = !!sym(var),
        x = !!sym(y.group), fill = factor(!!sym(x.group)))) +
        geom_split_violin(width = wd, alpha = 1,
            color = "white", draw_quantiles = c(0.25,
                0.48, 0.49, 0.5, 0.51, 0.52,
                0.75)) + scale_fill_manual(values = c("#00468b",
        "#ed0000"), labels = c("Control",
        "Intervention")) + scale_color_manual(values = c("#00468b",
        "#ed0000"), labels = c("Control",
        "Intervention"), guide = "none") +
        scale_y_continuous(limits = c(ymin,
            ymax), breaks = seq(ymin, ymax,
            breaks), minor_breaks = seq(ymin,
            ymax, breaks/5), guide = "axis_minor",
            position = y.position) + ggtitle(title) +
        theme_bw() + theme(axis.text = element_text(size = 16),
```

```
        legend.text = element_text(size = 22),
        plot.title = element_text(hjust = 0.5,
            size = 22), ggh4x.axis.ticks.length.minor = rel(0.5),
        axis.ticks.length = unit(0.3, "cm"),
        axis.title = element_blank(), legend.title = element_blank(),
        legend.position = "none")

    return(p)
}


p1 = fun.violin(datain = df, var = "hc",
    x.group = "lpg", y.group = "gacat2",
    ymin = 80, ymax = 360, breaks = 40, title = "HC (mm)",
    y.position = "left", wd = 1.1)

p2 = fun.violin(datain = df, var = "ac",
    x.group = "lpg", y.group = "gacat2",
    ymin = 60, ymax = 380, breaks = 40, title = "AC (mm)",
    y.position = "right", wd = 1.1)

p3 = fun.violin(datain = df, var = "fl",
    x.group = "lpg", y.group = "gacat2",
    ymin = 10, ymax = 77, breaks = 10, title = "FL (mm)",
    y.position = "left", wd = 1.1)

p4 = fun.violin(datain = df[!(df$gacat %in%
    c("14-17", "18-21")), ], var = "efw",
    x.group = "lpg", y.group = "gacat", ymin = 250,
    ymax = 4000, breaks = 500, title = "EFW/BW (g)",
    y.position = "right", wd = 1.3)

legend = doit4me::getlegend(p1)
lheight <- sum(legend$height)


g1 <- arrangeGrob(rbind(ggplotGrob(p1), ggplotGrob(p3),
    size = "first"))

g2 <- arrangeGrob(rbind(ggplotGrob(p2), ggplotGrob(p4),
    size = "last"))
```

```r
g <- arrangeGrob(arrangeGrob(g1, g2, ncol = 2),
    legend, ncol = 1, heights = unit.c(unit.c(unit(1,
        "npc") - lheight, lheight)))

gg <- ggdraw(g) + annotate("text", x = 0.5,
    y = 0.07, size = 9, label = "Gestational age (weeks)")


tiff("Figure-2.tiff",
    units = "in", width = 15, height = 10,
    res = 100, compression = "lzw")
gg
invisible(dev.off())


setEPS()
postscript("Figure-2.eps",
    width = 15, height = 10)
gg
invisible(dev.off())
```

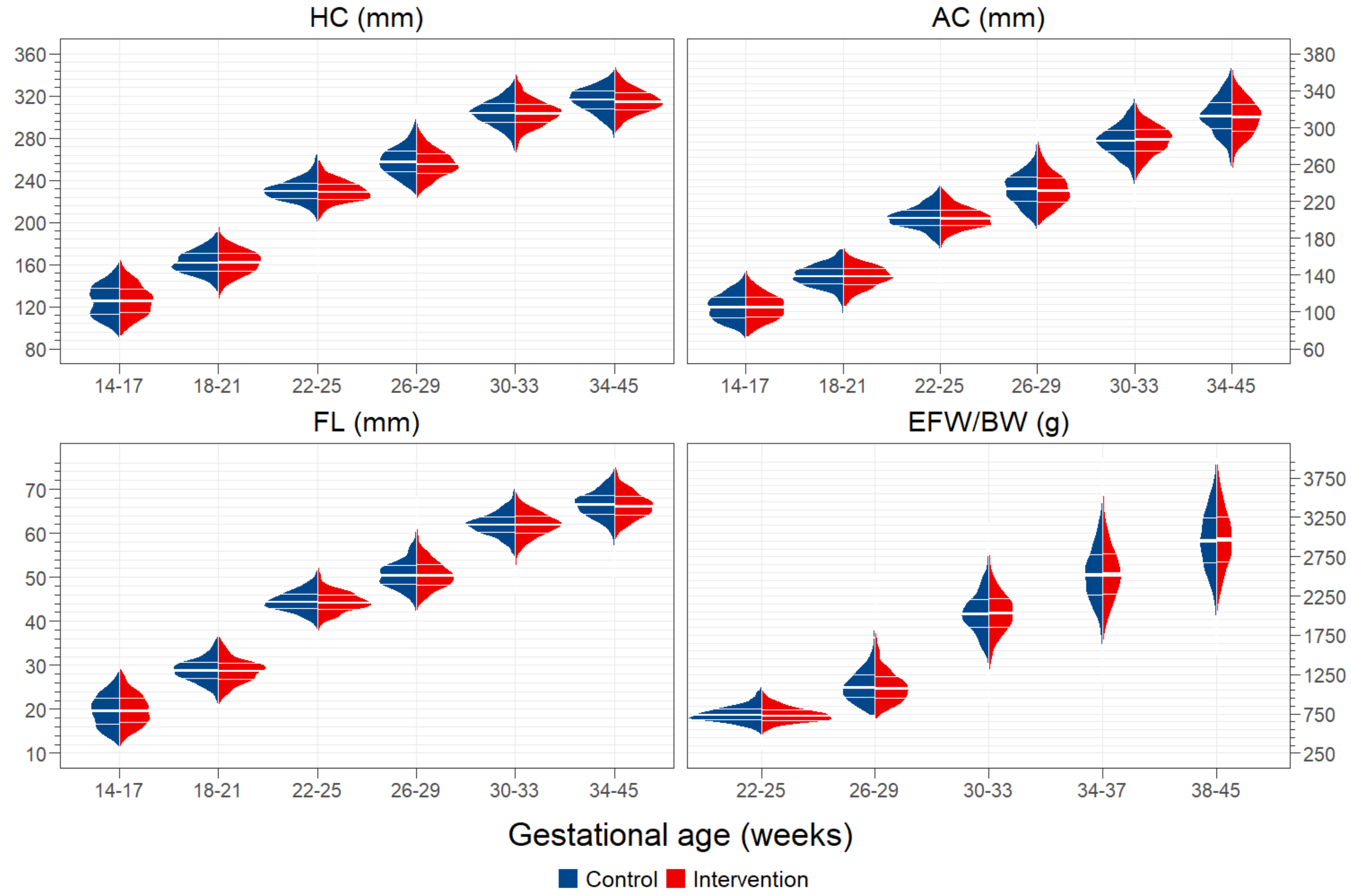

Figure 2. Teardrop plots for HC, AC, FL and EFW by intervention status.

Figure-S3

```
p2.hc.gua = fun.violin(datain = df[df$irc ==
    "Guatemala" & df$visit != "Birth", ],
    var = "hc", x.group = "lpg", y.group = "gacat2",
    ymin = 80, ymax = 360, breaks = 40, title = "Jalapa, Guatemala",
    y.position = "left", wd = 1.1)

p2.hc.ind = fun.violin(datain = df[df$irc ==
    "India" & df$visit != "Birth", ], var = "hc",
    x.group = "lpg", y.group = "gacat2",
    ymin = 80, ymax = 360, breaks = 40, title = "Tamil Nadu, India",
    y.position = "left", wd = 1.1)

p2.hc.per = fun.violin(datain = df[df$irc ==
    "Peru" & df$visit != "Birth", ], var = "hc",
    x.group = "lpg", y.group = "gacat2",
    ymin = 80, ymax = 360, breaks = 40, title = "Puno, Peru",
    y.position = "left", wd = 1.1)

p2.hc.rwa = fun.violin(datain = df[df$irc ==
    "Rwanda" & df$visit != "Birth", ], var = "hc",
    x.group = "lpg", y.group = "gacat2",
    ymin = 80, ymax = 360, breaks = 40, title = "Kayonza, Rwanda",
    y.position = "left", wd = 1.1)

legend = doit4me::getlegend(p2.hc.rwa)
lheight <- sum(legend$height)


g2.hc1 <- arrangeGrob(rbind(ggplotGrob(p2.hc.gua),
    ggplotGrob(p2.hc.per), size = "first"))


g2.hc2 <- arrangeGrob(rbind(ggplotGrob(p2.hc.ind),
    ggplotGrob(p2.hc.rwa), size = "last"))


g2.hc <- arrangeGrob(arrangeGrob(g2.hc1,
    g2.hc2, ncol = 2), legend, ncol = 1,
    heights = unit.c(unit.c(unit(1, "npc") -
        lheight, lheight)))
```

```r
gg2.hc <- ggdraw() + draw_plot(g2.hc, x = 0.05,
    y = 0, width = 0.95, height = 1) + annotate("text",
    x = 0.5, y = 0.07, size = 9, label = "Gestational age (weeks)") +
    annotate("text", x = 0.02, y = 0.5, size = 9,
        angle = 90, label = "Head circumference (mm)")

tiff("Figure-2_HC_S3.tiff",
    units = "in", width = 15, height = 10,
    res = 100, compression = "lzw")
gg2.hc
invisible(dev.off())

setEPS()
postscript("Figure-2_HC_S3.eps",
    width = 15, height = 10)
gg2.hc
invisible(dev.off())
```

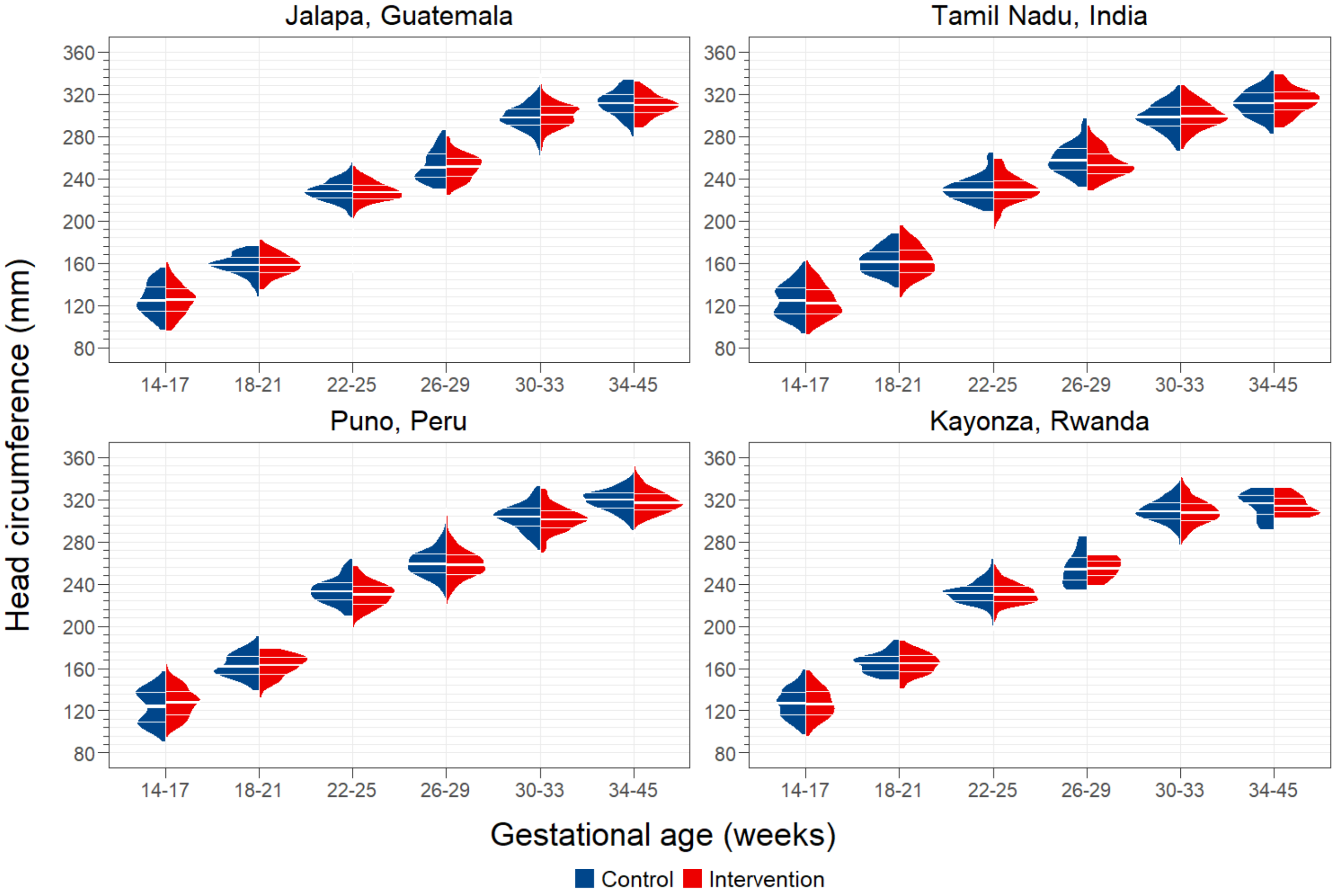

Jalapa, Guatemala
Tamil Nadu, India
Puno, Peru
Kayonza, Rwanda
360
320
280
240
200
160
120
80
14-17
18-21
22-25
26-29
30-33
34-45
Head circumference (mm)
Gestational age (weeks)
Control
Intervention

Figure-S3

Figure-S4

```
p2.ac.gua = fun.violin(datain = df[df$irc ==
    "Guatemala" & df$visit != "Birth", ],
    var = "ac", x.group = "lpg", y.group = "gacat2",
    ymin = 60, ymax = 360, breaks = 40, title = "Jalapa, Guatemala",
    y.position = "left", wd = 1.1)

p2.ac.ind = fun.violin(datain = df[df$irc ==
    "India" & df$visit != "Birth", ], var = "ac",
    x.group = "lpg", y.group = "gacat2",
    ymin = 60, ymax = 360, breaks = 40, title = "Tamil Nadu, India",
    y.position = "left", wd = 1.1)

p2.ac.per = fun.violin(datain = df[df$irc ==
    "Peru" & df$visit != "Birth", ], var = "ac",
    x.group = "lpg", y.group = "gacat2",
    ymin = 60, ymax = 360, breaks = 40, title = "Puno, Peru",
    y.position = "left", wd = 1.1)

p2.ac.rwa = fun.violin(datain = df[df$irc ==
    "Rwanda" & df$visit != "Birth", ], var = "ac",
    x.group = "lpg", y.group = "gacat2",
    ymin = 60, ymax = 360, breaks = 40, title = "Kayonza, Rwanda",
    y.position = "left", wd = 1.1)

legend = doit4me::getlegend(p2.ac.rwa)
lheight <- sum(legend$height)


g2.ac1 <- arrangeGrob(rbind(ggplotGrob(p2.ac.gua),
    ggplotGrob(p2.ac.per), size = "first"))

g2.ac2 <- arrangeGrob(rbind(ggplotGrob(p2.ac.ind),
    ggplotGrob(p2.ac.rwa), size = "last"))

g2.ac <- arrangeGrob(arrangeGrob(g2.ac1,
    g2.ac2, ncol = 2), legend, ncol = 1,
    heights = unit.c(unit.c(unit(1, "npc") -
        lheight, lheight)))

gg2.ac <- ggdraw() + draw_plot(g2.ac, x = 0.05,
```

```
    y = 0, width = 0.95, height = 1) + annotate("text",
    x = 0.5, y = 0.07, size = 9, label = "Gestational age (weeks)") +
    annotate("text", x = 0.02, y = 0.5, size = 9,
        angle = 90, label = "Abdominal circumference (mm)")

tiff("Figure-2_AC_S4.tiff",
    units = "in", width = 15, height = 10,
    res = 100, compression = "lzw")
gg2.ac
invisible(dev.off())

setEPS()
postscript("Figure-2_AC_S4.eps",
    width = 15, height = 10)
gg2.ac
invisible(dev.off())
```

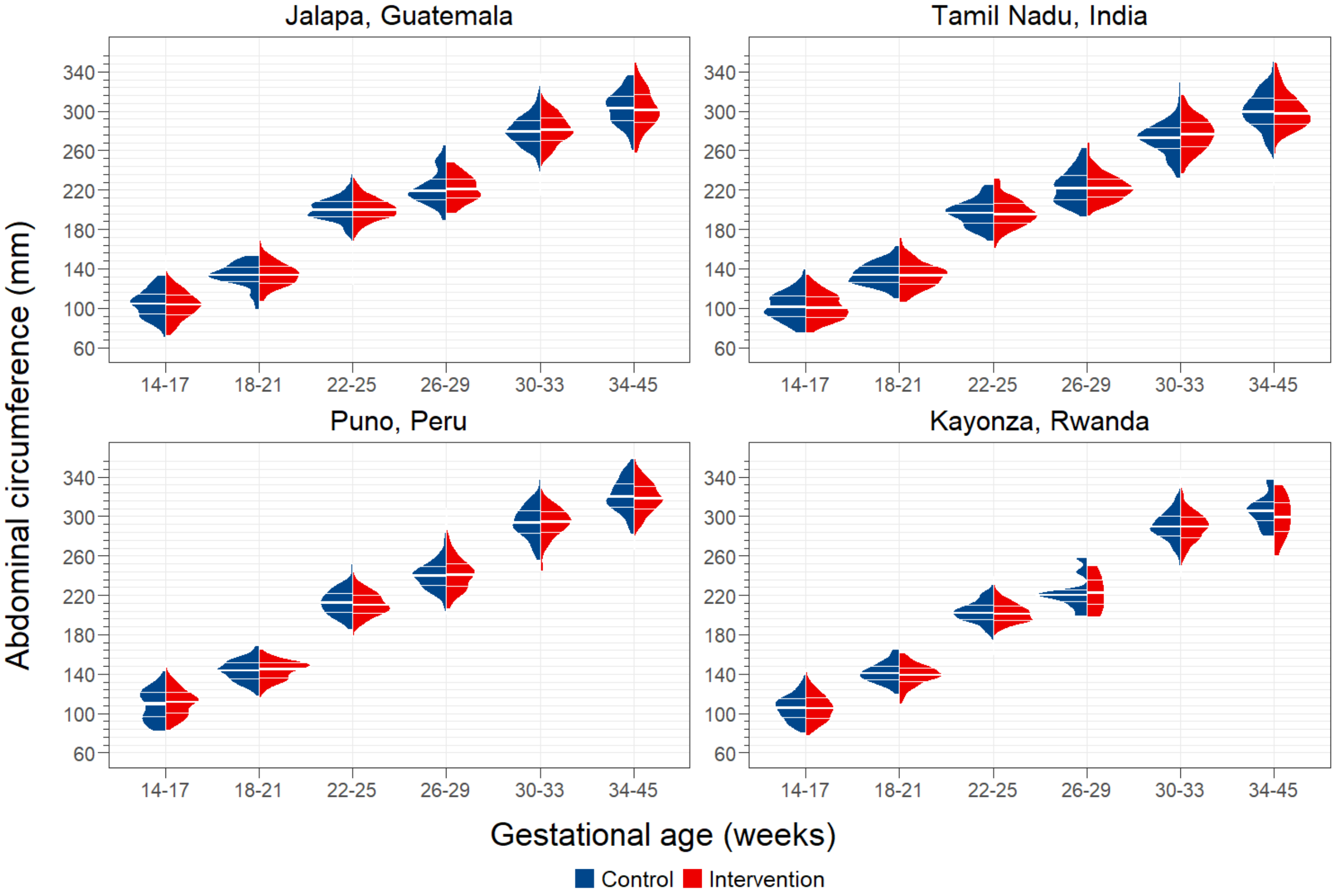

Jalapa, Guatemala
Tamil Nadu, India
Puno, Peru
Kayonza, Rwanda
340
300
260
220
180
140
100
60
14-17
18-21
22-25
26-29
30-33
34-45
Abdominal circumference (mm)
Gestational age (weeks)
Control
Intervention

Figure-S4

Figure-S5

```
p2.fl.gua = fun.violin(datain = df[df$irc ==
    "Guatemala" & df$visit != "Birth", ],
    var = "fl", x.group = "lpg", y.group = "gacat2",
    ymin = 10, ymax = 77, breaks = 10, title = "Jalapa, Guatemala",
    y.position = "left", wd = 1.1)

p2.fl.ind = fun.violin(datain = df[df$irc ==
    "India" & df$visit != "Birth", ], var = "fl",
    x.group = "lpg", y.group = "gacat2",
    ymin = 10, ymax = 77, breaks = 10, title = "Tamil Nadu, India",
    y.position = "left", wd = 1.1)

p2.fl.per = fun.violin(datain = df[df$irc ==
    "Peru" & df$visit != "Birth", ], var = "fl",
    x.group = "lpg", y.group = "gacat2",
    ymin = 10, ymax = 77, breaks = 10, title = "Puno, Peru",
    y.position = "left", wd = 1.1)

p2.fl.rwa = fun.violin(datain = df[df$irc ==
    "Rwanda" & df$visit != "Birth", ], var = "fl",
    x.group = "lpg", y.group = "gacat2",
    ymin = 10, ymax = 77, breaks = 10, title = "Kayonza, Rwanda",
    y.position = "left", wd = 1.1)

legend = doit4me::getlegend(p2.fl.rwa)
lheight <- sum(legend$height)


g2.fl1 <- arrangeGrob(rbind(ggplotGrob(p2.fl.gua),
    ggplotGrob(p2.fl.per), size = "first"))

g2.fl2 <- arrangeGrob(rbind(ggplotGrob(p2.fl.ind),
    ggplotGrob(p2.fl.rwa), size = "last"))

g2.fl <- arrangeGrob(arrangeGrob(g2.fl1,
    g2.fl2, ncol = 2), legend, ncol = 1,
    heights = unit.c(unit.c(unit(1, "npc") -
        lheight, lheight)))

gg2.fl <- ggdraw() + draw_plot(g2.fl, x = 0.05,
```

```
    y = 0, width = 0.95, height = 1) + annotate("text",
    x = 0.5, y = 0.07, size = 9, label = "Gestational age (weeks)") +
    annotate("text", x = 0.02, y = 0.5, size = 9,
        angle = 90, label = "Femur length (mm)")

tiff("Figure-2_FL_S5.tiff",
    units = "in", width = 15, height = 10,
    res = 100, compression = "lzw")
gg2.fl
invisible(dev.off())

setEPS()
postscript("Figure-2_FL_S5.eps",
    width = 15, height = 10)
gg2.fl
invisible(dev.off())
```

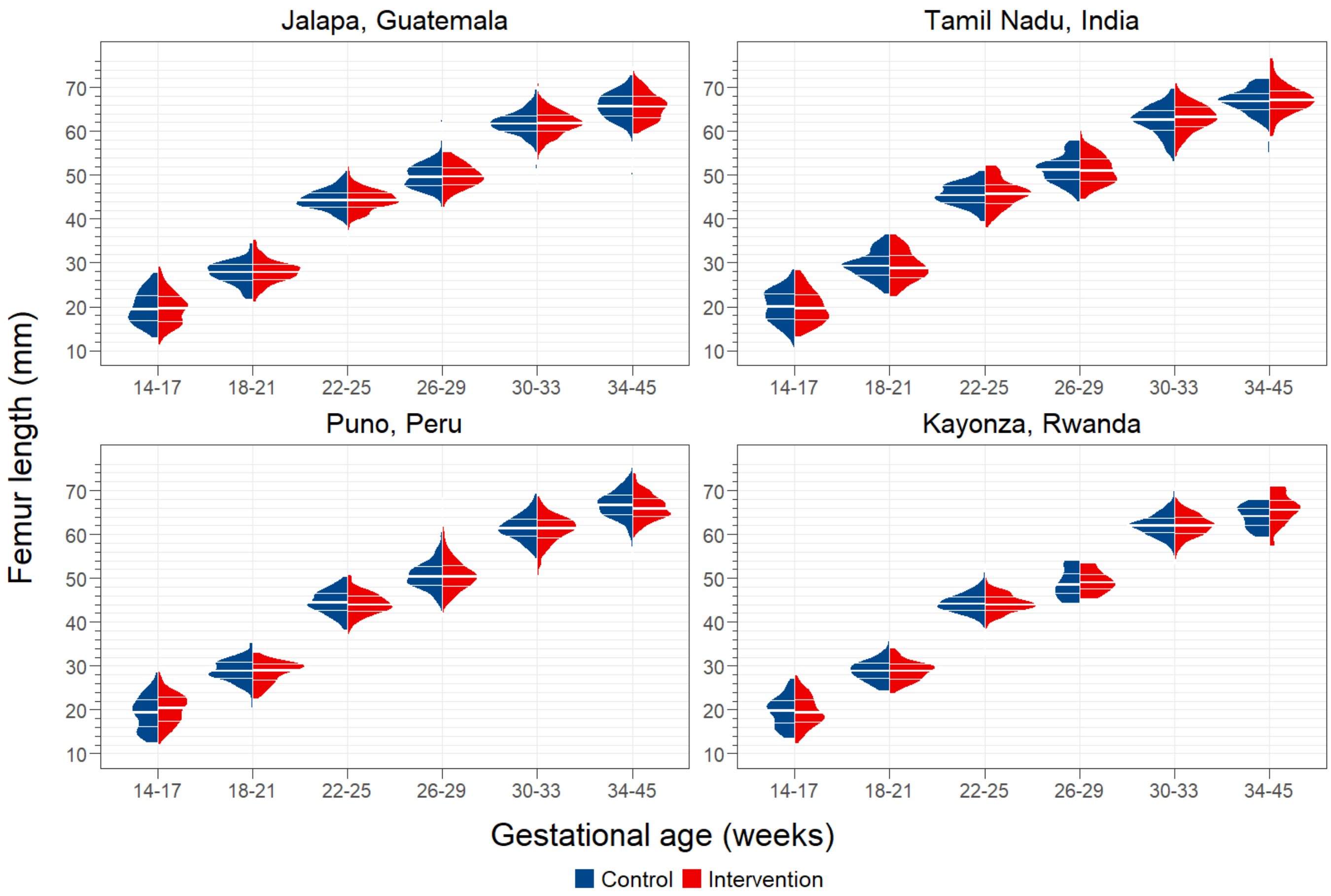
Jalapa, Guatemala
Tamil Nadu, India
Puno, Peru
Kayonza, Rwanda
Femur length (mm)
10
20
30
40
50
60
70
14-17
18-21
22-25
26-29
30-33
34-45
Gestational age (weeks)
Control
Intervention

Figure-S5

Figure-S6

```
p2.efw.gua = fun.violin(datain = df[df$irc ==
    "Guatemala" & (df$ga >= 24) | df$visit ==
    "Birth", ], var = "efw", x.group = "lpg",
    y.group = "gacat", ymin = 0, ymax = 4000,
    breaks = 500, title = "Jalapa, Guatemala",
    y.position = "left", wd = 1.1)

p2.efw.ind = fun.violin(datain = df[df$irc ==
    "India" & (df$ga >= 24) | df$visit ==
    "Birth", ], var = "efw", x.group = "lpg",
    y.group = "gacat", ymin = 0, ymax = 4000,
    breaks = 500, title = "Tamil Nadu, India",
    y.position = "left", wd = 1.1)

p2.efw.per = fun.violin(datain = df[df$irc ==
    "Peru" & (df$ga >= 24) | df$visit ==
    "Birth", ], var = "efw", x.group = "lpg",
    y.group = "gacat", ymin = 0, ymax = 4000,
    breaks = 500, title = "Puno, Peru", y.position = "left",
    wd = 1.1)

p2.efw.rwa = fun.violin(datain = df[df$irc ==
    "Rwanda" & (df$ga >= 24) | df$visit ==
    "Birth", ], var = "efw", x.group = "lpg",
    y.group = "gacat", ymin = 0, ymax = 4000,
    breaks = 500, title = "Kayonza, Rwanda",
    y.position = "left", wd = 1.1)

legend = doit4me::getlegend(p2.efw.rwa)
lheight <- sum(legend$height)


g2.efw1 <- arrangeGrob(rbind(ggplotGrob(p2.efw.gua),
    ggplotGrob(p2.efw.per), size = "first"))

g2.efw2 <- arrangeGrob(rbind(ggplotGrob(p2.efw.ind),
    ggplotGrob(p2.efw.rwa), size = "last"))

g2.efw <- arrangeGrob(arrangeGrob(g2.efw1,
    g2.efw2, ncol = 2), legend, ncol = 1,
```

```
    heights = unit.c(unit.c(unit(1, "npc") -
        lheight, lheight)))

gg2.efw <- ggdraw() + draw_plot(g2.efw, x = 0.05,
    y = 0, width = 0.95, height = 1) + annotate("text",
    x = 0.5, y = 0.07, size = 9, label = "Gestational age (weeks)") +
    annotate("text", x = 0.02, y = 0.5, size = 9,
        angle = 90, label = "Estimated fetal weight/ Birth weight (g)")

tiff("Figure-2_EFW_S6.tiff",
    units = "in", width = 15, height = 10,
    res = 100, compression = "lzw")
gg2.efw
invisible(dev.off())

setEPS()
postscript("Figure-2_EFW_S6.eps",
    width = 15, height = 10)
gg2.efw
invisible(dev.off())
```

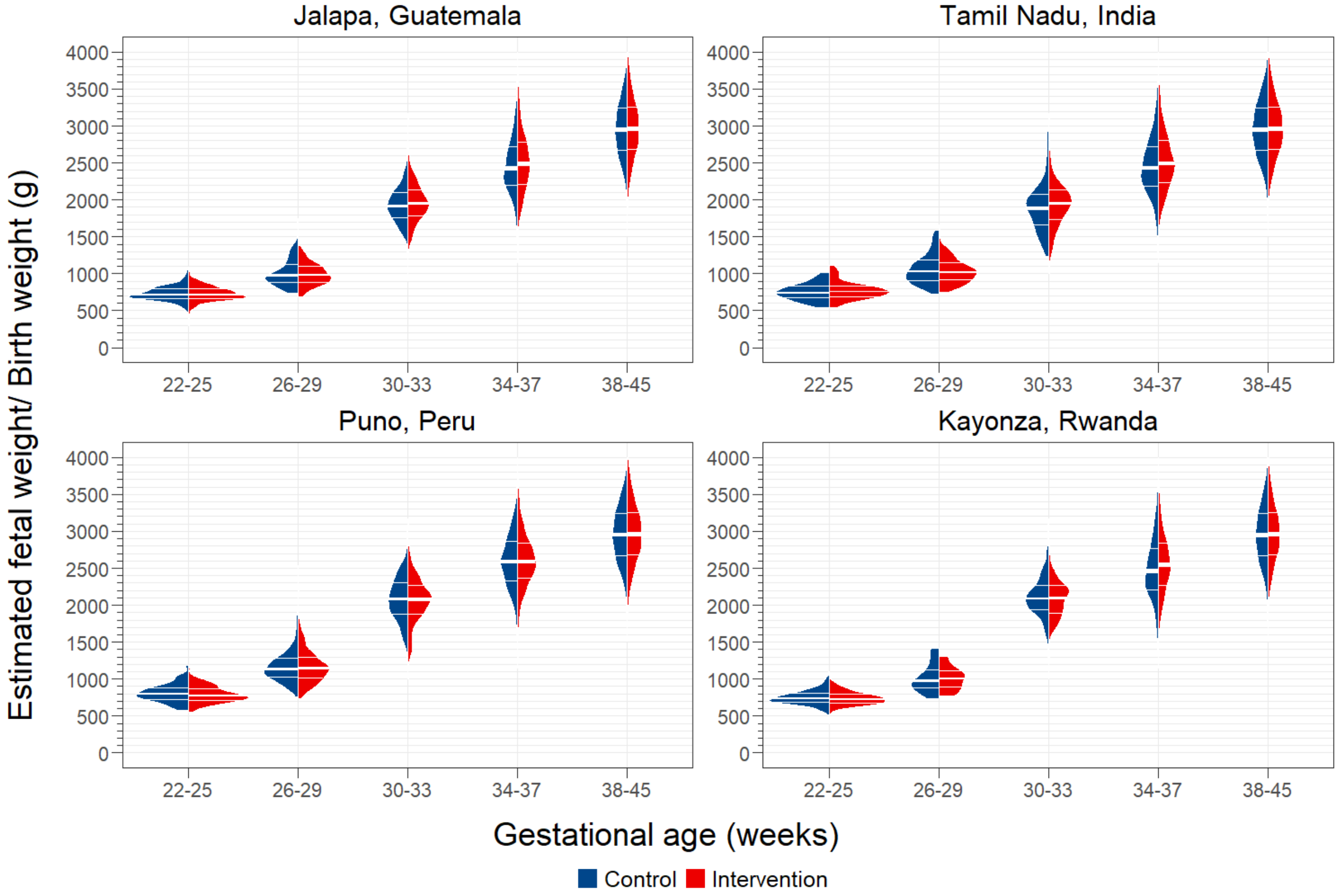
Jalapa, Guatemala
Tamil Nadu, India
Puno, Peru
Kayonza, Rwanda
Estimated fetal weight/ Birth weight (g)
0
500
1000
1500
2000
2500
3000
3500
4000
22-25
26-29
30-33
34-37
38-45
Gestational age (weeks)
Control
Intervention

Figure-S6

Figure-S2

```
# datain3 = df[df$irc == 'Guatemala' &
# df$visit != 'Birth',] var3 = 'hcz'
# title3 = 'Jalapa, Guatemala' x.group
# = 'gacat2' x.min3 = -0.65 x.max3 =
# 1.5 gap.3 = 0.5 birth = 'yes' brk =
# seq(-0.65, 1.5, 0.3)

fun.fig3 <- function(datain3 = df, var3 = "hcz",
    title3 = "HC (mm)", x.min3 = -0.2, x.max3 = 0.6,
    gap.3 = 0.1, birth = NULL, x.group = "gacat",
    brk = NULL) {

    # if(is.null(birth) == 'FALSE'){
    df.fig3 <- datain3 %>%
        dplyr::filter(!(is.na(!!sym(x.group))))
    # df.fig3$gacat =
    # ifelse(df.fig3$visit == 'Birth',
    # 'Birth', df.fig3$gacat) }

    # df.fig3 <- datain3 %>%
    # dplyr::filter(!is.na(gacat))

    df.fig3 <- df.fig3 %>%
        dplyr::group_by(!!sym(x.group), lpg) %>%
        dplyr::summarise(mean = mean(!!sym(var3),
            na.rm = TRUE), sd = sd(!!sym(var3),
            na.rm = TRUE), ub = Rmisc::CI(na.omit(!!sym(var3)))[[1]],
            lb = Rmisc::CI(na.omit(!!sym(var3)))[[3]]) %>%
        dplyr::mutate(lab.mean = sprintf("%3.2f",
            round(mean, 2)), lab.sd = sprintf("%3.2f",
            round(sd, 2)), lab = paste0(lab.mean,
            " ± ", lab.sd))

    for (vv in colnames(df.fig3)) {
        df.fig3[[vv]] <- ifelse(df.fig3[[vv]] %in%
            c("NaN", " NA", "NA", "NaN ±  NA"),
            NA, df.fig3[[vv]])
    }
```

```
if (x.group == "gacat") {
    df.fig3$gacat <- factor(df.fig3$gacat,
        levels = c("38-45", "34-37",
            "30-33", "26-29", "22-25",
            "18-21", "14-17"))
}

if (x.group == "gacat2") {
    df.fig3$gacat2 <- factor(df.fig3$gacat2,
        levels = c("34-45", "30-33",
            "26-29", "22-25", "18-21",
            "14-17"))
}

p3 <- ggplot(data = df.fig3) + geom_point(aes(x = mean,
    y = !!sym(x.group), color = factor(lpg)),
    shape = "diamond", size = 9, position = position_dodge(width = 0.8)) +
    geom_errorbar(aes(y = !!sym(x.group),
        xmin = lb, xmax = ub, color = factor(lpg)),
        position = position_dodge(width = 0.8),
        size = 1.5, width = 0) + geom_text(aes(y = !!sym(x.group),
    x = Inf, group = factor(lpg)), label = df.fig3$lab,
    position = position_dodge(width = 0.8),
    hjust = -0.1, size = 4.5)
if (is.null(brk) == "TRUE") {
    p3 <- p3 + scale_x_continuous(limits = c(x.min3,
        x.max3), breaks = seq(x.min3,
        x.max3, gap.3), minor_breaks = seq(x.min3,
        x.max3, gap.3/10), guide = "axis_minor")
} else {
    p3 <- p3 + scale_x_continuous(limits = c(x.min3,
        x.max3), breaks = brk, minor_breaks = seq(x.min3,
        x.max3, gap.3/10), guide = "axis_minor")
}

p3 <- p3 + scale_color_manual(values = c("#00468b",
    "#ed0000"), labels = c("Control",
    "Intervention")) + ggtitle(title3) +
    theme_bw() + theme(axis.text = element_text(size = 16),
    legend.text = element_text(size = 22),
    plot.title = element_text(hjust = 0.5,
```

```
            size = 22), panel.grid.minor = element_blank(),
        axis.ticks.length = unit(0.3, "cm"),
        ggh4x.axis.ticks.length.minor = rel(0.5),
        axis.title = element_blank(), legend.title = element_blank(),
        legend.position = "none", plot.margin = unit(c(1,
            6, 1, 1), "lines")) + coord_cartesian(clip = "off")
    return(p3)
}

p3.hc <- fun.fig3(datain3 = df, var3 = "hcz",
    title3 = "HC", x.group = "gacat2", x.min3 = -1,
    x.max3 = 1, gap.3 = 0.2, birth = "yes")

p3.ac <- fun.fig3(datain3 = df, var3 = "acz",
    title3 = "AC", x.group = "gacat2", x.min3 = -1,
    x.max3 = 1, gap.3 = 0.2, birth = "yes")

p3.fl <- fun.fig3(datain3 = df, var3 = "flz",
    title3 = "FL", x.group = "gacat2", x.min3 = -1,
    x.max3 = 1, gap.3 = 0.2, birth = "yes")

# df.fig3 <- df[(df$ga >= 24 & df$ga
# <=38) | df$visit == 'Birth',]

p3.efw <- fun.fig3(datain3 = df, var3 = "efwz",
    title3 = "EFW/BW", x.group = "gacat",
    x.min3 = -1, x.max3 = 1, gap.3 = 0.2,
    birth = "yes")

legend.p3 = doit4me::getlegend(p3.efw)
lheight.p3 <- sum(legend.p3$height)


g3.a <- arrangeGrob(rbind(ggplotGrob(p3.hc),
    ggplotGrob(p3.fl), size = "first"))

g3.b <- arrangeGrob(rbind(ggplotGrob(p3.ac),
    ggplotGrob(p3.efw), size = "last"))

g3 <- arrangeGrob(g3.a, g3.b, ncol = 2)
```

```
gg3 <- ggdraw() + draw_plot(g3, x = 0.04,
    y = 0.1, width = 0.95, height = 0.9) +
    draw_plot(legend.p3, x = 0, y = 0.03,
        width = 0.95, height = 0.05) + annotate("text",
    x = 0.5, y = 0.08, size = 9, label = "Z-scores") +
    annotate("text", x = 0.02, y = 0.5, size = 9,
        angle = 90, label = "Gestational age (weeks)") +
    annotate("text", x = 0.48, y = 0.96,
        size = 6, label = "Mean ± SD") +
    annotate("text", x = 0.955, y = 0.96,
        size = 6, label = "Mean ± SD") +
    annotate("text", x = 0.48, y = 0.515,
        size = 6, label = "Mean ± SD") +
    annotate("text", x = 0.955, y = 0.515,
        size = 6, label = "Mean ± SD")

tiff("Figure-S2.tiff",
    units = "in", width = 15, height = 10,
    res = 100, compression = "lzw")
gg3
invisible(dev.off())

setEPS()
postscript("Figure-S2.eps",
    width = 15, height = 10)
gg3
invisible(dev.off())
```

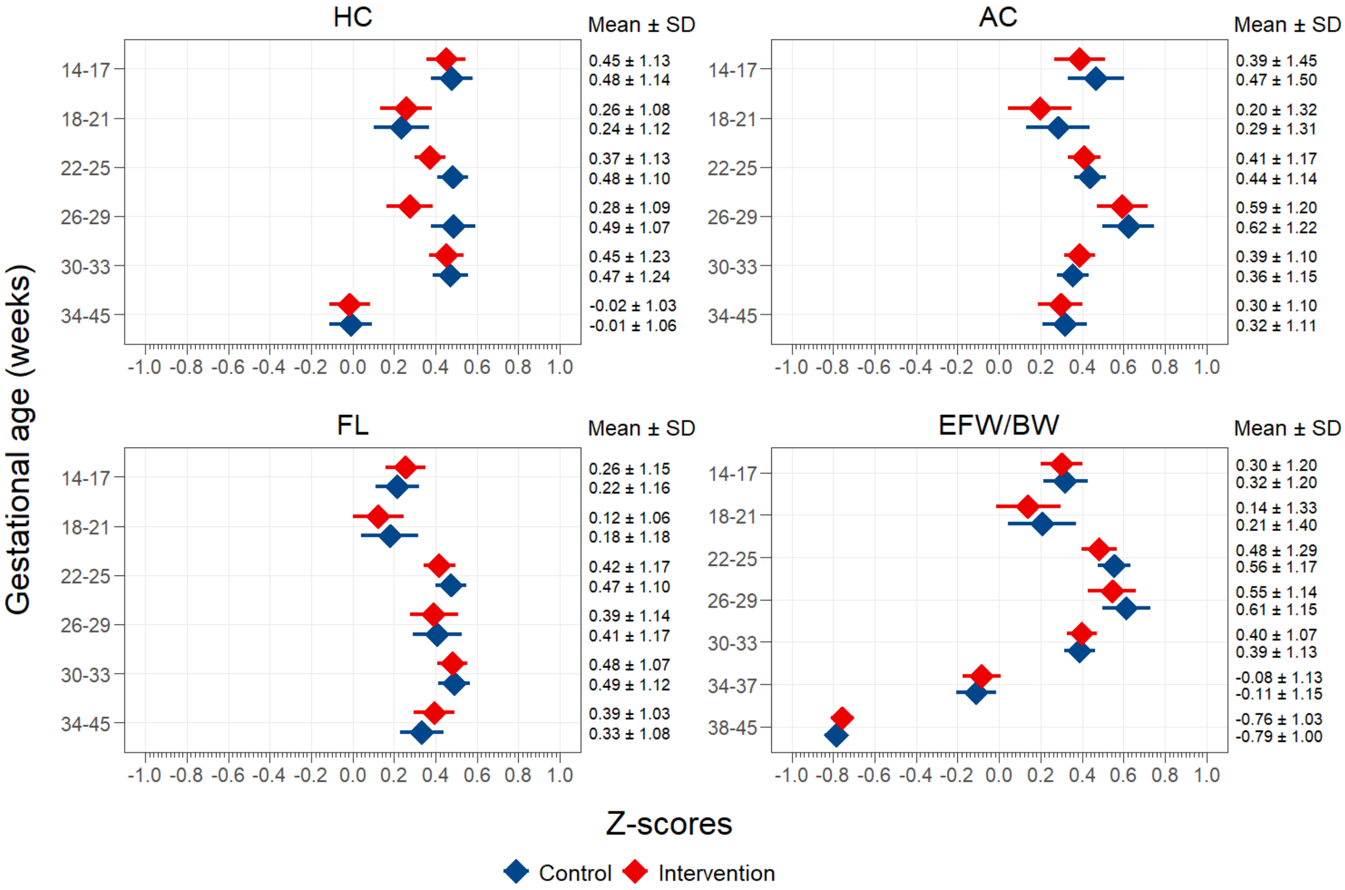

Figure S-2. Z-scores, mean +/- SD for HC, AC, FL, EFW by intervention status.

Figure-S7

```
p3.hc.gua <- fun.fig3(datain3 = df[df$irc ==
    "Guatemala" & df$visit != "Birth", ],
    var3 = "hcz", title3 = "Jalapa, Guatemala",
    x.group = "gacat2", x.min3 = -0.65, x.max3 = 1.5,
    gap.3 = 0.5, birth = "yes", brk = seq(-0.65,
        1.5, 0.3))

p3.hc.ind <- fun.fig3(datain3 = df[df$irc ==
    "India" & df$visit != "Birth", ], var3 = "hcz",
    title3 = "Tamil Nadu, India", x.group = "gacat2",
    x.min3 = -0.65, x.max3 = 1.5, gap.3 = 0.5,
    birth = "yes", brk = seq(-0.65, 1.5,
        0.3))

p3.hc.per <- fun.fig3(datain3 = df[df$irc ==
    "Peru" & df$visit != "Birth", ], var3 = "hcz",
    title3 = "Puno, Peru", x.group = "gacat2",
    x.min3 = -0.65, x.max3 = 1.5, gap.3 = 0.5,
    birth = "yes", brk = seq(-0.65, 1.5,
        0.3))

p3.hc.rwa <- fun.fig3(datain3 = df[df$irc ==
    "Rwanda" & df$visit != "Birth", ], var3 = "hcz",
    title3 = "Kayonza, Rwanda", x.group = "gacat2",
    x.min3 = -0.65, x.max3 = 1.5, gap.3 = 0.5,
    birth = "yes", brk = seq(-0.65, 1.5,
        0.3))

legend.p3 = doit4me::getlegend(p3.hc.rwa)
lheight.p3 <- sum(legend.p3$height)


g3.hc.a <- arrangeGrob(rbind(ggplotGrob(p3.hc.gua),
    ggplotGrob(p3.hc.per), size = "first"))

g3.hc.b <- arrangeGrob(rbind(ggplotGrob(p3.hc.ind),
    ggplotGrob(p3.hc.rwa), size = "last"))

g3.hc <- arrangeGrob(g3.hc.a, g3.hc.b, ncol = 2)
```

```
gg3.hc <- ggdraw() + draw_plot(g3.hc, x = 0.04,
    y = 0.1, width = 0.95, height = 0.9) +
    draw_plot(legend.p3, x = 0, y = 0.03,
        width = 0.95, height = 0.05) + annotate("text",
    x = 0.5, y = 0.08, size = 9, label = "Head circumference Z-scores") +
    annotate("text", x = 0.02, y = 0.5, size = 9,
        angle = 90, label = "Gestational age (weeks)") +
    annotate("text", x = 0.48, y = 0.96,
        size = 6, label = "Mean ± SD") +
    annotate("text", x = 0.955, y = 0.96,
        size = 6, label = "Mean ± SD") +
    annotate("text", x = 0.48, y = 0.515,
        size = 6, label = "Mean ± SD") +
    annotate("text", x = 0.955, y = 0.515,
        size = 6, label = "Mean ± SD")

tiff("Figure-3_HC_S7.tiff",
    units = "in", width = 15, height = 10,
    res = 100, compression = "lzw")
gg3.hc
invisible(dev.off())

setEPS()
postscript("Figure-3_HC_S7.eps",
    width = 15, height = 10)
gg3.hc
invisible(dev.off())
```

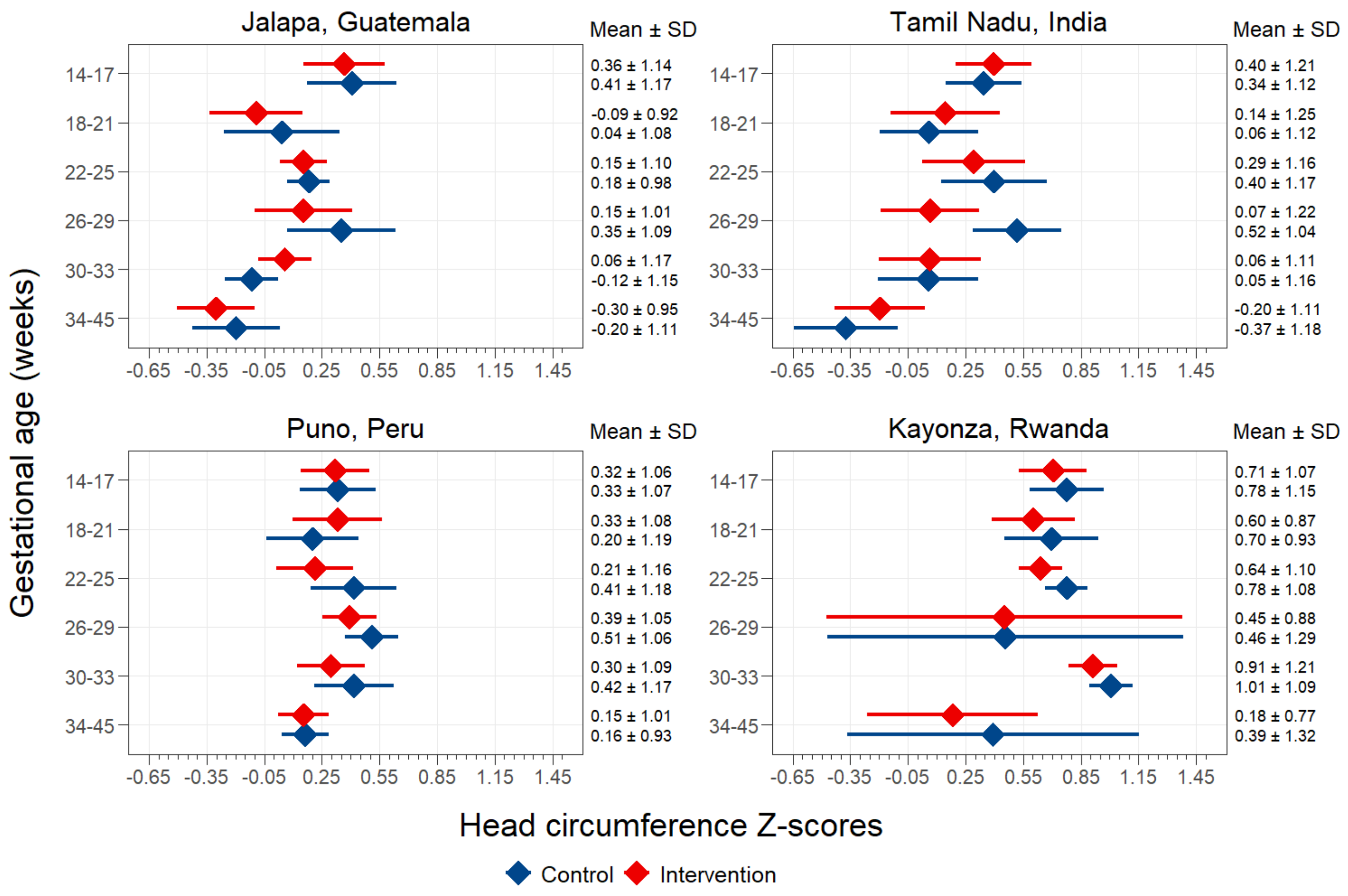
Jalapa, Guatemala
Mean ± SD
14-17
18-21
22-25
26-29
30-33
34-45
0.36 ± 1.14
0.41 ± 1.17
-0.09 ± 0.92
0.04 ± 1.08
0.15 ± 1.10
0.18 ± 0.98
0.15 ± 1.01
0.35 ± 1.09
0.06 ± 1.17
-0.12 ± 1.15
-0.30 ± 0.95
-0.20 ± 1.11
Tamil Nadu, India
Mean ± SD
0.40 ± 1.21
0.34 ± 1.12
0.14 ± 1.25
0.06 ± 1.12
0.29 ± 1.16
0.40 ± 1.17
0.07 ± 1.22
0.52 ± 1.04
0.06 ± 1.11
0.05 ± 1.16
-0.20 ± 1.11
-0.37 ± 1.18
Puno, Peru
Mean ± SD
0.32 ± 1.06
0.33 ± 1.07
0.33 ± 1.08
0.20 ± 1.19
0.21 ± 1.16
0.41 ± 1.18
0.39 ± 1.05
0.51 ± 1.06
0.30 ± 1.09
0.42 ± 1.17
0.15 ± 1.01
0.16 ± 0.93
Kayonza, Rwanda
Mean ± SD
0.71 ± 1.07
0.78 ± 1.15
0.60 ± 0.87
0.70 ± 0.93
0.64 ± 1.10
0.78 ± 1.08
0.45 ± 0.88
0.46 ± 1.29
0.91 ± 1.21
1.01 ± 1.09
0.18 ± 0.77
0.39 ± 1.32
-0.65 -0.35 -0.05 0.25 0.55 0.85 1.15 1.45
Gestational age (weeks)
Head circumference Z-scores
Control
Intervention

Figure-S7

Figure-S8

```
p3.ac.gua <- fun.fig3(datain3 = df[df$irc ==
    "Guatemala" & df$visit != "Birth", ],
    var3 = "acz", title3 = "Jalapa, Guatemala",
    x.group = "gacat2", x.min3 = -1.5, x.max3 = 1.5,
    gap.3 = 0.5, birth = "yes", brk = seq(-1.5,
        1.5, 0.5))

p3.ac.ind <- fun.fig3(datain3 = df[df$irc ==
    "India" & df$visit != "Birth", ], var3 = "acz",
    title3 = "Tamil Nadu, India", x.group = "gacat2",
    x.min3 = -1.5, x.max3 = 1.5, gap.3 = 0.5,
    birth = "yes", brk = seq(-1.5, 1.5, 0.5))

p3.ac.per <- fun.fig3(datain3 = df[df$irc ==
    "Peru" & df$visit != "Birth", ], var3 = "acz",
    title3 = "Puno, Peru", x.group = "gacat2",
    x.min3 = -1.5, x.max3 = 1.5, gap.3 = 0.5,
    birth = "yes", brk = seq(-1.5, 1.5, 0.5))

p3.ac.rwa <- fun.fig3(datain3 = df[df$irc ==
    "Rwanda" & df$visit != "Birth", ], var3 = "acz",
    title3 = "Kayonza, Rwanda", x.group = "gacat2",
    x.min3 = -1.5, x.max3 = 1.5, gap.3 = 0.5,
    birth = "yes", brk = seq(-1.5, 1.5, 0.5))

legend.p3 = doit4me::getlegend(p3.ac.rwa)
lheight.p3 <- sum(legend.p3$height)


g3.ac.a <- arrangeGrob(rbind(ggplotGrob(p3.ac.gua),
    ggplotGrob(p3.ac.per), size = "first"))

g3.ac.b <- arrangeGrob(rbind(ggplotGrob(p3.ac.ind),
    ggplotGrob(p3.ac.rwa), size = "last"))

g3.ac <- arrangeGrob(g3.ac.a, g3.ac.b, ncol = 2)

gg3.ac <- ggdraw() + draw_plot(g3.ac, x = 0.04,
    y = 0.1, width = 0.95, height = 0.9) +
    draw_plot(legend.p3, x = 0, y = 0.03,
```

```
        width = 0.95, height = 0.05) + annotate("text",
    x = 0.5, y = 0.08, size = 9, label = "Abdominal circumference Z-scores") +
    annotate("text", x = 0.02, y = 0.5, size = 9,
        angle = 90, label = "Gestational age (weeks)") +
    annotate("text", x = 0.48, y = 0.96,
        size = 6, label = "Mean ± SD") +
    annotate("text", x = 0.955, y = 0.96,
        size = 6, label = "Mean ± SD") +
    annotate("text", x = 0.48, y = 0.515,
        size = 6, label = "Mean ± SD") +
    annotate("text", x = 0.955, y = 0.515,
        size = 6, label = "Mean ± SD")

tiff("Figure-3_AC_S8.tiff",
    units = "in", width = 15, height = 10,
    res = 100, compression = "lzw")
gg3.ac
invisible(dev.off())

setEPS()
postscript("Figure-3_AC_S8.eps",
    width = 15, height = 10)
gg3.ac
invisible(dev.off())
```

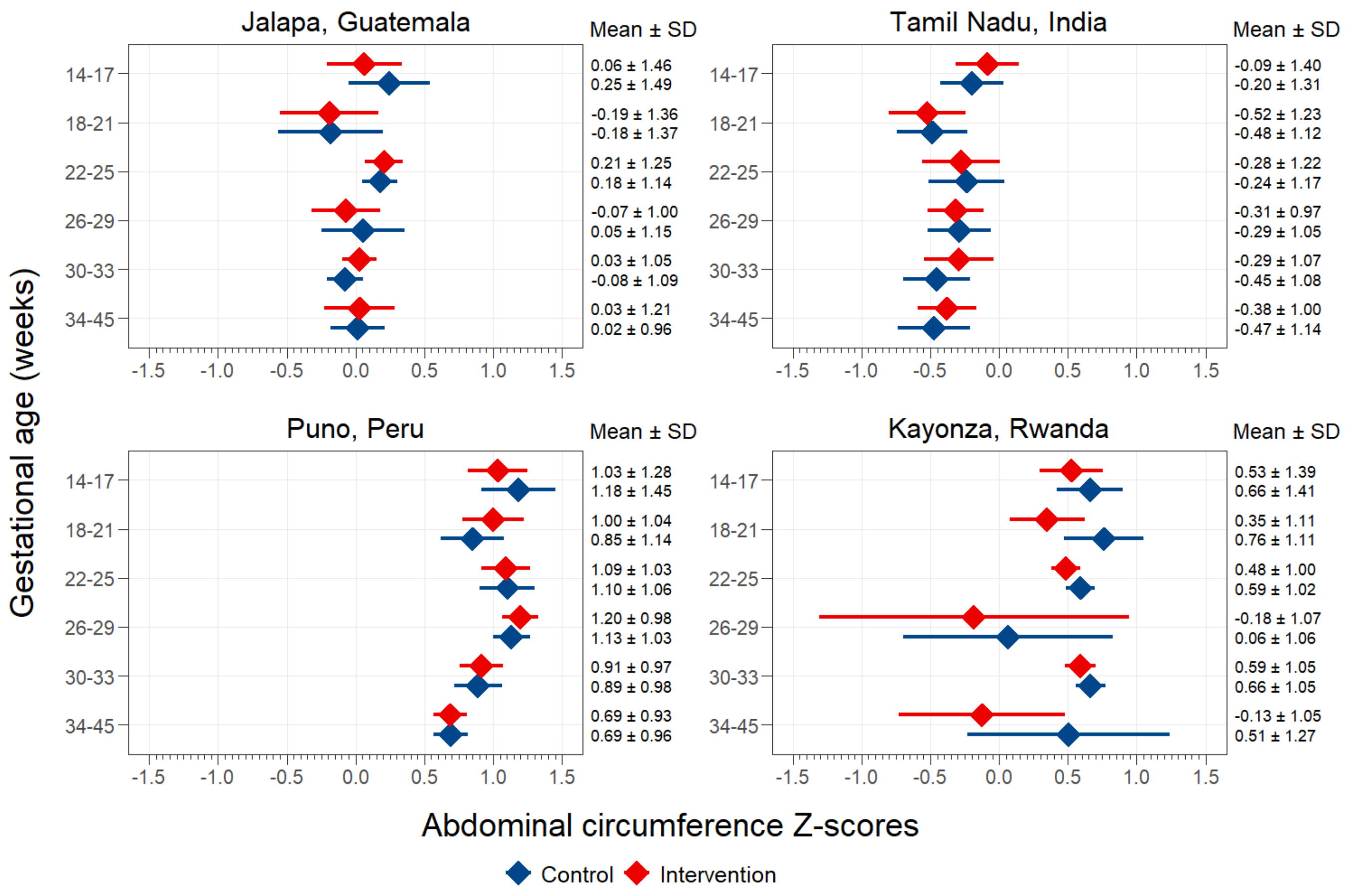
Jalapa, Guatemala
Mean ± SD
0.06 ± 1.46
0.25 ± 1.49
-0.19 ± 1.36
-0.18 ± 1.37
0.21 ± 1.25
0.18 ± 1.14
-0.07 ± 1.00
0.05 ± 1.15
0.03 ± 1.05
-0.08 ± 1.09
0.03 ± 1.21
0.02 ± 0.96
Tamil Nadu, India
Mean ± SD
-0.09 ± 1.40
-0.20 ± 1.31
-0.52 ± 1.23
-0.48 ± 1.12
-0.28 ± 1.22
-0.24 ± 1.17
-0.31 ± 0.97
-0.29 ± 1.05
-0.29 ± 1.07
-0.45 ± 1.08
-0.38 ± 1.00
-0.47 ± 1.14
Puno, Peru
Mean ± SD
1.03 ± 1.28
1.18 ± 1.45
1.00 ± 1.04
0.85 ± 1.14
1.09 ± 1.03
1.10 ± 1.06
1.20 ± 0.98
1.13 ± 1.03
0.91 ± 0.97
0.89 ± 0.98
0.69 ± 0.93
0.69 ± 0.96
Kayonza, Rwanda
Mean ± SD
0.53 ± 1.39
0.66 ± 1.41
0.35 ± 1.11
0.76 ± 1.11
0.48 ± 1.00
0.59 ± 1.02
-0.18 ± 1.07
0.06 ± 1.06
0.59 ± 1.05
0.66 ± 1.05
-0.13 ± 1.05
0.51 ± 1.27
14-17
18-21
22-25
26-29
30-33
34-45
-1.5
-1.0
-0.5
0.0
0.5
1.0
1.5
Gestational age (weeks)
Abdominal circumference Z-scores
Control
Intervention

Figure-S8

Figure-S9

```
p3.fl.gua <- fun.fig3(datain3 = df[df$irc ==
    "Guatemala" & df$visit != "Birth", ],
    var3 = "flz", title3 = "Jalapa, Guatemala",
    x.group = "gacat2", x.min3 = -1, x.max3 = 1.5,
    gap.3 = 0.5, birth = "yes", brk = seq(-1,
        1.5, 0.5))

p3.fl.ind <- fun.fig3(datain3 = df[df$irc ==
    "India" & df$visit != "Birth", ], var3 = "flz",
    title3 = "Tamil Nadu, India", x.group = "gacat2",
    x.min3 = -1, x.max3 = 1.5, gap.3 = 0.5,
    birth = "yes", brk = seq(-1, 1.5, 0.5))

p3.fl.per <- fun.fig3(datain3 = df[df$irc ==
    "Peru" & df$visit != "Birth", ], var3 = "flz",
    title3 = "Puno, Peru", x.group = "gacat2",
    x.min3 = -1, x.max3 = 1.5, gap.3 = 0.5,
    birth = "yes", brk = seq(-1, 1.5, 0.5))

p3.fl.rwa <- fun.fig3(datain3 = df[df$irc ==
    "Rwanda" & df$visit != "Birth", ], var3 = "flz",
    title3 = "Kayonza, Rwanda", x.group = "gacat2",
    x.min3 = -1, x.max3 = 1.5, gap.3 = 0.5,
    birth = "yes", brk = seq(-1, 1.5, 0.5))

legend.p3 = doit4me::getlegend(p3.fl.rwa)
lheight.p3 <- sum(legend.p3$height)


g3.fl.a <- arrangeGrob(rbind(ggplotGrob(p3.fl.gua),
    ggplotGrob(p3.fl.per), size = "first"))

g3.fl.b <- arrangeGrob(rbind(ggplotGrob(p3.fl.ind),
    ggplotGrob(p3.fl.rwa), size = "last"))

g3.fl <- arrangeGrob(g3.fl.a, g3.fl.b, ncol = 2)

gg3.fl <- ggdraw() + draw_plot(g3.fl, x = 0.04,
    y = 0.1, width = 0.95, height = 0.9) +
    draw_plot(legend.p3, x = 0, y = 0.03,
```

```
        width = 0.95, height = 0.05) + annotate("text",
    x = 0.5, y = 0.08, size = 9, label = "Femur length Z-scores") +
    annotate("text", x = 0.02, y = 0.5, size = 9,
        angle = 90, label = "Gestational age (weeks)") +
    annotate("text", x = 0.48, y = 0.96,
        size = 6, label = "Mean ± SD") +
    annotate("text", x = 0.955, y = 0.96,
        size = 6, label = "Mean ± SD") +
    annotate("text", x = 0.48, y = 0.515,
        size = 6, label = "Mean ± SD") +
    annotate("text", x = 0.955, y = 0.515,
        size = 6, label = "Mean ± SD")

tiff("Figure-3_FL_S9.tiff",
    units = "in", width = 15, height = 10,
    res = 100, compression = "lzw")
gg3.fl
invisible(dev.off())

setEPS()
postscript("Figure-3_FL_S9.eps",
    width = 15, height = 10)
gg3.fl
invisible(dev.off())
```

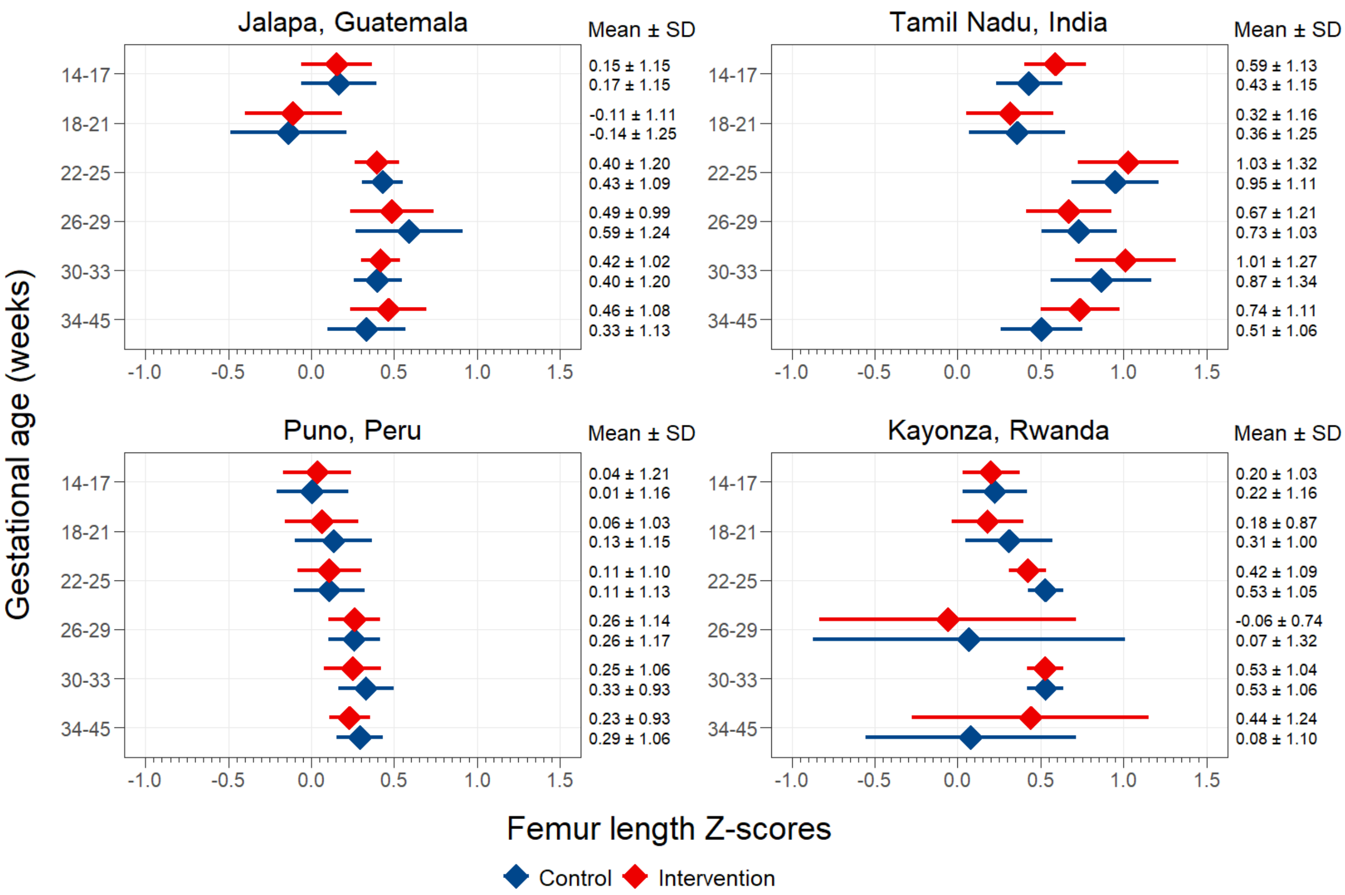
Jalapa, Guatemala
Mean ± SD
14-17
18-21
22-25
26-29
30-33
34-45
0.15 ± 1.15
0.17 ± 1.15
-0.11 ± 1.11
-0.14 ± 1.25
0.40 ± 1.20
0.43 ± 1.09
0.49 ± 0.99
0.59 ± 1.24
0.42 ± 1.02
0.40 ± 1.20
0.46 ± 1.08
0.33 ± 1.13
Tamil Nadu, India
Mean ± SD
0.59 ± 1.13
0.43 ± 1.15
0.32 ± 1.16
0.36 ± 1.25
1.03 ± 1.32
0.95 ± 1.11
0.67 ± 1.21
0.73 ± 1.03
1.01 ± 1.27
0.87 ± 1.34
0.74 ± 1.11
0.51 ± 1.06
Puno, Peru
Mean ± SD
0.04 ± 1.21
0.01 ± 1.16
0.06 ± 1.03
0.13 ± 1.15
0.11 ± 1.10
0.11 ± 1.13
0.26 ± 1.14
0.26 ± 1.17
0.25 ± 1.06
0.33 ± 0.93
0.23 ± 0.93
0.29 ± 1.06
Kayonza, Rwanda
Mean ± SD
0.20 ± 1.03
0.22 ± 1.16
0.18 ± 0.87
0.31 ± 1.00
0.42 ± 1.09
0.53 ± 1.05
-0.06 ± 0.74
0.07 ± 1.32
0.53 ± 1.04
0.53 ± 1.06
0.44 ± 1.24
0.08 ± 1.10
-1.0
-0.5
0.0
0.5
1.0
1.5
Gestational age (weeks)
Femur length Z-scores
Control
Intervention

Figure-S9

Figure-S10

```
p3.efw.gua <- fun.fig3(datain3 = df[df$irc ==
    "Guatemala", ], var3 = "efwz", title3 = "Jalapa, Guatemala",
    x.min3 = -1.55, x.max3 = 1.2, gap.3 = 0.5,
    birth = "yes", brk = seq(-1.5, 1.2, 0.3))

p3.efw.ind <- fun.fig3(datain3 = df[df$irc ==
    "India", ], var3 = "efwz", title3 = "Tamil Nadu, India",
    x.min3 = -1.55, x.max3 = 1.2, gap.3 = 0.5,
    birth = "yes", brk = seq(-1.5, 1.2, 0.3))

p3.efw.per <- fun.fig3(datain3 = df[df$irc ==
    "Peru", ], var3 = "efwz", title3 = "Puno, Peru",
    x.min3 = -1.55, x.max3 = 1.2, gap.3 = 0.5,
    birth = "yes", brk = seq(-1.5, 1.2, 0.3))

p3.efw.rwa <- fun.fig3(datain3 = df[df$irc ==
    "Rwanda", ], var3 = "efwz", title3 = "Kayonza, Rwanda",
    x.min3 = -1.55, x.max3 = 1.2, gap.3 = 0.5,
    birth = "yes", brk = seq(-1.5, 1.2, 0.3))

legend.p3 = doit4me::getlegend(p3.efw.rwa)
lheight.p3 <- sum(legend.p3$height)


g3.efw.a <- arrangeGrob(rbind(ggplotGrob(p3.efw.gua),
    ggplotGrob(p3.efw.per), size = "first"))

g3.efw.b <- arrangeGrob(rbind(ggplotGrob(p3.efw.ind),
    ggplotGrob(p3.efw.rwa), size = "last"))

g3.efw <- arrangeGrob(g3.efw.a, g3.efw.b,
    ncol = 2)

gg3.efw <- ggdraw() + draw_plot(g3.efw, x = 0.04,
    y = 0.1, width = 0.95, height = 0.9) +
    draw_plot(legend.p3, x = 0, y = 0.03,
        width = 0.95, height = 0.05) + annotate("text",
    x = 0.5, y = 0.08, size = 9, label = "Estimated fetal weight/ Birth weight Z-scores")
    annotate("text", x = 0.02, y = 0.5, size = 9,
        angle = 90, label = "Gestational age (weeks)") +
```

```
    annotate("text", x = 0.48, y = 0.96,
        size = 6, label = "Mean ± SD") +
    annotate("text", x = 0.955, y = 0.96,
        size = 6, label = "Mean ± SD") +
    annotate("text", x = 0.48, y = 0.515,
        size = 6, label = "Mean ± SD") +
    annotate("text", x = 0.955, y = 0.515,
        size = 6, label = "Mean ± SD")



tiff("Figure-3_EFW_S10.tiff",
    units = "in", width = 15, height = 10,
    res = 100, compression = "lzw")
gg3.efw
invisible(dev.off())

setEPS()
postscript("Figure-3_EFW_S10.eps",
    width = 15, height = 10)
gg3.efw
invisible(dev.off())
```

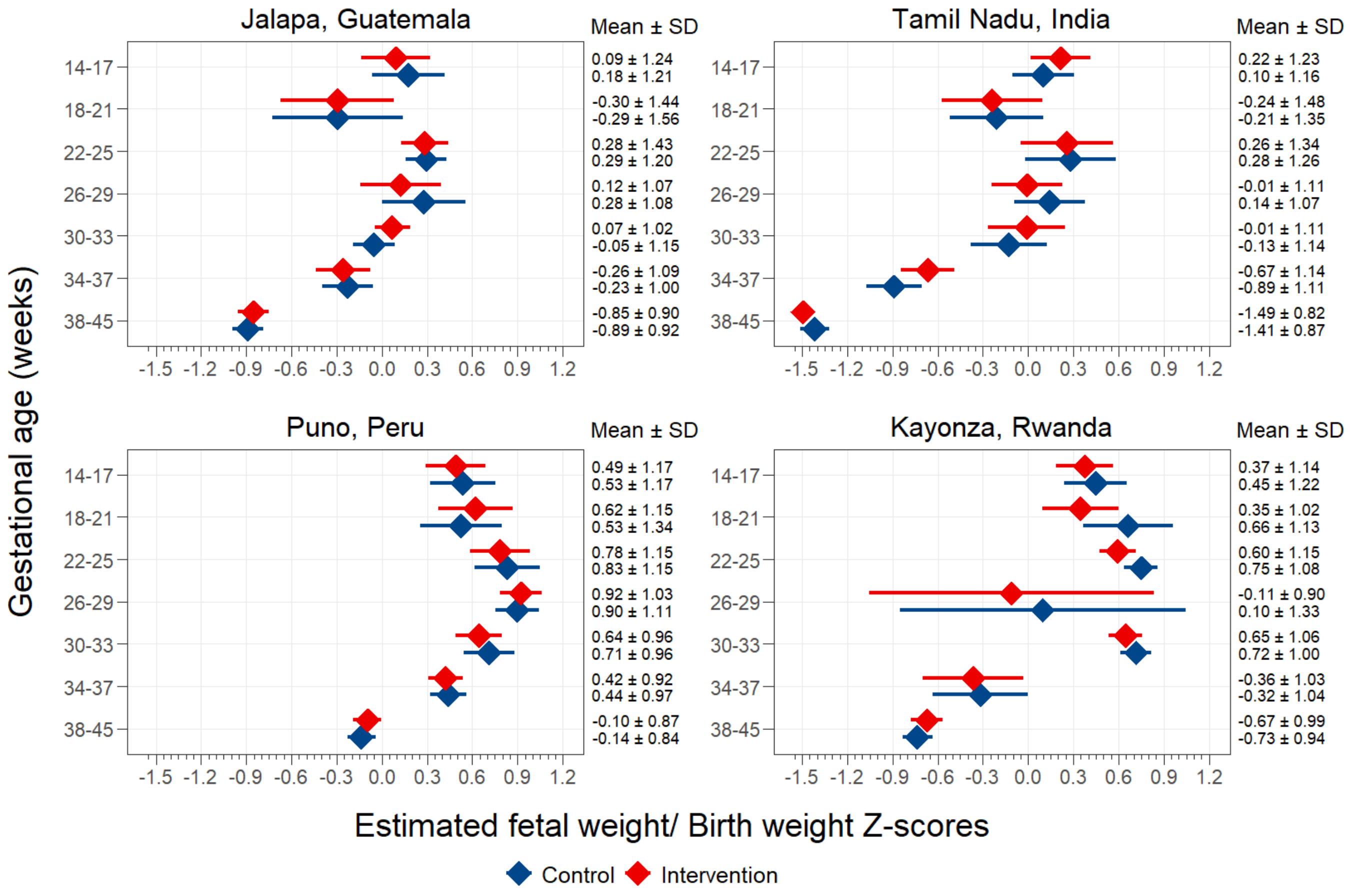
Jalapa, Guatemala
Tamil Nadu, India
Puno, Peru
Kayonza, Rwanda
Mean ± SD
Gestational age (weeks)
14-17
18-21
22-25
26-29
30-33
34-37
38-45
0.09 ± 1.24
0.18 ± 1.21
-0.30 ± 1.44
-0.29 ± 1.56
0.28 ± 1.43
0.29 ± 1.20
0.12 ± 1.07
0.28 ± 1.08
0.07 ± 1.02
-0.05 ± 1.15
-0.26 ± 1.09
-0.23 ± 1.00
-0.85 ± 0.90
-0.89 ± 0.92
0.22 ± 1.23
0.10 ± 1.16
-0.24 ± 1.48
-0.21 ± 1.35
0.26 ± 1.34
0.28 ± 1.26
-0.01 ± 1.11
0.14 ± 1.07
-0.01 ± 1.11
-0.13 ± 1.14
-0.67 ± 1.14
-0.89 ± 1.11
-1.49 ± 0.82
-1.41 ± 0.87
0.49 ± 1.17
0.53 ± 1.17
0.62 ± 1.15
0.53 ± 1.34
0.78 ± 1.15
0.83 ± 1.15
0.92 ± 1.03
0.90 ± 1.11
0.64 ± 0.96
0.71 ± 0.96
0.42 ± 0.92
0.44 ± 0.97
-0.10 ± 0.87
-0.14 ± 0.84
0.37 ± 1.14
0.45 ± 1.22
0.35 ± 1.02
0.66 ± 1.13
0.60 ± 1.15
0.75 ± 1.08
-0.11 ± 0.90
0.10 ± 1.33
0.65 ± 1.06
0.72 ± 1.00
-0.36 ± 1.03
-0.32 ± 1.04
-0.67 ± 0.99
-0.73 ± 0.94
-1.5 -1.2 -0.9 -0.6 -0.3 0.0 0.3 0.6 0.9 1.2
Estimated fetal weight/ Birth weight Z-scores
Control
Intervention

Figure-S10

Figure-3

```
# var = 'hc' lab = 'HC' horjust = -0.3
# grouped = NULL lab.int = 'hcz.int'
# lab.cont = 'hcz.cont'


fun.fig4 <- function(var, grouped = NULL,
    lab, horjust, lab.int, lab.cont) {

    if (var != "efw") {
        tab4 <- tab4[tab4$visits != "Birth",
            ]
    }

    p1 <- ggplot(data = tab4) + geom_point(aes(x = !!sym(var),
        y = visits), shape = "diamond", size = 10) +
        geom_errorbar(aes(xmin = !!sym(paste0(var,
            ".lb")), xmax = !!sym(paste0(var,
            ".ub")), y = visits), size = 1.5,
            width = 0) + scale_x_continuous("Difference in Z-score",
        limits = c(-0.4, 0.4), breaks = seq(-0.4,
            0.4, 0.1), minor_breaks = seq(-0.4,
            0.4, 0.02), guide = "axis_minor",
        labels = c("-0.4", "-0.3", "-0.2",
            "-0.1", "0.0", "0.1", "0.2",
            "0.3", "0.4")) + geom_vline(aes(xintercept = 0),
        linetype = "dashed", size = 1) +
        scale_y_discrete("") + coord_cartesian(clip = "off") +
        theme_bw() + theme(panel.grid = element_blank(),
        axis.title = element_blank(), axis.ticks.length = unit(0.3,
            "cm"), ggh4x.axis.ticks.length.minor = rel(0.5),
        axis.text = element_text(size = 22)) +
        annotate("text", x = -Inf, y = Inf,
            hjust = horjust, vjust = 1.8,
            label = lab, size = 8)

    p2 <- ggplot(data = tab4) + geom_text(aes(y = visits,
        x = 0), label = tab4[[lab.int]],
        size = 6.5) + geom_text(aes(y = visits,
        x = 45), label = tab4[[lab.cont]],
```

```
        size = 6.5) + geom_text(aes(y = visits,
        x = 110), label = tab4[[paste0(var,
        ".lab")]], hjust = 0.5, size = 6.5) +
        scale_x_continuous(limits = c(0,
            150)) + theme_void() + coord_cartesian(clip = "off")

    theme.x <- theme(axis.text.x = element_blank(),
        axis.title.x = element_blank(), axis.ticks.x = element_blank())

    if (var %in% c("hc", "ac", "fl")) {
        p1 <- p1 + theme.x + theme(plot.margin = unit(c(1,
            0, -0.3, 2.43), "lines"))
    } else {
        p1 <- p1 + theme(plot.margin = unit(c(1,
            0, -0.3, 1), "lines"))
    }

    p <- plot_grid(p1, p2, align = "v", ncol = 2,
        rel_widths = c(1.1, 1.5), rel_heights = c(1.1,
            1))

    return(p)
}

p.hc <- fun.fig4(var = "hc", lab = "HC",
    horjust = -0.3, lab.int = "hcz.int",
    lab.cont = "hcz.cont")

p.ac <- fun.fig4(var = "ac", lab = "AC",
    horjust = -0.3, lab.int = "acz.int",
    lab.cont = "acz.cont")

p.fl <- fun.fig4(var = "fl", lab = "FL",
    horjust = -0.3, lab.int = "flz.int",
    lab.cont = "flz.cont")

p.efw <- fun.fig4(var = "efw", lab = "EFW/BW",
    horjust = -0.1, lab.int = "efwz.int",
    lab.cont = "efwz.cont")

p4 <- arrangeGrob(rbind(ggplotGrob(p.hc),
```

```r
    ggplotGrob(p.ac), ggplotGrob(p.fl), ggplotGrob(p.efw),
    size = "first"))

g4 <- ggdraw() + draw_plot(p4, x = 0, y = 0.08,
    height = 0.88, width = 1) + annotate("text",
    x = 0.52, y = 0.96, size = 8, label = "Intervention") +
    annotate("text", x = 0.66, y = 0.96,
        size = 8, label = "Control") + annotate("text",
    x = 0.86, y = 0.96, size = 7, label = "Difference (98.75% CI)") +
    annotate("text", x = 0.16, y = 0.06,
        size = 7, label = "Control better",
        vjust = 0.5) + annotate("text", x = 0.34,
    y = 0.06, size = 7, label = "Intervention better",
    vjust = 0.5) + annotate("segment", x = 0.16 -
    0.06, xend = 0.16 - 0.06 - 0.07, y = 0.06,
    yend = 0.06, arrow = arrow(length = unit(0.3,
        "cm"))) + annotate("segment", x = 0.34 +
    0.08, xend = 0.34 + 0.08 + 0.07, y = 0.06,
    yend = 0.06, arrow = arrow(length = unit(0.3,
        "cm"))) + annotate("text", x = 0.25,
    y = 0.015, size = 9, label = "Difference in Z-score",
    vjust = 0.5)


tiff("Figure-3.tiff",
    units = "in", width = 15, height = 15,
    res = 100, compression = "lzw")
g4
invisible(dev.off())

setEPS()
postscript("Figure-3.eps",
    width = 15, height = 15)
g4
invisible(dev.off())
```

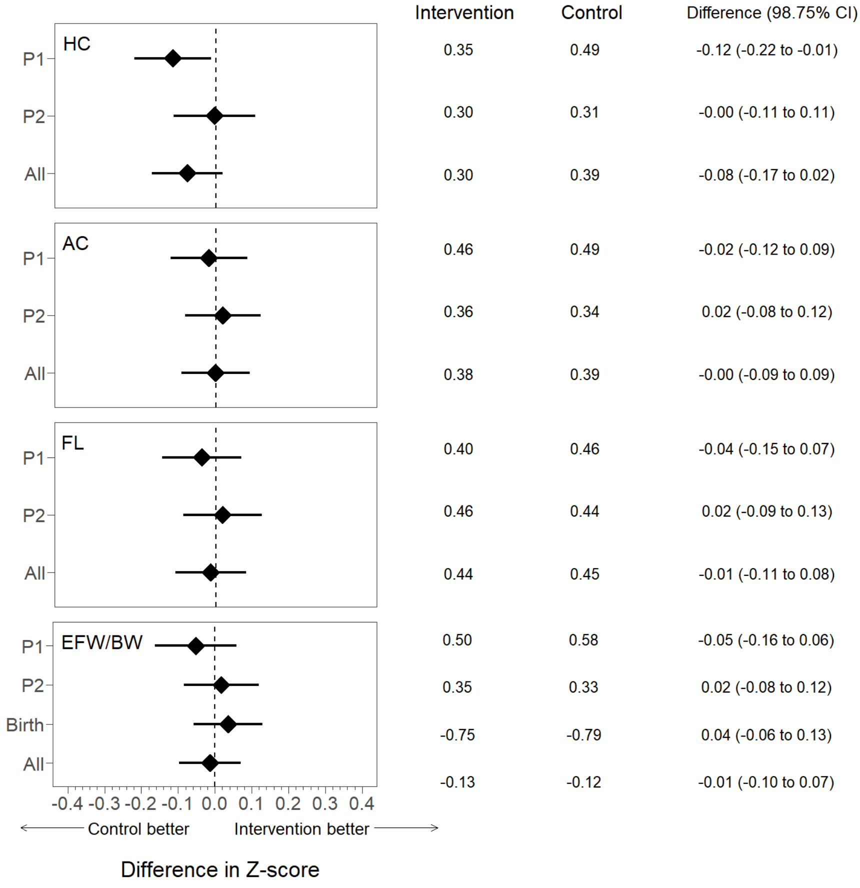

		Intervention	Control	Difference (98.75% CI)
HC	P1	0.35	0.49	-0.12 (-0.22 to -0.01)
	P2	0.30	0.31	-0.00 (-0.11 to 0.11)
	All	0.30	0.39	-0.08 (-0.17 to 0.02)
AC	P1	0.46	0.49	-0.02 (-0.12 to 0.09)
	P2	0.36	0.34	0.02 (-0.08 to 0.12)
	All	0.38	0.39	-0.00 (-0.09 to 0.09)
FL	P1	0.40	0.46	-0.04 (-0.15 to 0.07)
	P2	0.46	0.44	0.02 (-0.09 to 0.13)
	All	0.44	0.45	-0.01 (-0.11 to 0.08)
EFW/BW	P1	0.50	0.58	-0.05 (-0.16 to 0.06)
	P2	0.35	0.33	0.02 (-0.08 to 0.12)
	Birth	-0.75	-0.79	0.04 (-0.06 to 0.13)
	All	-0.13	-0.12	-0.01 (-0.10 to 0.07)

Figure 3. ITT effects.

Figure-S11

```
tab5 <- rbind(tab5, df.temp)


tab5$fl.lab <- paste0(sprintf("%3.2f", round(tab5$fl,
    2)), " (", sprintf("%3.2f", round(tab5$fl.lb,
    2)), " to ", sprintf("%3.2f", round(tab5$fl.ub,
    2)), ")")

tab5$ac.lab <- paste0(sprintf("%3.2f", round(tab5$ac,
    2)), " (", sprintf("%3.2f", round(tab5$ac.lb,
    2)), " to ", sprintf("%3.2f", round(tab5$ac.ub,
    2)), ")")

tab5$hc.lab <- paste0(sprintf("%3.2f", round(tab5$hc,
    2)), " (", sprintf("%3.2f", round(tab5$hc.lb,
    2)), " to ", sprintf("%3.2f", round(tab5$hc.ub,
    2)), ")")

tab5$efw.lab <- paste0(sprintf("%3.2f", round(tab5$efw,
    2)), " (", sprintf("%3.2f", round(tab5$efw.lb,
    2)), " to ", sprintf("%3.2f", round(tab5$efw.ub,
    2)), ")")

tab5$weeks <- factor(tab5$weeks, levels = c("40",
    "33", "25"))

tab5$groups <- factor(tab5$groups, levels = c(2,
    1), labels = c("Late randomization",
    "Early randomization"))

fun.fig5 <- function(var, lab, hjst = -0.3) {

    if (var == "efw") {
        tab5$weeks <- factor(tab5$weeks,
            levels = c("40", "33", "25"))
    }

    if (var != "efw") {
        tab5 <- tab5[1:4, ]
```

```
    tab5$weeks <- factor(tab5$weeks,
        levels = c("33", "25"))
}

p <- ggplot(data = tab5)

if (var == "efw") {
    p <- p + annotate("rect", xmin = -Inf,
        xmax = Inf, ymin = 1.5, ymax = 2.5,
        alpha = 0.3, fill = "grey")
} else {
    p <- p + annotate("rect", xmin = -Inf,
        xmax = Inf, ymin = -Inf, ymax = 1.5,
        alpha = 0.3, fill = "grey")
}

p <- p + geom_point(aes(x = !!sym(var),
    y = weeks, color = factor(groups)),
    position = position_dodge(width = 0.8),
    shape = "diamond", size = 10) + geom_errorbar(aes(xmin = !!sym(paste0(var,
    ".lb")), xmax = !!sym(paste0(var,
    ".ub")), y = weeks, color = factor(groups)),
    position = position_dodge(width = 0.8),
    size = 1.5, width = 0) + scale_color_manual(values = c("#00468b",
    "#ed0000"), labels = c("Late randomization",
    "Early randomization"), guide = guide_legend(reverse = TRUE)) +
    scale_x_continuous("Difference in Z-score",
        limits = c(-0.4, 0.4), breaks = seq(-0.4,
            0.4, 0.1), minor_breaks = seq(-0.4,
            0.4, 0.02), guide = "axis_minor",
        labels = c("-0.4", "-0.3", "-0.2",
            "-0.1", "0.0", "0.1", "0.2",
            "0.3", "0.4")) + geom_text(aes(y = weeks,
    x = Inf, group = factor(groups)),
    position = position_dodge(width = 0.8),
    label = tab5[[paste0(var, ".lab")]],
    hjust = -0.2, size = 6) + geom_vline(aes(xintercept = 0),
    linetype = "dashed", size = 1) +
    scale_y_discrete("") + coord_cartesian(clip = "off") +
    theme_bw() + theme(panel.grid = element_blank(),
    axis.title = element_blank(), axis.ticks.length = unit(0.3,
```

```
            "cm"), ggh4x.axis.ticks.length.minor = rel(0.5),
        legend.position = "none", legend.title = element_blank(),
        axis.text = element_text(size = 18),
        plot.margin = unit(c(0, 15.25, -0.3,
            3), "lines")) + annotate("text",
        x = -Inf, y = Inf, hjust = hjst,
        vjust = 1.8, label = lab, size = 8)

    return(p)
}

theme.x <- theme(axis.text.x = element_blank(),
    axis.title.x = element_blank(), axis.ticks.x = element_blank())

p5.hc <- fun.fig5(var = "hc", lab = "HC") +
    theme.x + theme(plot.margin = unit(c(1,
    13.25, -0.3, 3), "lines"))
p5.ac <- fun.fig5(var = "ac", lab = "AC") +
    theme.x
p5.fl <- fun.fig5(var = "fl", lab = "FL") +
    theme.x
p5.efw <- fun.fig5(var = "efw", lab = "EFW/BW",
    hjst = -0.1)

# p5 <- arrangeGrob(ggplotGrob(p5.hc),
# ggplotGrob(p5.ac), ggplotGrob(p5.fl),
# ggplotGrob(p5.efw),
# heights=c(2,2,2,3))

legend <- getlegend(p5.hc)

p5 <- plot_grid(p5.hc, p5.ac, p5.fl, p5.efw,
    align = "v", nrow = 4, rel_heights = c(2,
        2, 2, 3))


g5 <- ggdraw() + draw_plot(p5, x = 0, y = 0.1,
    height = 0.85, width = 1) + annotate("text",
    x = 0.02, y = 0.5, size = 9, label = "Gestational age (weeks)",
    angle = 90, vjust = 0.5) + annotate("text",
    x = 0.82, y = 0.96, size = 7, label = "Difference (98.75% CI)") +
```

```
    annotate("text", x = 0.24, y = 0.08,
        size = 6, label = "Control better",
        vjust = 0.5) + annotate("text", x = 0.54,
    y = 0.08, size = 6, label = "Intervention better",
    vjust = 0.5) + annotate("segment", x = 0.25 -
    0.11, xend = 0.25 - 0.11 - 0.07, y = 0.08,
    yend = 0.08, arrow = arrow(length = unit(0.3,
        "cm"))) + annotate("segment", x = 0.54 +
    0.13, xend = 0.54 + 0.13 + 0.07, y = 0.08,
    yend = 0.08, arrow = arrow(length = unit(0.3,
        "cm"))) + draw_plot(legend, x = -0.1,
    y = 0.03, height = 0.03, width = 1) +
    annotate("text", x = 0.37, y = 0.05,
        size = 8, label = "Difference in Z-score",
        vjust = 0.5)


tiff("Figure-S11.tiff",
    units = "in", width = 8, height = 12,
    res = 100, compression = "lzw")
g5
invisible(dev.off())

setEPS()
postscript("Figure-S11.eps",
    width = 8, height = 12)
g5
invisible(dev.off())
```

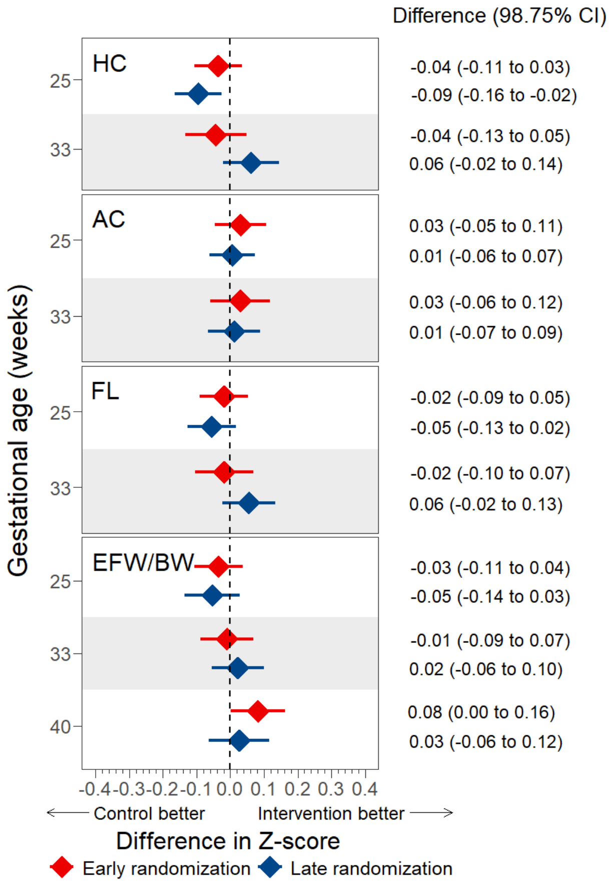

Figure S11. Early vs. late randomization vs. controls.

Figure-S12

```
fun.fig6 <- function(exp.var = "avgpm",
                     child.var = "hcz",
                     y.title = "HC",
                     title = bquote(PM[2.5]),
                     y.min = -2,
                     y.max = 2,
                     y.break = 0.5){

tab6 <- hap %>%
  dplyr::filter(!is.na(!!sym(exp.var))) %>%
  dplyr::arrange(!!sym(exp.var)) %>%
  dplyr::mutate(quant.var = ntile(!!sym(exp.var), 4)) %>%
  dplyr::group_by(visit, quant.var) %>%
  dplyr::summarise(mean = mean(!!sym(child.var), na.rm = TRUE),
                   sd = sd(!!sym(child.var), na.rm = TRUE)) %>%
  dplyr::mutate(mean = ifelse(mean == "NaN", NA, mean),
                sd = ifelse(mean == "NaN", NA, sd))

p <-
ggplot(data = tab6) +
  geom_point(aes(x = visit,
                 y = mean,
                 color = factor(quant.var)),
             shape = "diamond",
             position = position_dodge(width = 0.8),
             size = 7) +
  geom_errorbar(aes(x = visit,
                    ymin = mean-(1.96*sd),
                    ymax = mean + (1.96*sd),
                    color = factor(quant.var)),
                position = position_dodge(width = 0.8),
                size = 1,
                width = 0) +
  geom_hline(aes(yintercept = 0), linetype = "dashed") +
  geom_hline(aes(yintercept = 2), linetype = "dashed") +
  geom_hline(aes(yintercept = -2), linetype = "dashed") +
  scale_x_discrete("") +
  scale_y_continuous(y.title,
                     limits = c(y.min, y.max),
```

```r
                      breaks = seq(y.min, y.max, y.break),
                      minor_breaks = seq(y.min, y.max, 0.5),
                      guide = "axis_minor") +
  scale_color_manual(values = c("gray90", "gray70", "gray50", "gray0"),
                      labels = c("Q1", "Q2", "Q3", "Q4")) +
  theme_bw() +
  #guides(color = guide_legend(override.aes = list(size = 7, linelength = 9))) +
  theme(panel.grid = element_blank(),
        legend.position = "none",
        axis.title.x = element_blank(),
        axis.title.y = element_text(size = 18, angle = 0, vjust = 0.5),
        axis.ticks.length = unit(0.3, "cm"),
        ggh4x.axis.ticks.length.minor = rel(0.5),
        legend.title = element_blank(),
        legend.key.width = unit(1,"cm"),
        axis.text = element_text(size = 14),
        plot.title = element_text(size = 22, hjust = 0.5))

if(child.var == "hcz" & exp.var == "avgpm"){p <- p + ggtitle(bquote(PM[2.5]))}
if(child.var == "hcz" & exp.var == "avgbc"){p <- p + ggtitle(bquote(BC[ ]))}
if(child.var == "hcz" & exp.var == "avgco"){p <- p + ggtitle(bquote(CO[ ]))}
if(exp.var != "avgpm"){p <- p + theme(axis.title.y = element_blank(),
                                      axis.text.y = element_blank(),
                                      axis.ticks.y = element_blank())}
if(child.var != "efwz"){p <- p + theme(axis.text.x = element_blank(),
                                       axis.ticks.x = element_blank())}

return(p)
}


y.title.list <- c("  HC", "  AC", "  FL", "EFW/BW         ")
exp.varlist <- c("avgpm", "avgbc", "avgco")
child.varlist <- c("hcz", "acz", "flz", "efwz")

for(cc in 1:4){
  for(ee in 1:3){
    assign(paste0("p_",
                  substr(exp.varlist[ee], 4, 5), "_",
                  child.varlist[cc]),
```

```r
            fun.fig6(exp.var = exp.varlist[ee],
                     child.var = child.varlist[cc],
                     title = title.list[ee],
                     y.title = y.title.list[cc],
                     y.min = -4,
                     y.max = 4,
                     y.break = 2)
            )
  }
}


legend = doit4me::getlegend(p_pm_acz)
lheight <- sum(legend$height)


p6_c1 <- arrangeGrob(rbind(ggplotGrob(p_pm_hcz),
                           ggplotGrob(p_pm_acz),
                           ggplotGrob(p_pm_flz),
                           ggplotGrob(p_pm_efwz), size = "first"))

p6_c2 <- arrangeGrob(rbind(ggplotGrob(p_bc_hcz),
                           ggplotGrob(p_bc_acz),
                           ggplotGrob(p_bc_flz),
                           ggplotGrob(p_bc_efwz), size = "first"))

p6_c3 <- arrangeGrob(rbind(ggplotGrob(p_co_hcz),
                           ggplotGrob(p_co_acz),
                           ggplotGrob(p_co_flz),
                           ggplotGrob(p_co_efwz), size = "first"))


#p6 <- arrangeGrob(arrangeGrob(p6_c1, p6_c2, p6_c3, ncol=3))

p6 <- plot_grid(p6_c1, p6_c2, p6_c3, ncol = 3, rel_widths = c(1.17, 1, 1))

g6 <- ggdraw() +
  draw_plot(p6, x = 0.04, y = 0.1, height = 0.90, width = 0.90) +
  annotate("text", x = 0.5, y= 0.075, size = 9, label = "Visit") +
  draw_plot(legend, x = 0.5, y = 0.01, height = 0.1, width = 0.95, hjust = 0.5) +
  annotate("text", x = 0.97, y= 0.5, size = 9, label = "Z-scores", angle = -90)
```

```r
hap.tab <- left_join(hap, df[,c("hhid", "lpg")], by = "hhid")

pm.int <- mean(hap.tab$pm[hap.tab$lpg == 1], na.rm = TRUE)
pm.cont <- mean(hap.tab$pm[hap.tab$lpg == 0], na.rm = TRUE)

bc.int <- mean(hap.tab$bc[hap.tab$lpg == 1], na.rm = TRUE)
bc.cont <- mean(hap.tab$bc[hap.tab$lpg == 0], na.rm = TRUE)

co.int <- mean(hap.tab$co[hap.tab$lpg == 1], na.rm = TRUE)
co.cont <- mean(hap.tab$co[hap.tab$lpg == 0], na.rm = TRUE)

((pm.cont - pm.int)/pm.cont)*100
```

```
[1] 38.88527
```

```r
((bc.cont - bc.int)/bc.cont)*100
```

```
[1] 38.83283
```

```r
((co.cont - co.int)/co.cont)*100
```

```
[1] 38.28569
```

```r
quantile(hap$avgpm, na.rm = TRUE)
```

```
       0%         25%         50%         75%        100%
 9.355287   37.451456   68.835603  118.814302 2099.882946
```

```r
tiff("Figure-S12.tiff",
     units="in", width = 15, height = 10, res=100, compression = 'lzw')
g6
invisible(dev.off())


setEPS()
postscript("Figure-S12.eps", width = 15, height = 10)
```

```
g6
invisible(dev.off())
```

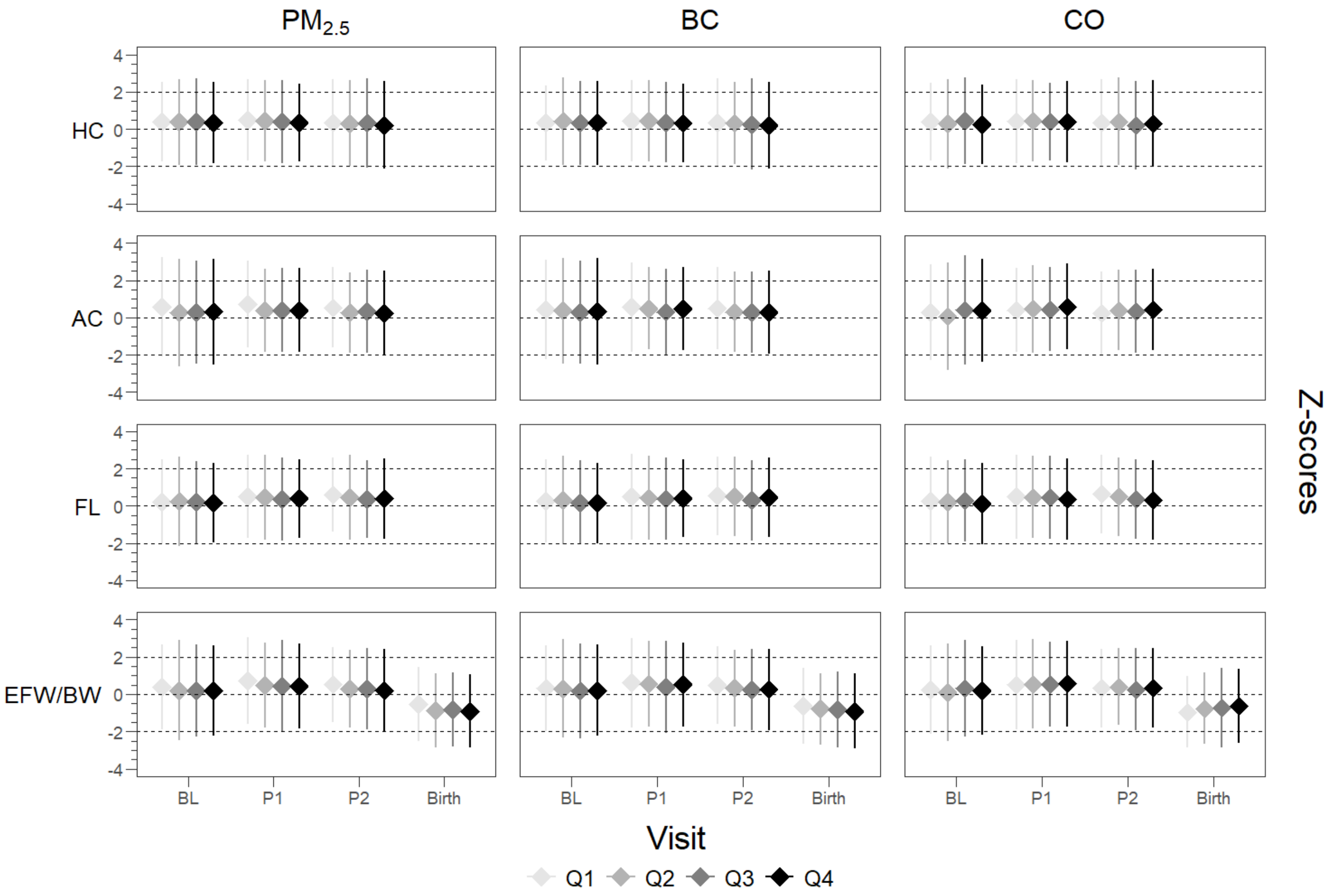

Figure S12. E-R visualization.

Figure-4

```
write.csv(tab7, "tab7.csv")
write.csv(tab7.trunc, "tab7.trunc.csv")
write.csv(tab7.lag, "tab7.lag.csv")

###   Figure-7

# child.var = "flz"
# exp.var = "bc"
# x.min = -0.2
# x.max = 0.2
# data = tab7
# y.title = "AC"

fun.fig7 <- function(child.var = "hcz",
                     exp.var = "pm",
                     x.min = -0.3,
                     x.max = 0.3,
                     data = tab7,
                     y.title = ""){

var <- paste0(child.var, "_", exp.var)
var.lb <- paste0(var, "_lb")
var.ub <- paste0(var, "_ub")

df.fig7 <- data[, c("group", var, var.lb, var.ub)]

if(child.var == "efwz"){
  df.fig7$group <- factor(df.fig7$group, levels = c("40", "33", "25"))
}

if(child.var != "efwz"){
  df.fig7$group <- factor(df.fig7$group, levels = c("33", "25"))
  df.fig7 <- df.fig7 %>% dplyr::filter(group %in% c("33", "25"))
}

#mean7 <- sprintf("%3.2f", round(df.fig7[[var]], 2))

mean7 <- NA
if(sprintf("%3.2f", round(abs(df.fig7[[var]][1]), 2)) == "0.00"){
```

```
  mean7[1] <- sprintf("%3.3f", round(df.fig7[[var]][1], 3))
}else{
  mean7[1] <- sprintf("%3.2f", round(df.fig7[[var]][1], 2))
}

if(sprintf("%3.2f", round(abs(df.fig7[[var]][2]), 2)) == "0.00"){
  mean7[2] <- sprintf("%3.3f", round(df.fig7[[var]][2], 3))
}else{
  mean7[2] <- sprintf("%3.2f", round(df.fig7[[var]][2], 2))
}

if(length(df.fig7[[var.ub]]) == 3){
if(sprintf("%3.2f", round(abs(df.fig7[[var]][3]), 2)) == "0.00"){
  mean7[3] <- sprintf("%3.3f", round(df.fig7[[var]][3], 3))
}else{
  mean7[3] <- sprintf("%3.2f", round(df.fig7[[var]][3], 2))
}
}


#lb7 <- sprintf("%3.2f", round(df.fig7[[var.lb]], 2))

ub7 <- NA
if(sprintf("%3.2f", round(abs(df.fig7[[var.ub]][1]), 2)) == "0.00"){
  ub7[1] <- sprintf("%3.3f", round(df.fig7[[var.ub]][1], 3))
}else{
  ub7[1] <- sprintf("%3.2f", round(df.fig7[[var.ub]][1], 2))
}

if(sprintf("%3.2f", round(abs(df.fig7[[var.ub]][2]), 2)) == "0.00"){
  ub7[2] <- sprintf("%3.3f", round(df.fig7[[var.ub]][2], 3))
}else{
  ub7[2] <- sprintf("%3.2f", round(df.fig7[[var.ub]][2], 2))
}

if(length(df.fig7[[var.ub]]) == 3){
if(sprintf("%3.2f", round(abs(df.fig7[[var.ub]][3]), 2)) == "0.00"){
  ub7[3] <- sprintf("%3.3f", round(df.fig7[[var.ub]][3], 3))
}else{
  ub7[3] <- sprintf("%3.2f", round(df.fig7[[var.ub]][3], 2))
}
```

```
}

lb7 <- NA
if(sprintf("%3.2f", round(abs(df.fig7[[var.lb]][1]), 2)) == "0.00"){
  lb7[1] <- sprintf("%3.3f", round(df.fig7[[var.lb]][1], 3))
}else{
  lb7[1] <- sprintf("%3.2f", round(df.fig7[[var.lb]][1], 2))
}

if(sprintf("%3.2f", round(abs(df.fig7[[var.lb]][2]), 2)) == "0.00"){
  lb7[2] <- sprintf("%3.3f", round(df.fig7[[var.lb]][2], 3))
}else{
  lb7[2] <- sprintf("%3.2f", round(df.fig7[[var.lb]][2], 2))
}

if(length(df.fig7[[var.lb]]) == 3){
if(sprintf("%3.2f", round(abs(df.fig7[[var.lb]][3]), 2)) == "0.00"){
  lb7[3] <- sprintf("%3.3f", round(df.fig7[[var.lb]][3], 3))
}else{
  lb7[3] <- sprintf("%3.2f", round(df.fig7[[var.lb]][3], 2))
}
}



#ub7 <- sprintf("%3.2f", round(df.fig7[[var.ub]], 2))

ub7[which(substr(df.fig7[[var.ub]], 1, 5) == "0.000")] <- "0.001"


df.fig7$lab <- paste0(mean7, " (",
                      lb7, " to ",
                      ub7, ")")

p <- ggplot(data = df.fig7)

if(child.var == "efwz"){
  p <- p + annotate("rect", xmin = -Inf, xmax = Inf, ymin = 1.5, ymax = 2.5,
           alpha = 0.3,fill = "grey")
}else{
  p <- p + annotate("rect", xmin = -Inf, xmax = Inf, ymin = -Inf, ymax = 1.5,
```

```
            alpha = 0.3,fill = "grey")
}

 p <- p +
  geom_point(aes(x = !!sym(var),
                 y = group),
             shape = "diamond",
             color = "black",
             position = position_dodge(width = 0.8),
             size = 7) +
  geom_errorbar(aes(y = group,
                    xmin = !!sym(var.lb),
                    xmax = !!sym(var.ub)),
                color = "black",
                position = position_dodge(width = 0.8),
                size = 1,
                width = 0) +
  geom_text(aes(y = group,
                x = Inf),
            size = 4.5,
            hjust = 1.04,
            label = df.fig7$lab) +
  scale_y_discrete(y.title) +
  geom_vline(aes(xintercept = 0), linetype = "dashed") +
  scale_x_continuous(limits = c(x.min, x.max),
                     breaks = seq(x.min, x.max, 0.1),
                     labels = c("-0.2", "-0.1", "0.0",
                                "0.1", "0.2"),
                     minor_breaks = seq(x.min, x.max, 0.02),
                     guide = "axis_minor") +
   coord_cartesian(xlim = c(-0.2, 0.4))

p <- p +
  theme_bw() +
  #guides(color = guide_legend(override.aes = list(size = 7, linelength = 9))) +
  theme(panel.grid = element_blank(),
        legend.position = "none",
        axis.title.x = element_blank(),
        axis.title.y = element_text(size = 18, vjust = 0.5, hjust = 0, angle = 0),
        axis.ticks.length = unit(0.3, "cm"),
        ggh4x.axis.ticks.length.minor = rel(0.5),
```

```
        legend.title = element_blank(),
        legend.key.width = unit(1,"cm"),
        axis.text = element_text(size = 14),
        plot.title = element_text(size = 22, hjust = 0.5))

if(child.var == "hcz" & exp.var == "pm"){p <- p + ggtitle(bquote(PM[2.5]))}
if(child.var == "hcz" & exp.var == "bc"){p <- p + ggtitle(bquote(BC[ ]))}
if(child.var == "hcz" & exp.var == "co"){p <- p + ggtitle(bquote(CO[ ]))}
if(exp.var != "pm"){p <- p + theme(axis.title.y = element_blank(),
                                      axis.text.y = element_blank(),
                                      axis.ticks.y = element_blank())}
if(child.var != "efwz"){p <- p + theme(axis.text.x = element_blank(),
                                       axis.ticks.x = element_blank())}

return(p)
}

y.title.list <- c("          HC", "          AC", "          FL", "EFW/BW")
exp.varlist7 <- c("pm", "bc", "co")
child.varlist7 <- c("hcz", "acz", "flz", "efwz")

for(ccc in 1:4){
  for(eee in 1:3){
    assign(paste0("p7_",
                  exp.varlist7[eee], "_",
                  child.varlist7[ccc]),

           fun.fig7(child.var = child.varlist7[ccc],
                    exp.var = exp.varlist7[eee],
                    data = tab7,
                    x.min = -0.2,
                    x.max = 0.2,
                    y.title = y.title.list[ccc])
           )
  }
}


p7_c1 <- plot_grid(p7_pm_hcz,
                   p7_pm_acz,
                   p7_pm_flz,
```

```
                      p7_pm_efwz, align = "v", nrow = 4, rel_heights = c(2.45, 2, 2, 3))

p7_c2 <- plot_grid(p7_bc_hcz,
                   p7_bc_acz,
                   p7_bc_flz,
                   p7_bc_efwz, align = "v", nrow = 4, rel_heights = c(2.45, 2, 2, 3))

p7_c3 <- plot_grid(p7_co_hcz,
                   p7_co_acz,
                   p7_co_flz,
                   p7_co_efwz, align = "v", nrow = 4, rel_heights = c(2.45, 2, 2, 3))



#p7 <- arrangeGrob(arrangeGrob(rbp7_c1, p7_c2, p7_c3, ncol=3), size = "last")

p7 <- plot_grid(p7_c1, p7_c2, p7_c3, ncol = 3, rel_widths = c(1.335, 1, 1))

g7 <- ggdraw() +
  draw_plot(p7, x = 0.01, y = 0.1, height = 0.90, width = 0.95) +
  #draw_plot(legend7, x = 0.5, y = 0.01, height = 0.1, width = 0.90, hjust = 0.5) +
  annotate("text", x = 0.5, y= 0.07, size = 8,
           label = "Z-score") +
  annotate("text", x = 0.99, y= 0.5, size = 8,
           label = "Gestational age (weeks)", angle = -90)


tiff("Figure-4.tiff",
     units="in", width = 15, height = 10, res=100, compression = 'lzw')
g7
invisible(dev.off())


setEPS()
postscript("Figure-4.eps", width = 15, height = 10)
g7
invisible(dev.off())
```

.

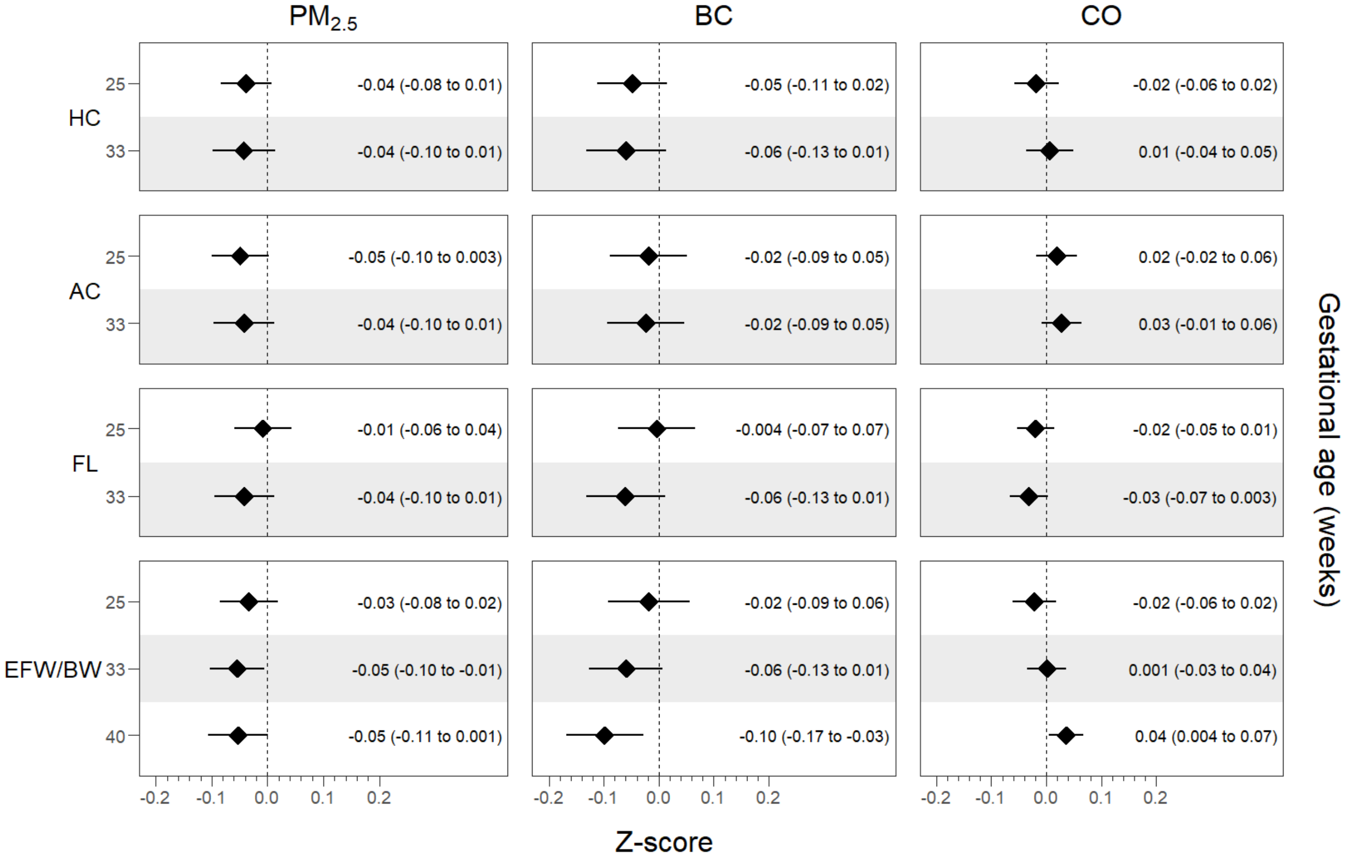

Figure 4. Exposure-response effects.

REFERENCES

1. Johnson MA, Steenland K, Piedrahita R, Clark ML, Pillarisetti A, Balakrishnan K, Peel JL, Naeher LP, Liao J, Wilson D, Sarnat J, Underhill LJ, Burrowes V, McCracken JP, Rosa G, Rosenthal J, Sambandam S, de Leon O, Kirby MA, Kearns K, Checkley W, Clasen T; HAPIN Investigators. Air Pollutant Exposure and Stove Use Assessment Methods for the Household Air Pollution Intervention Network (HAPIN) Trial. Environ Health Perspect 2020;128(4):47009.
2. Johnson M, Pillarisetti A, Piedrahita R, Balakrishnan K, Peel JL, Steenland K, Underhill LJ, Rosa G, Kirby MA, Díaz-Artiga A, McCracken J, Clark ML, Waller L, Chang HH, Wang J, Dusabimana E, Ndagijimana F, Sambandam S, Mukhopadhyay K, Kearns KA, Campbell D, Kremer J, Rosenthal JP, Checkley W, Clasen T, Naeher L; the Household Air Pollution Intervention Network (HAPIN) Trial Investigators. Exposure Contrasts of Pregnant Women during the Household Air Pollution Intervention Network Randomized Controlled Trial. Environ Health Perspect 2022;130(9):97005.
3. Nicolaou L, Underhill L, Hossen S, Simkovich S, Thangavel G, Rosa G, McCracken JP, Davila-Roman V, Fuentes LL, Quinn AK, Clark M, Diaz A, Pillarisetti A, Steenland K, Waller LA, Jabbarzadeh S, Peel JL, Checkley W; HAPIN Investigators. Cross-sectional analysis of the association between personal exposure to household air pollution and blood pressure in adult women: Evidence from the multicountry Household Air Pollution Intervention Network (HAPIN) trial. Environ Res 2022;214:114121.
4. Quinn AK, Williams KN, Thompson LM, Harvey SA, Piedrahita R, Wang J, Quinn C, Pillarisetti A, McCracken JP, Rosenthal JP, Kirby MA, Diaz Artiga A, Thangavel G, Rosa G, Miranda JJ, Checkley W, Peel JL, Clasen TF. Fidelity and Adherence to a Liquefied Petroleum Gas Stove and Fuel Intervention during Gestation: The Multi-Country Household Air Pollution Intervention Network (HAPIN) Randomized Controlled Trial. Int J Environ Res Public Health. 2021;18(23):12592.
